# Characterization of polyamine metabolism predicts prognosis, immune profile, and therapeutic efficacy in lung adenocarcinoma patients

**DOI:** 10.3389/fcell.2024.1331759

**Published:** 2024-04-08

**Authors:** Zhouhua Li, Yue Wu, Weichang Yang, Wenjun Wang, Jinbo Li, Xiaotian Huang, Yanqiang Yang, Xinyi Zhang, Xiaoqun Ye

**Affiliations:** ^1^ Department of Respiratory Diseases, The Second Affiliated Hospital, Jiangxi Medical College, Nanchang University, Nanchang, China; ^2^ Health Team, Jiangsu Marine Police Bureau, Nanjing, China

**Keywords:** polyamine, lung adenocarcinoma, molecular subtype, prognosis, immune

## Abstract

**Background:**

Polyamine modification patterns in lung adenocarcinoma (LUAD) and their impact on prognosis, immune infiltration, and anti-tumor efficacy have not been systematically explored.

**Methods:**

Patients from The Cancer Genome Atlas (TCGA) were classified into subtypes according to polyamine metabolism-related genes using the consensus clustering method, and the survival outcomes and immune profile were compared. Meanwhile, the geneCluster was constructed according to the differentially expressed genes (DEGs) of the subtypes. Subsequently, the polyamine metabolism-related score (PMRS) system was established using the least absolute shrinkage and selection operator (LASSO) multivariate regression analysis in the TCGA training cohort (*n* = 245), which can be applied to characterize the prognosis. To verify the predictive performance of the PMRS, the internal cohort (*n* = 245) and the external cohort (*n* = 244) were recruited. The relationship between the PMRS and immune infiltration and antitumor responses was investigated.

**Results:**

Two distinct patterns (C1 and C2) were identified, in which the C1 subtype presented an adverse prognosis, high CD8^+^ T cell infiltration, tumor mutational burden (TMB), immune checkpoint, and low tumor immune dysfunction and exclusion (TIDE). Furthermore, two geneClusters were established, and similar findings were observed. The PMRS, including three genes (SMS, SMOX, and PSMC6), was then constructed to characterize the polyamine metabolic patterns, and the patients were divided into high- and low-PMRS groups. As confirmed by the validation cohort, the high-PMRS group possessed a poor prognosis. Moreover, external samples and immunohistochemistry confirmed that the three genes were highly expressed in tumor samples. Finally, immunotherapy and chemotherapy may be beneficial to the high-PMRS group based on the immunotherapy cohorts and low half-maximal inhibitory concentration (IC_50_) values.

**Conclusion:**

We identified distinct polyamine modification patterns and established a PMRS to provide new insights into the mechanism of polyamine action and improve the current anti-tumor strategy of LUAD.

## Introduction

According to the 2022 cancer statistics, lung cancer is a common cause of cancer-related deaths worldwide, with the second-highest cancer incidence rate (Siegel et al., 2022). Among the numerous clinical subtypes of pulmonary cancer, lung adenocarcinoma (LUAD) attracts significant attention in the medical community owing to its high aggressiveness and lethality (Inamura, 2018). Although current treatment methods for LUAD, including chemotherapy, have shown excellent anti-cancer potential, a small percentage of patients still experience adverse reactions and present a poor survival prognosis. Therefore, an effective biomarker is urgently needed to assess the survival prognosis of patients and to realize personalized treatment schedules.

Natural polyamines, small polycationic molecules containing putrescine, spermidine, and spermine, are essential nutrients for eukaryotic cell growth (Bae et al., 2018) and contribute significantly to DNA replication, cell proliferation, and apoptosis (Kalac, 2014). In addition, the emergence and progression of tumors are closely correlated with disturbances in polyamine homeostasis (Casero et al., 2018). In this context, the modulation of polyamine synthase activity and polyamine transmembrane transport systems holds promise for targeted cancer therapy. For instance, the upregulation of ornithine decarboxylase (ODC), a critical rate-limiting enzyme in the polyamine synthesis pathway regulated by c-MYC transcription (Pegg, 2006), is an essential mechanism promoting colorectal cancer (CRC) development. Based on this, the combined blocking of ODC and eukaryotic translation initiation factor 5A (eIF5A) can effectively inhibit c-MYC and produce a synergistic anti-tumor effect in CRC (Coni et al., 2023). Similarly, 5′-methylthioadenosine phosphorylase (MTAP) plays a critical metastasis inhibitory role in breast cancer by inhibiting ODC activity (Zhang et al., 2022). As the second rate-limiting enzyme for polyamine synthesis, the S-adenosylmethionine decarboxylase proenzyme (AMD1) is mainly involved in the synthetic processes of spermidine and spermine. As reported, the activation of mTORC1, which is correlated with tumor proliferation, can induce an increase in the expression of AMD1 in prostate cancer cells (Zabala-Letona et al., 2017). Another study showed that AMD1 is critical for driving cancer stemness in myeloid leukemia and promoting the progression of chronic myeloid leukemia (Sari et al., 2021). Serving as an important rate-limiting enzyme in the process of polyamine catabolism, spermidine–spermine N^1^-acetyltransferase (SSAT) is important in the regulation of the cell cycle and DNA repair, whose mutation is followed by tumor migration and progression (Thakur et al., 2019). Notably, the activation of SSAT is controlled by p53, which can induce the ferroptotic response in lung cancer cells (Ou et al., 2016). However, the action mechanism of the polyamine metabolism patterns in lung cancer remains unclear and requires comprehensive analysis.

Currently, the correlation between tumor immunity and tumor metabolism is being studied extensively, suggesting that polyamine metabolism has shown great value in modulating the anti-tumor immunotherapy response, thus making it a potential target for immunotherapy. In general, malignant tumors have elevated levels of polyamines to assist the growth of cells with an immunosuppressive phenotype (Geiger et al., 2016; Gautam et al., 2023). In glioblastoma, immunosuppressive tumor-associated myeloid cells have higher polyamine levels than cytotoxic CD8^+^ T cells (Miska et al., 2021). Furthermore, ODC can inhibit the M1 macrophage responses associated with colitis and associated carcinogenesis (Singh et al., 2018). Thus, targeting polyamines may represent a novel strategy to remodel the tumor immune microenvironment (TME) to enhance anti-tumor responses, supporting ongoing research on polyamine inhibitors with potential TME-regulatory properties (Holbert et al., 2022). In particular, the overall impact of polyamine metabolism on the immunotherapeutic response to LUAD has not been adequately explored to date.

In summary, a comprehensive investigation of polyamine metabolism patterns and characteristics of polyamine-mediated TME cell infiltration is promising to efficiently assess the patient prognosis and benefit the search for new therapeutic targets. In this study, we divided 490 LUAD patients into two subtypes based on genes related to polyamine metabolism. Then, we found that survival outcomes and the immune profile were notably different in the two subtypes. To this end, we constructed a polyamine metabolism-related score (PMRS) system for the characterization of polyamine metabolism patterns, which could be a significant addition to the assessment of the clinical prognosis of LUAD patients and their sensitivity to immunotherapy and chemotherapy.

## Materials and methods

### Data collection

The transcriptome data, clinical and somatic mutation information about 490 LUAD patients, were gained from The Cancer Genome Atlas (TCGA) database (https://portal.gdc.cancer.gov/). External verification cohorts (GSE13213 and GSE50081), including 244 samples with LUAD, were derived from the Gene Expression Omnibus (GEO) database (https://www.ncbi.nlm.nih.gov/geo/). The copy number variation (CNV) data of LUAD patients was obtained from the UCSC Xena database (https://xenabrowser.net/datapages/). The gene set, REACTOME_METABOLISM_OF_ POLYAMINES, was attained from the MSigDB (http://www.gsea-msigdb.org/gsea/index.jsp), and then 59 polyamine metabolism-related genes were included for further analysis (Supplementary Table S1).

### Landscape of genetic variation and consensus clustering analysis

The polyamine metabolism-related gene expression was drawn from TCGA. Then, the Cox analysis was utilized to filter the prognosis-related genes. Meanwhile, multiple approaches were used to characterize the genetic diversity of prognosis-related genes. First, the mutation rate for each gene was calculated using the “maftools” package, and the “RCircos” package was utilized to display the CNV. Additionally, the correlation analysis was performed to elaborate on the interrelationship among prognostic genes. Finally, we exploited the differential expressions of prognostic genes by using the Wilcoxon test.

Subsequently, consensus clustering (Wilkerson and Hayes, 2010) (parameters: reps = 50, pItem = 0.8, pFeature = 1, clusterAlg = “pam,” and distance = “canberra”) was used to establish the molecular subtype. The “survival” package was used to investigate the prognosis between different groups. Moreover, the differential expressions of prognostic genes among different subtypes and their correlation with clinical characteristics were displayed by the “heatmap” package. We used the single-sample gene set enrichment analysis (ssGSEA) method to investigate 16 kinds of immune cells (Rooney et al., 2015). Furthermore, to assess the association between various subtypes and the effectiveness of immunotherapy, we compared some widely reported therapeutic predictors for the response to the immune checkpoint blockade (ICB), which include the expression of immune checkpoint, tumor mutational burden (TMB), and tumor immune dysfunction and exclusion (TIDE) scores among different subtypes. The TMB score was derived from somatic mutation data, and the TIDE score was obtained from the TIDE website (http://tide.dfci.harvard.edu/).

### Construction and analysis of geneCluster groups

The differentially expressed genes (DEGs) between different subtypes were discerned using the “limma” package. |logFC| > 1 and adjusted *p*-value <0.01 were considered statistically significant. Then, the Cox regression was exploited to filter out DEGs correlated with prognosis. The patients were categorized into different geneCluster groups on the basis of survival-related genes by applying the consensus clustering method. Using the same method mentioned earlier, we compared the immune landscape and immunotherapy response prediction between different geneCluster groups.

### Establishment and verification of the PMRS system

First, we applied the “caret” package to divide the TCGA LUAD cohort into a training cohort and a test cohort at a ratio of 1:1. Afterward, we used the least absolute shrinkage and selection operator (LASSO) to further compress the correlation coefficient to identify subsequent genes according to the polyamine metabolism-related prognostic genes in the training cohort. We used 1,000-fold alteration and cross-validation to ensure the stability of the screening results. Furthermore, the genes were sequentially subjected to multivariate Cox analysis. Finally, the PMRS also called the risk score, was constructed using a previously reported formula: PMRS = Σ(corresponding coefficient × gene expression). The patients were separated into two groups according to the median PMRS. Then, the prognosis of each patient group was evaluated using the “survival” package, while the accuracy of the PMRS in predicting 1-, 3-, and 5-year survival rates was assessed using the “timeROC” package. In addition, the PMRS prognostic significance was validated using both the internal cohort, including the TCGA test and all cohorts, and the external cohort, including the GSE13213 and GSE50081 cohorts, according to the same method used in the TCGA training cohort.

Subsequently, the GSE46539 cohort, containing 92 lung adenocarcinoma samples and 92 matched normal lung tissue samples, was used to validate the gene expression profile of the PMRS. The “pROC” package was used to estimate the efficacy of each gene to differentiate the tumor. Moreover, the individuals were categorized into two cohorts according to the median gene expression levels. Then, an overall survival analysis was conducted to examine the prognosis using the GEPIA dataset (http://gepia.cancer-pku.cn/). The immunohistochemical results of each gene from the HPA database are applied to verify the gene expression difference between the LUAD tissues and normal tissues (https://www.proteinatlas.org/). The antibody information of each gene applied for immunohistochemical analysis is available in Supplementary Table S2. The immunohistochemical protocol was performed as previously reported in the literature (Uhlen et al., 2015).

### Comprehensive analysis of the PMRS

We used a series of methods to evaluate the clinical significance of the PMRS. The correlation between PMRS and clinical features, including age, gender, pathological M, pathological N, pathological T, and pathological stage, was investigated using the Wilcoxon test. Meanwhile, the prognosis between different PMRS groups in clinical subgroups was evaluated with a survival analysis. Then, we investigated the correlation among the PMRS signature, cluster subgroup, and geneCluster group. The “survcomp” package was used to calculate the concordance index (C-index) of each signature to compare the predictive performance.

Subsequently, we used the gene set variation analysis (GSVA) package to explore the functional pathways enriched in various PMRS groups by using “h.all.v7.5.1.symbols.gmt.” The independent prognostic value of the PMRS was ascertained using the univariate and multivariate Cox analyses. To demonstrate the odds of survival, the PMRS, and other clinical factors were applied to construct the nomogram. Then, the area under the curve (AUC) and Cox analysis were utilized to assess the clinical application value.

### Immune infiltration characterization, immunotherapy response, and chemotherapeutic drug efficacy evaluation

The ssGSEA method was used to investigate 16 kinds of immune cell infiltrations and to evaluate the activation of 13 immune-related functional pathways between the two PMRS groups (Ye et al., 2021). Additionally, microenvironment cell population data were also used to observe the correlation between the PMRS and eight kinds of immune cells and two kinds of stroma cells using the “MCPcounter” package (Becht et al., 2016). Furthermore, a series of algorithms, including quanTIseq, CIBERSORT, and xCELL, were used to explore the relationship between the PMRS and CD8^+^ T cell infiltration.

Then, we also explored the common biomarkers of immunotherapy responsiveness among the various PMRS groups. First, the immune checkpoint expression levels, including PDCD1 (programmed cell death protein 1[PD-1]) and CD274 (programmed cell death ligand 1[PD-L1]), were compared between the different groups. Second, the “maftools” package was exploited to characterize the mutation of the top 20 genes between different PMRS groups. Moreover, we also compared the distribution of the TMB and TIDE scores between different groups. Furthermore, in order to verify the good performance of the PMRS in predicting the immunotherapy response, the three immunotherapy cohorts, namely, melanoma treated with an anti-PD-1 antibody (GSE78220), non-small-cell lung cancer (NSCLC) treated with an anti-PD-1 antibody (GSE126044), and anti-PD-1/PD-L1 antibody (GSE135222), were applied to evaluate the proportion of treatment respondents in different PMRS groups. Ultimately, the “pRRophetic” package (Geeleher et al., 2014) was used to assess the PMRS in predicting diverse group treatment responses to common chemotherapy drugs. The half-maximal inhibitory concentration (IC_50_) data for chemotherapy drugs were obtained from the Cancer Genome Project (CGP) database (https://www.sanger.ac.uk/group/cancer-genome-project/).

### Statistical analysis

Data analysis was performed using R software in the study. The statistical significance of measurement data conforming to the normal distribution was defined with the *t*-test, while the non-normal distribution data were analyzed by the Wilcoxon rank test. The “survival” package was utilized to carry out all survival analyses by the Kaplan–Meier procedure. The relationship between molecular subtype, geneCluster groups, and clinical features was assessed by chi-squared test. The cutoff for statistical significance was set as *p* < 0.05.

## Results

### Genetic variation depiction of polyamine metabolism-related genes in LUAD

**FIGURE 1:**
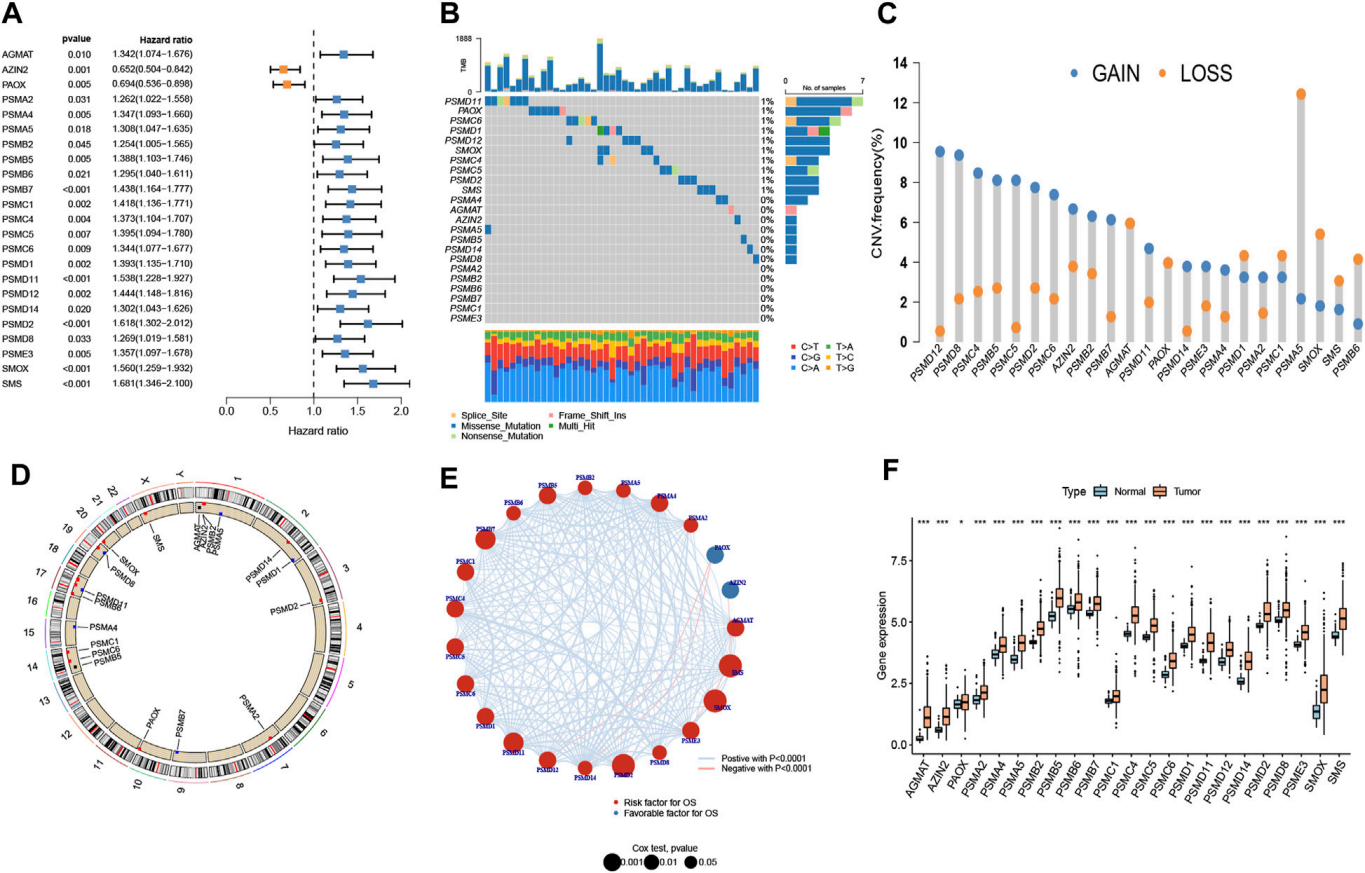
Landscape of genetic variation. **(A)** Prognosis-related genes using Cox regression analysis. **(B)** A mutation map of polyamine metabolism-related genes. **(C)** Copy number variation (CNV) of polyamine metabolism-related genes. **(D)** Position of the CNV on chromosomes. **(E)** Interplay and prognostic significance of polyamine metabolism-related genes. **(F)** Difference in the gene expression level between normal and tumor groups. *p < 0.05 and ***p < 0.001.

The 23 prognosis-related genes for further analysis were obtained from polyamine metabolism-related genes by using Cox regression analysis (Figure 1A). In total,10 polyamine metabolism-related genes presented gene mutations, mainly missense mutations (Figure 1B). The CNV amplification and deletion frequencies are shown in Figure 1C. The position of CNV on the chromosome is shown in Figure 1D. The result of the correlation analysis showed that prognosis-related genes had complicated interactions (Figure 1E). The upregulation of polyamine metabolism-related genes was displayed in tumor samples (Figure 1F). All the above-mentioned results manifested that genetic variation in polyamine metabolism-related genes played a vital role in the tumorigenesis and progression of LUAD.

### Distinct molecular subtype establishment and comprehensive analysis

**FIGURE 2:**
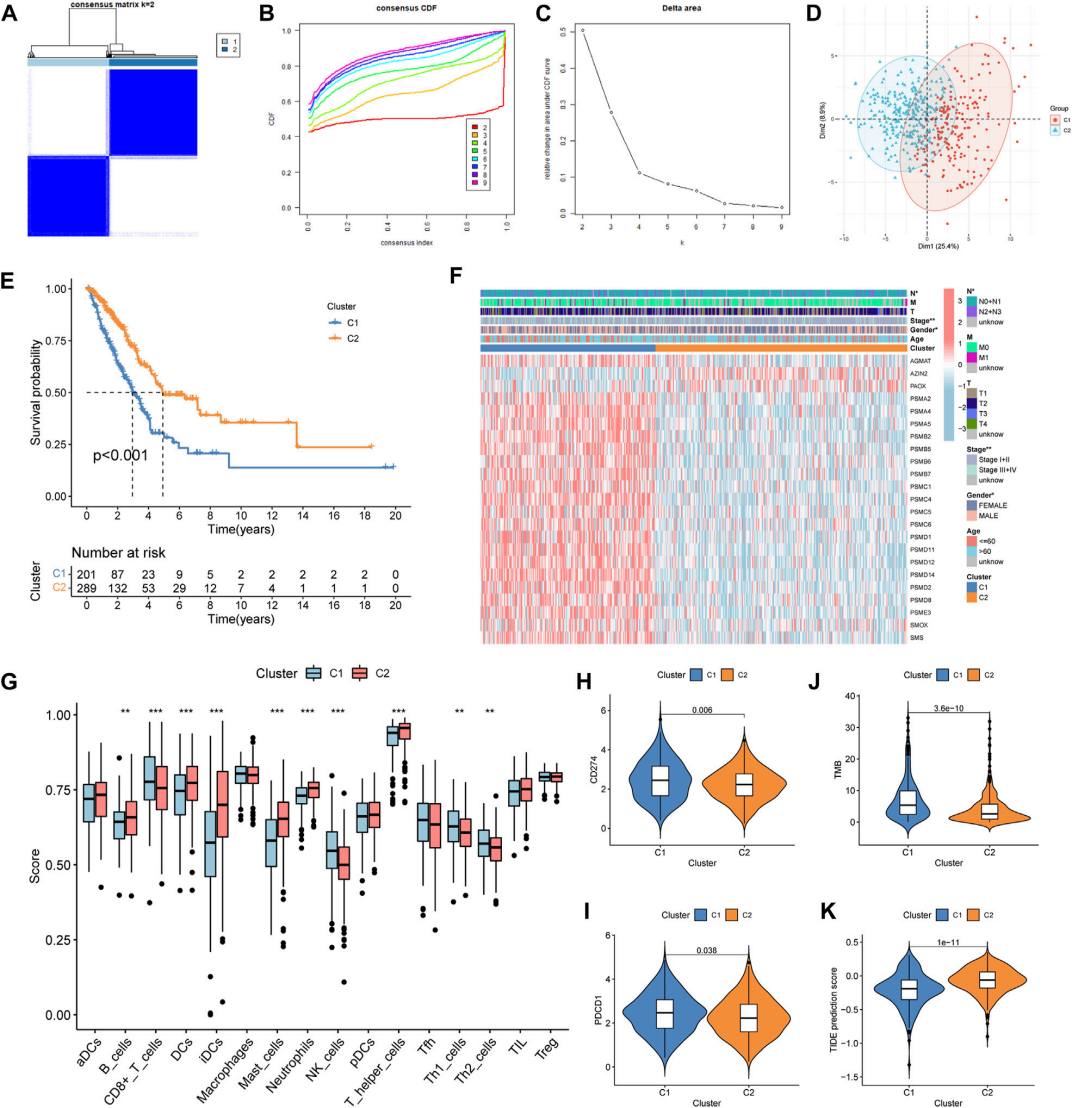
Construction, clinical features, immune profile, and immunotherapy response investigation of the subtype. **(A–C)** Optimal value of consensus clustering. **(D)** Principal component analysis (PCA). **(E)** Difference in the survival outcomes between the cluster 1 (C1) and cluster 2 (C2) subtypes. **(F)** Heatmap of genes and clinicopathological characteristics between the two subtypes. **(G)** Correlation analysis between the two subtypes and immune cell infiltration. **(H, I)** Distribution of CD274 and PDCD1 in the two subtypes. **(J)** Distribution of the tumor mutation burden (TMB). **(K)** Distribution of tumor immune dysfunction and exclusion (TIDE). *p < 0.05; **p < 0.01; and ***p < 0.001.

According to the prognosis-related genes, the consensus clustering separated LUAD patients into two different subtypes: cluster 1 (C1) and cluster 2 (C2) (Figures 2A–C). The principal component analysis (PCA) presented consensus clustering that separated patients into two subtypes (Figure 2D). The C1 subtype had 201 LUAD patients, and the C2 subtype had 289 patients. The results of the survival analysis disclosed that patients in the C2 group had better survival prognoses than those in the C1 group (Figure 2E). The clinical correlation analysis showed that the ratio of advanced stage (stages III + IV) and lymph node metastasis (pathological N2 + N3) was positive for the C1 subtype (Figure 2F). The heatmap indicated that genes related to polyamine metabolism were more active in the C1 subtype than in the C2 subtype, except for AZIN2 and PAOX (Figure 2F). Immune cell infiltration scoring showed that B cells, DCs, iDCs, mast cells, neutrophils, and T helper cells were more active in the C2 subtype, while the C1 subtype exhibited higher activity in CD8^+^ T cells, NK cells, Th1 cells, and Th2 cells (Figure 2G). On this basis, we could speculate that the two molecular subtypes may have markedly different immune cell infiltrations. Subsequently, we investigated the immunotherapeutic response markers in two subtypes. The expression levels of two checkpoints recommended in the guidelines, CD274 and PDCD1, were upregulated in the C1 subtype (Figures 2H, I). Additionally, compared to the C2 subtype, patients in the C1 subtype had higher TMB and TIDE scores (Figures 2J, K). Moreover, compared to the C2 subtype, we found that the proportion of patients with high-TMB and low-TIDE scores was higher in the C1 subtype after the patients were further divided into low- and high-score groups according to the median score (Supplementary Figures S1A, B).

### GeneCluster construction and overall analysis

**FIGURE 3:**
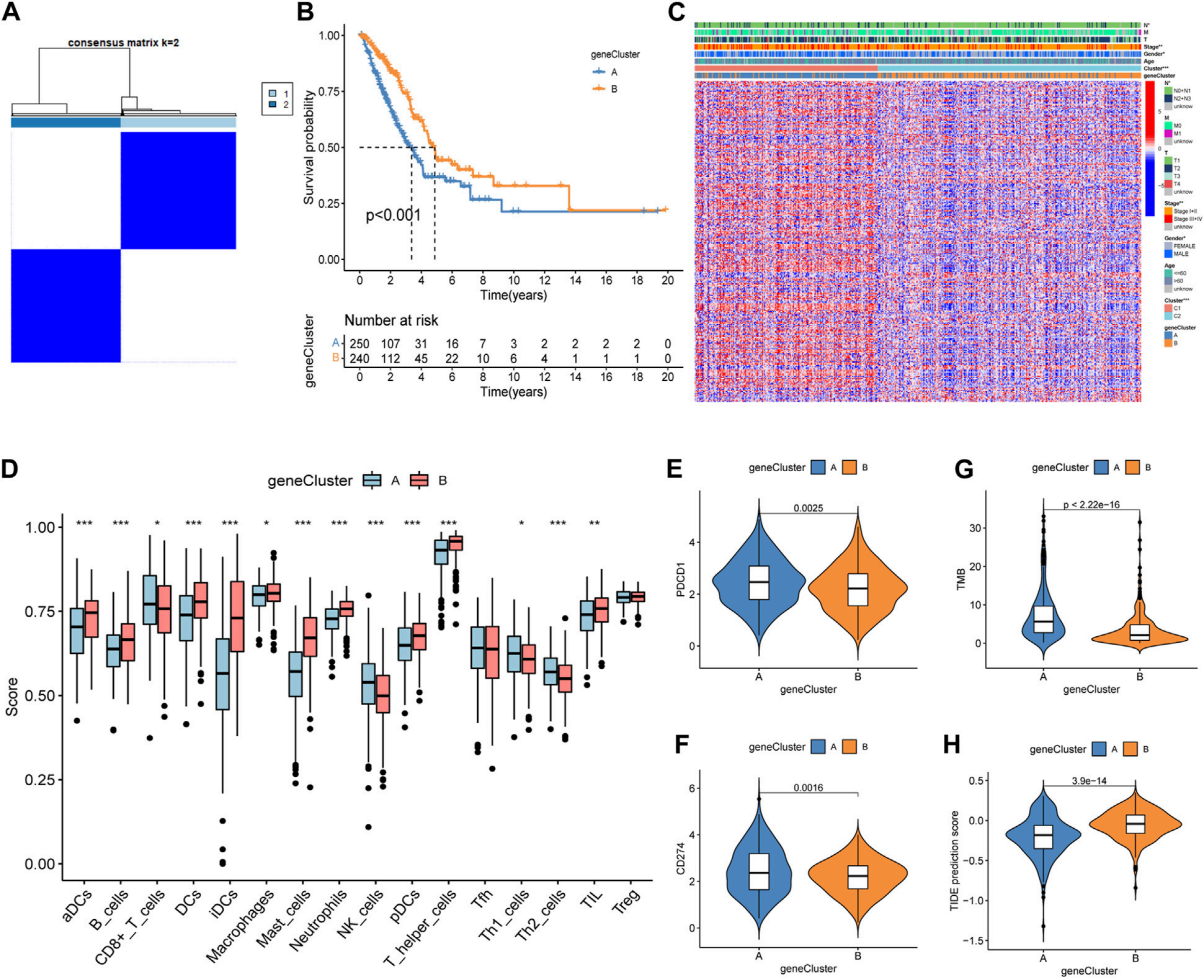
Identification, clinical characteristics, immune profile, and immunotherapy response analysis of the geneCluster. **(A)** Optimal value of consensus clustering. **(B)** Variation in survival outcomes between geneCluster A and geneCluster B. **(C)** Heatmap of genes and clinicopathological characteristics between the two clusters. **(D)** Correlation analysis between the two subtypes and immune cell infiltration. **(E, F)** Distribution of PDCD1 and CD274. **(G)** Distribution of the TMB. **(H)** Distribution of TIDE. *p < 0.05; **p < 0.01; and ***p < 0.001.

A total of 2,576 DEGs were identified between the two subtypes, i.e., 1,816 upregulated genes and 760 downregulated genes (Supplementary Table S3). Then, a univariate Cox regression analysis was used to identify 297 prognosis-related genes (Supplementary Table S4). Two geneCluster groups were constructed according to prognosis-related genes (Figure 3A). Based on the survival analysis, the geneCluster A group exhibited a poorer survival outcome than the geneCluster B group (Figure 3B). Patients in the geneCluster A group were characterized as advanced stage (stages III + IV), lymph node metastasis (pathological N2 + N3), and the C1 molecular subtype (Figure 3C). Furthermore, compared to the geneCluster B group, the genes associated with prognosis showed higher activation in the geneCluster A group (Figure 3C). All of the above-mentioned results suggested that the geneCluster A group was correlated with the C1 subtype. Then, we also investigated the trait of immune cell infiltration and immunotherapeutic response markers. The CD8^+^ T cells, NK cells, Th1 cells, and Th2 cells were activated in the geneCluster A group, while B cells, DCs, iDCs, macrophages, mast cells, neutrophils, pDCs, T helper cells, and TIL were activated in the geneCluster B group (Figure 3D). Upregulation of checkpoints and higher TMB and TIDE scores were observed in the geneCluster A group (Figures 3E–H). The geneCluster A group presented a greater proportion of a high TMB score (24% vs. 7%) and a low TIDE score (69% vs. 34%) (Supplementary Figures S2A, B). In summary, individuals belonging to the geneCluster A group might exhibit favorable reactions to immunotherapy.

### Polyamine metabolism-related score system development and validation

**FIGURE 4:**
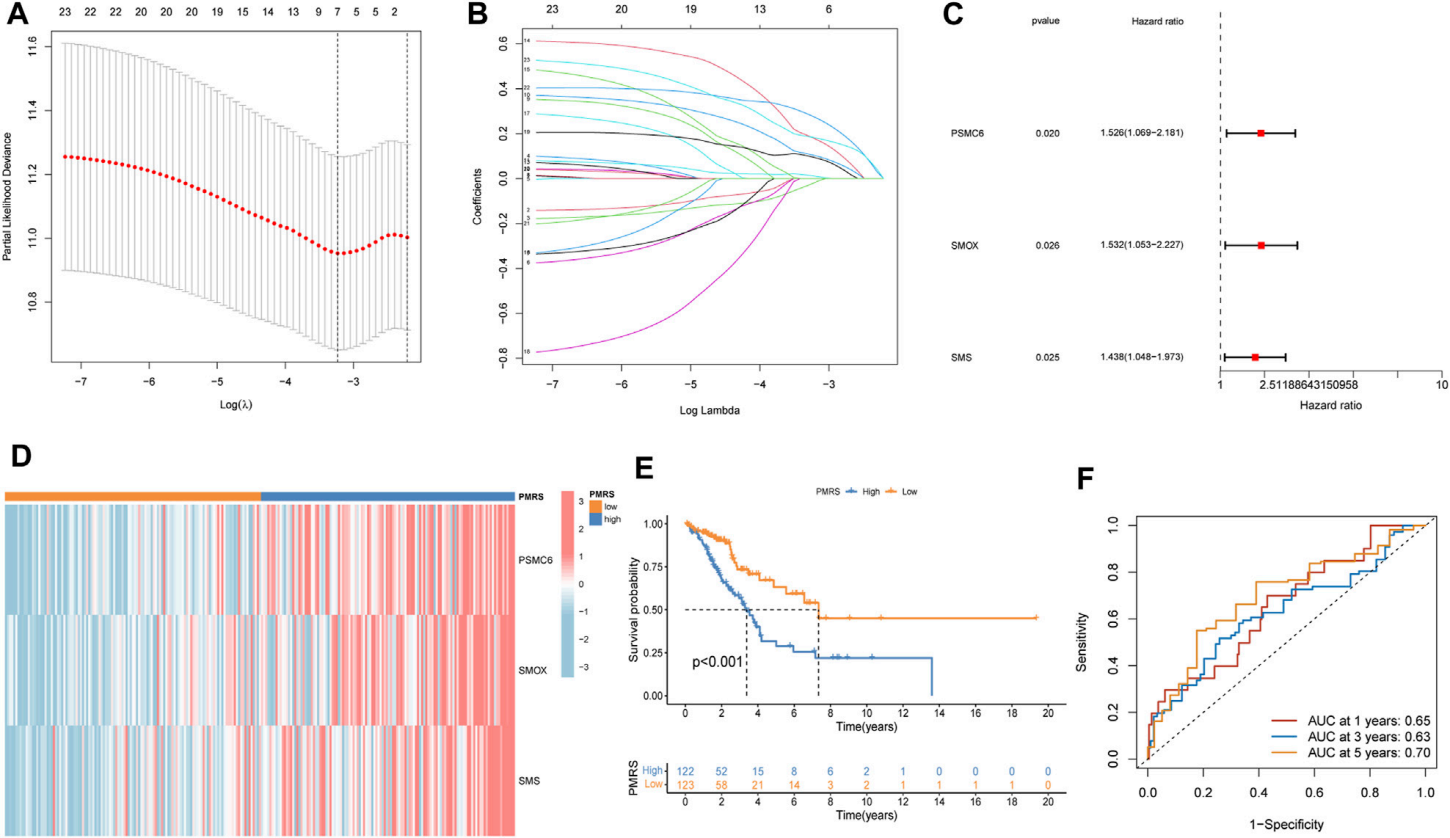
Establishment of the polyamine metabolism-related score (PMRS) based on the Cancer Genome Atlas (TCGA) training cohort. **(A, B)** Seven genes using LASSO regression analysis. **(C)** Three genes using multivariate Cox regression. **(D)** Heatmap of the distribution of the three genes. **(E)** Difference in the survival outcomes between the two groups. **(F)** Area under the curve (AUC) for predicting 1-, 3-, and 5-year survival rates.

**FIGURE 5:**
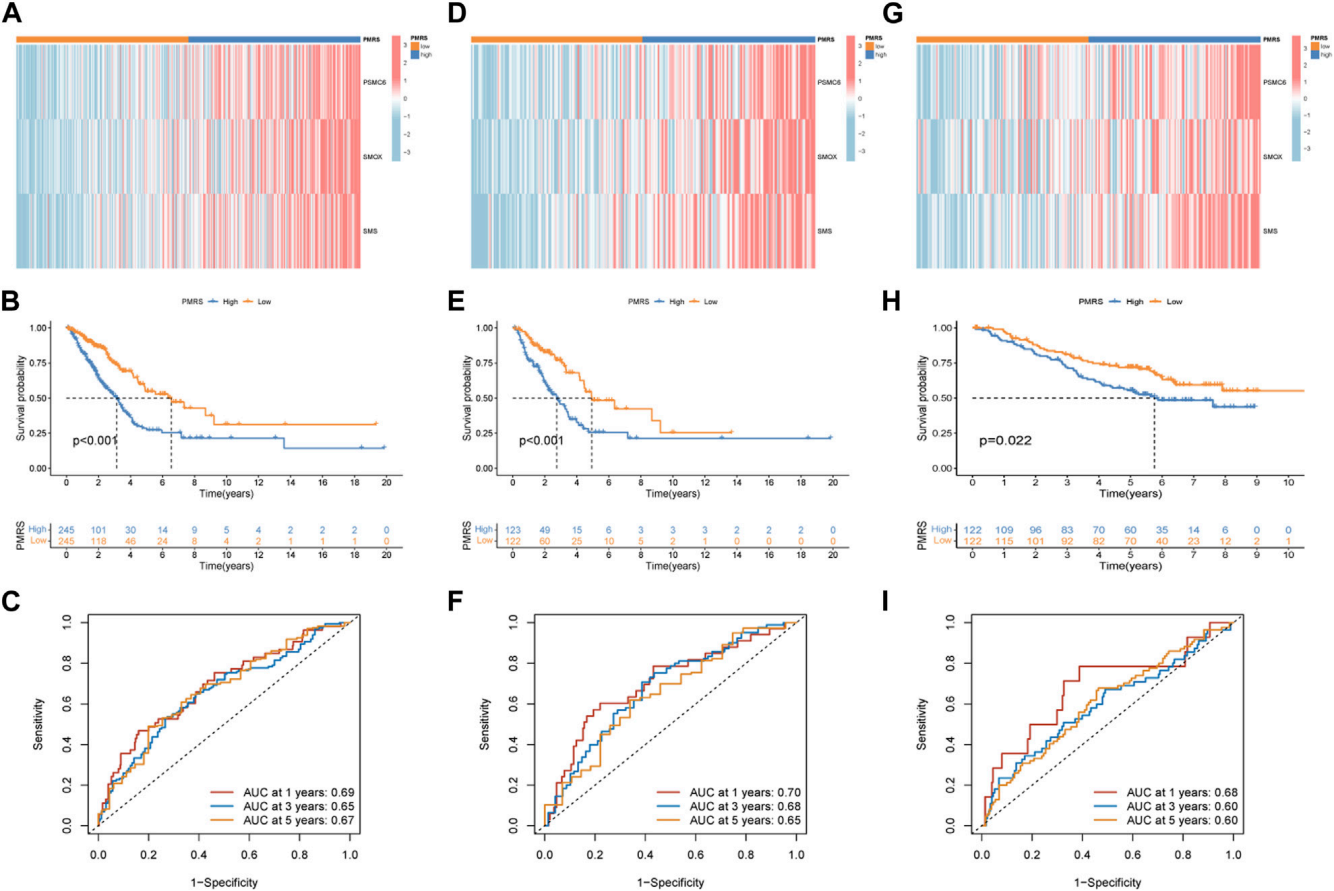
Validation of the PMRS in the internal and external cohorts. **(A)** Heatmap of the distribution of the three genes. **(B)** Survival difference analysis. **(C)** AUC for predicting 1-, 3-, and 5-year survival rates. The same analysis was performed in the TCGA test cohort **(D–F)** and the GSE13213 and GSE50081 lung adenocarcinoma (LUAD) cohorts **(G–I)**.

The entire TCGA cohort was randomly divided into the training cohort (*n* = 245) and the test cohort (*n* = 245). The polyamine metabolism-related score system was constructed according to the training cohort. Based on the previous regression analysis, 23 genes were selected for the follow-up study. Subsequently, LASSO regression was applied to eliminate collinearity (Tibshirani, 1996). Seven polyamine metabolism-related genes were selected after 1,000-time cross-validation (Figures 4A, B, Supplementary Table S5). Finally, three genes were chosen to establish the polyamine metabolism-related score after the multivariate regression analysis (Figure 4C). The polyamine metabolism-related score, also called the PMRS model, was calculated according to the following equation: 0.4229 * *PSMC6* (mRNA expression level) + 0.4262 * *SMOX* (mRNA expression level) + 0.3632 * *SMS* (mRNA expression level). The corresponding coefficient was generated by multivariate regression analysis (Supplementary Table S6). Based on the median PMRS, the TCGA training cohort could be categorized into high- and low-PMRS groups (Supplementary Figures S3A–C). The heatmap indicated that the high-PMRS group exhibited greater activity of the three prognostic genes related to polyamine metabolism than the low-PMRS group (Figure 4D). A total of 122 patients were included in the high-PMRS group, and the low-PMRS group consisted of 123 patients. In addition, the patients in the high-PMRS group had poorer survival prognoses than those in the low-PMRS group (Figure 4E). The AUC of 1-, 3-, and 5-year survival rates was 0.65, 0.63, and 0.70, respectively (Figure 4F), which indicated that the polyamine metabolism-related score had a good performance in predicting the prognosis of LUAD patients. Moreover, the TCGA internal cohort and external cohort were applied to confirm the predictive efficiency of the PMRS. The same method was used to divide the LUAD patients into the high- and low-PMRS groups. The patients could be clearly divided into two distinct groups in three cohorts using the same formula (Supplementary Figures S4A–C, D–F, G–I). The heatmap also showed that the three prognostic polyamine metabolism-related genes were activated in the high-PMRS group (Figures 5A, D, G). Compared to the high-PMRS group, the survival analysis manifested that the patients presented more favorable prognoses in the low-PMRS group (Figures 5B, E, H). The AUC of 1-, 3-, and 5-year survival rates was 0.69, 0.65, and 0.67 in the entire TCGA cohort, 0.70, 0.68, and 0.65 in the TCGA test cohort, and 0.68, 0.60, and 0.60 in the external GEO cohort, respectively (Figures 5C, F, I). All the above results demonstrated that the polyamine metabolism-related score had good effectiveness in predicting LUAD patient prognosis.

### Comprehensive analysis of the polyamine metabolism-related score system

**FIGURE 6:**
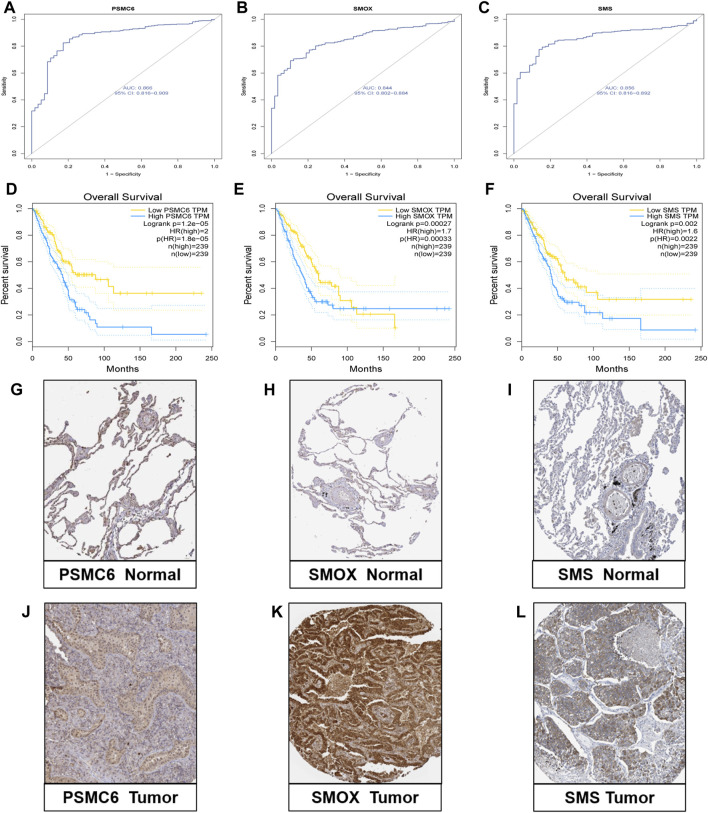
Validation research of the three genes. **(A–C)** Diagnostic ROC curve of the three genes. **(D–F)** Correlation analysis between expression levels of the three genes and survival outcomes. **(G–L)** Protein level of three genes according to immunohistochemical results.

We comprehensively explored the three genes in the model. First, in the GSE46539 lung adenocarcinoma cohort, the expression level of *PSMC6*, SMOX, and SMS was higher in the tumor samples than in the normal samples (Supplementary Figure S5), which was consistent with the previous result in TCGA lung cancer samples. Then, the diagnostic ROC curve was applied to evaluate the efficiency of correctly distinguishing between patients and non-patients. The AUC of PSMC6, SMOX, and SMS were 0.866, 0.844, and 0.856, respectively (Figures 6A–C), indicating that the three genes might have good performance in distinguishing between lung cancer patients and non-patients. The patients with high expression of PSMC6, SMOX, and SMS had adverse prognoses based on the data obtained from the GEPIA dataset (Figures 6D–F). The result was consistent with that of the patients in the high-PMRS group, who had more activated polyamine metabolism-related genes but a worse survival prognosis. The immunohistochemical analysis results show that the protein level of PSMC6, SMOX, and SMS was higher in the LUAD sample than in normal tissues according to the HPA database (Figures 6G–L).

**FIGURE 7:**
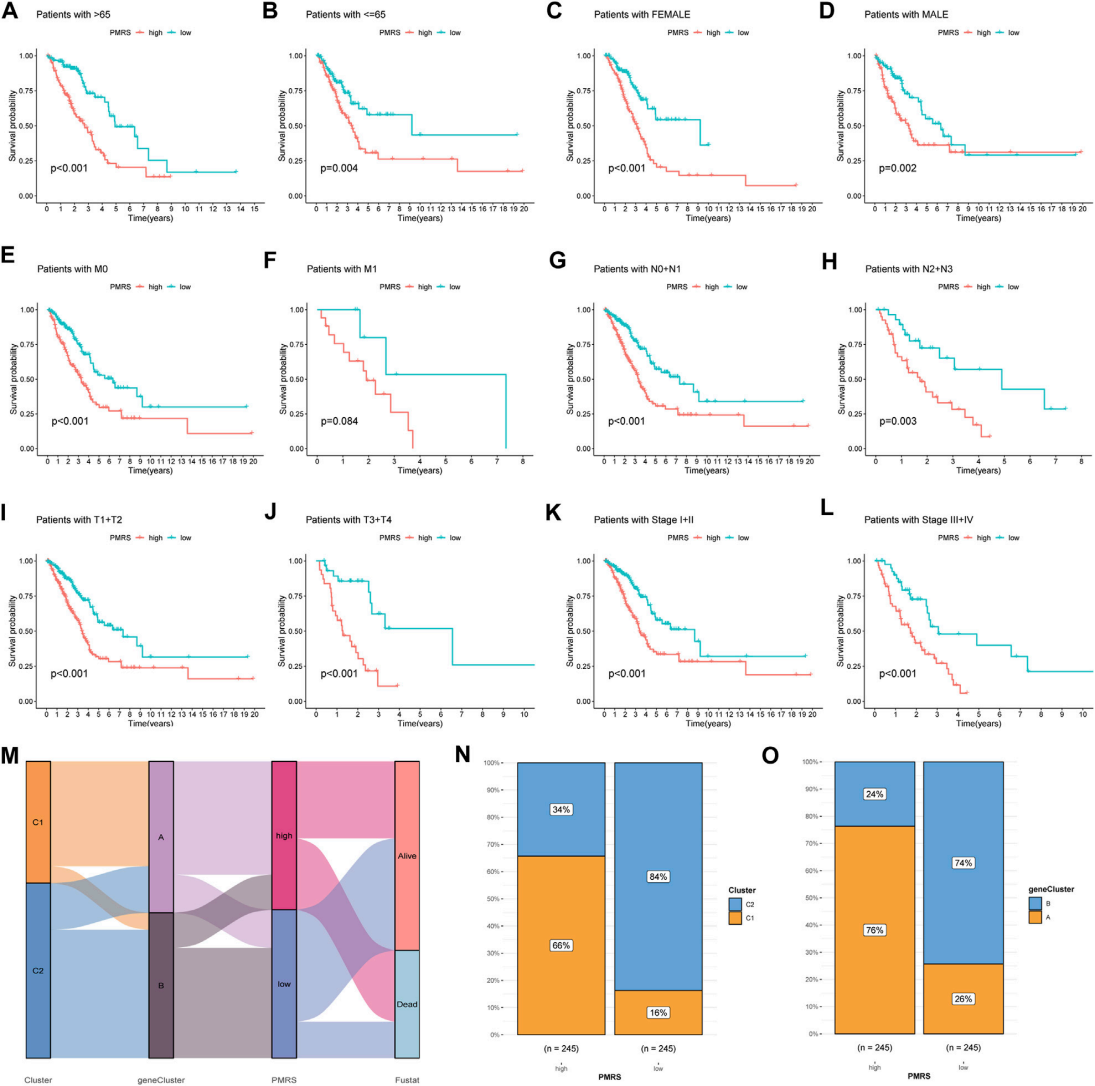
Correlation analysis between the PMRS, survival rate in different clinical subgroups, and polyamine modification patterns. **(A–L)** Survival rate of the high- and low-PMRS groups within various clinical subgroups. **(M)** Sankey diagram of the association between PMRS, clusters, geneClusters, and survival status. The cluster **(N)** and geneCluster **(O)** distribution in the two PMRS groups.

Subsequently, we examined the association between the PMRS and clinical features. The PMRS was high in patients with lymph node metastasis (pathological N2 + N3) and advanced stage (stages III + IV) (Supplementary Figure S6). The patients were categorized into high- and low-PMRS groups with the same method in different clinical subgroups. The individuals belonging to the high-PMRS group were observed to have a poorer prognosis in most clinical subgroups except for the tumor metastatic group (M1) (Figures 7A–L). The Sankey figure presented a close correlation between the C1 subtype, geneCluster A, and high PMRS (Figure 7M). All patients in these groups had a high death rate, which was identical to previous findings. The higher distribution of the C1 subtype and geneCluster A was found in the high-PMRS group (Figures 7N, O). By reviewing the previous study, the signature constructed by Wang et al. (2023) was compared with the PMRS. We found that the AUC at 1-, 3-, and 5-year survival rates (0.68, 0.68, and 0.66, respectively) and C-index (0.664 vs. 0.642) were similar to the PMRS (Supplementary Figures S7A, B), suggesting that the two score systems had similar efficiencies in estimating the prognosis of LUAD patients.

**FIGURE 8:**
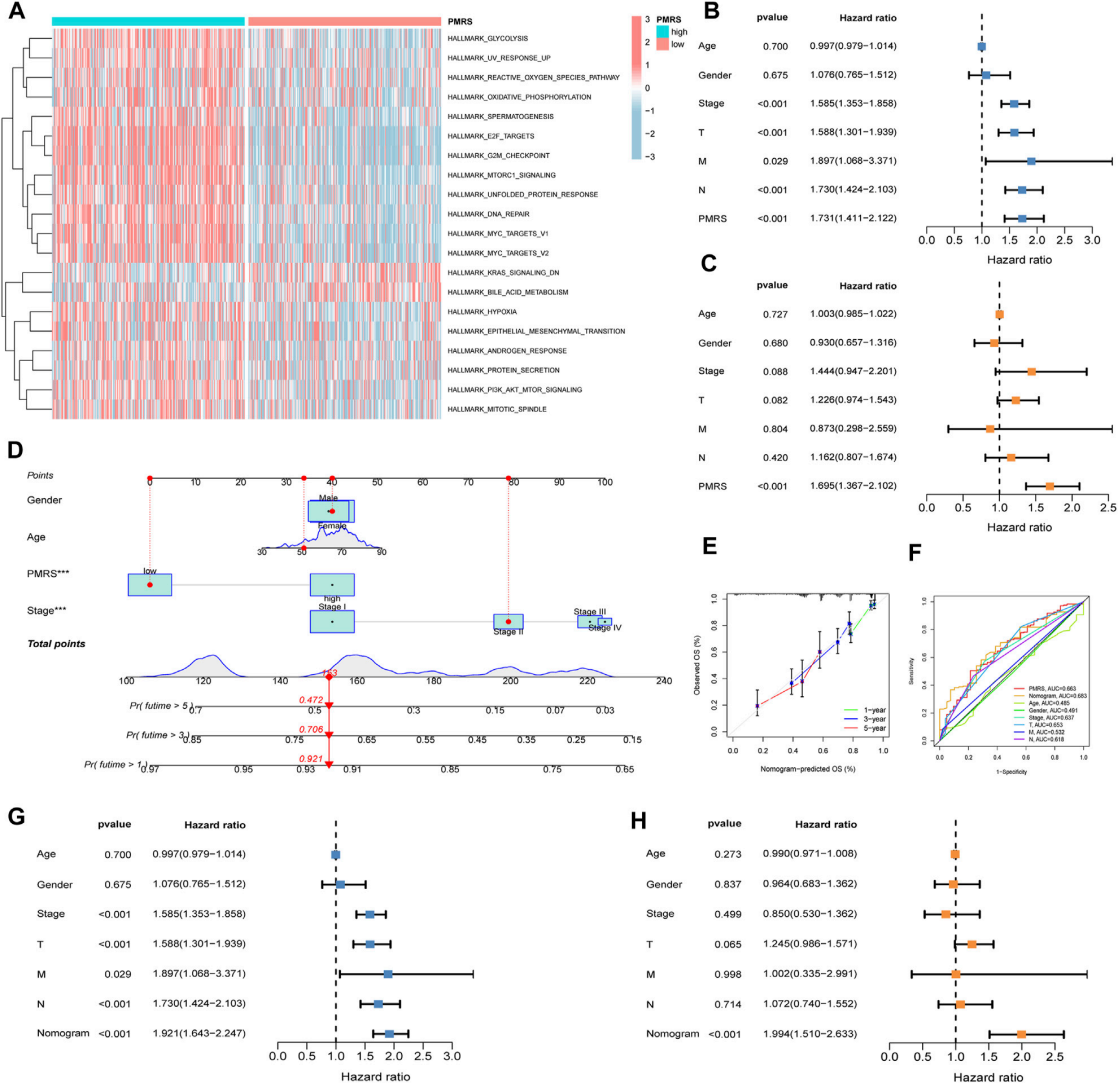
Comprehensive investigation of the PMRS. **(A)** Functional enrichment analysis in the high- and low-PMRS groups. Univariate **(B)** and multivariate analyses **(C)** of the PMRS and clinical factors. **(D)** Construction of the nomogram. **(E)** Calibration curve of the nomogram. **(F)** AUC for predicting the survival rate. Univariate **(G)** and multivariate analyses **(H)** of the nomogram and clinical factors.

Furthermore, functional enrichment analysis revealed that activated pathways were mainly cancer-related in the high-PMRS group, such as E2F_targets, G2M_checkpoint, MYC_targets_V1, MYC_targets_V2, EPITHELIAL_MESENCHYMAL_TRANSITION, MITOTIC spindle, and PI3K_AKT_MTOR_signaling (Figure 8A).

Finally, the clinical predictive value of the score was evaluated. Both the univariate and multivariate analyses confirmed that the PMRS was an independent marker for predicting survival prognosis (Figures 8B, C). In order to comprehensively predict patient prognosis, a nomogram was constructed using PMRS and other clinical factors. Figure 8D shows that the 1-, 3-, and 5-year overall survival probabilities were 0.921, 0.706, and 0.472, respectively. The 1-, 3-, and 5-year predicted overall survival probabilities were almost identical to the actual overall survival probabilities, suggesting the excellent efficacy of the scoring system in estimating LUAD patient prognosis (Figure 8E). Moreover, the result of the ROC curve showed that the nomogram had better performance in predicting the prognosis than other indices (Figure 8F). The nomogram was found to be a reliable survival prognostic indicator according to the univariate and multivariate analyses (Figures 8G, H).

### Immune infiltration characterization and therapeutic efficacy prediction

**FIGURE 9:**
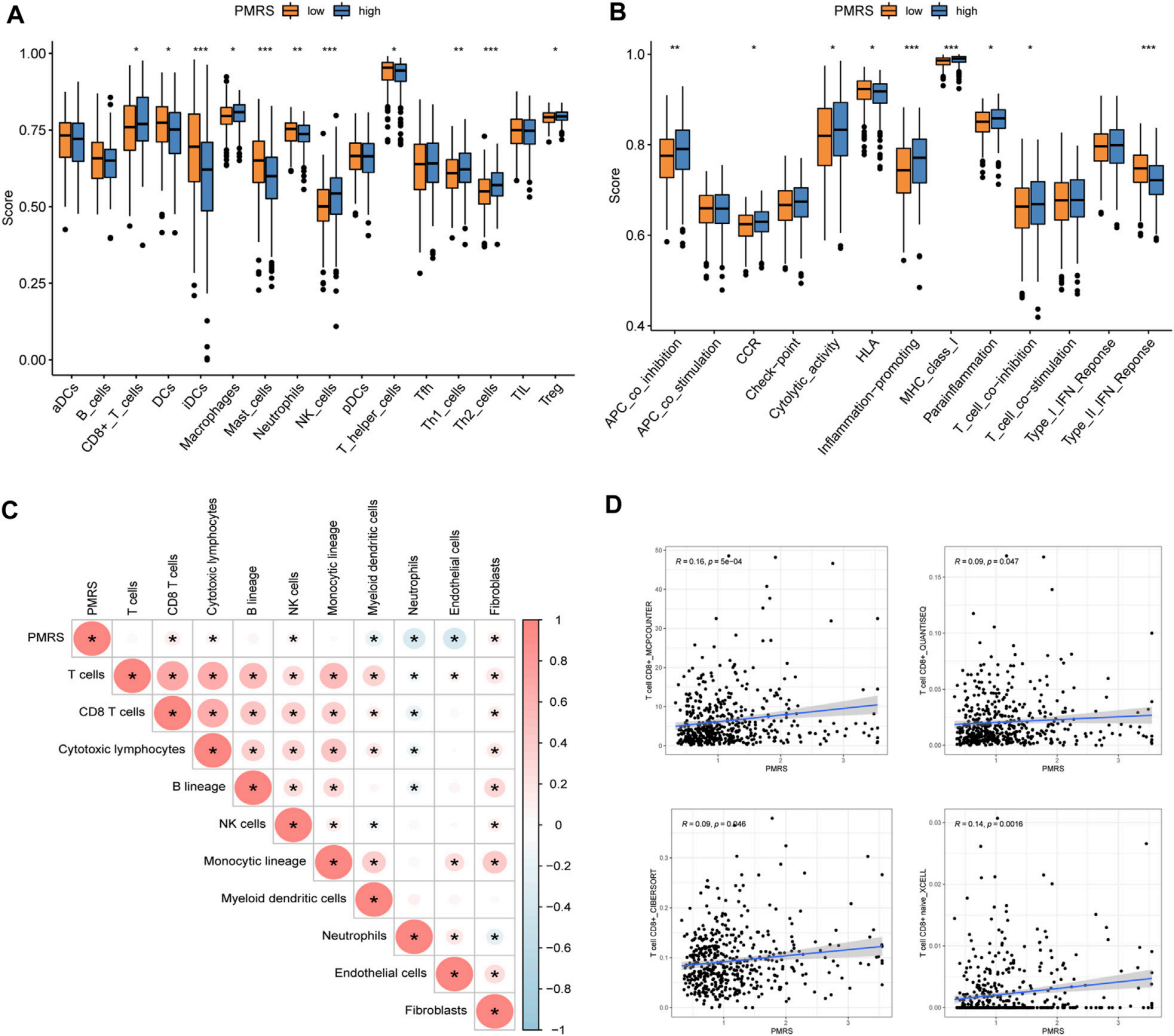
Immune profile in the high- and low-PMRS groups. **(A)** Immune profile and **(B)** immune function pathway analysis by single-sample gene set enrichment analysis (ssGSEA). **(C)** Correlation analysis between the PMRS, immune cells, and stroma cells. **(D)** Correlation analysis between the PMRS and CD8^+^ T cells. *p < 0.05; **p < 0.01; and ***p < 0.001.

In order to characterize the tumor’s immune microenvironment, we applied a series of immune infiltration methods. A total of 16 kinds of immune cell infiltration scores between the two PMRS groups were calculated by the ssGSEA method. Then, we observed that CD8^+^ T cells, macrophages, NK cells, Th1 cells, Th2 cells, and Treg cells were activated in the high-PMRS group, while DCs, iDCs, mast cells, neutrophils, and T helper cells were activated in the low-PMRS group (Figure 9A). The results of immune-related functional pathways showed that more immune pathways were activated in the high-PMRS group, including APC co-inhibition, CCR, cytolytic activity, inflammation promotion, MHC class I, para-inflammation, T cell co-inhibition, only HLA, and IFN type II response, suggesting the high-PMRS group to be the immuno-active phenotype (Figure 9B). The result of the MCP-counter immune infiltration method showed that the PMRS was positively correlated with CD8^+^ T cells, cytotoxic lymphocytes, NK cells, and fibroblasts while negatively correlated with myeloid dendritic cells, neutrophils, and endothelial cells (Figure 9C). Moreover, we also found that the PMRS was positively correlated with CD8^+^ T cell infiltration by using different software (Figure 9D).

**FIGURE 10:**
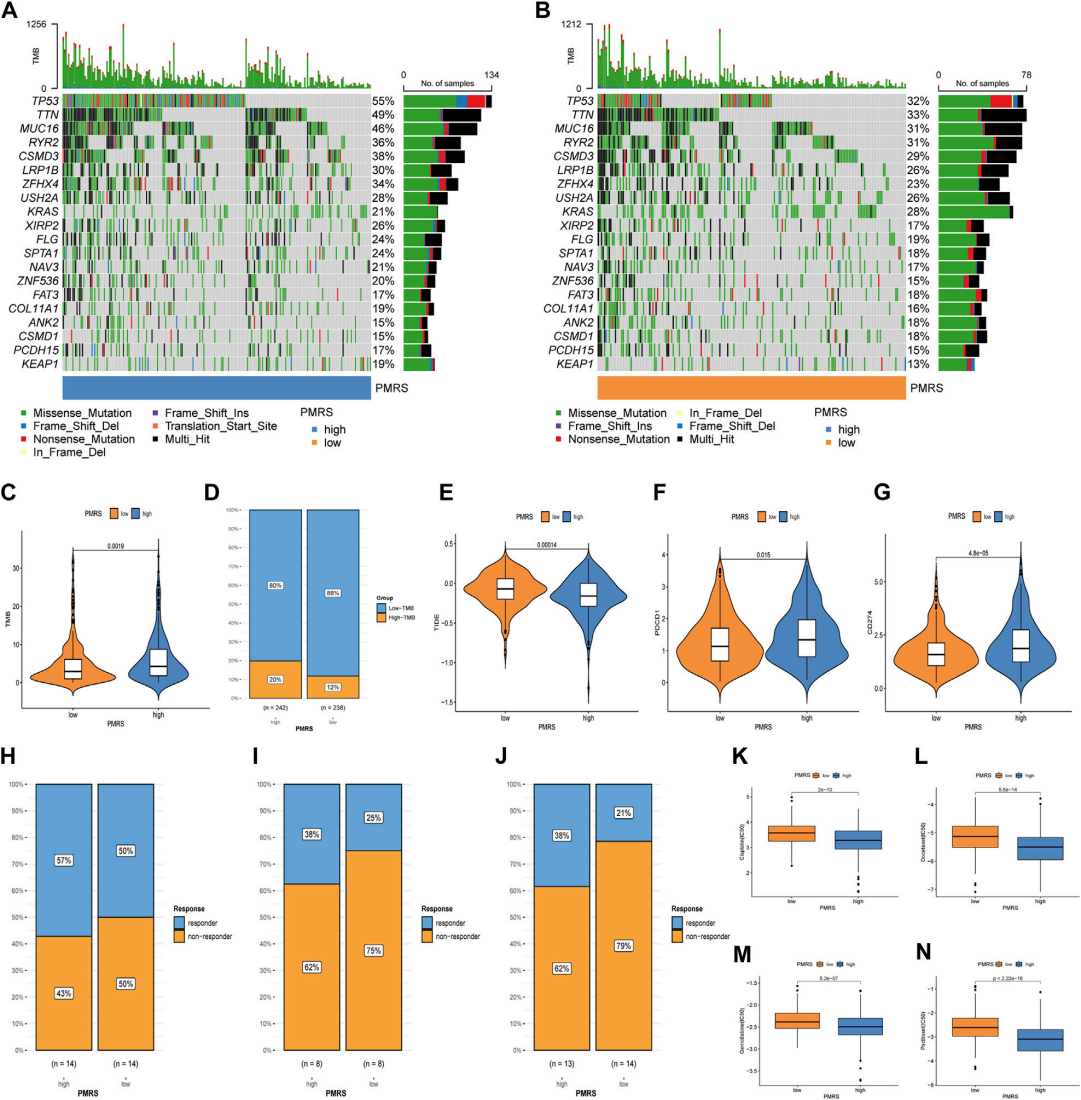
Immunotherapy response and chemotherapeutic drug efficacy evaluation. **(A, B)** Mutation map of the PMRS. **(C, D)** Distribution of the TMB. **(E)** Distribution of TIDE. **(F, G)** Distribution of PDCD1 and CD274. **(H–J)** Distribution of responders between high- and low-PMRS groups in the GSE78220, GSE126044, and GSE135222 immunotherapy cohorts. **(K–N)** Comparison of chemotherapy drug sensitivity between the two groups.

Subsequently, we investigated the application value of predicting the immunotherapy response. The mutation waterfall displays distinct gene mutation frequencies in the two PMRS groups. The high-PMRS group had more gene mutation frequency than the low-PMRS group, and TP53 was the top mutation gene in both groups (55% vs. 32%) (Figures 10A, B). TP53, TTN, MUC16, and RYR2 are the main genes involved in resistance to immunotherapeutic drugs in low-TMB patients (Figure 10B). Compared to the low-PMRS group, the high-PMRS group displayed a higher TMB value (Figure 10C). The high-PMRS group still had a higher proportion of high TMB after the TMB score was split into high- and low-TMB scores (Figure 10D). The PMRS was positively associated with the TMB (R = 0.16, *p* < 0.001; Supplementary Figures S8A, B). We also found that the C1 subtype and geneCluster A were correlated with high-PMRS and -TMB groups, which were consistent with the previous results. After integrating the TMB score and PMRS, the low-TMB and high-PMRS groups had the worst survival outcome, while the high-TMB and low-PMRS groups had the most advantageous survival outcome (Supplementary Figure S8C). Then, the relationship between the PMRS and TIDE score was investigated, and the results revealed that the individuals in the high-PMRS group had a low TIDE score (Figure 10E, Supplementary Figure S8D). In addition, a significant upregulation of PDCD1 and CD274 was found in the high-PMRS group (Figures 10F, G). To demonstrate the strong power of the polyamine metabolism-related score system in evaluating immunotherapy response, three immunotherapy cohorts were applied to compare the distribution of responders in the two PMRS groups. In the GSE78220, GSE126044, and GSE135222 cohorts, the high-PMRS group exhibited a greater number of immunotherapy responders than the low-PMRS group (Figures 10H–J). We further analyzed the sensitivity of common chemotherapy drugs between the two PMRS groups (Figures 10K–N). The results showed that the IC_50_ value of cisplatin, docetaxel, gemcitabine, and paclitaxel was lower in the high-PMRS group than in the low-PMRS group, indicating that the high-PMRS group was sensitive to these drugs. In summary, all the above results could serve as a benchmark for treatment stratification for individuals with LUAD.

## Discussion

Lung cancer is a heterogeneous tumor, including its molecular basis and histology (Vogelstein et al., 2013). LUAD, being the most prevalent form of lung cancer, exhibits not only high invasiveness but also has the highest heterogeneity (Devarakonda et al., 2015). In recent years, the treatment of lung cancer based on traditional histological classifications has improved, but the emergence of drug resistance remains an intractable problem. According to previous reports, the prognosis and therapeutic response of lung cancer are linked to polyamine (Takahashi et al., 2015; Al-Habsi et al., 2022). Therefore, investigating the distinctive molecular classifications of lung cancer, especially those related to polyamine, will help improve the patient’s prognosis and determine a personalized treatment protocol.

First, we observed that the polyamine metabolism-related genes displayed distinct expression levels and highly complicated interaction relationships between lung adenocarcinoma and normal samples, suggesting that polyamine metabolism may play a vital role in lung adenocarcinoma. So, according to the polyamine metabolism-related genes, the lung adenocarcinoma patients were split into two different clusters. Then, the study illustrated the two polyamine metabolism patterns with disparate characteristics. C1 presented a higher expression level of polyamine metabolism-related genes and a poorer survival rate. The previous reports revealed that the increasing level of polyamine is related to a poor prognosis in lung cancer (Takahashi et al., 2015), which is consistent with the results. In addition, the C1 subtype was strongly correlated with advanced-stage tumor (stages III + IV) and lymph node involvement (pathologic stage N). This condition may be attributed to the fact that polyamine could maintain tumor cell continual proliferation and promote tumor lymph node metastasis (López-Contreras et al., 2019; Xu et al., 2020). The TME is a heterogeneous ecosystem containing tumor cells and non-tumor cells. The immune cells are part of the main components of non-tumor cells, especially CD8^+^ T cells, which are the cells leading to damage to the tumor cell (Raskov et al., 2020). However, the CD8^+^ T cells exerting an anti-tumor immune response relied on their normal cellular function (Raskov et al., 2020). The immune checkpoints, including PD-1 and PD-L1, expressed on CD8^+^ T cells and tumor cells, could apparently restrain the T cell immunity (Dammeijer et al., 2020). We observed that the C1 subtype had higher expression levels of PD-1 and PD-L1, suggesting these had defective immunity, which could lead to a poor prognosis. Luckily, PD-1 and PD-L1 were the widely supported biomarkers to guide immunotherapy, and the ICB targeting the two biomarkers has achieved great clinical therapeutic results in NSCLC (Ettinger et al., 2022). Our findings observed that the C1 subtype exhibited PD-1 and PD-L1 overexpression compared to the C2 subtype, suggesting that the C1 subtype could potentially derive advantages from immunotherapy. Additionally, TMB is also a strong biomarker to elevate the patient response to antitumor immunotherapy in various cancer types (Goodman et al., 2017). The result showed that the C1 subtype possessed a higher TMB than the C2 subtype. After the classification of the TMB score into high- and low-TMB groups, the C1 subtype still had a higher proportion of high TMB, indicating that the C1 subtype may present sensitivity to immunotherapy. Finally, we compared the TIDE scores between the two subtypes. The TIDE is a computational method that evaluates the probability of tumor immune evasion in the gene expression profiles of tumor samples, and its results are thought to be an alternative to single biomarkers for effectively predicting the effects of ICB (Jiang et al., 2018). Generally, the TIDE score has an inverse relationship with the effects of ICB. According to the results of the TIDE score comparison between the two subtypes, the effects of ICB may be better in the C1 subtype than in the C2 subtype.

Furthermore, in this study, the mRNA transcriptome differences between different polyamine metabolism patterns were found to be significantly correlated with polyamine metabolism-related gene signatures. Two geneCluster types were established according to the DEGs, which were similar to the clustering results of the polyamine metabolism phenotypes. Parallel to the characteristics of the polyamine metabolism patterns, the geneCluster was remarkably associated with different clinical prognoses, profiles of immune infiltration, and antitumor immunity. This demonstrated again that polyamine metabolism patterns are involved in tumor development, shaping distinct TME landscapes and immunotherapy effects. In summary, we believe that the polyamine metabolism patterns will become a novel biomarker to predict the survival prognosis and immunotherapy response of LUAD patients.

Subsequently, the PMRS system was constructed to characterize the polyamine metabolism patterns. Three genes, namely proteasome 26S subunit, ATPase 6 (PSMC6), spermine oxidase (SMOX), and spermine synthase (SMS), were selected through a series of steps to build the score, which was applied to divide the patients into high- and low-PMRS groups. The high-PMRS group presented upregulation of PSMC6, SMOX, and SMS. PSMC6 is a PSMC family member with an ATPase function for unpacking and relocating the substrates (Bhattacharyya et al., 2014). The PSMC family member contains PSMC1, PSMC2, PSMC3, PSMC4, PSMC5, and PSMC6, which constitute the 19S proteasome complex (Gu and Enenkel, 2014). Jia et al. (2022) revealed the prognostic effect and related immune profile of PSMC genes in LUAD. As reported, PSMC6 showed upregulation in the LUAD sample, which may activate WNT signaling to promote tumor progression. For knockdown, PSMC6 could inhibit cancer cell development, migration, and invasion (Zhang et al., 2021a). In the study, we observed that PSMC6 was overexpressed in the TCGA LUAD sample, and we validated this with other LUAD cohorts and immunohistochemistry detection results (Figures 6A, J). Similarly, the high expression level of PSMC6 was related to adverse survival outcomes. All these results were consistent with previous reports, which could partially explain the worse prognosis of the high-PMRS group. Considering that this protein upregulation could result in the abnormal degradation of the mediators of the cell cycle and apoptosis regulators, targeting the proteasome activity is a potential antitumor method (Park et al., 2018). Then, we found that the high-PMRS group and the tumor sample had a high expression level of SMOX. SMOX is a key enzyme involved in the polyamine metabolism pathway, which takes spermine as a substrate to catalyze the production of spermidine, the aldehyde 3-aminopropanal, and H_2_O_2_ (Cervelli et al., 2013). Previous research has indicated that SMOX overexpression is a significant predisposing factor and a poor prognostic indicator for many malignant tumors and is strongly correlated with the occurrence and development of gastric cancer (McNamara et al., 2021) and prostate cancer (Peng et al., 2021). Sun et al. (2019) discovered a novel, new SMOX inhibitor that possessed anti-tumor activity against lung cancer A549 cells by effectively inhibiting SMOX activity, interfering with polyamine metabolism, and depleting the cellular polyamine content. The expression level result of SMS was similar to that of PSMC6 and SMOX. SMS is a highly specific aminopropyltransferase that catalyzes spermidine to generate spermine (Pegg and Michael, 2010). Currently, SMS is correlated with tumorigenesis in many tumors, including pancreatic cancer (Guo et al., 2022), breast cancer (Hanash et al., 2020), and colorectal cancer (Guo et al., 2020). However, it is unclear what role SMS may play in the occurrence and progression of LUAD. This research implied that SMS presented a higher expression level in the LUAD sample than in the normal sample, which was verified by other LUAD samples and immunohistochemistry tests (Figures 6C, L). In addition, SMS showed upregulation in the high-PMRS group, which had an adverse survival outcome. The mechanisms of SMS contributing to LUAD malignancy require further research in the future. Then, the internal and external samples were utilized to validate the expression levels of the three genes and the contribution of the PMRS to survival prognosis. Similar results were obtained, which demonstrated the good performance of the PMRS for evaluating the prognosis of LUAD patients.

We further investigated the application of the three genes to other clinical factors. First, PSMC6 (AUC = 0.866), SMOX (AUC = 0.844), and SMS (AUC = 0.856) displayed the capacity to differentiate normal samples from LUAD samples, so polyamines may be applied in diagnosing LUAD in the future. Second, the PMRS was positively correlated with lymph node metastasis (pathological stages N2 + N3) and advanced stage (stages III + IV), leading to adverse survival in the high-PMRS group. After dividing the patients into different clinical subgroups, the high-PMRS group still possessed poor survival outcomes, indicating that the score was powerful enough to assess the survival rate of patients with different clinical situations. Subsequent correlation analysis revealed that the high-PMRS group exhibited a connection with the C1 subtype and geneCluster A group, so the adverse survival of the high-PMRS group proved the poor survival of the C1 subtype and geneCluster A group. The functional enrichment analysis disclosed that the cancer-related regulation pathways were mainly activated in the high-PMRS group, including E2F_targets (Kent and Leone, 2019), PI3K_AKT_MTOR (He et al., 2021), and epithelial–mesenchymal transition (EMT) (Dongre and Weinberg, 2019), so these may mechanistically explain the tumor progression and unfavorable prognosis observed in patients belonging to the high-PMRS group. Currently, some mTOR and EMT inhibitors have been developed (Chen and Zhou, 2020; Zhang et al., 2021b), but their combination therapies have not been studied *in vitro* or *in vivo*. Therefore, exploring the efficacy differences of the combined treatment with these two inhibitors in high- and low-PMRS groups holds great prospects. Given that the PMRS is a separate indicator of prognosis, a nomogram combining the PMRS with other clinical factors was established. Subsequent investigations confirmed that the nomogram outperformed other clinical factors when applied in estimating the survival rate, and it is also an independent predictor of prognosis. All the above results demonstrate that the PMRS has great potential for aiding the clinical assessment of LUAD patient prognosis.

Finally, the PMRS prediction of immune cell infiltration and antitumor efficacy was investigated. Similarly, the CD8^+^ T cells and NK cells were more convergent in the high-PMRS group than in the low-PMRS group by ssGSEA analysis, and we found that the PMRS showed a positive correlation with the CD8^+^ T cell infiltration, which was consistent with the C1 subtype and geneCluster A group. The TMB and immune checkpoint (PD-1 and PD-L1) expression levels were high, and the TIDE was low in the high-PMRS group. Additionally, we found that the high-PMRS group presented a high frequency of tumor protein P53 (TP53), titin (TTN), mucin 16 (MUC16), and ryanodine receptor (RYR2) mutations. In LUAD patients, the TP53 mutation is strongly linked to elevated immune checkpoint expression, and patients with a TP53 mutation show a favorable response to ICB (Dong et al., 2017; Schoenfeld et al., 2020). Wang et al. (2021) reported that the TTN mutation is related to a high-immunogenicity and inflammatory tumor immune microenvironment, suggesting that the TTN mutation may be a potential predictive marker for patients with LUAD to accept immunotherapeutic drugs. The MUC16 mutation seemed to correlate with genomic factors linked to response and better outcomes to ICB treatment in solid tumors, so the mutation shows potential as an indicator to guide response to immunotherapy (Zhang et al., 2020). An et al. (2022) indicated that the RYR2 mutation combined with a dendritic cell-related risk score is useful for predicting the prognosis and discovering appropriate patients for immunotherapy. Furthermore, in the lung cancer and melanoma immunotherapy cohorts, the high-PMRS group had a higher percentage of patients, achieving better therapeutic effects than the low-PMRS group. All these findings suggest that the PMRS may be a robust biomarker to estimate the immunotherapeutic response. Cytotoxic chemotherapy is one of the main therapy methods for lung cancer; nevertheless, the emergence of drug resistance remarkably limited the drug efficacy and resulted in a shortened overall survival time (Min and Lee, 2021). We investigated the performance of the PMRS in assessing the effects of common chemotherapy drugs. Compared to the low-PMRS group, the study disclosed that cisplatin, docetaxel, gemcitabine, and paclitaxel are more suitable for the high-PMRS group.

Wang et al. (2023) reported a polyamine metabolism-related signature, but it contained 14 genes and mainly focused on evaluating the survival outcome. In contrast, the PMRS constructed by the research had fewer genes than the previous study, but it was possible to achieve the same effectiveness in evaluating prognostic effects and could characterize immune cell infiltration and evaluate the immunotherapy response and chemotherapeutic drug efficacy. This research explored the polyamine metabolism patterns and established a novel score system, which may have great potential for improving the prognosis of LUAD patients, but there are some limitations. First, functional characterization of polyamine metabolism-related genes should be carried out in *in vitro* and *in vivo* experiments to explore the mechanisms of the impact of polyamine on immune infiltration. Second, clinical trials should be conducted to verify the association between PMRS and the effectiveness of chemotherapy drugs. Finally, the difference in gene mutation frequency between the high-PMRS and low-PMRS groups is based on methodological prediction, so *in vitro* and *in vivo* experiments that bolster the findings of our research need to be implemented in the future.

## Conclusion

In conclusion, two distinct polyamine metabolism patterns were identified, and the distribution of survival outcomes, immune infiltration, and immunotherapy response was significantly different between the two subtypes, suggesting that the polyamine modification patterns had a great impact on the prognosis of patients and TME. Subsequently, we constructed a polyamine metabolism-related score system and applied it to evaluate the prognosis, immune profile, and effectiveness of treatment in LUAD patients. Additionally, the scoring system could characterize the individual tumor polyamine alteration and help guide immunotherapy and chemotherapy. These discoveries will offer new perspectives on the mechanism of polyamine and improve the current antitumor strategies in LUAD.

## Data Availability

The datasets presented in this study can be found in online repositories. The names of the repository/repositories and accession number(s) can be found in the article/Supplementary Material.

## Supplementary material

SUPPLEMENTARY TABLE S1 The polyamine metabolism-related gene.

SUPPLEMENTARY TABLE S2 The antibody information about each gene was applied for immunohistochemistry.

SUPPLEMENTARY TABLE S3 The DEGs between two subtypes.

SUPPLEMENTARY TABLE S4 The prognosis-related genes using univariate Cox regression analysis.

SUPPLEMENTARY TABLE S5 Seven polyamine metabolism-related genes using LASSO regression analysis.

SUPPLEMENTARY TABLE S6 The coefficients of three key prognostic genes.

SUPPLEMENTARY FIGURE S1
**(A)** The distribution of TMB. **(B)** The distribution of TIDE.

SUPPLEMENTARY FIGURE S2
**(A)** The distribution of TMB. **(B)** The distribution of TIDE.

SUPPLEMENTARY FIGURE S3
**(A-C)** The segmentation of high- and low-PMRS groups based on the median of the PMRS.

SUPPLEMENTARY FIGURE S4
**(A-C)** The segmentation of high- and low-PMRS groups in the TCGA cohort. The same analysis in the TCGA test cohort **(D–F)** and the GSE13213 and GSE50081 LUAD cohorts **(G–I)**.

SUPPLEMENTARY FIGURE S5 The comparison of genes between tumor and normal samples in the GSE46539 lung adenocarcinoma cohort.

SUPPLEMENTARY FIGURE S6 Correlation between clinical features and the PMRS.

SUPPLEMENTARY FIGURE S7
**(A)** AUC of the 14-gene signature (Wang) for predicting 1-, 3-, and 5-year survival rates. **(B)** C-index of each signature.

SUPPLEMENTARY FIGURE S8 The correlation analysis between PMRS, TMB, clusters **(A)**, and geneClusters **(B)**. **(C)** The survival analysis stratified by the TMB and PMRS. **(D)** The distribution of TIDE.

## Abbreviations

LUAD, lung adenocarcinoma; TCGA, The Cancer Genome Atlas; DEGs, differentially expressed genes; PMRS, polyamine metabolism-related score; TMB, tumor mutational burden; TIDE, tumor immune dysfunction and exclusion; GEO, Gene Expression Omnibus; CNV, copy number variation; ssGSEA, single-sample gene set enrichment analysis; ICB, immune checkpoint blockade; LASSO, least absolute shrinkage and selection operator; GSVA, gene set variation analysis; AUC, area under the curve; NSCLC, non-small-cell lung cancer; IC_50_, half-maximal inhibitory concentration; PCA, principal component analysis.
