# Supplementary figures and images for: Characterization of polyamine metabolism predicts prognosis, immune profile, and therapeutic efficacy in lung adenocarcinoma patients
Source: Front Cell Dev Biol. 2024 Apr 8;12:1331759. doi: 10.3389/fcell.2024.1331759 (PMC11033315; doi:10.3389/fcell.2024.1331759)

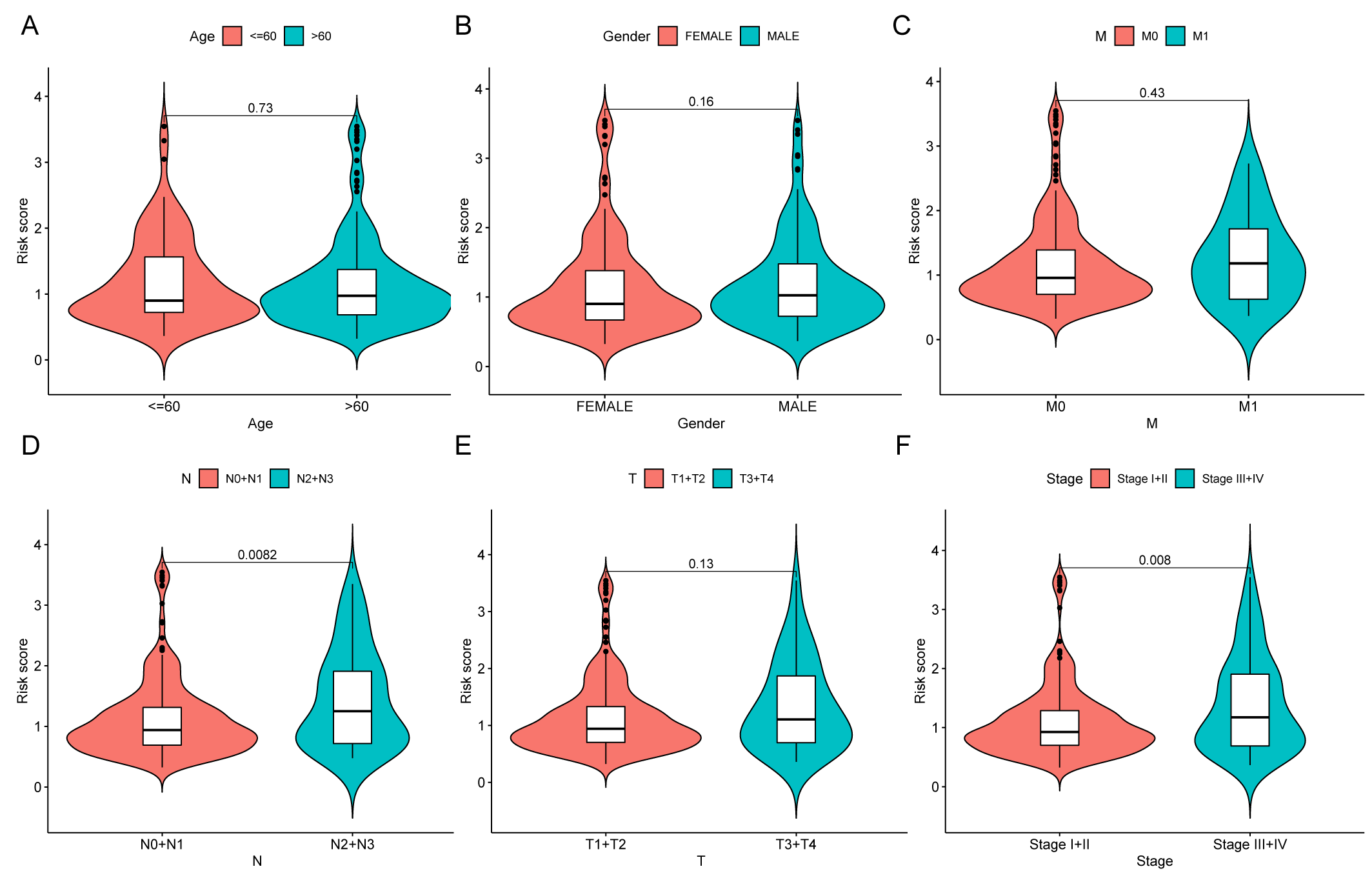

Supplement: Supplementary file 1 [file Image6.TIF]

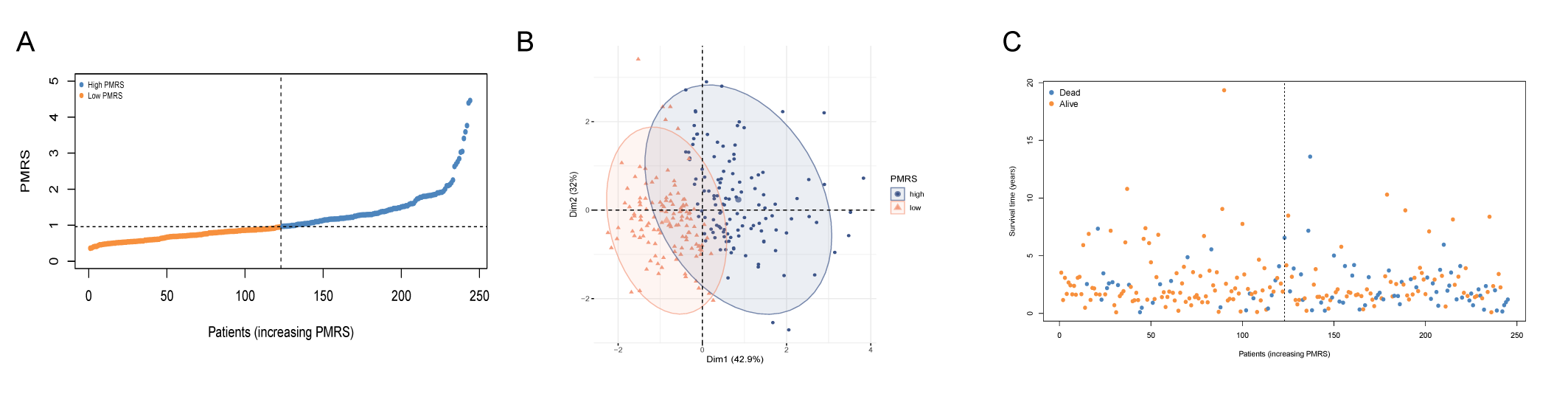

Supplement: Supplementary file 3 [file Image3.TIF]

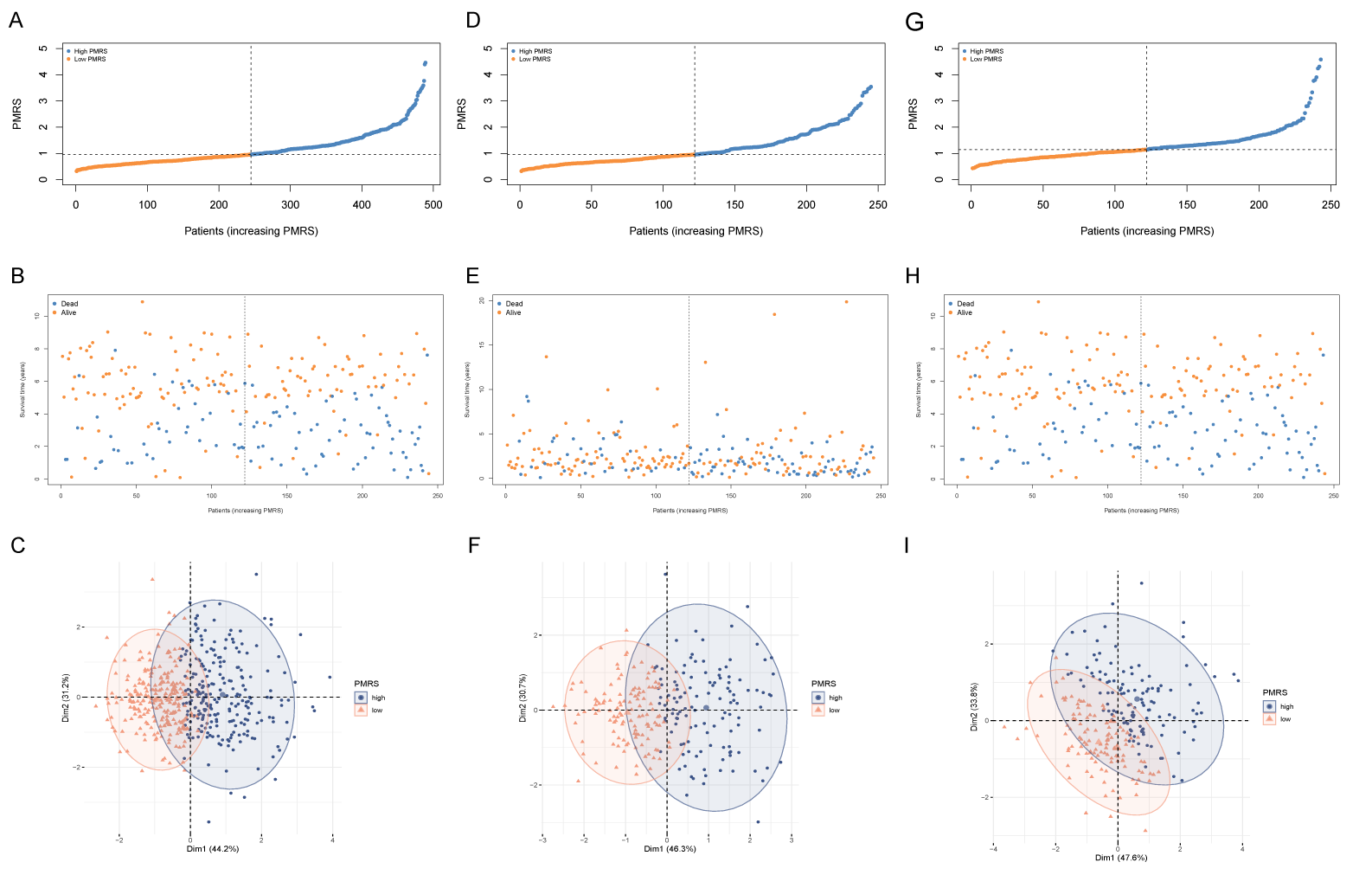

Supplement: Supplementary file 4 [file Image4.TIF]

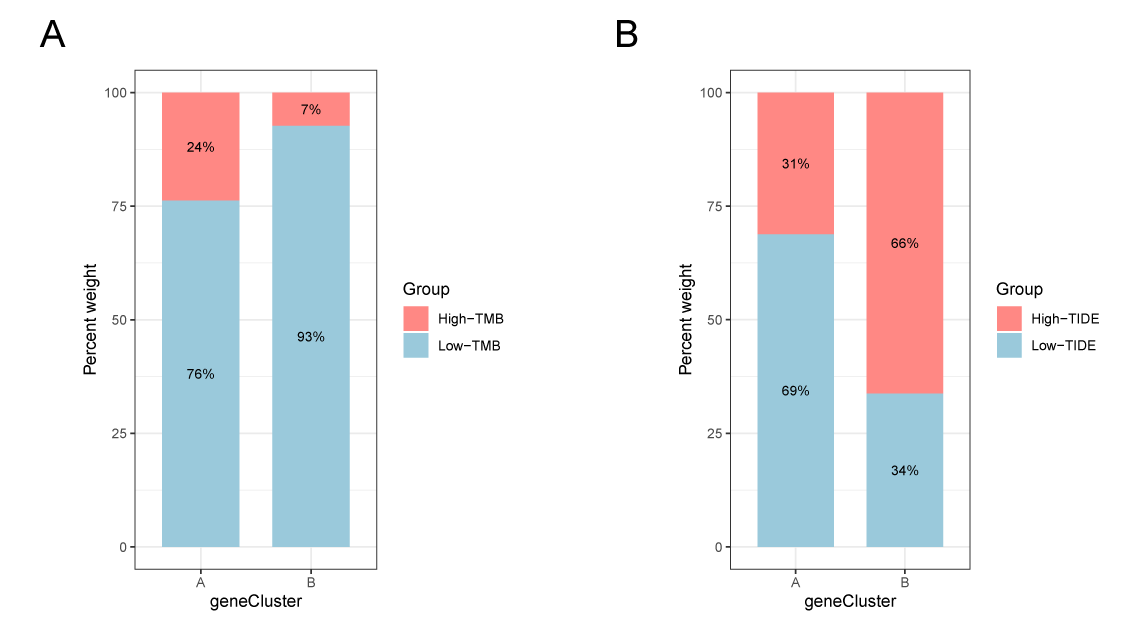

Supplement: Supplementary file 5 [file Image2.TIF]

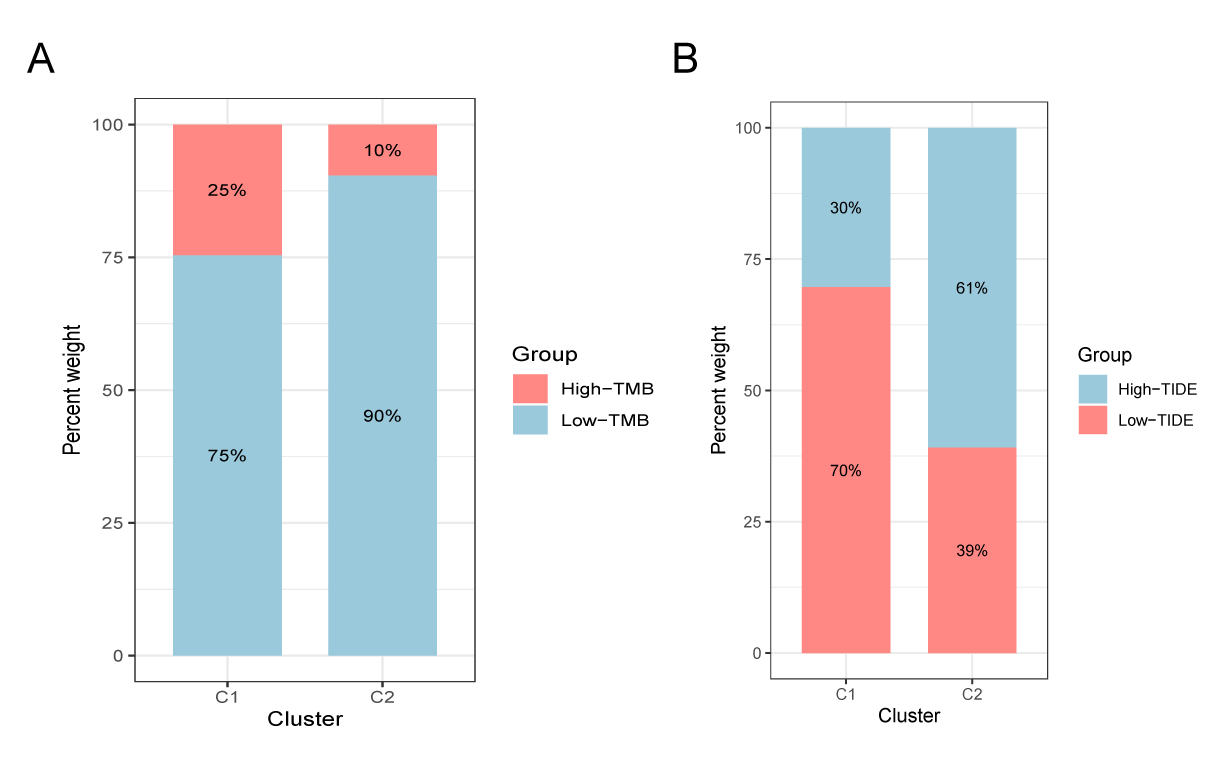

Supplement: Supplementary file 6 [file Image1.TIF]

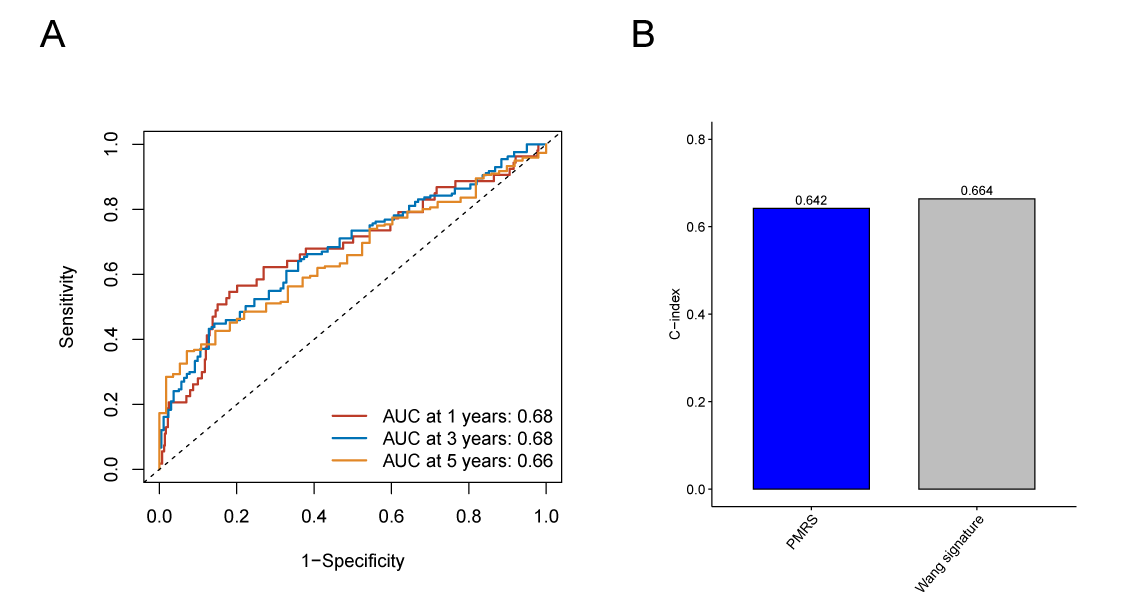

Supplement: Supplementary file 7 [file Image7.TIF]

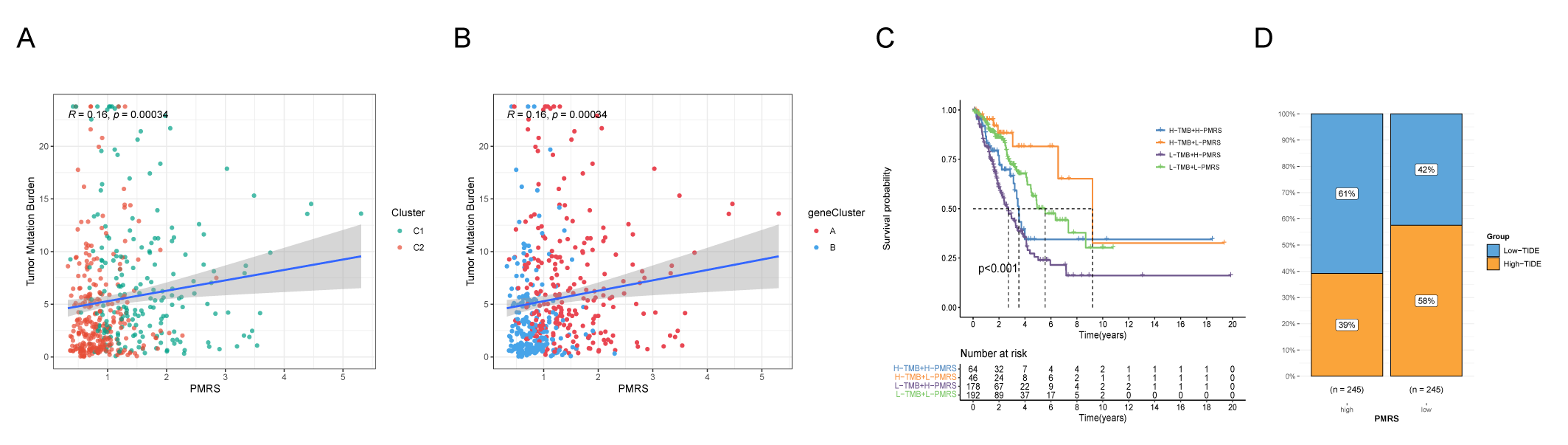

Supplement: Supplementary file 12 [file Image8.TIF]

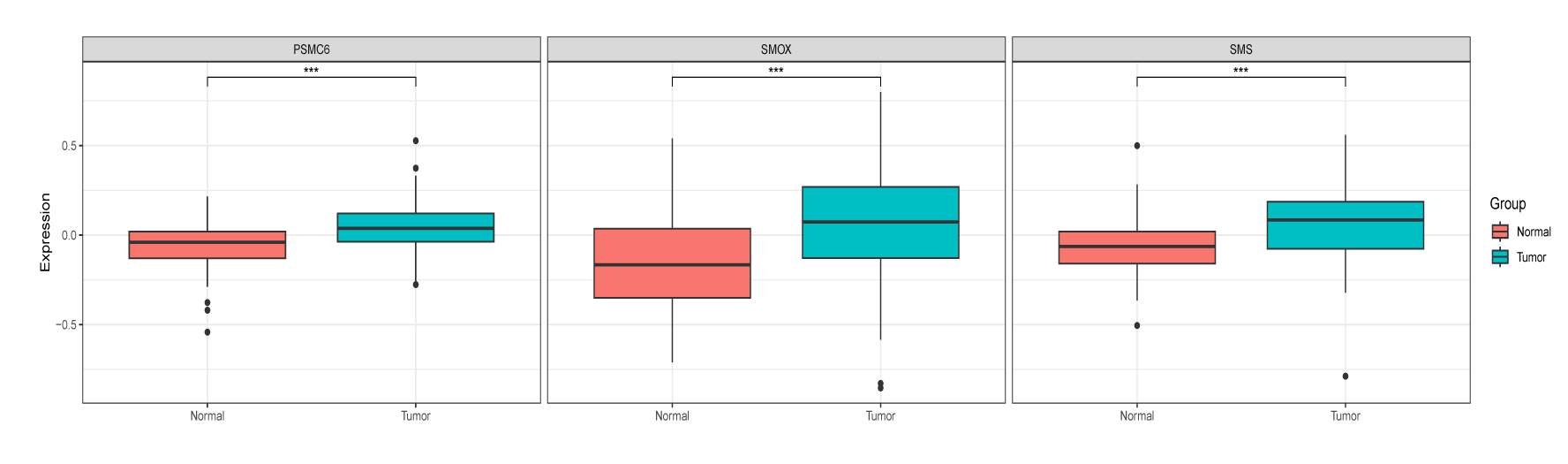

Supplement: Supplementary file 14 [file Image5.TIF]
