# Supplementary material for: Characterization of polyamine metabolism predicts prognosis, immune profile, and therapeutic efficacy in lung adenocarcinoma patients
Source: Front Cell Dev Biol. 2024 Apr 8;12:1331759. doi: 10.3389/fcell.2024.1331759 (PMC11033315; doi:10.3389/fcell.2024.1331759)
Supplement: Supplementary file 2 [file Table1.DOCX]

REACTOME_METABOLISM_OF_POLYAMINES

https://www.gsea-msigdb.org/gsea/msigdb/human/geneset/REACTOME_METABOLISM_OF_POLYAMINES

AGMAT

AMD1

AZIN1

AZIN2

NQO1

OAZ1

OAZ2

OAZ3

ODC1

PAOX

PSMA1

PSMA2

PSMA3

PSMA4

PSMA5

PSMA6

PSMA7

PSMA8

PSMB1

PSMB10

PSMB11

PSMB2

PSMB3

PSMB4

PSMB5

PSMB6

PSMB7

PSMB8

PSMB9

PSMC1

PSMC2

PSMC3

PSMC4

PSMC5

PSMC6

PSMD1

PSMD10

PSMD11

PSMD12

PSMD13

PSMD14

PSMD2

PSMD3

PSMD4

PSMD5

PSMD6

PSMD7

PSMD8

PSMD9

PSME1

PSME2

PSME3

PSME4

PSMF1

SAT1

SEM1

SMOX

SMS

SRM
