# Supplementary material for: Characterization of polyamine metabolism predicts prognosis, immune profile, and therapeutic efficacy in lung adenocarcinoma patients
Source: Front Cell Dev Biol. 2024 Apr 8;12:1331759. doi: 10.3389/fcell.2024.1331759 (PMC11033315; doi:10.3389/fcell.2024.1331759)
Supplement: Supplementary file 8 [file Table2.DOCX]

| **Target protein** | **Vendor catalogue ID** | **Tissue** | **Staining** | **Intensity** | **Quantity** | **Location** |
| --- | --- | --- | --- | --- | --- | --- |
| PSMC6 | HPA042823 | Normal | Medium | Moderate | 75%-25% | Cytoplasmic/ membranous nuclear |
| PSMC6 | HPA042823 | Tumor | Medium | Moderate | >75% | Nuclear |
| SMOX | HPA047117 | Normal | Medium | Moderate | 75%-25% | Cytoplasmic/ membranous nuclear |
| SMOX | HPA047117 | Tumor | High | Strong | >75% | Cytoplasmic/ membranous nuclear |
| SMS | HPA029852 | Normal | Not detected | Negative | None | / |
| SMS | HPA029852 | Tumor | Medium | Moderate | >75% | Cytoplasmic/ membranous |
