# Supplementary material for: Characterization of polyamine metabolism predicts prognosis, immune profile, and therapeutic efficacy in lung adenocarcinoma patients
Source: Front Cell Dev Biol. 2024 Apr 8;12:1331759. doi: 10.3389/fcell.2024.1331759 (PMC11033315; doi:10.3389/fcell.2024.1331759)
Supplement: Supplementary file 9 [file Table5.DOCX]

id futime fustat PAOX PSMB7 PSMC1 PSMC6 PSMD2 SMOX SMS

TCGA-49-4510 2.45479452054795 1 4.70816107084232 4.34052653395919 4.32272118873298 4.98030847358445 4.52825334021209 4.8719829923562 4.05284946030268

TCGA-69-7763 1.89041095890411 0 4.83664432021799 4.86993515108222 5.05874335544962 5.19605453656112 4.95309114253302 5.42293112332058 4.49199786232718

TCGA-95-7039 3.48493150684931 0 5.64710093067358 4.68021316778459 5.07915546440747 4.95818428218915 4.52492793712345 4.34052653395919 5.36481021747551

TCGA-95-A4VK 1.78356164383562 0 5.25733086237873 5.35406029799732 4.69379767353218 4.82670556068671 4.20387133212243 5.01501081057102 4.6869664694162

TCGA-49-AARO 10.2986301369863 0 5.15661664469614 4.52492793712345 4.95818428218915 5.23053317285912 4.80241956207367 5.45074474082551 5.38765365796728

TCGA-55-7994 1.65205479452055 0 4.75930034919142 4.28174789628393 5.50677723493176 4.73272544830853 5.82446290729509 4.17303206018344 4.598344329566

TCGA-78-7149 10.7945205479452 0 4.90869441992528 4.6869664694162 3.97178863070305 4.92791250435688 4.28174789628393 4.85765100664681 4.24303164168801

TCGA-53-7626 2.54520547945205 1 5.94544404570681 5.08717531991403 5.50337913754276 4.70816107084232 5.08161845529761 4.60360062598397 4.36899298148057

TCGA-78-7539 2.16712328767123 0 6.02969354559291 5.50337913754276 4.71622628584955 4.6249569887363 4.41588575730423 4.46421492033324 4.72190294546832

TCGA-83-5908 2.25753424657534 0 4.97116122088623 5.9670969075463 6.27768789026263 6.17826740809128 6.08894364647097 5.87667250987434 6.59109505615328

TCGA-69-8254 1.12054794520548 0 5.52696972064213 5.14623884118183 5.39469067466805 4.83304009008021 4.98611100920672 5.22490800517098 4.89594974385834

TCGA-44-A47G 0.961643835616438 0 4.98611100920672 4.69988820108664 4.65669749616631 4.76227887369914 5.39469067466805 4.7136245551658 5.45074474082551

TCGA-44-2668 2.08493150684932 1 4.59509417438844 5.63738960708029 6.24640002569411 6.12904916587147 6.63864479412907 5.75748655020916 5.30129551200087

TCGA-55-5899 2.54794520547945 0 4.54993794022504 4.51222152885824 6.40432570602522 5.81276325671543 5.32313419531973 5.54660541066437 4.66300647702884

TCGA-J2-A4AD 1.50684931506849 1 5.09499802417961 4.62195132384969 4.90373698575988 5.72044308787751 5.25337206309265 4.82202165175364 5.63356955900206

TCGA-69-7980 1.12602739726027 0 4.72619071303912 5.35017070969008 5.58838427993837 4.6651087637667 5.60412104568954 5.23053317285912 5.24794870620138

TCGA-05-4417 1.24657534246575 0 4.94322806346756 5.1070429609878 5.16341170598797 4.38053241058664 4.54532091053601 5.17998681670756 4.92455312058737

TCGA-44-6146 1.99452054794521 0 4.88725008385658 4.4096376642013 4.10546304343291 4.19442088577543 4.40378758807867 5.85265234899436 5.0760656222892

TCGA-67-3770 1.67123287671233 0 4.37504842723099 4.74736569442777 5.5348321339118 6.13641661524228 4.68363218591554 4.29941848627659 4.58632574928033

TCGA-MP-A4T4 7.16986301369863 1 5.30129551200087 5.18693725193171 4.78071114469456 4.93557461567931 5.46251678286192 5.16855014735371 5.42816930855285

TCGA-NJ-A4YF 5.92054794520548 0 4.067075734095 5.07915546440747 4.40378758807867 4.39336202373133 5.81276325671543 4.56501479720614 4.72619071303912

TCGA-50-5055 5.01369863013699 1 6.06381540714332 5.53034917555286 4.97663580279272 5.68074054932273 4.86707704259779 5.14850696063783 4.854651044916

TCGA-44-6776 7.16712328767123 0 4.96152809342821 5.45074474082551 5.65563662634017 4.92455312058737 4.46421492033324 4.39336202373133 4.60360062598397

TCGA-62-8399 7.38630136986301 0 5.56315286868292 6.09640430055241 4.85765100664681 4.7368855347186 4.79325688415672 4.72913220217783 4.8641033809139

TCGA-64-5775 0.16986301369863 1 3.91694385317839 5.37464850389324 5.67540425515099 5.43149212334626 6.43065932304858 5.68074054932273 7.82085842471194

TCGA-64-1677 1.72054794520548 1 5.12186035974213 6.59109505615328 4.77731504594479 6.5541492986356 5.39163201969549 5.20186164859352 5.21033319941472

TCGA-NJ-A4YP 0.136986301369863 0 4.3153294999997 5.11713015001565 4.08919262478158 4.61522851216861 4.677739616492 5.25093854069401 5.07359404760905

TCGA-69-7760 0.553424657534247 0 5.1601514454942 5.28562966294167 5.41166427036383 4.12188024296367 6.07299917011788 5.08161845529761 5.40686320881734

TCGA-64-5778 3.57534246575342 0 4.8521561569892 6.92499467603188 4.56501479720614 5.09499802417961 5.09204281631416 3.99576122753024 5.7528907826479

TCGA-55-8620 1.02739726027397 1 5.45948587463351 6.11913736922898 5.05500763581931 4.64439257268276 5.51672658547113 5.15661664469614 6.08894364647097

TCGA-53-7813 1.16164383561644 0 4.85765100664681 5.47761875985422 5.15457487587607 5.70549590416465 4.63179647925294 4.49199786232718 5.20186164859352

TCGA-55-6982 2.72602739726027 1 5.19824982713734 5.27302778977192 4.49667962467061 5.48385238471085 4.94576628955782 4.8399123619932 5.9670969075463

TCGA-49-AAR9 0.712328767123288 1 4.24303164168801 6.45497186177606 5.3806123981936 6.00081278626219 6.5541492986356 6.35474846268005 6.7917544053942

TCGA-05-4424 2.5013698630137 0 4.88429666028332 4.64439257268276 6.00081278626219 5.36481021747551 5.87667250987434 4.78071114469456 6.1878687357199

TCGA-55-A48Z 1.78356164383562 0 5.74044341673256 5.36772483343786 5.28944198619386 6.00945265646471 4.30579401671983 5.74664690454395 5.1151919660021

TCGA-MN-A4N4 3.21917808219178 0 7.23601681628151 3.75004255696854 4.41588575730423 4.98891258099903 4.96653293861653 5.98599552121818 5.03895492483204

TCGA-05-4405 1.67123287671233 0 5.39921282158178 4.78894039050948 4.52088250841532 4.18538808950802 5.24124956397002 4.88725008385658 4.74992100693531

TCGA-75-7027 8.38082191780822 0 4.96407345132971 4.94322806346756 5.00091037668946 5.07043908254873 4.79522764114779 6.69623099107954 6.12904916587147

TCGA-49-6761 0.96986301369863 0 5.6598948198276 5.11977511833695 4.53806324755926 4.69000480261886 5.40686320881734 5.00886733426488 5.15661664469614

TCGA-55-8619 1.13972602739726 0 5.62868387907542 4.80241956207367 4.21695209102212 4.3153294999997 4.76882838343843 5.01934792071741 4.83664432021799

TCGA-50-5939 1.26027397260274 1 4.49199786232718 5.97462084300779 5.46661360744635 4.81122064061802 5.97462084300779 5.94544404570681 5.7725028549258

TCGA-55-7726 1.78630136986301 0 4.76227887369914 6.10652971501267 5.90057358126119 5.16855014735371 5.61612912359804 5.69102369655262 4.90869441992528

TCGA-86-A4P8 2.20547945205479 0 6.00945265646471 4.067075734095 4.36212613551082 4.62195132384969 4.22582309670354 4.46021633795082 4.71827468816372

TCGA-50-5045 5.95616438356164 1 6.13641661524228 5.65563662634017 4.7368855347186 6.38298797716996 4.78622502216663 5.5562466763786 4.71128869450524

TCGA-97-A4M7 1.72328767123288 0 5.72044308787751 4.28823479686995 5.15124385212914 5.40686320881734 5.13409318935653 5.30129551200087 4.22582309670354

TCGA-50-5066 3.95068493150685 0 4.80845387453467 5.86026539501079 6.21932743488592 6.16350013336462 5.33422367054468 5.04401576942903 5.11713015001565

TCGA-L4-A4E5 1.58356164383562 0 5.25093854069401 5.62490569127957 4.42842197890458 5.2215362497871 5.56315286868292 4.84387806789306 4.65120580746482

TCGA-86-8671 2.2986301369863 0 6.52675813391858 4.74992100693531 4.64439257268276 4.68021316778459 4.6074822239846 4.95585928052126 4.46421492033324

TCGA-62-A472 2.49315068493151 0 4.82670556068671 5.29898921272872 5.57593821979501 5.8832445358893 5.29580550575862 4.38648567886514 5.28562966294167

TCGA-44-7659 1.89315068493151 0 4.9694432354 4.96653293861653 4.19442088577543 6.35474846268005 5.12705768485864 4.3307752985421 4.53462364457964

TCGA-55-A48Y 1.72602739726027 0 4.89276658901782 4.76227887369914 4.90869441992528 4.8641033809139 5.23315036180009 5.63738960708029 5.49376891158473

TCGA-86-8054 3.14520547945205 0 4.18538808950802 5.74664690454395 4.89276658901782 5.03700330074137 4.85765100664681 4.84679765133552 4.56501479720614

TCGA-38-6178 1.22739726027397 0 5.17803699204651 6.67038688371618 6.34327930434214 5.13725668467939 5.62490569127957 4.95309114253302 4.88429666028332

TCGA-78-8640 19.3479452054795 0 5.20418528097581 5.20186164859352 4.84679765133552 4.84679765133552 5.57593821979501 5.62490569127957 4.40378758807867

TCGA-55-A48X 1.88767123287671 0 5.05307049743809 5.43149212334626 5.24794870620138 5.06344998060719 5.5217140633467 4.99341017564513 4.42842197890458

TCGA-55-8614 1.46849315068493 0 5.12396900081901 4.9954899727389 6.04491601986667 5.7136548452647 6.24640002569411 5.60412104568954 4.68363218591554

TCGA-64-1678 3.25753424657534 0 4.48813492886391 6.3295656418649 5.99338881275725 6.92499467603188 3.75004255696854 4.65669749616631 5.05500763581931

TCGA-44-7671 2.43561643835616 0 4.49667962467061 5.44313129089287 4.72913220217783 3.75004255696854 5.45074474082551 5.01698137111624 4.61522851216861

TCGA-44-2665 3.56438356164384 0 4.50868799266451 5.11230705609135 5.09204281631416 5.07915546440747 4.46930389429201 5.11977511833695 4.65946063606462

TCGA-55-8097 1.3041095890411 0 6.24640002569411 4.75329651494594 4.52492793712345 4.54993794022504 4.67494772988294 5.12705768485864 5.01204991644541

TCGA-97-A4M6 1.55616438356164 0 5.41166427036383 4.50115175236105 5.36481021747551 5.57593821979501 4.3153294999997 5.59673720348444 5.00599953960783

TCGA-86-8358 1.78904109589041 0 4.5697629915264 5.05874335544962 6.36540629336816 4.8521561569892 5.49376891158473 4.77434755796381 5.00886733426488

TCGA-50-5936 0.704109589041096 1 5.11977511833695 5.45948587463351 5.88907567756982 5.12911876825784 5.7136548452647 6.13641661524228 5.7797042285245

TCGA-78-7167 7.34520547945205 1 4.84387806789306 4.60360062598397 5.01934792071741 5.44313129089287 4.53806324755926 4.55524310280527 3.68589193779175

TCGA-75-5125 5.55342465753425 1 4.64761300444413 5.74044341673256 5.38765365796728 4.98611100920672 6.02063975954864 4.53462364457964 5.43447837493162

TCGA-62-8402 4.1041095890411 1 5.42293112332058 5.24124956397002 7.82085842471194 5.33020134034042 4.83664432021799 6.46861263233495 5.02298388111108

TCGA-71-6725 0.701369863013699 0 5.54660541066437 4.64040010392167 4.55780563820776 5.30129551200087 5.06587349893486 4.01291492082692 5.4470097069513

TCGA-97-A4M0 1.78630136986301 0 5.65563662634017 4.9694432354 4.48813492886391 4.79325688415672 4.86993515108222 4.73272544830853 4.64040010392167

TCGA-44-A4SU 1.12054794520548 1 5.18693725193171 4.32272118873298 5.26933752300249 5.68686907176196 4.79922658685188 5.41166427036383 5.95550151204503

TCGA-78-7145 2.26301369863014 1 4.22582309670354 4.54532091053601 5.51092920157204 5.766502096287 5.72044308787751 4.98327017057208 5.64710093067358

TCGA-95-8494 0.23013698630137 0 4.42237712992835 5.25983070532304 5.11713015001565 4.23419056213206 5.68686907176196 5.0760656222892 6.34327930434214

TCGA-50-8459 3.06575342465753 0 5.39469067466805 4.68363218591554 4.61151990003138 4.17303206018344 4.65669749616631 6.06381540714332 4.95585928052126

TCGA-86-7953 2.73150684931507 0 4.78894039050948 6.12904916587147 5.28021882690186 5.6598948198276 6.67038688371618 5.00091037668946 5.41914273046275

TCGA-78-7148 1.71506849315068 1 4.92455312058737 5.30915450537318 5.13926724175136 5.1601514454942 4.74044841881869 4.98891258099903 4.90373698575988

TCGA-44-3917 3.24109589041096 0 4.3558475242814 4.03598711655745 5.15661664469614 6.82600730414282 4.13498905520495 3.91694385317839 3.94850930038125

TCGA-55-A4DF 1.20547945205479 1 5.13926724175136 4.57261778019952 5.75748655020916 6.43065932304858 6.61888746744597 5.99338881275725 7.23601681628151

TCGA-50-5068 4.10684931506849 1 5.57593821979501 5.34296277284586 5.27766157790134 6.3295656418649 4.01291492082692 4.24303164168801 5.24124956397002

TCGA-49-4514 4.65753424657534 0 6.16350013336462 5.58263716200823 3.82773477327855 4.58974475128415 4.69379767353218 5.36232993445074 5.17803699204651

TCGA-55-8208 1.84657534246575 0 5.9670969075463 5.25093854069401 5.53896169362172 5.11713015001565 5.7725028549258 5.58838427993837 5.08434729206195

TCGA-55-6543 1.19178082191781 0 5.50337913754276 4.92455312058737 5.7528907826479 5.19020200046397 5.13725668467939 6.17826740809128 4.48813492886391

TCGA-49-4501 3.89315068493151 1 4.91165818347331 5.01204991644541 5.40686320881734 5.42293112332058 5.16590948244236 4.52088250841532 5.48385238471085

TCGA-49-AAR4 2.40821917808219 1 5.43447837493162 4.75638248910094 5.21252572577479 5.55170255595065 5.16855014735371 5.50337913754276 5.82446290729509

TCGA-55-6642 6.70958904109589 0 4.98030847358445 5.00599953960783 5.09900251472474 4.96407345132971 4.43449486931811 5.3806123981936 4.4096376642013

TCGA-05-5423 0.413698630136986 0 4.79522764114779 5.305845166598 5.44313129089287 6.10652971501267 4.61151990003138 4.19442088577543 5.39469067466805

TCGA-05-4432 2.08493150684932 0 6.45497186177606 4.84387806789306 5.55170255595065 4.94576628955782 5.50677723493176 5.21033319941472 5.66886233643835

TCGA-05-4398 3.92054794520548 0 4.36899298148057 5.79881862404872 4.86707704259779 5.87667250987434 5.27766157790134 5.79881862404872 5.21845791601729

TCGA-44-7662 0.597260273972603 0 4.68021316778459 5.90057358126119 5.17803699204651 5.17803699204651 5.50337913754276 5.9670969075463 5.6598948198276

TCGA-55-A490 0.271232876712329 1 4.32272118873298 5.47183251949017 6.82600730414282 4.63179647925294 5.32003253913737 5.08717531991403 5.91644891986612

TCGA-97-8175 1.50958904109589 0 4.51610407080754 5.46661360744635 5.4379337879895 5.5348321339118 5.19293679099683 5.82446290729509 5.79881862404872

TCGA-55-8507 1.14520547945205 0 6.7917544053942 5.01934792071741 5.59673720348444 5.19293679099683 4.74736569442777 5.51092920157204 4.95818428218915

TCGA-38-4629 2.36712328767123 1 4.677739616492 5.78809474521384 5.91644891986612 5.37818449048402 5.80786071299967 5.29282914439146 6.92499467603188

TCGA-97-A4M3 1.47945205479452 0 5.31418739394226 4.98891258099903 4.27104693944814 4.8189648954976 3.87935730457942 5.42816930855285 4.5414927281635

TCGA-55-8205 1.64109589041096 0 5.7005554922005 5.51672658547113 5.66509577421623 5.17264592701774 5.8175071996365 5.04969156106796 6.20350903070615

TCGA-55-7727 0.326027397260274 0 4.53462364457964 5.87125254816225 5.34048776516519 5.845561356816 5.35789177242723 3.68589193779175 5.72044308787751

TCGA-05-4427 2.16712328767123 0 4.28174789628393 5.83804452921552 5.10227338093085 4.66300647702884 5.7005554922005 4.74992100693531 5.70549590416465

TCGA-05-4397 2.0027397260274 1 4.96653293861653 6.69623099107954 6.75848760900896 6.06381540714332 7.82085842471194 5.4470097069513 6.75848760900896

TCGA-44-8117 1.05479452054795 0 5.23887541246387 4.84885337036964 5.74044341673256 5.37464850389324 4.8995725669227 4.34752525368611 4.38053241058664

TCGA-49-6742 1.33698630136986 1 4.58246995167545 6.07299917011788 5.5562466763786 5.38765365796728 5.2215362497871 5.01204991644541 5.67540425515099

TCGA-86-8280 1.92054794520548 0 5.46661360744635 4.72913220217783 5.51672658547113 5.00285032849301 4.90869441992528 4.82439249979648 5.18693725193171

TCGA-86-7955 2.93698630136986 0 5.00091037668946 5.00886733426488 4.78622502216663 4.677739616492 5.83804452921552 5.65563662634017 6.16350013336462

TCGA-L9-A8F4 1.3041095890411 0 5.8175071996365 5.14194365676337 5.69587355706738 5.64710093067358 6.26210027520436 5.15124385212914 5.23887541246387

TCGA-55-8511 1.51232876712329 0 6.82600730414282 5.42816930855285 5.34747421070946 5.91644891986612 5.14850696063783 5.25983070532304 5.86026539501079

TCGA-55-A494 1.31780821917808 0 6.75848760900896 3.45300333262003 4.22582309670354 5.01204991644541 4.34752525368611 5.53896169362172 5.0252100478494

TCGA-44-3919 2.81095890410959 1 5.13409318935653 5.17998681670756 5.36772483343786 5.04169611804675 5.41914273046275 4.61850584999048 4.90185151980137

TCGA-55-8514 1.42465753424658 0 5.07359404760905 4.21695209102212 4.12188024296367 3.82773477327855 4.39866922169541 4.28174789628393 6.69623099107954

TCGA-91-6849 0.0958904109589041 0 5.17998681670756 5.22490800517098 4.88922252922638 4.66788429904357 5.09499802417961 4.80241956207367 4.47488644156056

TCGA-69-7979 1.11780821917808 0 6.02063975954864 5.46251678286192 6.09640430055241 5.0276555512826 6.14838543563254 5.09204281631416 4.39336202373133

TCGA-44-2662 3.50684931506849 0 4.57643710935277 4.46421492033324 5.01501081057102 5.04969156106796 5.43447837493162 5.11230705609135 6.36540629336816

TCGA-L9-A743 1.81917808219178 0 5.27302778977192 5.39921282158178 4.95309114253302 4.7136245551658 5.15124385212914 5.32313419531973 5.04401576942903

TCGA-50-5049 8.47671232876712 0 6.69623099107954 5.5217140633467 5.25733086237873 5.23549462353562 4.91396920913763 5.07359404760905 5.03493977671219

TCGA-05-5428 1.83561643835616 0 4.12188024296367 4.97338140922844 6.3295656418649 6.63864479412907 4.6869664694162 4.28823479686995 4.77731504594479

TCGA-55-7281 2.38904109589041 0 5.23053317285912 5.25337206309265 4.37504842723099 4.40378758807867 4.81372041343819 4.37504842723099 4.75329651494594

TCGA-67-6217 1.15616438356164 0 4.89594974385834 4.79922658685188 5.1601514454942 5.26380639199806 4.95079473345915 4.03598711655745 4.91396920913763

TCGA-MN-A4N5 0.23013698630137 0 4.86032344958086 5.16590948244236 4.62195132384969 5.49376891158473 5.07359404760905 5.06587349893486 3.91694385317839

TCGA-55-7724 1.93150684931507 0 4.23419056213206 5.32003253913737 6.61888746744597 5.92153242381988 6.02969354559291 4.92791250435688 5.5348321339118

TCGA-MN-A4N1 2.26575342465753 0 6.27768789026263 5.19020200046397 4.68021316778459 4.96653293861653 5.83175275787624 5.31680769598627 4.8521561569892

TCGA-69-A59K 1.61917808219178 0 4.41588575730423 3.82773477327855 4.26420472669646 4.39866922169541 5.94544404570681 4.54993794022504 4.61151990003138

TCGA-91-8497 1.18904109589041 1 5.91644891986612 4.98611100920672 4.23419056213206 4.55780563820776 4.14857109588688 4.97338140922844 4.26420472669646

TCGA-44-3396 3.0958904109589 0 4.6074822239846 4.87448825782311 4.82982726463627 5.19824982713734 5.33020134034042 4.89276658901782 4.91640747301757

TCGA-38-4627 3.14246575342466 1 4.79922658685188 4.8641033809139 5.7005554922005 5.31680769598627 5.17264592701774 5.46661360744635 5.10972413689344

TCGA-55-A57B 1.4958904109589 0 4.56046429082293 4.65669749616631 5.72044308787751 5.02298388111108 5.35017070969008 4.87448825782311 4.01291492082692

TCGA-NJ-A7XG 1.69041095890411 0 4.87448825782311 4.48813492886391 4.46930389429201 4.42842197890458 4.51222152885824 4.94812177498575 3.75004255696854

TCGA-49-AAQV 1.85479452054795 1 5.29898921272872 5.31680769598627 6.1878687357199 6.08894364647097 4.84679765133552 5.29580550575862 5.14623884118183

TCGA-55-8091 1.64383561643836 0 5.2215362497871 5.48385238471085 6.14838543563254 5.66509577421623 5.08434729206195 5.7005554922005 4.36212613551082

TCGA-64-5779 2.36712328767123 0 5.4470097069513 4.854651044916 5.08434729206195 5.4470097069513 5.1601514454942 5.43149212334626 7.32568041540727

TCGA-86-8278 2.58630136986301 0 5.12911876825784 5.25733086237873 5.2215362497871 5.20693533373832 5.42816930855285 5.25733086237873 4.76882838343843

TCGA-44-6777 2.7041095890411 1 4.5414927281635 4.54993794022504 5.14194365676337 4.08919262478158 5.25093854069401 5.10227338093085 4.76444119290761

TCGA-55-8092 0.421917808219178 1 6.61888746744597 5.7797042285245 6.35474846268005 4.65447126593921 4.93878716007944 5.37054702248304 5.14850696063783

TCGA-73-4675 2.52602739726027 1 5.66509577421623 4.23419056213206 4.94576628955782 4.52825334021209 5.06842105631952 5.05307049743809 4.9331128359336

TCGA-55-6980 5.77808219178082 0 4.6869664694162 4.57643710935277 5.62490569127957 4.87448825782311 5.18693725193171 5.03895492483204 5.99338881275725

TCGA-50-8457 3.08219178082192 0 5.20693533373832 4.79325688415672 4.34752525368611 3.68589193779175 4.62838436729772 4.96407345132971 4.51222152885824

TCGA-55-6971 3.83561643835616 0 5.26380639199806 4.8995725669227 5.95550151204503 5.34048776516519 5.14416406346 5.67540425515099 4.41588575730423

TCGA-64-1679 6.81643835616438 0 4.88922252922638 4.48505801352703 5.05307049743809 4.10546304343291 5.70549590416465 4.91165818347331 5.58838427993837

TCGA-95-7948 1.3041095890411 0 5.00599953960783 4.46021633795082 4.52825334021209 4.01291492082692 5.05874335544962 5.14416406346 5.7136548452647

TCGA-78-7158 0.49041095890411 1 4.74992100693531 5.37818449048402 5.5718992101466 4.95585928052126 5.01204991644541 4.58974475128415 4.65447126593921

TCGA-49-6767 1.85479452054795 0 4.08919262478158 6.61888746744597 5.6412904121533 5.83175275787624 5.47761875985422 5.7136548452647 6.3295656418649

TCGA-86-8055 0.33972602739726 1 4.4096376642013 4.82202165175364 5.54279929638393 5.49844091093089 6.00945265646471 5.48385238471085 4.82439249979648

TCGA-49-6745 1.43013698630137 0 4.28823479686995 5.34048776516519 5.1151919660021 4.8719829923562 5.36772483343786 4.74044841881869 6.06381540714332

TCGA-86-7711 2.86575342465753 1 4.90185151980137 4.43449486931811 4.5697629915264 4.42237712992835 5.8832445358893 4.71128869450524 6.23103018595568

TCGA-50-5946 4.43013698630137 0 5.12705768485864 5.41914273046275 5.03895492483204 5.01698137111624 5.93522956651236 4.67494772988294 4.71622628584955

TCGA-93-A4JQ 1.44109589041096 0 5.7136548452647 5.13217960845189 4.80241956207367 6.02969354559291 5.13217960845189 5.23887541246387 4.17303206018344

TCGA-78-7540 3.27945205479452 1 4.39336202373133 4.93071637995424 4.67132283096329 4.88429666028332 5.10227338093085 5.81276325671543 5.10227338093085

TCGA-86-7713 3.16986301369863 0 5.48385238471085 5.61612912359804 4.91396920913763 4.58632574928033 4.67132283096329 4.13498905520495 4.94576628955782

TCGA-50-5051 1.30958904109589 1 4.61522851216861 5.8672628002617 4.77135066936307 5.06049154834126 5.00091037668946 5.06344998060719 4.94322806346756

TCGA-55-8204 1.41095890410959 0 5.63356955900206 5.08161845529761 6.23103018595568 5.59673720348444 5.5562466763786 6.20350903070615 5.19824982713734

TCGA-73-A9RS 0.931506849315068 1 4.4788438434817 5.38765365796728 4.70816107084232 6.1878687357199 4.854651044916 4.8189648954976 4.72913220217783

TCGA-05-4415 0.249315068493151 1 4.20387133212243 6.46861263233495 6.67038688371618 5.90057358126119 5.98599552121818 6.63864479412907 5.56780278985751

TCGA-44-6148 1.92876712328767 0 5.55170255595065 4.75930034919142 4.73272544830853 4.25373755333422 4.50115175236105 5.16341170598797 4.58246995167545

TCGA-95-7947 1.30684931506849 0 5.13217960845189 6.30162242979033 5.56780278985751 4.77731504594479 5.86026539501079 5.03493977671219 5.75748655020916

TCGA-97-7937 1.54520547945205 0 5.61099127224392 4.39336202373133 4.82439249979648 5.97462084300779 4.66788429904357 4.14857109588688 5.59673720348444

TCGA-99-8032 0.120547945205479 0 5.72791149551452 6.04491601986667 4.54993794022504 4.6869664694162 4.25373755333422 5.30915450537318 5.12705768485864

TCGA-86-8669 2.56986301369863 0 5.63738960708029 5.28944198619386 4.51222152885824 4.50868799266451 5.51092920157204 5.24389641740117 5.25093854069401

TCGA-38-4625 8.14520547945206 0 4.84885337036964 5.81276325671543 5.11977511833695 4.57643710935277 5.74664690454395 5.48794148586485 7.01396439607183

TCGA-55-7283 1.66849315068493 0 4.94812177498575 4.77434755796381 4.55524310280527 4.03598711655745 4.80845387453467 4.84885337036964 4.78894039050948

TCGA-44-2666 0.265753424657534 1 5.47761875985422 4.74044841881869 5.00599953960783 5.22737883793572 4.9331128359336 4.95818428218915 4.85765100664681

TCGA-55-7907 0.93972602739726 1 5.37054702248304 4.82982726463627 5.69102369655262 5.60412104568954 5.34048776516519 4.5697629915264 5.47183251949017

TCGA-55-6975 0.323287671232877 1 4.55524310280527 5.66509577421623 5.93522956651236 5.7797042285245 5.95550151204503 6.02063975954864 5.51092920157204

TCGA-55-8203 1.4986301369863 0 5.7725028549258 5.28021882690186 5.29898921272872 5.54660541066437 4.55524310280527 5.49844091093089 4.77434755796381

TCGA-50-5933 6.55616438356164 1 4.6368245983018 4.89594974385834 5.06049154834126 4.77434755796381 5.11977511833695 5.11713015001565 5.48794148586485

TCGA-44-2661 3.17534246575342 0 5.24794870620138 4.44491089699902 5.19824982713734 5.8175071996365 4.21695209102212 4.59509417438844 4.93071637995424

TCGA-05-4422 1 0 6.23103018595568 5.27766157790134 5.22490800517098 4.95079473345915 3.94850930038125 4.45481598126603 5.29282914439146

TCGA-S2-AA1A 1.40547945205479 0 6.11913736922898 4.66788429904357 4.6869664694162 4.64761300444413 4.8641033809139 5.73197212832148 4.27104693944814

TCGA-38-4628 4.08767123287671 1 5.4379337879895 5.14850696063783 5.31680769598627 5.56780278985751 5.58263716200823 4.10546304343291 5.68686907176196

TCGA-78-7166 0.706849315068493 1 5.04401576942903 6.17826740809128 3.99576122753024 5.11977511833695 3.45300333262003 5.63356955900206 5.12396900081901

TCGA-50-6593 0.920547945205479 1 5.00886733426488 5.1151919660021 5.01698137111624 4.70572358491324 5.33697673575084 4.69000480261886 5.08717531991403

TCGA-44-3398 3.18630136986301 0 4.6249569887363 5.82446290729509 4.93878716007944 6.59109505615328 4.27104693944814 5.28562966294167 4.93878716007944

TCGA-MP-A4TI 1.17534246575342 1 5.51672658547113 5.50677723493176 5.19293679099683 5.11230705609135 5.23549462353562 5.24124956397002 5.09900251472474

TCGA-55-8302 1.30958904109589 0 4.77135066936307 5.48794148586485 5.49844091093089 5.25733086237873 5.1151919660021 5.61612912359804 6.52675813391858

TCGA-91-6830 0.164383561643836 0 5.28944198619386 4.81372041343819 4.854651044916 4.54532091053601 5.31680769598627 5.00599953960783 5.57593821979501

TCGA-44-8120 0.712328767123288 0 4.46930389429201 4.77135066936307 4.95079473345915 4.3307752985421 5.44313129089287 4.5052589650689 5.19020200046397

TCGA-55-6981 3.77808219178082 1 4.75638248910094 3.94850930038125 5.28562966294167 5.28021882690186 5.66509577421623 5.66509577421623 5.68074054932273

TCGA-86-8668 1.15890410958904 0 5.29580550575862 3.68589193779175 4.677739616492 4.20387133212243 4.82202165175364 4.72190294546832 3.99576122753024

TCGA-50-5941 4.03835616438356 0 5.41573566371201 5.3806123981936 5.87125254816225 5.14850696063783 5.5348321339118 4.81696587622992 4.62838436729772

TCGA-55-6968 3.54246575342466 1 6.49923579053703 5.6598948198276 5.70549590416465 5.41166427036383 5.845561356816 5.15457487587607 6.43065932304858

TCGA-67-3771 1.67123287671233 0 5.16590948244236 5.09499802417961 7.23601681628151 7.01396439607183 6.34327930434214 4.87954367252006 4.39866922169541

TCGA-50-6597 3.47397260273973 1 5.50677723493176 7.82085842471194 3.75004255696854 3.91694385317839 4.18538808950802 4.25373755333422 5.8672628002617

TCGA-44-A47B 0.786301369863014 0 5.19293679099683 4.3558475242814 5.13409318935653 5.72791149551452 5.03208295984944 4.63179647925294 5.03700330074137

TCGA-50-5935 1.78904109589041 1 5.62490569127957 5.24794870620138 5.12705768485864 6.21932743488592 4.56046429082293 4.82670556068671 4.95079473345915

TCGA-69-7973 0.63013698630137 0 4.93878716007944 4.3307752985421 5.7136548452647 5.41573566371201 5.06049154834126 5.39469067466805 5.17569818810011

TCGA-44-6779 1.36986301369863 1 4.71622628584955 5.8175071996365 6.45497186177606 5.56315286868292 6.27768789026263 6.38298797716996 4.86993515108222

TCGA-69-8253 1.16712328767123 0 4.50115175236105 5.87667250987434 4.98611100920672 4.91396920913763 4.51610407080754 5.69587355706738 4.7368855347186

TCGA-05-4249 4.17260273972603 0 5.04969156106796 5.17264592701774 5.40361501200538 5.14416406346 5.08717531991403 5.35406029799732 4.80845387453467

TCGA-75-7025 9.05479452054795 0 5.35406029799732 3.87935730457942 5.08717531991403 5.12705768485864 5.01698137111624 5.08434729206195 4.69379767353218

TCGA-MP-A4TK 1.59452054794521 1 6.00081278626219 5.12396900081901 4.13498905520495 5.08717531991403 5.25733086237873 5.83175275787624 5.07915546440747

TCGA-MP-A4TA 2.6027397260274 1 7.32568041540727 5.29580550575862 4.87448825782311 5.61612912359804 5.75748655020916 5.28021882690186 5.61612912359804

TCGA-97-8179 1.19178082191781 0 5.1070429609878 4.89276658901782 5.32003253913737 5.22490800517098 4.98891258099903 4.47488644156056 4.61850584999048

TCGA-49-AARN 3.10958904109589 1 6.08894364647097 5.15124385212914 4.99149081263546 5.3806123981936 5.20186164859352 6.09640430055241 4.89276658901782

TCGA-55-6985 3.37808219178082 0 5.80786071299967 4.52825334021209 5.14623884118183 5.05874335544962 5.22490800517098 5.27302778977192 4.677739616492

TCGA-L9-A443 0.528767123287671 1 4.76882838343843 4.80845387453467 3.94850930038125 5.42816930855285 3.99576122753024 4.83664432021799 4.6249569887363

TCGA-97-A4M2 1.70958904109589 0 5.87667250987434 4.5052589650689 4.5414927281635 4.68363218591554 4.71622628584955 4.72378149985436 4.58974475128415

TCGA-55-7815 2.11780821917808 0 3.87935730457942 5.09022163821186 6.00945265646471 6.26210027520436 4.32272118873298 4.12188024296367 4.6368245983018

TCGA-49-AAR2 6.09315068493151 0 4.99821565600027 6.43065932304858 4.05284946030268 4.88209051097053 4.59509417438844 4.6368245983018 4.8719829923562

TCGA-55-7576 1.83561643835616 0 5.58838427993837 5.69587355706738 5.33422367054468 4.72913220217783 5.47183251949017 4.80845387453467 4.9954899727389

TCGA-78-7160 1.90958904109589 1 4.70572358491324 5.766502096287 4.9954899727389 4.91165818347331 4.95585928052126 5.36772483343786 5.07043908254873

TCGA-55-8510 1.47671232876712 0 4.81696587622992 4.8521561569892 4.7136245551658 4.82202165175364 5.19605453656112 5.20418528097581 5.87667250987434

TCGA-73-4662 6.89041095890411 0 6.10652971501267 4.44919623910713 5.04401576942903 4.48505801352703 5.18413626987738 4.46930389429201 4.81372041343819

TCGA-55-8206 2.43287671232877 0 5.01204991644541 4.36899298148057 5.39921282158178 5.0252100478494 4.26420472669646 4.88429666028332 4.98030847358445

TCGA-49-4512 2.47945205479452 1 5.73197212832148 5.40361501200538 4.28174789628393 4.47488644156056 4.3558475242814 4.4096376642013 5.33020134034042

TCGA-86-8279 2.6 0 6.38298797716996 4.72190294546832 4.71827468816372 4.74044841881869 4.89276658901782 4.72619071303912 5.20418528097581

TCGA-38-4632 3.71780821917808 1 5.41914273046275 5.33697673575084 4.86032344958086 5.45074474082551 4.93557461567931 5.5217140633467 5.04969156106796

TCGA-38-4631 0.96986301369863 1 4.44919623910713 5.8832445358893 6.46861263233495 7.23601681628151 6.13641661524228 5.86026539501079 5.97462084300779

TCGA-95-7562 0.238356164383562 1 4.97663580279272 5.85265234899436 7.32568041540727 6.04491601986667 5.59282272526322 4.61151990003138 4.96653293861653

TCGA-05-4430 2.08493150684932 0 5.46251678286192 5.07359404760905 5.16590948244236 5.10972413689344 5.48794148586485 5.32003253913737 5.5217140633467

TCGA-95-7043 1.37808219178082 1 4.17303206018344 5.52696972064213 5.61612912359804 5.21252572577479 5.69102369655262 4.86707704259779 5.69587355706738

TCGA-78-7535 2.6 1 5.36772483343786 5.53896169362172 3.45300333262003 4.22582309670354 3.82773477327855 4.74315870529341 4.99821565600027

TCGA-91-8499 0.0986301369863014 0 4.29941848627659 6.52675813391858 6.17826740809128 6.7917544053942 6.12904916587147 4.82982726463627 6.38298797716996

TCGA-55-6986 8.93424657534247 0 4.95818428218915 5.29282914439146 5.18413626987738 5.16341170598797 5.29282914439146 5.6598948198276 5.39163201969549

TCGA-78-7143 13.5917808219178 1 5.8832445358893 4.42237712992835 4.87725025464731 5.31418739394226 5.37054702248304 4.65120580746482 5.60412104568954

TCGA-69-8255 0.353424657534247 0 5.35017070969008 5.26380639199806 4.9183976637933 5.45948587463351 4.98327017057208 5.19824982713734 5.23053317285912

TCGA-05-4384 1.16712328767123 0 5.01698137111624 4.91640747301757 5.20186164859352 5.06842105631952 5.35406029799732 4.51222152885824 4.08919262478158

TCGA-73-7499 4.19452054794521 1 4.74315870529341 7.32568041540727 6.30162242979033 6.40432570602522 6.17826740809128 4.43449486931811 4.98327017057208

TCGA-NJ-A4YQ 3.92328767123288 0 5.54279929638393 5.15661664469614 5.07359404760905 4.6368245983018 5.69587355706738 5.07043908254873 5.49844091093089

TCGA-62-8398 1.21643835616438 1 5.28021882690186 4.90679500785572 4.79325688415672 4.21695209102212 5.10972413689344 6.82600730414282 5.8832445358893

TCGA-MP-A4SW 4.87123287671233 1 5.20186164859352 4.91165818347331 4.81696587622992 4.9183976637933 4.99341017564513 4.76882838343843 4.99149081263546

TCGA-73-4677 0.104109589041096 1 4.77731504594479 5.12186035974213 5.19605453656112 5.20186164859352 5.05307049743809 4.52492793712345 4.43449486931811

TCGA-55-7913 1.53698630136986 1 6.43065932304858 5.6412904121533 5.73197212832148 5.51092920157204 5.87125254816225 6.36540629336816 5.31680769598627

TCGA-44-7669 1.57260273972603 1 4.65447126593921 5.16855014735371 5.37464850389324 4.72378149985436 6.49923579053703 4.36899298148057 6.26210027520436

TCGA-55-8505 1.20547945205479 0 4.72190294546832 4.59222214082247 4.92075349471314 4.89594974385834 5.62868387907542 5.93522956651236 5.43149212334626

TCGA-62-A471 3.41369863013699 0 4.05284946030268 6.82600730414282 4.98327017057208 5.40361501200538 5.78809474521384 6.49923579053703 6.61888746744597

TCGA-78-7161 0.797260273972603 1 4.91640747301757 5.19824982713734 5.25337206309265 6.09640430055241 4.05284946030268 5.40361501200538 4.55524310280527

TCGA-53-A4EZ 2.93424657534247 0 4.47488644156056 4.81696587622992 5.12911876825784 5.78809474521384 5.29898921272872 5.37464850389324 5.37054702248304

TCGA-62-8397 3.53150684931507 0 5.05874335544962 4.56501479720614 4.47488644156056 4.28823479686995 4.38648567886514 4.32272118873298 4.29941848627659

TCGA-38-7271 2.19178082191781 1 5.7797042285245 4.66300647702884 4.69000480261886 4.45481598126603 4.91640747301757 4.90185151980137 4.52492793712345

TCGA-80-5608 7.75890410958904 0 5.59673720348444 4.82439249979648 5.12186035974213 4.94105936490657 4.84387806789306 4.92075349471314 5.2152621214988

TCGA-55-8207 2.67671232876712 0 4.30579401671983 4.26420472669646 5.21845791601729 4.46021633795082 4.77731504594479 4.52825334021209 4.21695209102212

TCGA-44-7661 1.52602739726027 1 4.90373698575988 5.24389641740117 5.4470097069513 4.98327017057208 5.53896169362172 5.36481021747551 5.83804452921552

TCGA-J2-8194 1.98356164383562 0 4.82439249979648 5.05500763581931 5.06842105631952 4.99341017564513 4.77135066936307 5.04680132896077 4.8399123619932

TCGA-50-5932 3.38356164383562 1 4.69988820108664 4.37504842723099 5.43447837493162 5.48794148586485 4.70572358491324 4.97663580279272 4.9183976637933

TCGA-44-2657 3.7013698630137 0 5.85265234899436 4.7368855347186 4.83304009008021 4.94322806346756 4.74992100693531 5.06842105631952 4.83304009008021

TCGA-MP-A5C7 6.15890410958904 0 5.93522956651236 4.95818428218915 4.81372041343819 5.00091037668946 4.84885337036964 4.59222214082247 4.46930389429201

TCGA-55-8087 1.26575342465753 0 5.81276325671543 5.03493977671219 4.74992100693531 4.81372041343819 4.50868799266451 5.16590948244236 4.98891258099903

TCGA-55-8621 1.41095890410959 0 6.1878687357199 5.09204281631416 4.99821565600027 4.50115175236105 4.83304009008021 5.21252572577479 4.78071114469456

TCGA-55-A492 1.63287671232877 0 5.3806123981936 5.59673720348444 4.43914420742014 4.70302126184901 4.69641108273487 4.53806324755926 4.59509417438844

TCGA-55-8085 2.47671232876712 0 4.63179647925294 5.73197212832148 5.63738960708029 6.52675813391858 5.41573566371201 4.89594974385834 5.44313129089287

TCGA-05-4390 3.08493150684932 0 4.21695209102212 4.08919262478158 6.7917544053942 6.27768789026263 5.16341170598797 5.26933752300249 4.67132283096329

TCGA-80-5611 7.10958904109589 0 6.34327930434214 5.55170255595065 6.38298797716996 5.95550151204503 4.88922252922638 4.67132283096329 5.90057358126119

TCGA-91-A4BD 1.65205479452055 0 5.34048776516519 5.40686320881734 4.36899298148057 5.12186035974213 4.57643710935277 4.067075734095 4.72378149985436

TCGA-78-8655 6.46575342465753 0 5.70549590416465 5.49844091093089 4.78894039050948 4.75930034919142 4.97663580279272 5.17264592701774 4.28174789628393

TCGA-49-4494 2.96164383561644 1 4.82982726463627 5.33020134034042 5.2152621214988 5.36772483343786 5.45948587463351 5.54279929638393 5.3806123981936

TCGA-86-6851 0.49041095890411 0 6.12904916587147 4.69641108273487 5.47761875985422 4.84885337036964 5.6412904121533 4.66300647702884 4.10546304343291

TCGA-86-8359 1.21643835616438 1 4.44491089699902 5.30129551200087 4.29941848627659 5.20418528097581 4.82439249979648 6.43065932304858 4.25373755333422

TCGA-64-1680 3.08493150684932 0 4.61151990003138 4.62838436729772 4.42237712992835 4.6074822239846 4.6368245983018 4.43914420742014 4.53806324755926

TCGA-55-7284 0.665753424657534 1 5.44313129089287 4.64761300444413 4.87954367252006 4.26420472669646 4.90373698575988 6.7917544053942 5.51672658547113

TCGA-55-8096 1.96986301369863 1 4.94105936490657 5.00285032849301 5.64710093067358 5.34747421070946 4.65946063606462 6.04491601986667 5.41573566371201

TCGA-93-A4JP 1.58356164383562 0 5.22490800517098 4.74315870529341 5.21033319941472 5.25337206309265 4.8399123619932 5.33020134034042 5.27302778977192
