# Supplementary material for: Characterization of polyamine metabolism predicts prognosis, immune profile, and therapeutic efficacy in lung adenocarcinoma patients
Source: Front Cell Dev Biol. 2024 Apr 8;12:1331759. doi: 10.3389/fcell.2024.1331759 (PMC11033315; doi:10.3389/fcell.2024.1331759)
Supplement: Supplementary file 10 [file Table3.DOCX]

gene Mean1 Mean2 logFC pValue fdr

KIAA0319 2.03815150887065 0.62666969683391 -1.70148416312931 8.2648009077857e-05 0.00020895018009608

AL158801.3 0.0330580996517413 0.238615334532872 2.85161106166215 1.51910795274314e-05 4.45311923982046e-05

GRM5-AS1 0.0217259810447761 0.0108065742249135 -1.00751207487879 0.000303018742763339 0.000684074503885871

AC091588.1 0.0647888745273632 0.252118457508651 1.96028373976667 7.29682084741192e-15 1.12686646062516e-13

TEX26 0.109770728233831 0.335886477750865 1.61348032358431 0.000336554986119145 0.000752762419885395

AC141586.5 0.0673185164378109 0.156261573865052 1.21488776209869 4.12861119501431e-17 9.04063380173269e-16

AC104964.1 0.0405047495522388 0.0821804846712803 1.02070475193194 0.000111024493423824 0.000273609649949683

TMEM171 1.6848776719403 0.7486581083391 -1.17026491560104 4.03497272389023e-13 4.68207484083147e-12

STEAP1B 1.42986441373134 0.414191878062284 -1.78750718159205 8.16372824357591e-13 8.970589291354e-12

RPS2P45 0.0187421963134328 0.0657858890588235 1.81148813612659 3.30439785638352e-15 5.35715953598632e-14

AC092681.2 0.0174158215323383 0.0482782215813149 1.47097400392132 3.39240346485727e-14 4.70021452704389e-13

AC092120.2 0.0306655585074627 0.202257826643599 2.7215043988654 0.0021978521073165 0.00415506014620213

LINC02712 0.075652756318408 0.189467646366782 1.32448696237837 1.94782053138127e-11 1.68265608127851e-10

PTPRJ-AS1 0.0784046852736318 0.223974756608997 1.51432436648282 0.00330000781364921 0.00600620308134065

LINC01224 0.885644953970149 0.388380271595156 -1.18925853436291 2.35063311446604e-06 8.01518709395913e-06

ATP5MC2P2 0.0439750476616915 0.110419290553633 1.32823519047098 7.74386511875627e-06 2.40375338778781e-05

C1QL4 0.156337934547264 0.0536657051626298 -1.54259554315978 3.67476126286255e-15 5.91204238466416e-14

AGBL1 0.0153909879850746 0.0534243127647059 1.79541059982856 7.74201742377874e-08 3.53398086437006e-07

AC009542.1 0.0601252308706468 0.133070039107266 1.14614334943375 3.65948946521858e-09 2.10842715133196e-08

Z83843.1 1.00350821298507 2.06534687612457 1.04133167924772 3.99021933788013e-11 3.29705434716077e-10

SLC6A17 0.311990137522388 0.116758735072664 -1.41796993771356 9.74935882675647e-07 3.59311364926276e-06

AL136369.2 0.0263628580099502 0.0836674201038062 1.66615916799826 1.29651760077385e-10 9.7739130047312e-10

LINC01600 0.0585696881293532 0.022165040916955 -1.40186818669607 2.52828118344163e-12 2.5544325957565e-11

LINC00540 0.575092452905473 0.211592981415225 -1.44250213123211 4.11625428187183e-05 0.000110469585525655

ADGRG7 0.271093657248756 0.127663036636678 -1.08645048922617 0.00270508182253725 0.00502267398821411

AC024581.1 0.102144877860697 0.0461928916608997 -1.14487409435496 2.91294591342603e-07 1.19372296572074e-06

MTHFD2 26.9003813930348 13.2594587370242 -1.0206047428771 6.93546679460015e-35 1.42620313407755e-32

TEKT4 0.131819247104478 0.379558094743945 1.52575968429798 1.60494854115558e-07 6.89852940446411e-07

LINC01765 0.352960017761194 0.790787186366782 1.16378472570183 3.509998713267e-06 1.15899763940435e-05

CSAG1 7.41698960955224 1.61789493564014 -2.19671582420431 5.02350278618453e-11 4.07935870552693e-10

AC106875.1 0.637651962641791 0.112355319231834 -2.50470077263676 9.05040578578554e-08 4.07951959040776e-07

BEST3 0.0589017804378109 0.0110267323598616 -2.4173059145359 3.5949538888789e-10 2.48350565447936e-09

OR7E94P 0.0182180609452736 0.0448693356747405 1.30036041111088 4.72439747750856e-09 2.67087003122242e-08

SLCO1B1 0.234861125452736 0.0887384237923875 -1.40417710340928 0.000456820177905264 0.000994035473443311

KCNK16 0.032076626800995 0.138012870373702 2.10520847150008 3.39411820865895e-06 1.12343135673269e-05

OR13J1 0.0182149348507463 0.038386365550173 1.07547213619365 5.41362124696888e-05 0.000141795193549702

PATE2 0.0348137345323383 0.0113262257197232 -1.61998939625819 5.99931599820024e-08 2.79144764462022e-07

ANKRD18B 0.348117026293532 0.170399239387543 -1.03065348173623 8.70837803355674e-10 5.63192573227186e-09

MCM4 20.7659748159204 10.0363396539792 -1.04898839672853 3.88127167351106e-33 6.63454876690797e-31

EDNRB 2.11062425572139 4.23088383114187 1.00328930816639 6.71714563970634e-16 1.20074466173836e-14

TAF1L 0.0174323073084577 0.0408277628408305 1.22778698259597 0.00205631466566067 0.00391263434714201

CDH20 0.0586159274079602 0.228984067280277 1.96588257982188 6.02657931239615e-16 1.08510167343012e-14

LCNL1 0.0847698666915423 0.178172737093426 1.07165317790994 2.76720900390349e-09 1.63497874825579e-08

AL136164.3 0.0203454194825871 0.0757035745155709 1.89565739567387 7.95467358374445e-05 0.000201818480997598

ATOH7 0.180172109288557 0.0746666475778547 -1.27083983595989 1.00578124186174e-06 3.69509867993427e-06

CASC8 0.629847783810945 0.290386442698962 -1.11702911296349 7.70651924416343e-06 2.39297571898127e-05

AC092139.2 0.0197861580646766 0.0681638679204152 1.78451569548077 0.00337296195499092 0.00612471846162539

PTCHD1-AS 0.0454137204676617 0.0929355894463668 1.03310294603437 0.000341015768478923 0.000761369899420289

B3GNT6 4.28988649885075 9.42225856371972 1.13513344506655 3.71816935012432e-08 1.79414135013938e-07

TTLL10-AS1 0.0462593923134328 0.135764509678201 1.55328817342198 2.66891573902816e-08 1.32117367352797e-07

AL353804.2 0.185348259303483 0.464804573702422 1.32638569884725 3.56120313701265e-06 1.17503807211118e-05

LINC01612 0.610164060895522 1.48699671847751 1.28513235188647 1.43867785253243e-08 7.46496666036083e-08

TMEM163 3.38966960985075 7.56597286951557 1.1583809419689 1.17155580203416e-23 6.37018910251178e-22

POLE2 3.38869517761194 1.63301486228374 -1.05319194718114 1.04037323154984e-30 1.27597344766314e-28

AC129510.2 0.0695610648258706 0.141528673252595 1.02474244563736 3.51888283720061e-05 9.54965723332374e-05

MIR23A 0.562071699502488 1.56201288477509 1.47458027239402 1.34337684590308e-09 8.39035321659037e-09

GAS6-AS1 0.439162585621891 1.18574797013841 1.43297034415012 1.50848510308456e-20 5.33037048699777e-19

DNAH8-AS1 0.0260071604975124 0.0664198746089965 1.35270610706946 9.94512523130658e-09 5.32392200188364e-08

AC007663.2 0.0565754550895522 0.11808086983737 1.06152706449418 3.58000316791166e-09 2.06829502835623e-08

FAM83C 0.14755680680597 0.00919086291349481 -4.00492634380306 9.68868237024495e-05 0.000241422615548651

CENPI 1.99922281144279 0.894185845536332 -1.1607926538903 7.66320788418411e-34 1.39725823754957e-31

AP005436.2 0.0950204600995025 0.193072928823529 1.022835797484 0.00031281414980375 0.000703979840132688

KCNQ1OT1 0.0802184073333333 0.160814287653979 1.0033903613314 4.63476210970113e-08 2.19765505721786e-07

SLC10A2 0.0157290499900498 1.02743593734948 6.02947309474685 2.20665007834737e-08 1.10778046334069e-07

INMT 3.79887360696517 11.6214120155709 1.61314175084172 3.75312639615143e-27 3.02796480633456e-25

IGHV1-45 5.26634279955224 11.7948897680277 1.16328859737106 1.0424575935375e-05 3.15914802276107e-05

TENM1 0.504219530940298 1.09024120731834 1.11252344810677 2.53993132407975e-07 1.05125789518131e-06

NPY6R 0.0261853060995025 0.0711849740034602 1.44281527412107 3.43039818619411e-09 1.99069362173581e-08

PLPPR4 0.418726719353234 0.847702816193772 1.01754959917483 7.46319947308586e-14 9.78047463291319e-13

AC110741.1 0.208925744129353 1.69509147695502 3.02030095222389 1.85785346432372e-05 5.3452863716867e-05

LEMD1-AS1 0.0596443709751244 0.122172058595156 1.03445647779069 3.39084576939867e-10 2.35559135872628e-09

ELOVL3 1.34150635934826 0.49607995482699 -1.43520932530505 0.000639138045403901 0.00135142026145163

SNORA31 1.52640406268657 3.38394369134948 1.14856864593837 4.32397524791437e-10 2.9535645112502e-09

RXFP2 0.015178278318408 0.0421763359377163 1.47442561244573 2.29130989503666e-06 7.83341570365658e-06

AL049779.3 0.0561093439303483 0.124947501868512 1.1550091082426 4.39949219552873e-08 2.09627372034339e-07

CENPM 7.67924982238806 3.44093358200692 -1.1581653375256 1.47855904414696e-37 4.12638672014483e-35

PKHD1L1 0.0745642354577114 0.290076330989619 1.95987686592464 1.937835415909e-05 5.55089047042353e-05

AL162171.2 0.0435769800995025 0.105833124982699 1.28015312523219 1.88689425982736e-07 8.01842106996244e-07

AC116903.2 0.0448968250447761 0.0918948590415225 1.03337072781459 5.61256605907964e-07 2.17365734516891e-06

ST3GAL5 9.80247439353234 20.2323989584775 1.04544951741239 8.85261742459089e-27 6.95744501616554e-25

AC103563.2 0.0355292745273632 0.0793771873356401 1.15971621198478 1.16358170697377e-06 4.22516724452106e-06

NEIL1 1.55191985766169 3.26493953702422 1.07300221737973 1.15626886191938e-23 6.31216634201498e-22

AC090950.1 0.0201061559850746 0.0988846313079585 2.29810902916515 1.2894020587878e-08 6.75189475547508e-08

BOK-AS1 0.204473648109453 0.423941576782007 1.05195053590386 1.26492207612001e-08 6.64150869300869e-08

AC010624.3 0.0765012556218905 0.226456738131488 1.56568013465832 1.42092846301785e-06 5.07607020161157e-06

CDK5R2 1.20851220250249 0.438328296647059 -1.46314831889613 3.95807324793795e-08 1.90218420894576e-07

AC078899.4 0.100952193482587 0.306407014768166 1.60177706837188 0.00283136162091803 0.00523051268707248

ESYT3 1.06165348741294 2.76455713564014 1.38073542604853 3.31927779048638e-29 3.34989843431006e-27

TOGARAM2 0.103987223119403 0.282143508179931 1.44002287937042 8.84397578226938e-10 5.70747375283312e-09

AC007684.1 0.199056276467662 0.61166842799308 1.6195734496059 6.2280936927208e-05 0.000161000342623737

AC027544.1 0.0938803173631841 0.212904120484429 1.18130924753624 8.05612902408152e-06 2.49360716170925e-05

RN7SKP51 0.354452464477612 1.43308056321799 2.01545564947768 5.41513871020917e-26 3.91809639482066e-24

SFTPD 69.1129091820896 235.291707621453 1.7674233634279 1.31785300804011e-19 4.10048689077327e-18

NMRAL2P 4.31307289995522 1.10916591030796 -1.95924091922432 0.000209216772410808 0.000488815882917786

AC021517.1 0.010014783318408 0.0395844406401384 1.98280225800484 1.26990611319304e-08 6.66383963849379e-08

SLC9C1 0.0940710998606965 0.210518537394464 1.16212379940944 7.5299943998364e-07 2.83870455194534e-06

AC097374.1 0.0062277373681592 0.0132467136435986 1.0888544776439 0.000123948819945367 0.000302867883990511

MIR3659HG 0.0394819260696517 0.0925461374048443 1.22898040629766 5.41278712061139e-11 4.37469644647522e-10

C1orf87 0.172163390129353 0.554173908858131 1.68656039678655 4.16264242072867e-08 1.9924443508388e-07

ENPP7P12 0.0231076998507463 0.0491660882352941 1.08928991512007 0.00118756253693432 0.00237286932971608

LCMT1-AS2 0.0206851440597015 0.0619310424152249 1.58206772439523 0.00049027156019297 0.00105999424278875

AC005884.1 0.0915774055721393 0.235168413875433 1.36063070227458 2.07241154913286e-15 3.46881614864037e-14

CCNA2 13.0744316169154 4.41701310276817 -1.56560521258592 1.45367368200215e-46 3.3131646002299e-43

BIRC5 19.5593411024876 6.23391908304498 -1.64964643703698 2.03295598370121e-49 7.94304945060401e-46

AL353152.1 0.0463422624378109 0.149106273044983 1.68594057079951 1.20652097532472e-11 1.08262298802924e-10

FAM107A 1.3080742899005 2.9703899467128 1.18320785888642 3.20590937069662e-14 4.45084372023109e-13

AC010680.1 0.00371446611442786 0.0138601445432526 1.8997155328454 3.58923813490408e-06 1.18328909100321e-05

TERT 0.395208688318408 0.154843892190311 -1.35180018656355 9.49948288150502e-06 2.90170284195946e-05

TRIM51BP 0.113914844880597 0.243189027103806 1.09412236966272 2.74118425758928e-07 1.12874720633946e-06

LINC01422 0.0464380268706468 0.106923680508651 1.20320282454814 1.50507201979772e-13 1.87805583810076e-12

AC129492.2 0.193027088557214 0.420232215882353 1.12238344321344 3.36903347089425e-06 1.11661494703051e-05

ART3 0.114776495467662 0.0335802749930796 -1.7731412797619 0.000429560190022925 0.00093995289200152

AC124947.2 0.771339829402985 0.382114502858131 -1.01336159365124 2.18219835427522e-07 9.14964356728917e-07

RAD54L 3.29374935373134 1.4056114899654 -1.22853288407118 6.92132486277547e-33 1.13352236525095e-30

SNORD46 1.47139583333333 3.51831920010381 1.25770096641527 0.00165150513188764 0.00320393905487729

MGAT5B 0.647197992661692 0.174460672653979 -1.89130527585063 6.88074699600667e-05 0.00017652042992288

FOXM1 12.8139137850746 4.48914191107266 -1.51319958020223 1.46881439414728e-35 3.29279292458427e-33

LINC00518 0.0505951456069652 0.0201856443806228 -1.32566932869255 8.8513886514909e-09 4.78051894980798e-08

AC073592.5 0.0338817769154229 0.0858485784775087 1.34128470739559 3.01120508997432e-05 8.28952785211853e-05

MIR6769A 0.229527863681592 0.479850751211073 1.0639164505736 1.35728052942182e-08 7.073479893233e-08

ZNF385B 1.15999383752736 4.35538050143599 1.90868162221935 4.57227828978865e-15 7.26622958894361e-14

AC006058.3 0.189960673930348 0.0544918064359862 -1.8015895565957 0.00310851261431273 0.00569061713530476

AC092127.1 0.00867254360199005 0.0278512938131488 1.6832172546301 0.00304831832195921 0.00559015060383428

CCDC180 0.0623632937910448 0.143175690491349 1.19901752834068 7.75275321943489e-16 1.37955628205299e-14

PKIB 7.43426555318408 2.83605465121107 -1.39030488862189 2.17809140625025e-10 1.56847814536452e-09

GABRQ 0.192870236333333 0.0386183551695502 -2.32027190083808 3.90189534443404e-05 0.000105139970002665

ABCA3 17.5345845253731 45.8651251238754 1.38719432859775 1.69158048274686e-24 1.02582541470347e-22

AC008937.2 0.0366580737313433 0.161188378685121 2.13654484878013 2.52210464544793e-06 8.54871261036075e-06

AC118555.1 0.0195200712437811 0.101062728477509 2.37222081174574 0.000604530933567181 0.0012841880414029

TTLL10 0.174071190651741 0.575857913076125 1.72603543362372 1.21779917721772e-10 9.21130190574495e-10

SLC2A1 60.792136960199 24.4568045570934 -1.31364881462237 2.73784119409391e-25 1.81747467617642e-23

HS3ST5 0.331812657880597 0.680797941027682 1.03685775101202 8.3524766043984e-08 3.78965220853179e-07

AC011444.2 0.0465881404477612 0.181663064602076 1.96323047135263 0.000518819507310402 0.0011153225368398

BICRA-AS1 0.04676793960199 0.128274166435986 1.45563887427895 0.000160111824811656 0.000382584169893307

RSPO1 0.121197742174129 0.455258374418685 1.90932273293325 8.822586474402e-15 1.34249432241223e-13

PCCA-AS1 0.038674170199005 0.142285643598616 1.87934786115335 1.46084577280976e-07 6.33187510084738e-07

PLCE1-AS1 0.0233187117014925 0.0477402634186851 1.03371844129696 0.000310842766399982 0.000700061730981514

AC140479.1 0.0347693624875622 0.10637964567474 1.61333361686898 2.30930899053978e-09 1.38053772439919e-08

GRIA1 0.0960780053532338 0.334678670124567 1.80049850242814 1.04662603207911e-17 2.46982070555338e-16

RAB44 0.0703701917313433 0.178764377688581 1.34502293093847 1.96433455688243e-17 4.47378539855501e-16

AC012651.1 0.262662074353234 0.854974775605536 1.70267395118343 2.00531457724335e-28 1.89776310337736e-26

ZBBX 0.294579950840796 0.759858978937716 1.36707245218233 0.000811829620899911 0.00167979574304831

TPX2 29.1422032139303 9.15670120588235 -1.67021010083732 7.40895991859153e-45 9.21068426243083e-42

KIF4A 7.80499608855721 2.71997976539792 -1.5208019902521 1.29374962092955e-42 8.22884933312167e-40

AC239859.5 1.52090221233831 0.56442182200692 -1.43008172433078 1.15344935267838e-07 5.104322407836e-07

AC005592.1 0.0416566831791045 0.115362705771626 1.46955703006691 9.43230173466319e-13 1.0261473844194e-11

DUXAP1 0.0566065510447761 0.117297229307958 1.05112800556565 2.55795126204858e-05 7.16069263224449e-05

TBX4 1.5561349680597 3.30523414557093 1.08678528224558 4.73117282469617e-21 1.80470818347894e-19

CDCA5 10.4241380686567 3.73261361280277 -1.48167001906936 5.17714756584339e-42 2.88969359032279e-39

PRDM16-DT 0.254967908139303 0.90224797567474 1.82320832936781 3.46978203219601e-28 3.17386416657394e-26

AC008740.1 0.0938011016915423 0.296790704152249 1.66176913332446 7.60681456355352e-10 4.97005203805993e-09

TRIM16L 6.22098575771144 2.60028121038062 -1.25847554976585 1.66206118864889e-09 1.02197332530457e-08

UPK1B 11.9734077445323 2.35406457756747 -2.34660801273594 0.00167956405882995 0.0032511909554108

AF131215.2 0.00783188315920398 0.0692734489930796 3.14487135744675 1.00958107490886e-08 5.40036033615438e-08

AC013726.1 0.0361636992587065 0.0849657192975779 1.23233862001812 4.44237616673352e-18 1.11774598123424e-16

KCNJ4 0.0526994027711443 0.0128247851314879 -2.03885195737101 7.47495648181011e-05 0.000190549035117445

RPL21P30 0.0691700245273632 0.153738492249135 1.15225955167605 0.00258815393579507 0.00482917247537148

C6orf118 0.275518055985075 0.889597307910035 1.6910055537605 2.08271592928943e-08 1.05232367755525e-07

AC025271.4 0.0714249001094527 0.180324565619377 1.3360969299249 4.01938183026609e-16 7.47823762297807e-15

AL161618.1 0.112770564179104 0.349957005051903 1.63378714772228 3.74258082208838e-07 1.50153418635935e-06

AC009927.1 0.0482900930497512 0.123914153764706 1.35954183635301 0.0025919664644089 0.00483529655559535

AC011899.1 0.0219044364079602 0.051098177633218 1.22204874457068 6.3148849931645e-07 2.41758265065858e-06

DAAM2 2.39878366865672 5.11468316055363 1.09234181623447 6.34098112116877e-22 2.76155786089118e-20

GGTLC1 6.63525001646269 19.3360229907612 1.54306836106465 1.15957655694939e-23 6.31761331326012e-22

RN7SKP70 0.371846550099502 0.803813535570934 1.11215348418141 1.08221632488179e-13 1.38375953648981e-12

ABCA9-AS1 0.116306325552239 0.052802352816609 -1.13925544171823 0.00119959665523504 0.00239394151920309

GGTLC4P 0.223450713930348 0.47629652899654 1.09190338093756 1.03832887366708e-14 1.5552187675134e-13

FLACC1 0.172972993781095 0.404683749235294 1.22624810837289 8.00537748729547e-09 4.36236830717475e-08

FGF12 1.0642216919801 0.234822999013841 -2.1801530945241 4.61005815011456e-07 1.81495739751883e-06

TESMIN 1.68317772059701 0.565223346401384 -1.57429455146455 2.90688376638798e-23 1.50289737260324e-21

AC008268.1 2.90797260330846 7.55653616022837 1.3777113917975 6.31255261345964e-10 4.18237194714441e-09

RMST 0.0356831402587065 0.100373043442907 1.49205737689544 2.74939841926289e-09 1.62550900922698e-08

POU5F2 0.00905315975124378 0.0767787586747405 3.08421391811224 5.34635911236427e-05 0.000140221443923248

SPIN4-AS1 0.0323794345273632 0.0925542156401384 1.51522091245083 1.44574049205654e-06 5.15864350394604e-06

SLC13A5 0.253485057358209 0.108300889619377 -1.22685561081346 0.00329282191107066 0.00599489399340968

AP002518.1 0.0105157224577114 0.0265756587474048 1.33755748277252 0.000124578616056826 0.000304270865257563

CTSV 3.94098172865672 0.97412474366782 -2.01637662409936 7.0852090870736e-33 1.15345516983014e-30

ITPK1-AS1 0.00587945568656716 0.0212254610138408 1.85204138597643 0.00332855658860655 0.00605413464776147

MIR135B 0.173887510099502 0.486617547577855 1.48463403401326 2.30621138919986e-12 2.34304908969599e-11

LINC01518 0.781433582487562 0.272102278235294 -1.52197422145858 7.23072386942351e-07 2.73489555841146e-06

AC092944.2 0.0106901847761194 0.043448589550173 2.02302255455737 2.64859290922195e-09 1.56862312835038e-08

FCF1P7 0.146787642238806 0.374926021557093 1.35287544299213 5.21976063197602e-09 2.93383586692446e-08

AC087286.4 0.0552347817412935 0.298912202456747 2.43607285941572 1.92537378984575e-07 8.16418188407462e-07

AC079793.1 0.0769870812935323 0.206152644186851 1.42102468399945 0.00450499204095581 0.00796969808021613

AC073592.7 0.0405186855721393 0.138739086401384 1.77571501063796 0.000182079482368622 0.000430555581966275

RIMS1 0.101435898242786 0.269434426380623 1.40936588610851 4.5604240136873e-07 1.79645105537012e-06

PSMD10P1 0.214369046019901 0.534404842076125 1.31783647788664 2.91205282246915e-07 1.19353581139714e-06

HCN1 0.102031264223881 0.353603210138408 1.79312008431753 0.00010714886566485 0.000264869981555825

SNORD94 2.1671920358209 6.77159554878893 1.64366881015597 0.00158027931176134 0.00307688883209875

CDC45 6.65610973681592 2.40805840968858 -1.46680883242649 1.63128992678447e-42 9.91461766612339e-40

TTK 4.18654322039801 1.39972122283737 -1.58061999939219 1.98781681668827e-44 2.26528291401767e-41

LINC02043 0.0759014187711443 0.0369035040795848 -1.04036904288782 1.45055047673952e-07 6.29223719886215e-07

SLC17A3 0.0184392196517413 0.0524365237854671 1.50779444487797 1.56825328159885e-07 6.75886026658187e-07

APCDD1L 0.976458429208955 0.420466901474048 -1.21556638737148 9.01357035629937e-08 4.06733458579092e-07

LINC01730 0.407886091492537 0.869005121799308 1.09119836660299 3.00651545338649e-07 1.22804839081891e-06

AC092667.1 0.0225227445174129 0.0485850859930796 1.10913088408075 2.80529070274656e-08 1.38168018584762e-07

AC026471.3 1.12943807661692 2.38917603356401 1.08090798025688 6.63197873897479e-19 1.88353705618858e-17

HOXA10 1.69048641326866 0.614760245076125 -1.45934264321463 1.90628926462263e-10 1.39032030366477e-09

AC007000.2 0.0989570326865672 0.248554363737024 1.32868728653473 2.32949713296469e-07 9.70919637101254e-07

HDC 0.513222522741294 1.07400644847405 1.06534626595799 6.79672349643865e-15 1.05320332933483e-13

AQP3 112.640515839801 249.972839543253 1.15004550338993 4.162968188667e-18 1.05423314777817e-16

MLLT11 6.28000994975124 2.66125874705882 -1.23865805830299 0.00016037444168625 0.000383144739702912

ABCC2 7.71529479497512 1.03621558861592 -2.89639708954888 0.000131104562870134 0.000318871480168801

CAPN3 0.25039428580597 0.69983830650519 1.48281999520205 9.1350532124148e-28 8.08555680775226e-26

NUF2 6.68969270646766 2.48237650657439 -1.43021799339994 7.63073636949686e-41 3.47834399509565e-38

LINC01423 0.0918977623880597 0.33142795716263 1.8505936653277 0.000255897952999944 0.000587394797696053

C10orf90 0.097652690800995 0.0323553540553633 -1.59365533353828 0.000140584299550813 0.000339482658724593

CD1A 2.27082444430348 6.94030991564014 1.61178391026997 2.15286987313184e-15 3.59468809707911e-14

HOXD1 1.1602044358607 3.25621367657439 1.48881633369086 4.08739514623056e-11 3.37225512064573e-10

LINC01108 0.123603417885572 0.491468378131488 1.99137995698284 8.56550950709271e-05 0.00021583442511423

RNU6-418P 1.64282940920398 3.94557738415225 1.26405375627491 9.43965978268873e-13 1.02653954296834e-11

CALR3 0.0549921697313433 0.0224573743598616 -1.29203694707481 3.73819537997766e-05 0.000101037299775066

ANKRD44-IT1 0.0643459763233831 0.366392495529412 2.50946811247408 0.000488073253730403 0.00105549169680766

THBS4-AS1 0.0890811532985075 0.197157038695502 1.1461530756006 3.51628680799581e-14 4.8473006148531e-13

LPAL2 0.280936188497512 0.722538231349481 1.36283145065674 2.45538270403555e-27 2.04739990717598e-25

KRT79 0.0540432374825871 0.117606556515571 1.12178248444142 0.00021891099458395 0.000509723795493873

MTHFD2P7 0.0373040915422886 0.0147447144567474 -1.33913599137238 3.08100521386764e-10 2.15678250829997e-09

AL445430.2 0.0573570776616915 0.174980862560554 1.60915371908867 7.15426487347558e-07 2.70822345037449e-06

ARHGEF2-AS1 0.243477072835821 1.09655792128028 2.17112418703665 1.40403784331572e-17 3.25720075738632e-16

AC010255.1 0.0770632332835821 0.177852423771626 1.20656601313132 2.02986846714565e-05 5.79446674008877e-05

PLCH2 0.462917288487562 0.947780586712803 1.03379866700217 1.17855716495787e-11 1.05961664896771e-10

ALPL 21.8178478845771 54.9699203145329 1.33313358696977 1.32664635567428e-14 1.95599880472731e-13

LINC02830 0.804753464577114 0.352677238927336 -1.19019841256093 0.000101996193577399 0.000252932803911675

CMA1 0.129823296965174 0.307730700622837 1.24511908057652 7.25134284751537e-10 4.75369671331604e-09

ERCC6L 1.79162091791045 0.717393448096886 -1.32042894102454 1.76074485386282e-38 5.53521514404001e-36

NCAPGP1 0.0462245501044776 0.0924648193633218 1.00024528111414 2.1242067889998e-08 1.07032158583538e-07

COL4A2-AS2 0.0470683015472637 0.113203149238754 1.26608639393482 0.00240994859058023 0.00452599697537384

AL354733.2 0.0243680358855721 0.0588463412352941 1.27196274712548 8.78199554840614e-09 4.74866702745963e-08

AC037441.1 0.0234247950298507 0.0712737005224914 1.60533340671384 1.73313789928636e-20 6.04608693182168e-19

RAP2CP1 0.121454267960199 0.053436809550173 -1.18450740859758 1.64611307612938e-07 7.06658179754176e-07

RNU6-1280P 0.413191590497512 0.868961475017301 1.0724813252984 1.47686823132519e-07 6.39625433519302e-07

MAMDC2 1.47929971278607 3.67686233581315 1.3135607835185 2.15259343807455e-21 8.56964054313522e-20

SCN2B 0.152903753004975 0.314599830069204 1.04089407356685 2.59060203402779e-15 4.26311465888448e-14

AC036103.1 0.116159632985075 0.264711601937716 1.1883126273642 3.40094817799602e-17 7.51947717608659e-16

LINC01863 0.128307603880597 0.289928751972318 1.17609174000535 2.99346765008047e-07 1.22342110325315e-06

CFAP70 0.407908068059701 0.875159225086505 1.10130147984101 1.83119776262358e-07 7.79991571527097e-07

SERPIND1 2.76999886971144 9.62566508997232 1.79700084026083 1.35492199838349e-10 1.0158244545763e-09

UCA1 4.99448299200498 1.42079799915225 -1.81363388887105 0.000375423012783375 0.000830729724888779

AP000821.1 0.0110633750199005 0.0270107266816609 1.28774088908872 6.67483040272687e-10 4.40532363693484e-09

AC011458.2 0.0715542448756219 0.171648332076125 1.26234657767785 0.000185517856858558 0.000438086115099427

AL162511.1 1.75265156925373 4.80133259692042 1.45389566367742 3.01388733901749e-23 1.55235063506833e-21

AC112722.1 0.192510761442786 0.553690276574395 1.5241400913409 4.45324036399276e-23 2.25548377694818e-21

ZMYND10 1.3794344958209 3.13607698131488 1.18488402360602 2.49633904923689e-07 1.03525205453569e-06

AC098851.1 0.282731792985075 0.574894527058823 1.02386317419517 4.09231845318785e-10 2.80443271597814e-09

TXNRD1 105.16884718408 37.6242774567474 -1.48297163542104 2.90059427568615e-16 5.54764010070043e-15

LINC02428 0.292673512537313 0.114802326539792 -1.35014030582771 1.09601646499104e-05 3.30606047397209e-05

CCDC13-AS1 0.0682944112437811 0.14265331233218 1.06267381792156 3.39494697258327e-14 4.70135694684316e-13

AC092802.1 0.0717402042537313 0.145500496591696 1.02017031909014 2.20207022766978e-07 9.2258916554486e-07

CCDC58P3 0.0696752094029851 0.0344156490657439 -1.01758071435823 1.28124865122738e-08 6.71562871043864e-08

AC022497.1 0.271634839402985 0.823390901062284 1.59990897718813 7.35536196776964e-31 9.19907314851205e-29

AGTR2 1.16591292316418 3.81521192982007 1.7103031549237 1.04298022347413e-06 3.82072182052203e-06

AC236972.3 0.204666883104478 0.830681944186851 2.02101851561233 1.33774677119258e-21 5.52679368460983e-20

SEPTIN7P10 0.0128401001741294 0.041520293532872 1.69316018511955 0.000723245267994803 0.00151151565357693

SMCR5 0.0155880654875622 0.0421537856089965 1.43522030022387 1.1725622936558e-07 5.18086893885075e-07

AC020900.1 0.0665212289054726 0.197762138096886 1.57187951990991 1.60783770926371e-06 5.68615790450055e-06

PARD6BP1 0.0864823323383085 0.193142653044983 1.15918946323419 3.31151743502314e-05 9.03441414941475e-05

OVCH2 0.0828464540199005 0.182110634923875 1.13630332160547 4.26046677080029e-08 2.03499417012553e-07

HBA2 5.83500921154229 12.4306559256055 1.09109558617783 1.84143390785933e-06 6.43126259480943e-06

AC006116.5 0.0403579856716418 0.104110214256055 1.36718554417903 0.00279390171433755 0.00517004139967064

MIR6757 0.236230466666667 0.683043641868512 1.53178271865811 7.31302159749059e-13 8.10746415449403e-12

MIR548V 0.179306306467662 0.726134351903114 2.01781027455797 6.52177530245548e-07 2.49019216575685e-06

SLC46A2 0.9412041560199 3.66436541723183 1.9609837826883 9.13803816894866e-21 3.32790071798596e-19

AC104958.1 0.0737359522039801 0.18368942816263 1.31682847194542 2.2813656891792e-06 7.80332061018648e-06

HTR1D 1.85204618630846 0.828530896820069 -1.16049267372781 6.89165893574008e-10 4.53311380212821e-09

LINC02038 0.824289773731343 2.15445797491349 1.38610145557373 7.19977195546983e-13 7.99812197327782e-12

LINC00942 7.62845254943284 1.45168412049481 -2.39366286850416 0.00118466733913067 0.00236761021139866

OR7E62P 0.203465181691542 0.0658349656401384 -1.62785600901388 9.06514390475879e-09 4.88631623561594e-08

Z82217.1 0.0194860694278607 0.0624835110034602 1.6810324108811 0.000416050357922299 0.000912947471852927

ITPKB-IT1 0.0380168833830846 0.0816039647404844 1.10199898248043 1.42800448108893e-05 4.20770551150423e-05

AC011632.1 0.281716582353234 0.0847765530069204 -1.73250727279589 5.3814367020911e-06 1.71681201215667e-05

AC091544.2 0.106938780318408 0.239597310958477 1.16382658920319 4.42638112425912e-12 4.28688115256682e-11

DEPDC1 3.38516415189055 1.11670882574394 -1.59997273212644 2.47364318669252e-44 2.70616564624162e-41

GPR19 0.722799694975124 0.325439437266436 -1.15120680770429 7.39072371652357e-24 4.1591829968502e-22

AC002306.1 0.028834455920398 0.0676329728961938 1.22993297038447 1.27499514826377e-05 3.79859665631962e-05

AC073370.1 0.467191713034826 0.224309599515571 -1.05852332109411 0.00108134813456379 0.00218087688815867

TSPAN11 3.03906186368159 6.10260940439446 1.00580021120664 1.15756863632556e-11 1.04177368224758e-10

PCDHA1 0.255547798626866 0.0936771143217993 -1.44782462342684 0.000311543167699804 0.00070140810311077

AC016769.3 0.417842165174129 0.147254204636678 -1.50464925582656 4.67840971034476e-08 2.21719815591629e-07

AL353748.2 0.124438626915423 0.270143979446367 1.11829414871272 0.00019161407304547 0.000451002142667264

YWHAEP1 0.197370690845771 0.0742515432179931 -1.41041485255676 0.000154381208313868 0.000369957596371181

AP000688.2 0.10447359780597 0.320845132145329 1.61873869921934 1.10383045221233e-05 3.32706225126815e-05

AC131206.1 1.5207609038806 0.50626960650519 -1.58681556748803 5.62929555775459e-06 1.78962261425768e-05

SND1-IT1 0.028286297880597 0.122536436197232 2.11503552441851 0.00527971994734722 0.00919337496402537

AC011447.7 2.12578892527363 1.04239409865398 -1.02809753384208 0.00171170755954709 0.00330826102421122

AL035658.1 0.0832138412935323 0.18941029432526 1.18661931974808 1.34616311934629e-06 4.8317009598584e-06

AC023421.1 2.07284898163184 5.66045681686505 1.44930347576934 1.3420990753424e-09 8.38811922089e-09

TCF7L1-IT1 0.0374734057711443 0.138548623114187 1.88645336539294 0.0011114337446715 0.00223463301600864

GPR17 0.126374307532338 0.32266612299654 1.35233892903274 1.18028512211224e-15 2.04179621061162e-14

PI16 0.276786783522388 0.649251922875433 1.23000332530186 1.56347778155305e-07 6.74146576154437e-07

AC006518.1 0.0199050624875622 0.0475897144705882 1.25751439816329 4.73721881037655e-05 0.000125655062034525

EDA2R 0.542158257860696 1.35415464910035 1.32060656322428 1.32652030476556e-22 6.36497023426984e-21

LINC01096 0.100370929696517 0.0427430690069204 -1.23157907969988 5.06718110054646e-05 0.000133591096105596

AL513123.1 0.312878822636816 0.12094089083045 -1.37130190153895 1.92282605561736e-07 8.15569991463592e-07

AL354864.1 0.0565668744776119 0.0218766598269896 -1.37056497779097 2.50304916267555e-10 1.77767838481372e-09

CA1 0.0237117517960199 0.106018816069204 2.16064617852 3.89702823130472e-11 3.22980976139952e-10

DNASE2B 0.266193999328358 0.583396526574394 1.13199874704639 4.93034224576437e-10 3.33279437522629e-09

MYBL2 34.5974225930348 11.4025519487889 -1.60130782232436 8.56257144988437e-39 2.89118924881898e-36

RN7SL444P 0.18504939960199 0.636699003806228 1.78270105216911 0.00012346408110015 0.000301791278763884

AC105053.1 0.0670151207562189 0.166423704806228 1.31230238492414 3.91226269271877e-16 7.29980229789009e-15

AC026523.2 0.00451710910447761 0.0136629017958478 1.59679225740669 0.00065187065079371 0.00137598690277132

ERBB4 0.15657192738806 0.369291309743945 1.23793374571659 2.47570942337238e-17 5.58208184082725e-16

ATOH8 2.9306056238806 7.3284750484083 1.322314189845 1.21835558400703e-20 4.35582029053494e-19

LINC01234 0.574658349776119 0.105304569013841 -2.44813645478468 2.33072499559247e-15 3.86569609638897e-14

KCNV1 0.155108269343284 0.0471374742802768 -1.71832924004813 0.000255482691576912 0.000586589289340878

AC108063.1 0.104493745223881 0.318661769619377 1.6086093587868 4.28437032375345e-14 5.81526195308471e-13

LINC02016 0.0502727640298507 0.182376587370242 1.85907161847898 8.37747351457444e-06 2.58517319895759e-05

GREB1L 0.720997886691542 0.332930007041522 -1.11477612410342 1.21474895081053e-08 6.3977245916942e-08

SNORA13 0.395605128457711 0.88280002100346 1.15802553523483 0.00292147961520682 0.00538136230306483

MAGEB2 2.82539445340796 0.250013343737024 -3.49837529457068 1.20835457885222e-10 9.14963946057813e-10

GNA14-AS1 0.0898770797512438 0.272130317439446 1.59827253807541 4.73125515657817e-11 3.85381103543469e-10

AC010976.2 0.124571355223881 0.433096257820069 1.79771534332704 1.49933241739815e-16 3.01077398060495e-15

KL 0.523999300945274 1.06021861242215 1.01672498063181 2.31928538745751e-13 2.80674581181252e-12

AP001381.1 0.210937326069652 0.426056234636678 1.01422945509346 0.000314044906901562 0.000706575205968881

AL590233.1 0.0795729655223881 0.197426808477509 1.31096763444495 2.82280768521649e-05 7.82523719751378e-05

LGI3 0.385393055333333 1.72709355087197 2.16394375088996 5.76960183751237e-10 3.85062494524069e-09

LHFPL3-AS2 1.0980034980199 4.12287454607266 1.90876791199541 1.76588899348557e-17 4.04837082747949e-16

AC008278.2 0.0526230682089552 0.11111476816609 1.07828330382152 0.00428533005619303 0.0076170648623435

SRGAP3-AS4 0.036156627761194 0.10012425017301 1.4694594058225 3.75212012515304e-07 1.50447860171435e-06

CRTAC1 5.12994923114428 12.0173986191003 1.22810817940061 1.61146812615731e-13 2.00425890179183e-12

MAGEE2 0.0933506144079602 0.189562977636678 1.02194580495783 5.6800337676076e-08 2.6559911701841e-07

AC009093.3 0.304609640771144 0.662164188903114 1.12022738589835 3.54803540511229e-09 2.05155958413998e-08

AC007014.1 0.0227074140298507 0.0687427947058823 1.59804508686704 0.000334441174564778 0.00074864676087303

AC012349.1 0.0079620964079602 0.0218412206678201 1.45583324290245 9.96694456399148e-09 5.33455839188194e-08

AC108676.1 0.4053187999801 0.139224744 -1.54164145386248 9.96724194958925e-06 3.0333708583004e-05

ROBO2 0.220269025467662 0.666838730259516 1.59807126351422 1.27591663244238e-23 6.88290333280061e-22

NEIL3 2.97103827895522 0.929860862179931 -1.67588043024988 8.41074821644103e-33 1.36114771431753e-30

RGS22 0.177327681253731 0.447925116394464 1.33683980255634 9.65402363277435e-13 1.04776804109674e-11

LMNTD2-AS1 1.59611303652736 3.74402092377163 1.23002567090256 1.19636025768379e-13 1.51623971490508e-12

CDCA4P1 0.22257742079602 0.507520094290657 1.1891576972312 3.28201416284703e-13 3.8797525850691e-12

KRT16 10.5923336064677 4.29717977023875 -1.30155842790946 0.000770427511671357 0.00160147990727198

LINC02454 0.293526347313433 0.133437875847751 -1.13732177905861 0.000406681359467168 0.000894110545130791

TRMT9B 0.145577886238806 0.291340284031142 1.00091397670145 8.50566838354391e-17 1.78260559609139e-15

AC027130.1 0.0345526870646766 0.0745799270588235 1.10998947683139 0.000949834315813287 0.00193807583836865

PTHLH 3.41040288472637 1.55343175692042 -1.13448331623947 4.69710803978297e-05 0.000124675761731429

AC010261.1 0.0285859306965174 0.106569458961938 1.89841687767629 7.36661176096154e-06 2.29420213689704e-05

AC025470.2 0.013802515721393 0.039659587266436 1.52273841925971 2.00490073593259e-08 1.01544509495845e-07

LINC01775 0.24732093119403 0.116189219792388 -1.08990812307463 4.97528839544791e-12 4.78292223604571e-11

GS1-24F4.2 0.0984090670895522 0.0394596913252595 -1.31841157631471 0.00146899674593259 0.00288255567522287

AL133304.2 0.225217266517413 0.868508478650519 1.94722249565994 1.07903727783549e-10 8.24118110829395e-10

MIR3178 0.163948177114428 0.330630042560554 1.01197795949687 2.54399668270301e-06 8.61757608024862e-06

FOXD3 0.28769842800995 0.0970073331038062 -1.56839162357941 6.20605643527374e-09 3.4450100163332e-08

AC009970.1 0.0514195387064677 0.109256078235294 1.08732497062799 7.53446358739468e-07 2.83996112341847e-06

SHANK2-AS2 0.0461699417412935 0.112178385847751 1.28076891205585 0.000251528574244005 0.000578433238507823

AC090001.1 0.222660858308458 0.591456599688581 1.40942434122359 3.38780221697951e-06 1.12201974611758e-05

PDZRN4 0.071105610238806 0.243122234643599 1.77364654226789 4.33445407860219e-07 1.71559072430926e-06

AC005912.2 0.0108067109701493 0.0245860530657439 1.18591264509868 5.76477241696316e-06 1.828653741637e-05

NEK2 8.86514394328358 3.11333773598616 -1.50968196697573 5.27749432816503e-45 6.87330808930065e-42

ISM2 0.177310232791045 0.0437042390519031 -2.02043067385466 6.84204790952303e-09 3.77278246623901e-08

SKA1 3.63798641741294 1.22599846346021 -1.56918298572724 2.4880754155599e-41 1.2372520475557e-38

DBF4P1 0.117207283980099 0.0582236474705882 -1.0093851041931 2.32412475232243e-18 6.10612987281638e-17

MFFP2 0.151814487810945 0.0709139778546713 -1.09816754494116 9.79078301129173e-08 4.38405231432267e-07

INMT-MINDY4 0.00933338175621891 0.0317122772076125 1.76456966740014 4.35132263386926e-17 9.49032488327945e-16

LINC02772 0.266456898855721 0.928591006955017 1.80114112252046 3.63625339355765e-21 1.40072577906763e-19

MS4A4E 0.199701532537313 0.469906170795848 1.2345273090313 1.27336170824656e-16 2.58356400004031e-15

ATP13A4-AS1 0.175940854776119 1.26539018889273 2.84641988383051 3.23234488013655e-15 5.24345388325828e-14

MIR6069 0.12198391641791 0.248554749134948 1.0268727281602 1.00718736612661e-06 3.69951308938528e-06

CSAG3 1.36759239572139 0.404609216747405 -1.75703721694861 1.24935946102937e-10 9.43140526059985e-10

RPS29P11 5.49620710114428 11.139295216955 1.01914968416346 1.23108300730978e-18 3.36364837661563e-17

MYOCD 0.125216312402985 0.323171079031142 1.36787557549301 1.5257316816382e-18 4.08704813837461e-17

CHST5 0.0177024939800995 0.0507168023702422 1.5185111624578 3.16892362762078e-05 8.67741902437208e-05

CYP4Z2P 0.137023785497512 0.776524310176471 2.50260474340159 3.01764103139305e-25 1.9887345110506e-23

AC010343.3 0.279192433482587 0.074982897432526 -1.8966263636135 7.5507968606585e-11 5.92240591164353e-10

AL138962.1 0.153892789402985 0.0529194491695502 -1.54005568622341 6.65666678591145e-08 3.07481568307175e-07

MIR429 1.99408402238806 4.50727250069204 1.17652847497862 1.37510340421827e-14 2.01982159534746e-13

AC012213.4 0.951237477263682 0.305311774359862 -1.63952232791855 1.44592346662165e-11 1.28188028564351e-10

AC009264.1 0.0465914154129353 0.0208449484013841 -1.16036635815594 0.000100637263506961 0.00024992546598705

ITLN2 0.441975624835821 1.37886108540138 1.64143840695378 0.00132449507429925 0.00262062795934924

AC005479.1 0.403374896766169 1.04363509685121 1.37142415848986 2.19640640250235e-18 5.79283655819087e-17

HOXA11-AS 0.25577964719403 0.0720156894186851 -1.82851831811015 1.9500027042926e-07 8.25892986946533e-07

ESCO2 1.10383497243781 0.466152530865052 -1.24365049491867 5.61499991109591e-36 1.3238814445558e-33

AC011346.1 0.145642523885572 0.313807660276817 1.10744892295516 5.46933468434957e-18 1.35271748414319e-16

LRRC52-AS1 0.0884808510995025 0.289851349065744 1.71187603234499 4.59229171772868e-15 7.29379665969102e-14

RPS26P21 0.0495734074129353 0.125168880761246 1.33623759634244 3.49021309420334e-06 1.15286628172055e-05

SNORD123 0.442816669651741 0.986595712802768 1.15574948539006 2.81773853605598e-09 1.66160303926544e-08

MELK 8.98397095920398 3.24164301522491 -1.47062804088802 1.18877284965851e-39 4.51568575530003e-37

AC027290.2 0.253054078557214 0.522268658650519 1.0453464032258 3.67145799387955e-11 3.05861639148966e-10

TEKT2 1.30135260437313 2.62910312552249 1.01455881576121 4.11801373104241e-06 1.34320424023864e-05

TIMP4 1.00940524378109 0.41587617816609 -1.27927953311303 0.00116592533218427 0.00233493764223674

AC004466.3 0.260795496169154 0.561859817958478 1.10729127298601 1.70198947789138e-11 1.48767696453593e-10

TEKT1 0.608670889218905 1.78657005436678 1.55345821240042 9.34928399042616e-06 2.86053157107233e-05

NKX2-5 0.244824170363184 0.0448390181072664 -2.44891940448988 0.00291688704512659 0.00537435062545219

AC131009.2 0.221800466318408 0.07968587816609 -1.47686641932879 1.65719034276191e-06 5.83998916048682e-06

SHE 2.17410377820896 5.00134544429066 1.20189544851781 8.05213423974481e-24 4.47613559871993e-22

GALNT17 0.742639389004975 1.5013304583045 1.01550782221999 6.88974463293887e-21 2.53272198536126e-19

AL136981.1 0.0304591440995025 0.0636280516228374 1.06278754225597 0.000184994074228719 0.000437000166708884

NIPAL4 0.573609354686567 0.139955128636678 -2.03510419712612 7.0516806046258e-05 0.000180668350853879

GCLC 24.664511618408 7.39549143737024 -1.73771879676784 1.19339811278249e-16 2.43941990916301e-15

AC025278.1 0.0239789399004975 0.0595507241522491 1.3123511725603 5.01364921344516e-07 1.96254910530593e-06

MIR186 2.49087131940299 5.66945377750865 1.18655925305927 2.6729391519954e-16 5.14460843118045e-15

NEK10 0.140840830761194 0.308093362595156 1.12930195983874 0.000404657677412175 0.000890162267933965

ERICH3 0.257683704094527 0.824039281096886 1.67711180670731 2.85876835673274e-05 7.91529809239122e-05

MFSD13B 0.161981473756219 0.344050922560554 1.08679329452852 1.84309587412282e-10 1.34655815853403e-09

CEP57L1P1 0.024869893278607 0.0123766660622837 -1.00677757267098 2.79003255397327e-07 1.14713455128035e-06

LUZP2 0.0826783262587065 0.193203582432526 1.22454075702793 7.29317125570053e-12 6.84047441163956e-11

HMCN2 0.125207557298507 0.270671470602076 1.11222118885293 3.97923161464985e-20 1.32883986154668e-18

SLAMF9 1.52440244447761 0.740684368927336 -1.04131303007622 1.87054758127933e-09 1.13763567596152e-08

MCM2 18.849453278607 7.96449039446367 -1.2428687198613 5.24159395358677e-37 1.3784384099096e-34

MKRN3 0.277286239343284 0.0952502970622837 -1.54158052557349 6.62940888406272e-06 2.08144108574349e-05

CFAP157 0.688521962985075 1.62723301463668 1.24084627481898 3.88329268566818e-05 0.000104669414559007

MIR6774 0.199758430845771 0.567270821453287 1.50578126474585 6.36524062804158e-08 2.94866753348471e-07

AC109361.2 0.135020155024876 0.297147608269896 1.13800498830489 1.90908904970423e-07 8.10644084915552e-07

CDK8P2 0.202341173034826 0.613710926955017 1.60076935648397 0.000402755639115841 0.00088640594912837

PARM1 25.6494125577114 51.4701021418685 1.00480886104489 1.31903465791405e-20 4.69123509674243e-19

AQP7 0.624364993218905 1.4404882880692 1.20609637473563 2.59023797379168e-18 6.73412629117894e-17

CFAP299 0.0722879801691542 0.161650957349481 1.16105436641787 0.00150094433700979 0.00293807812891624

MIR200A 0.546555257412935 1.36844714795848 1.3241004495043 1.84220498988566e-13 2.26446321228642e-12

AP000941.1 0.117271940870647 0.241550839529412 1.04246899947374 1.51900880080635e-11 1.34232280135876e-10

AC005479.2 0.515748833134328 1.24647412515571 1.27311237788257 4.37997894728672e-19 1.27846770766587e-17

MIR6124 0.230357194029851 0.594578344290657 1.36799426456652 3.56815681244146e-07 1.43640107183212e-06

PRAC2 0.597621804776119 0.22413770650519 -1.41484741444458 7.55313538664632e-05 0.000192434329599233

APOBEC4 0.187140227572139 0.548404792629758 1.55112146626233 4.15002680168253e-07 1.64951653867195e-06

BVES-AS1 0.103788529144279 0.0375979762076125 -1.46492009087585 2.82884329754095e-12 2.84026667355892e-11

AC083805.1 0.101141754656716 0.210545956093426 1.05775645389396 2.65219652278716e-12 2.67166999530747e-11

AL162426.1 0.112155296268657 0.27992281200692 1.31953131267966 2.20402557180412e-19 6.66078446285555e-18

ASPA 0.11373198020398 0.273585743564014 1.26635507242813 7.26283771371159e-18 1.7609805981384e-16

ATP6V1B1-AS1 0.0849130434726368 0.0355040725743945 -1.25800166116223 0.00079984998986085 0.00165676289175206

RPS7P7 0.0213481361691542 0.0496777571626298 1.21848992193674 0.000124705697420634 0.000304499671855579

DUSP9 1.58255447910448 0.763890583612457 -1.0508172522154 8.52297502130776e-05 0.000214841812749094

AC093248.1 0.0513419062189055 0.107235935432526 1.06257967664135 1.04293741678033e-06 3.82072182052203e-06

AL022341.2 0.0993138827064677 0.220876414878893 1.1531720705838 1.01746794128446e-06 3.73406827891505e-06

AC007671.1 0.0775717687064677 0.178906261418685 1.20560027829251 3.11198938574525e-12 3.10404484683197e-11

AC018445.3 0.0099998811840796 0.0270074932110727 1.43337687921202 0.000129399366672869 0.00031497620848193

AC094019.2 0.163999080597015 0.500387859688581 1.60935906064093 6.06769419043849e-22 2.65098140748391e-20

C8orf34-AS1 1.7097120519204 5.18768936240138 1.60133872570025 1.99326165577934e-19 6.06403851897274e-18

PRRT2 0.607890550696517 1.4474271100346 1.25160720015741 5.76606180703871e-15 9.06332128864993e-14

ATP11A-AS1 0.0239433799253731 0.107088585380623 2.16110598349147 6.59255424274925e-08 3.04777482317769e-07

AL121758.1 0.0526346693233831 0.0176691233356401 -1.57478292330863 2.10484736879266e-09 1.26828763023748e-08

AC005070.3 0.202517876915423 0.410120582387543 1.01799888381396 2.14437193193921e-12 2.19246999396401e-11

AC007342.1 0.0227312421890547 0.0532659094809689 1.22853596943565 0.000123343179988979 0.000301603573777253

RIMS2 0.388765915447761 0.115882078048443 -1.7462442770581 4.63330692545802e-14 6.24856727866257e-13

BRCA1 2.93196895323383 1.46192964256055 -1.00399594556841 1.73808232862415e-29 1.81437220182712e-27

SLC26A9 4.2139068120398 15.3668575304498 1.86659185370691 1.88959276516724e-20 6.55842158976193e-19

SNORA80B 0.595227784179104 4.35491891615917 2.87113208208538 0.0014142048121301 0.00278402804374564

AL158212.4 0.0164751594676617 0.0518745495536332 1.65473447438527 5.48626304847251e-06 1.74801135106854e-05

AL160153.1 0.0894075573134328 0.238274382698962 1.41415516543847 1.72546233434143e-11 1.50578796567448e-10

AC012456.2 0.154215905721393 0.0751448488581315 -1.03720545487228 0.000293586153167207 0.000664700437841317

LHFPL3 0.306063283447761 0.729486581276817 1.25305145648475 2.3993486615704e-14 3.38957571766273e-13

WFDC11 0.0444737088059702 0.0933046620415225 1.06899644758393 1.86188350661508e-05 5.35519128256625e-05

MEIOSIN 0.0447646896169154 0.0161187765017301 -1.47361894312925 1.33665667722214e-05 3.96416830644389e-05

AKR1B15 2.02409213501493 0.750268489771626 -1.43179608837377 3.25413682711758e-06 1.0811545459386e-05

MAGEA3 11.2672651977264 2.50244270602768 -2.17072844183248 2.57112189443118e-10 1.82412928178191e-09

TMEM59L 5.92198762154229 13.4771465914533 1.18636169852629 9.1830734201729e-08 4.13495321109201e-07

AL357134.1 0.0665369271641791 0.178822766885813 1.42630327972593 1.81025151798726e-05 5.22426706942614e-05

FGF5 0.327700143621891 0.0180112183114187 -4.18540862901183 1.45601405710401e-10 1.08329663933065e-09

C5orf38 2.95585084709453 5.92325347304498 1.00281635192123 1.91895133667198e-15 3.22577252968523e-14

AL445183.2 0.173405862378109 0.603840166418685 1.80001405269943 6.78158979014938e-19 1.92203607005788e-17

AL033381.1 0.00849279633333333 0.0188828167750865 1.15276243143909 2.35059921426428e-06 8.01518709395913e-06

RLIMP1 0.0189193650248756 0.268783115906574 3.82850694087423 0.00037453448260136 0.000829166108074081

CDT1 8.28685517164179 3.61523181764706 -1.19673654925632 4.19739770586323e-33 7.08634736144193e-31

LINC02000 0.0751827106965174 0.0352175928373702 -1.09410462969126 0.00494611262342111 0.00866988273092145

MS4A6E 0.0734715224825871 0.183797485259516 1.32285995194165 0.000295925219821992 0.000669497457368805

ATP8A1 3.0667255181592 6.53045793910035 1.09048511574597 2.54841252315362e-29 2.61045252839893e-27

COMETT 0.208075552587065 0.0994254822560554 -1.06541990866992 0.00476344846025252 0.00838138930699347

RNU4-25P 0.0804466287562189 0.254667625017301 1.66251169771704 0.000161224217171728 0.000385040371956581

MIR421 0.32134379039801 0.853652866020761 1.40953192963895 8.69280087545896e-08 3.93102023716605e-07

ZNF540 0.313053255422886 0.685747449411765 1.13126924728319 9.42882439085793e-27 7.34696145555454e-25

GAPDHP14 0.216599458059701 0.0712646391349481 -1.60377132609182 1.72843637781918e-08 8.85257208489786e-08

S100A9 1011.16463532836 362.057549730104 -1.4817269726637 0.00152492542102464 0.0029792635377544

AC018620.1 0.0340217122885572 0.0919469389619377 1.43434579352304 2.11151271548198e-05 6.00560240936274e-05

IGF2BP3 2.86570195666667 0.927871981598616 -1.62689089620758 3.71729286214146e-26 2.74778269674511e-24

RSPH1 1.85003005472637 3.78275335404844 1.03188800347479 4.78511817783609e-07 1.87900907629314e-06

AC026362.2 0.0175443072885572 0.0373621686055363 1.09057520941261 0.000199364510643831 0.000467514307305906

MIR9-1HG 0.562916161985075 0.166083826411073 -1.76100848277907 2.41963363141773e-10 1.72335884946028e-09

AC112191.1 0.0194469940298507 0.0391346023529412 1.00889761422298 5.55674645828681e-06 1.76943783483693e-05

AC011298.1 0.759383127960199 0.172851472920415 -2.13529504809068 4.69814627811642e-06 1.51669382325878e-05

AC087501.2 0.0248333223880597 0.0557941207612457 1.16783581881207 0.000843082887805778 0.00173880680050434

LYST-AS1 0.0373563628855721 0.121635174878893 1.70313459025152 2.18147300002043e-06 7.49305335970493e-06

AC010547.4 0.0107660560099502 0.0240661156885813 1.16051347115426 1.00266548043796e-11 9.17153875919004e-11

OTOG 0.0199042715606965 0.00651748605709343 -1.61069057738242 4.24205351589074e-06 1.38037077524821e-05

ADAMTS9-AS1 0.0623070553681592 0.175749273086505 1.49605128035397 1.03505984670802e-10 7.92522027084668e-10

AC015799.1 0.0977664215273632 0.248928770574394 1.34832203012824 0.000178363707438104 0.000422477826248807

LINC01996 0.0746608625671642 0.217323933955017 1.54142298623634 1.75215755204245e-07 7.481890561805e-07

AC004805.3 0.0343954204477612 0.0792957474740484 1.20502700700496 6.8240155032963e-06 2.13787885469821e-05

ANKRD26P4 0.0314810595522388 0.0725526125017301 1.20454346614788 1.00747940637162e-06 3.7000888632018e-06

SFTPA1 428.497701953821 1561.47729011938 1.86555221492696 2.41793424458531e-18 6.32222768541188e-17

AL031772.1 0.0219842567711443 0.0625962702698962 1.50960593859853 3.09271896268898e-05 8.49170400858785e-05

OGDHL 1.08538518228856 0.472057137484429 -1.20117372008642 4.59100672816945e-06 1.48526181707398e-05

HMGB1P14 0.163434126318408 0.333488314325259 1.02892694830532 2.50893987608605e-05 7.04078653919079e-05

DUXAP10 0.129993593064677 0.0515399760657439 -1.33467674720094 1.01332302342693e-09 6.48896855320219e-09

RXRG 0.252932689522388 0.75670851132526 1.58098416658412 7.31188995702067e-11 5.7465571932332e-10

FGF12-AS2 0.206803724427861 0.0508418615916955 -2.02417340689449 8.53144499092976e-07 3.17592242414494e-06

AL139241.1 0.0924223191044776 0.223306120041523 1.27270959508243 2.9938738123755e-12 2.99496886497695e-11

AC007496.3 0.0128979826368159 0.109505342754325 3.08578392286039 1.74076782229182e-06 6.10932887715659e-06

CYP4F24P 0.0289038060995025 0.129654441754325 2.16534024472563 2.27159840074662e-13 2.75880178776288e-12

FAM90A20P 0.0148200335422886 0.0332203306643599 1.16451772057916 7.30676188824957e-10 4.78658533278146e-09

HPSE2 0.110397229353234 0.344312534231834 1.64101473606934 4.80162459743003e-21 1.82903109665336e-19

AC007610.4 0.0680346992686567 0.137085244217993 1.01073064157036 2.39070184989472e-08 1.19208196161569e-07

AC024257.1 0.0633473574626866 0.15147581799308 1.25773115270648 2.95493538805035e-09 1.7339086647324e-08

AL451064.2 0.0725438630149254 0.159531507453287 1.1369159071701 7.87026336308639e-10 5.13237250787823e-09

C7 8.72147894766169 24.6980104599654 1.50175012432415 1.18168999777284e-20 4.2358088386746e-19

AL139246.4 0.0644798807462687 0.178696118304498 1.47058731526948 8.79645938450402e-08 3.97539487450106e-07

XRCC2 2.20521542338308 1.0711005633564 -1.04182565883602 2.5081204418675e-27 2.08501805729715e-25

AKAP14 0.372779121233831 0.851957459979239 1.19246033626541 0.000848093569399222 0.0017478469469293

RN7SL559P 0.0982062975124378 0.32847268349481 1.74188595193592 0.00340419859692777 0.00617487940217366

AL022314.1 0.0651683947761194 0.135960568062284 1.06094393065729 2.54656263561658e-05 7.13465356321589e-05

BTBD9-AS1 0.0584315567164179 0.362702700934256 2.63396785788338 1.14444617902055e-05 3.4369828699036e-05

AC097493.1 0.0351284852238806 0.108012881730104 1.62049010744814 1.18997675775091e-06 4.31242405253576e-06

PRSS3 3.69254278820895 1.69979045853633 -1.11925772938829 0.00101851210374495 0.002066491545803

AC046195.1 0.0975478424577114 0.35316302015917 1.85615241664132 1.96864263505229e-12 2.03025550786878e-11

RPL10P19 0.436492890049751 1.23659704359862 1.50234540221206 1.09882157527269e-28 1.05448316083186e-26

ZRANB2-AS1 0.0582446109950249 0.128035026608997 1.1363420654368 3.98129866208647e-11 3.29067749797718e-10

C2orf73 0.09542646560199 0.201276295141869 1.07671592786082 2.0903170887313e-05 5.95026773280611e-05

AL139023.1 0.822195655074627 0.25304108083045 -1.70011012468702 5.20740934234389e-06 1.66654160441265e-05

ALOXE3P1 0.116085547870647 0.317438590273356 1.45128914959583 2.08119805387759e-06 7.17790249351224e-06

MIR30C2 0.221584217363184 0.519101277543253 1.22816091117106 7.69875997685317e-08 3.51638419116457e-07

AP001025.1 0.259990147711443 0.0719870397231834 -1.85264785593429 8.95626384580218e-05 0.000224996616315504

ROS1 5.49527532288557 14.2104975040138 1.37069339402181 1.31785300804011e-19 4.10048689077327e-18

KRT81 21.7204732985075 8.32364805221453 -1.38376767025494 0.00012048813317468 0.000295070777429038

RNU6-247P 0.483339199004975 1.46057956730104 1.59543304554638 0.000346965814173448 0.000774148720643156

AC106738.2 0.139028974975124 0.432748259861592 1.63814243253227 4.41836925941661e-16 8.16502697601651e-15

AL031058.1 2.91087894089552 1.3668460683391 -1.09060406271686 3.36891197169831e-15 5.44883160413653e-14

DMRT1 0.114787692910448 0.0262195823425606 -2.13025136030768 0.00164184597321311 0.00318742812090989

CHRM1 0.0438675453880597 0.1148969286609 1.38911434583629 1.18097221607453e-05 3.53851775960105e-05

LPL 7.42666904378109 17.5990525128028 1.24471056627396 1.72702537178614e-16 3.44272185993811e-15

AC089998.3 0.0353398652736318 0.0916563548788927 1.37493837178624 5.87750263789343e-07 2.26605033467663e-06

ACBD7 1.06150187761194 0.46951296366782 -1.17687002549524 4.19187622173825e-07 1.66397408801947e-06

AL357078.2 0.0544822416915423 0.171145424013841 1.65136474903479 1.3572789213319e-06 4.86776534204399e-06

AL138899.1 0.0222080841492537 0.0555255235397924 1.32206615114445 2.33760424171855e-07 9.74150175392387e-07

PLA2G4F 1.2291426001393 3.49201567986159 1.50640773592865 2.07054797952866e-18 5.47673957834708e-17

SH2D5 0.27146294780597 0.054250429716263 -2.32304882660591 1.03292565824636e-11 9.41683891767932e-11

COLEC12 3.78757187129353 8.00734892560554 1.08005140918562 2.38910934491952e-16 4.6243553137685e-15

CLMAT3 0.0247632581890547 0.0558680757370242 1.17382298292601 1.05668205180003e-05 3.19763820720633e-05

SP9 0.234874473766169 0.031085634716263 -2.91756998761596 4.98820265984712e-13 5.68210507067134e-12

TTC29 0.208964715004975 0.52333659899654 1.32447980102797 1.08189392244102e-05 3.26670333172465e-05

FAM111B 4.96028678109453 2.39272758273356 -1.05176738081064 3.06776319072966e-21 1.20205334192631e-19

LINC02471 0.464522250845771 1.63970419761246 1.81961596716811 5.40245052187212e-10 3.62950188585612e-09

CFAP52 0.422494352462687 1.26150572 1.57814278594637 2.29695448314536e-07 9.58966304697627e-07

NPIPB13 0.0424569002537313 0.0902765533356401 1.08835229398264 0.00020993845679038 0.000490334482768309

AP000915.1 0.0152329135820896 0.0426197844290657 1.48433138512435 6.82221199192097e-09 3.76412140365218e-08

ARHGAP11A 4.63384789701493 1.90756330103806 -1.28047975687929 1.688082699652e-33 2.97864915067627e-31

AL355336.1 0.0244452570845771 0.0902586992595156 1.88451140978026 0.00264184436037355 0.00491794468120178

RNASE10 0.179407598626866 0.0875091260207612 -1.03573561191187 0.00517731912067227 0.00903206347436972

AL592295.2 0.0684790859701492 0.137054113079585 1.00101027449563 0.000239423753636836 0.00055296737560948

OTC 0.0300774918308458 0.0699260543529412 1.2171458366332 7.78134654239181e-12 7.26595520431601e-11

AC007347.1 0.108198105572139 0.292800621591696 1.43624337693974 1.2870822832346e-06 4.63424176493764e-06

CASR 0.110176224089552 0.598891407570934 2.44248150833471 3.80308813474199e-12 3.7227795449246e-11

LINC00659 0.511043872039801 0.134419691695502 -1.92670264885891 0.000121821079697453 0.00029817491764143

HP 9.03406498417911 31.07561317509 1.78233566182944 2.88795648809391e-06 9.69029688987467e-06

AHSG 0.134920421447761 0.0299910867197232 -2.16950302601857 1.39679067880008e-10 1.04292178720126e-09

AC004022.1 0.0234668720597015 0.0807917437058824 1.7835823128471 5.91650462108167e-08 2.75760738559277e-07

OR13K1P 0.0271622984179104 0.0874339260346021 1.68658761897271 4.92112813335827e-05 0.000130053970864189

MIRLET7D 1.7964986641791 3.59839336262976 1.0021650425292 2.60741276285126e-17 5.86453446249852e-16

RAX 0.0970420877960199 0.0402468155259516 -1.26973595293951 1.48982930269153e-05 4.37526376340742e-05

AC114684.1 0.0368953469154229 0.0763557111072664 1.04929718779824 6.48070605578103e-08 2.99860109331097e-07

UGT1A7 0.119944885089552 0.0166771652560554 -2.84642564794316 0.000860790170344638 0.00177211977108964

AQP4 8.58616494358209 19.182822592872 1.15972922365715 1.30131086471717e-12 1.38115954271822e-11

RPL12P7 0.0229078639303483 0.0558099008650519 1.28467814455069 2.05981980124846e-05 5.87201079467848e-05

KNL1 1.64258217402985 0.76741958916955 -1.09787804977055 1.51084992341652e-29 1.59543418553829e-27

LYPD8 0.433713733492537 0.185289146927336 -1.2269647468473 3.26162454475468e-05 8.91074131445815e-05

NTRK3 0.036998204920398 0.0871164896366782 1.2354905460225 2.0100281559993e-21 8.04894144459456e-20

RNU5F-1 0.541789660348259 1.86724712657439 1.78510811196482 0.00101282726465666 0.00205587247204688

LINC00592 0.41238923318408 0.159053751211073 -1.37449226917216 6.22528986190389e-08 2.88970939787969e-07

AC145146.1 0.0990716476119403 0.271060380622837 1.45207010774085 6.1766009144806e-10 4.10323135805306e-09

SLC26A5 0.16805243558209 0.405733408539792 1.27162064878762 2.61044789501367e-17 5.86653655945964e-16

AC005274.1 0.0946054888557214 0.250342152560554 1.40390543826447 2.09364089385354e-21 8.35928152509406e-20

PTTG4P 0.0849284658706468 0.195988348719723 1.20644779555785 0.00293075117870344 0.00539698658345941

DENND6A-AS1 0.104628478208955 0.235356757681661 1.16957369331083 0.00333786154830896 0.00606904090853943

MIR3186 0.452910123880597 0.924646603806228 1.02967729051111 1.10121948715957e-09 7.01078979837389e-09

MIR1-1HG-AS1 0.0217468941243781 0.0446926821522491 1.03922925710571 1.21372502418243e-12 1.29618818474773e-11

TSPAN19 0.198967108721393 0.620721781442907 1.64141681333524 0.00181644825899899 0.00349145125332928

RAET1L 0.341885429393035 0.101604892705882 -1.75054306308101 4.42897403874426e-09 2.51520847092308e-08

AP003119.3 1.19831922447761 0.450850315051903 -1.41029184906271 5.27051702929651e-09 2.96114709842357e-08

FNDC5 0.705646945870647 1.45302825442907 1.04204430671696 8.80799640973835e-10 5.68693819184003e-09

AC130456.7 0.0760363921243781 0.235232472124567 1.62932524361931 1.42634339587924e-08 7.40377526614106e-08

LINC01305 0.107849438542289 0.0456932990138408 -1.23896415326979 0.000138932766305251 0.000336117749531058

AC007336.3 0.0117592358507463 0.040685718249135 1.79072814696309 0.00160613666885004 0.00312275807869827

GGH 17.1672529472637 5.34347738754325 -1.68380838329072 5.18782204616657e-29 5.14083090444405e-27

AL050343.3 0.0825367226368159 0.191016985570934 1.21059287283283 1.12608992654942e-05 3.38669006940033e-05

AL353770.4 0.0300887095870647 0.0766958090138408 1.34992550966085 6.94771713550193e-06 2.17190608819268e-05

PLA2G1B 2.590194739801 10.6025437278201 2.03327795911349 8.12827974338845e-20 2.60009884189093e-18

AP000251.1 0.750603679751244 0.303388666678201 -1.30688416930878 4.27824794426479e-10 2.92379013682264e-09

SFTPD-AS1 0.0245473924477612 0.0825012358373702 1.74884594847107 1.99348659309372e-21 8.00614659634556e-20

PRR11 7.48375339900497 2.71645473183391 -1.46203701485702 8.35163321703225e-43 5.43850401156743e-40

CDK1 15.2118342189055 5.66451679965398 -1.42516932126244 4.13412103411409e-48 1.25631344758912e-44

SCTR 3.14876608850746 9.13170713177855 1.53609800242679 1.46659244170847e-19 4.52723513326486e-18

RPL18AP2 0.0575187508955224 0.115340760726644 1.00379819181216 3.22929964458362e-07 1.31060016737442e-06

AC011379.2 0.204945770731343 0.501455837429066 1.29088043136386 4.48880990605608e-20 1.47558835253166e-18

NALT1 0.323496777412935 0.66754748899654 1.04511913250598 1.96395567498926e-10 1.42667165234944e-09

IL12RB2 0.55571410660199 0.22514455999654 -1.30349124373107 3.59965064194747e-11 3.00336928179571e-10

CMTM5 0.0166188289850746 0.0345511260034602 1.05591399920801 1.28154114745135e-08 6.71587476198399e-08

AL135960.1 0.0219020636616915 0.0672325715951557 1.6180935224201 2.04636720930937e-22 9.48612596179852e-21

RFC4 10.3053519004975 4.51530504152249 -1.19049840933768 2.86328279803692e-41 1.39840686654125e-38

SFTA1P 13.1054486359701 35.163470916263 1.42391074109063 5.11291781376069e-23 2.56114106605046e-21

SLC9C2 0.0388885413631841 0.0877734607889273 1.17443966898938 6.37267253221471e-11 5.07697622359663e-10

AC007114.2 0.0426352823731343 0.10116821283737 1.24663634815531 2.76797916725753e-15 4.53318743859242e-14

AC093416.2 0.133000192288557 0.310714394290657 1.22416074638551 6.86920049535229e-06 2.15047050199518e-05

AP005271.1 0.264018400248756 0.0906488547058824 -1.5422777813043 1.75370945620092e-10 1.28520776064028e-09

AC090004.1 0.237016875621891 0.512934664740484 1.11378529088163 4.90609618946341e-24 2.8189439239879e-22

SPATA3-AS1 0.0452088786517413 0.0174252839965398 -1.37542396463239 7.84181005445059e-10 5.11503708536188e-09

AQP5 24.3518209367463 57.2599680009343 1.23349720850332 8.13603492317953e-11 6.35047945702347e-10

TMPOP2 0.19788930681592 0.0894863384948097 -1.14495430266941 1.91568893374305e-13 2.35161994335155e-12

AL109936.2 0.690667430945274 1.43459366536332 1.05457906672669 6.49263917800338e-23 3.23449328813101e-21

AC105118.1 0.459991065621891 1.46772423532872 1.67390318642587 1.31240790445392e-06 4.71797531372433e-06

MAS1L 0.0138912559850746 0.055962239716263 2.01027665585029 1.00405980574017e-11 9.18122222901827e-11

AC023796.1 0.131689784875622 0.282963705778547 1.10347357759468 5.8420868255915e-07 2.25392967527052e-06

RN7SL8P 0.630034010348259 2.5210005532872 2.00049481977379 1.97298783714782e-16 3.89611677588396e-15

CACNB1 1.78706628109453 3.80132666885813 1.08890986526104 1.12528197618409e-20 4.05486983512976e-19

SUMO2P15 0.0390237655223881 0.0803841741176471 1.04255849960519 2.43849995935687e-05 6.85577445398956e-05

ALOX15B 11.7217072660199 31.8708876629758 1.4430564878741 3.18407603219144e-21 1.24228929358682e-19

AC020911.2 0.0238403684577114 0.0673642503460208 1.49857663497495 5.20899910979655e-05 0.000136920831958612

MST1P2 1.17213795427861 2.4431128632526 1.0595781354667 6.94916117144073e-13 7.72599829426439e-12

STEAP1 22.6309382676617 10.8927316799308 -1.05493060069659 4.18772307120061e-15 6.67838052462605e-14

VSIG2 9.80535843447761 25.4792203740484 1.37767885981147 1.77040680445437e-23 9.34761121656892e-22

ADH1B 3.01679402669154 10.6620128870138 1.82139172682055 2.53733921694795e-22 1.16828665965533e-20

RNU6-1209P 0.266137583482587 0.615554119723183 1.20971344490505 3.02228647433283e-06 1.01026075620879e-05

CAGE1 0.0760036602288557 0.0265057303598616 -1.51976460406459 1.47422560041839e-06 5.25069282086769e-06

CCL8 5.75898117238806 2.70709219965398 -1.08906958099432 9.29873653744077e-09 5.00138533528034e-08

EFHB 0.285058696686567 0.588358599515571 1.04543671691002 1.62887538301777e-06 5.74909558982269e-06

CROCC2 0.159122928318408 0.359804534442907 1.17707163703685 2.38562170527107e-11 2.03768749653853e-10

AL354950.1 0.0341249172636816 0.0705981961591696 1.04880577374263 4.10613917008373e-05 0.000110230571556527

AL031848.1 0.10615691278607 0.213921793460208 1.01088514520809 2.54436766586357e-05 7.12923426507209e-05

SCGB3A2 87.1664225462687 559.509178419377 2.68231739265041 1.41061842200667e-20 5.0039447265736e-19

GLDCP1 0.0346542178159204 0.011036268567474 -1.65077848538976 0.00146966332500725 0.00288365678592067

EDN3 0.0345044240447761 0.134865127031142 1.96666409278107 2.0473541031883e-09 1.23691483813121e-08

VGF 4.02423278671144 1.72160170239446 -1.22496235242488 9.8559348791372e-05 0.000245188119833002

AP001527.1 0.0136544656119403 0.0285133922283737 1.06226683324802 1.90324338029838e-05 5.46208881963911e-05

SHISA3 7.73749180840796 18.7739358012803 1.27879324895361 4.20613192799607e-08 2.01114874529183e-07

ERI3-IT1 0.0867647493034826 0.189149280968858 1.12434436204031 1.15659760843209e-06 4.20371356685949e-06

KLK8 3.08927817661194 0.985724235449827 -1.64801378325545 0.000714125956311347 0.0014950508959825

MTND6P21 0.0342894173631841 0.0937692216262976 1.45135106633749 7.15233587279518e-10 4.69329141365039e-09

CFAP47 0.0852998182636816 0.184405567525952 1.11226764099403 0.00108959080726791 0.0021957197597095

SBSN 2.11712567847761 0.357484965231834 -2.56615244152244 1.3945840171121e-07 6.06871485569068e-07

FCGBP 3.45076353208955 9.34085473352941 1.43663895490265 1.06599517916256e-14 1.59403871788387e-13

ARL4AP4 0.160609726268657 0.371202384013841 1.20864671326447 9.07580554599824e-09 4.89109914646408e-08

MAB21L1 0.0134899979552239 0.0434754442802768 1.68831064105765 4.12330609310108e-13 4.76436086380712e-12

GINS2 6.47906144378109 2.46763931176471 -1.39265330455675 3.00408216147038e-37 8.13481654615989e-35

RNU6-638P 0.385853689552239 1.06950706584775 1.47082020785409 0.00395779905056461 0.0070836858865874

E2F7 1.30710109139303 0.427870290380623 -1.61112531117902 1.03791993471861e-25 7.22318326070076e-24

AC244034.3 0.00397342585572139 0.0200747054878893 2.33692349335092 3.6158664616544e-05 9.79730015120347e-05

LNCAROD 0.739675249422886 0.0847492423598616 -3.12561962594881 6.80983408784761e-08 3.14344240173219e-07

EXO1 4.16102259800995 1.42598196920415 -1.54498238373281 2.52314352709464e-42 1.50017337969649e-39

KCNJ5 0.891896708855721 1.82452199795848 1.03256999989058 1.68894513892859e-09 1.0361742833041e-08

FAT4 0.641398511393035 1.33202423249135 1.05432741790953 4.02750112563115e-20 1.34168277449466e-18

RPS27AP10 0.12741400960199 0.42146635467128 1.72589355102696 0.00232042175060413 0.00436926229803945

TCAM1P 0.723057283303483 0.269513203989619 -1.42375399218557 0.000136239364395789 0.000330156531652032

AC011444.4 0.0698787881094527 0.154183204221453 1.14171912212667 1.75295365691541e-05 5.0696079641151e-05

AC137932.1 1.38859513338308 3.66123328245675 1.39870368029648 5.93315679851992e-27 4.70353154897159e-25

PSAT1 21.2850597159204 9.33450995467128 -1.18919494704872 1.10482261801123e-20 3.98639823253392e-19

AC009994.1 0.142955845621891 0.336229107370242 1.23387500959706 0.00560296037924445 0.00970002319105809

BX842568.2 0.0604329079104478 0.138589960034602 1.19741647872119 1.48180525202949e-10 1.10039026997031e-09

LCN1P1 0.118560830099502 0.249926536920415 1.07587663953438 7.43460328764352e-11 5.83964388044372e-10

PRDM13 0.116000674985075 0.0311600666401384 -1.89636297621588 1.34540980652449e-08 7.01830215686531e-08

AC069277.1 0.175380290039801 0.0602360175986159 -1.54178832391719 3.21350176281085e-06 1.06869252447564e-05

DNAI2 0.323280257900498 1.16267954483737 1.84659620651103 1.6793561104885e-09 1.0309851766972e-08

RNA5SP217 0.327686998507463 0.782073143598616 1.2549851094385 5.93600945006254e-06 1.87861442327251e-05

PLA2G10 2.07268146791045 7.49225203844291 1.85390101404534 1.44013139047689e-18 3.88437806011272e-17

AC103740.2 0.192676806930348 0.0541353948442907 -1.83153884906058 2.14438625604462e-16 4.19545604588121e-15

FOSB 7.93008245059702 26.4015084957785 1.73521259171265 1.11287879644895e-08 5.90441029735767e-08

OVCH1 0.0464045604477612 0.100390290695502 1.11328124543939 2.1656842997302e-05 6.14816956587305e-05

AC013394.1 0.125580760572139 0.267675653529412 1.09187046940108 3.84962216190635e-07 1.53973626978852e-06

CCNE1 6.29181799253731 2.37408488235294 -1.4061054199821 2.54964256538968e-35 5.49215313963768e-33

AC006237.1 0.328370973034826 0.720201068858132 1.13307313761837 2.00178217889195e-10 1.4522212889309e-09

PLOD2 25.276244021393 11.1752760934256 -1.17747162433322 2.69773720957363e-19 8.05492496526624e-18

IRX3 11.7853562960199 25.064799449827 1.08866731583505 8.49909264104738e-19 2.36470176737178e-17

RSPH10B2 0.0154974600298507 0.0408051231764706 1.39671851415518 4.17129990453982e-07 1.65724945364852e-06

APCDD1L-DT 0.253324112910448 0.0651009649238754 -1.96023357573514 3.25341998114423e-05 8.89010255612895e-05

AC092338.1 0.175945553333333 0.400385313529412 1.18626000251498 0.000416098686736901 0.000912980271340092

WWC3-AS1 0.0275221697512438 0.079381488650519 1.52820840608765 8.14393165163989e-07 3.04368038633986e-06

AGAP11 0.0196664867263682 0.0458417457439446 1.22092273206394 7.73893519690347e-12 7.22882095749009e-11

AC108215.1 0.305448064348259 1.18944367612457 1.96128795418937 2.48853936333217e-31 3.28799766121424e-29

TAF7L 0.575196293975124 0.179605119176471 -1.67922590950246 0.000416997402445394 0.000914658670052252

AL031681.1 0.0815390744776119 0.178262223875433 1.12843752268881 6.99329850262828e-06 2.18565551419133e-05

TPSD1 0.936834984363184 2.31065887612457 1.30243743291604 1.35510969579372e-07 5.91481809447147e-07

AP002373.2 0.0153185283233831 0.0390196435363322 1.34892289648051 4.18998865187698e-05 0.000112250161258532

AC098476.1 0.141690624278607 0.408674116712803 1.52820657572626 1.62871106347003e-10 1.20035698156576e-09

AC005920.2 0.0466881282587065 0.273968066678201 2.55288008776868 0.000422936926349624 0.000926646233730851

FBXO5 3.7488528 1.84886605397924 -1.01980847017199 1.32013107388407e-38 4.35007046635293e-36

SYNDIG1L 0.182014292945274 0.497788097564014 1.45147999029438 7.96458042890799e-15 1.21871484145623e-13

LINC01910 0.0363484809651741 0.0117033335467128 -1.63497555211522 2.27933199380778e-05 6.44205125872097e-05

WFDC6 0.10851474800995 0.384020548477509 1.8232923802603 4.48919097552599e-05 0.00011953984342385

AC009119.2 0.0585423398507463 0.122256453806228 1.0623583113388 6.73889423673469e-10 4.44331623371972e-09

AC020928.1 0.501729129850746 0.16364582250519 -1.6163319276227 2.1705965849508e-10 1.56349266785368e-09

AC093648.1 0.0675388511442786 0.254765264809689 1.91537904716249 1.27539458188426e-18 3.4743069536389e-17

LINC02082 0.033428626119403 0.0760261874740484 1.18541238232643 3.20868010886461e-06 1.06731025058706e-05

MACC1-AS1 0.0447324685074627 0.314404015986159 2.81322536669142 0.00310190262112354 0.00568003727154049

CFAP77 0.349568240751244 1.02309656923183 1.54929630325158 1.80907852597865e-07 7.71170475148318e-07

AC009041.1 0.0275301344228856 0.0720364335605536 1.38771510259439 8.72621823159185e-11 6.77055513855424e-10

CACNA2D2 3.4833232079602 15.1768098650519 2.12333232307593 1.65784676138601e-30 1.95440124672015e-28

ESPL1 2.95355226129353 1.15387854737024 -1.35595976011071 2.5714840399178e-32 3.86429057646988e-30

AC015914.1 0.122996968 0.346096880276817 1.49255318497729 4.51097948213356e-20 1.48093908995013e-18

RPL12P29 0.0346997875124378 0.0912783223875433 1.39534544810339 0.00155896059939519 0.00304054570302064

C17orf64 0.272685304477612 0.124544759550173 -1.13057263399858 2.55253632820498e-06 8.64436213179869e-06

AC087284.1 0.151544963681592 0.332471324256055 1.13348400553208 0.00118801185535171 0.0023735937061779

AL357093.1 0.0604266243283582 0.24416817432526 2.01461891229453 2.5162132466807e-07 1.04254555819902e-06

CDC25A 2.18531760696517 0.925862648096886 -1.23897288119378 1.0788137389269e-34 2.16952615879785e-32

RPL13AP17 0.223736898333333 0.761093930802768 1.76627131268519 1.12429551855671e-12 1.2068085727051e-11

SHANK2-AS1 0.0168469169552239 0.0449796223564014 1.41678695114973 0.00103836710422801 0.00210303171657554

SOHLH1 0.312226134378109 0.0818323948719723 -1.93184732184424 2.02414928619221e-10 1.46766921997235e-09

AC008691.1 0.156043078691542 0.373651783283737 1.25975003992253 1.1599817882132e-09 7.35237587662364e-09

AL391903.1 0.354689374477612 0.812422889446367 1.19567477540217 1.83280017725894e-08 9.34683663025023e-08

SNRPGP4 0.256888938905473 0.604129708685121 1.23371356211829 8.88237929012258e-05 0.000223243037662978

SELENBP1 29.7448949353234 81.3026748096886 1.45066073446944 4.92751407050226e-31 6.29754718823536e-29

CASC15 1.21951352109453 0.57076081750865 -1.09534755087984 3.22276122045371e-05 8.81303917675292e-05

FKBP9P1 1.35585668114428 0.566534274463668 -1.25896954389658 0.000673452959941191 0.00141695041575441

WDR72 2.71453176826368 1.25781343313841 -1.10978541946617 0.000225876709957817 0.000524658671761483

MTCYBP21 0.034595662039801 0.104264801820069 1.59158915421556 4.93830803163998e-16 9.06461239364788e-15

AL356423.1 0.0313499144278607 0.0719716606574394 1.19896744206868 5.46952225516523e-05 0.000143122305471459

ATP5MC2P3 0.0455637224378109 0.150690286955017 1.72562890671505 8.59270820322182e-05 0.000216459951513417

AL137025.1 0.00712033154726368 0.018770389 1.39844222434032 0.00299857592053968 0.00550742404316434

KCNA2 0.0529040972089552 0.117995777114187 1.15728386600265 3.6800026770232e-07 1.47816233245094e-06

MTND5P1 0.0449956585422886 0.12723110784083 1.49959373763271 1.93816823077916e-18 5.1315489943669e-17

PHACTR2P1 0.0472406973134328 0.095203119183391 1.01097858167062 1.66494764581768e-06 5.86354855950471e-06

PRICKLE2-AS1 0.00441392709701493 0.0123812851574394 1.48802636520683 6.48655181445053e-12 6.12593895460021e-11

OR52V1P 0.0124420581094527 0.0333253466782007 1.42139473416395 2.47907259918426e-09 1.4752531677043e-08

AC012213.1 0.441118003661692 0.145598502802768 -1.59916912306464 2.26691066533803e-13 2.75433170577499e-12

RNU6-553P 0.128707403980099 0.259844943287197 1.01355593576089 1.56531410035352e-05 4.57502572546711e-05

RHOV 30.6982497353234 13.0526711975779 -1.23381132143694 2.6622630954363e-05 7.42155699318956e-05

WARS1P1 0.0903106302487562 0.343339429446367 1.92666782835885 0.000207910161598557 0.000486053758417004

RN7SKP271 0.114568880845771 0.258318768408304 1.17293723466797 2.81488645059917e-05 7.80802681783847e-05

AC092675.1 0.132608582089552 0.287611947404844 1.11694946057779 8.16451735369107e-05 0.000206701425181385

C9orf135 1.3822824029801 2.95747159801384 1.09731192251663 1.71581013565658e-09 1.05100576058695e-08

CCDC141 0.0495564910995025 0.131613291882353 1.40915925237144 1.15695604152084e-10 8.78233353749514e-10

KRT78 0.40614080380597 0.0851441340449827 -2.25400093317771 4.12124020812757e-06 1.34409634739195e-05

SNORA63 0.0918917317768301 0.188054062995551 1.03314051498665 3.83063635482456e-08 1.84418067777595e-07

MEIOC 0.407163962338308 0.101418168456747 -2.00529374995679 1.05554511616684e-07 4.69952123183511e-07

AC104964.2 0.0103340411293532 0.0380523641557093 1.88058155893294 3.7715429598853e-11 3.1334052233555e-10

FETUB 0.342691311681592 0.0329171359446367 -3.3799988951272 0.00450156051628128 0.00796414257845223

AL133406.1 0.0199209644776119 0.0488444528027682 1.29390722913254 0.000182657842605728 0.000431854425593591

AC018557.2 0.0682100498507463 0.149021896643599 1.12746810763624 0.00295130913849993 0.0054315527177157

PABPC4-AS1 0.32684439920398 0.682321334602076 1.06184735131751 9.03661250507708e-07 3.35029621816264e-06

AQP2 0.0438082448159204 0.248253878349481 2.50254193731971 5.11100629884434e-12 4.90133317929147e-11

PIMREGP2 0.0156564748756219 0.0326362820069204 1.05971729181315 1.58586494419879e-06 5.61540733089551e-06

AC087392.2 0.0738789435820895 0.164186798892734 1.15210299276686 3.88143393656339e-06 1.27256315230171e-05

AC053527.1 0.174370341940299 0.38320756550173 1.13597136598415 0.00179225050887653 0.00345003177208425

CLEC4F 0.131635773402985 0.439404763681661 1.73899890053758 1.53280108066105e-22 7.27814402015273e-21

G6PD 76.9377299253731 36.1227911730104 -1.09078189026958 3.27200072811855e-10 2.27881894357123e-09

C17orf102 0.00300485733333333 0.00653973422145329 1.12193550965847 3.30470891402899e-07 1.33842423809704e-06

MIRLET7A1HG 0.142977348641791 0.29829726816263 1.06096416097069 1.25906020176839e-11 1.12533648752828e-10

AC007673.1 0.0245386821243781 0.0557779713079585 1.18463769364701 8.33565372433103e-06 2.57401071875865e-05

CIBAR2 1.05757981666667 2.5512956899654 1.27046356486856 0.00018830120931327 0.000443933888496088

DUXAP9 0.181228093557214 0.0794592044982699 -1.1895203608034 3.98639403162358e-12 3.88414238563965e-11

CDC42-IT1 0.0765475742288557 0.218882369377163 1.51572718747227 7.44571274822972e-08 3.40934611860175e-07

UBE2S 11.6530770920398 4.96403004948097 -1.23112720694248 4.09996550809116e-33 6.9648482389002e-31

ARMH2 0.0385067081094527 0.0909919749134948 1.24062951744843 0.000464418411345507 0.00100872328067818

MT1G 10.0447851906468 4.91227396356401 -1.03198378319236 9.51494616143082e-09 5.10763056948249e-08

AC016831.4 0.074070453199005 0.490230995228374 2.72649163313178 0.000569987981705217 0.00121552992589768

AC114760.2 0.155495143880597 0.532752840515571 1.77659685450945 9.02153699528434e-11 6.98593894643133e-10

AP005264.4 0.170774171726368 0.0692241932802768 -1.30274155395075 2.78364044848921e-05 7.73233457913669e-05

AL356218.1 0.0534886399004975 0.157139450207612 1.55474099322168 2.71493890804097e-07 1.11962574087637e-06

DDX3P1 0.0416352580945274 0.120760659567474 1.53627286937708 1.29138323602776e-05 3.84239898883369e-05

FCF1P8 0.0478198300497512 0.113151837404844 1.2425791039786 0.0010344246407535 0.00209613350556481

PTCSC3 1.2609994321393 3.74469006065744 1.57027868910744 6.37988317900541e-24 3.62764667246981e-22

PRAME 13.066539454403 3.66618939287543 -1.8335238864324 1.28224724362416e-12 1.36297948360361e-11

AL354861.3 0.0056287685721393 0.0255412210726644 2.18193626107474 7.25649740632686e-13 8.05132673683731e-12

CLGN 4.43605448666667 1.05295152380623 -2.07483806724502 1.88252130933845e-12 1.94804986040131e-11

MPRIP-AS1 0.0454373493034826 0.107885947799308 1.24755638594283 0.00267171605703239 0.00496712896199007

RGS6 0.0744635678358209 0.185941507916955 1.32024221250511 6.17269849821466e-05 0.000159673984608125

CIT 6.86356338159204 14.9180942515571 1.12003356142445 2.44958310496901e-05 6.88339647805429e-05

AL731684.1 0.37828666641791 0.152688118027682 -1.30889212815901 0.00121092496170374 0.00241390653809018

MAGEA12 5.73366167482587 1.21607407840484 -2.23722566352284 3.98498284071387e-07 1.59015583153668e-06

TUBAP9 0.012627165238806 0.0397755157231834 1.65534984172651 2.30805854738073e-07 9.62864570940558e-07

SNX2P1 0.0653655591044776 0.232766412318339 1.83228030573006 4.53634265413209e-07 1.78825268939914e-06

LIN28B 0.364466370427861 0.0461578559204152 -2.98113758619892 2.17418787251042e-14 3.08583488911053e-13

NXF5 0.00472545210447761 0.0135351702249135 1.5181887617694 6.49544334925271e-10 4.2952218472452e-09

ABCC6 1.73402385273632 3.8367630799308 1.14576593729481 1.36252135251525e-29 1.44999840433043e-27

ITGB1-DT 1.14084287233831 0.18509307283737 -2.62377729566823 1.72592180300903e-16 3.44272185993811e-15

RN7SL663P 0.312914274527363 0.846078760553633 1.43502449555142 1.82857149492027e-05 5.27213054881608e-05

ECT2L 0.231885648835821 0.6228736863391 1.42552609038587 0.000214090817904834 0.000499350492043085

AF131215.4 0.0272953452537313 0.0907844619965398 1.73379045194962 6.47206010344586e-08 2.99612125641916e-07

ITGB2-AS1 1.58228790810945 3.23927582415225 1.03365918683576 4.30238673584307e-09 2.44789426306029e-08

SHOX2 1.04099935165672 0.258450159775087 -2.01001117083713 1.49829205308671e-14 2.19369848243691e-13

AC073149.1 0.21114312800995 0.674810209896194 1.67626050871355 6.13516790086526e-17 1.31091282881769e-15

AC063979.1 0.0172424904975124 0.0365032161314879 1.08205540716124 5.88326012343899e-05 0.000152982662460597

AL109659.2 0.100855084238806 0.274720713989619 1.44568188001393 1.6510023878862e-25 1.12605773837126e-23

C1orf158 0.12500472038806 0.339802695591696 1.44271472269717 5.14592249176286e-07 2.0097241203729e-06

AKR1C7P 0.753002646268657 0.228627114636678 -1.71965842102381 3.17765265138239e-08 1.55166577424225e-07

AC002044.3 0.0278273304975124 0.143429003183391 2.36576236367222 1.8777457683305e-05 5.39514095638609e-05

AC023509.2 0.039913900681592 0.148450104695502 1.89501492911427 3.22090812030333e-29 3.26266063297393e-27

PNPLA1 0.165593312855721 0.0500784753252595 -1.72538186957412 6.13254704423653e-05 0.000158740452072562

CCDC13 0.145695332736318 0.309783100761246 1.08830378234059 8.14324630097282e-12 7.58059177439097e-11

AC092127.2 0.0420366963681592 0.121088303598616 1.52633831753681 2.32560078591963e-06 7.94468916998525e-06

NCCRP1 7.65559891834328 2.30304038806228 -1.73297553642862 0.000695342862303361 0.0014597544747921

PLA2G2A 2.0205914978607 13.9450579557439 2.78690434297218 1.09041938414453e-05 3.28990294057947e-05

RN7SL473P 0.298097526069652 0.760353282595156 1.35088549070039 1.23496200461753e-07 5.42415462121237e-07

AL118508.3 0.0514331580049751 0.116100324965398 1.17460136706725 7.8371465501599e-18 1.88851064446584e-16

AL589935.1 0.0296604779004975 0.0646595512802768 1.12432165237561 1.43288018394606e-11 1.27155331054266e-10

ATP13A4 3.54950396054726 8.99410638685121 1.34136252545026 2.77842427945223e-19 8.28679433402601e-18

GOLGA2P11 0.0320434288308458 0.073663525200692 1.2009219070425 5.84972400277966e-20 1.90237754430468e-18

AC007906.2 3.50162804830846 9.15371886636678 1.38633213860734 2.83364644318182e-06 9.52325266908611e-06

AP005262.1 0.716734612487562 0.207108375640138 -1.79105312814358 5.96150707965203e-09 3.32139373861241e-08

AL391807.1 0.0526946162686567 0.115013590685121 1.12607687213244 5.84754950281387e-17 1.2553412786653e-15

CECR3 0.0120735953880597 0.0289334574463668 1.26088337126564 0.000170073837152717 0.000404691095017123

RNU6-767P 0.16732188358209 0.555322297231834 1.73069918005861 1.28040460844987e-06 4.61080527203475e-06

AC009336.2 0.0122075851691542 0.0322907663737024 1.40334383827721 1.36728735932173e-08 7.12020359433536e-08

PSRC1 4.22702399104478 2.10023717750865 -1.00909004152317 3.17636895090773e-29 3.2295052344731e-27

CYP17A1 0.0767836778159204 0.244101472048443 1.66860942553727 1.23802130445972e-13 1.56541297628171e-12

HTR2C 0.314464487597015 0.0486158093806228 -2.69339965767344 6.21742701225111e-05 0.000160785390303582

AL162408.1 0.0527098169651741 0.176448336089965 1.74310223811873 5.37544944449884e-06 1.71510198678305e-05

SDR9C7 0.040185011238806 0.0142183971937716 -1.49889864286014 0.000358052955042854 0.000796028964430341

PKMP3 0.296817403631841 0.622827440968858 1.0692568234735 4.85093028205266e-26 3.52853572378033e-24

TUBAP8 0.0077277524278607 0.0335811198200692 2.1195295594652 1.05359384050005e-08 5.61820852752513e-08

LINC02802 0.830085536019901 0.324798425951557 -1.35371536547394 6.64070504567772e-11 5.2707587721109e-10

AC063965.2 0.253585453233831 0.635540591107266 1.32551228255028 2.89104574922826e-05 7.99495462501445e-05

AL110505.1 0.0585240908457711 0.167692993771626 1.51871989144423 1.39621130873199e-07 6.0748296681228e-07

AL353600.1 0.117069019900498 0.266427448546713 1.18638337853858 1.03694289121425e-07 4.62347376503256e-07

AL031846.2 0.194055298960199 0.410356937612457 1.08041151558809 1.49899389281512e-21 6.15577822349753e-20

MAPK10 0.42545382119403 0.937745211245675 1.14019344041409 3.46523161411894e-17 7.64307134243169e-16

FAM53B-AS1 0.0198975012288557 0.0646395062802768 1.69982891336862 0.000527264171414501 0.00113209884504527

AC130352.1 0.02712937 0.0642339748096886 1.24348102777998 3.44218503784874e-09 1.99626295133933e-08

CHEK1 4.15236924029851 1.85991727820069 -1.1586962805186 5.94665347436696e-34 1.0989254900266e-31

AC087763.1 0.206713395124378 0.463842156782007 1.16600206769438 7.08153044229075e-15 1.09547430767337e-13

RNF103-CHMP3 0.128479421293532 0.0593734813875433 -1.11364668599608 1.49274033884286e-08 7.72935408317914e-08

CRB2 0.0506558032089552 0.137477916757785 1.44040043438439 0.000467848278408631 0.0010155278106727

MALAT1 24.7124229830846 76.6759201453287 1.6335371048173 1.53311720718929e-12 1.605927063065e-11

SFTA3 9.04690242286816 22.2922164601557 1.30104425115308 4.10515367925628e-29 4.11267227573843e-27

SNORA63C 0.189320388308458 1.04081718480969 2.45881499616637 0.000892598471798802 0.00183098838998704

PNMA2 3.26185902925373 7.49207682698962 1.19967126054904 2.88957967157845e-27 2.37327339392404e-25

CENPU 8.95527810447761 3.74843988685121 -1.25644796947534 3.84443533863503e-39 1.34801675014959e-36

RN7SL395P 0.112096504477612 0.625395226574395 2.48002691664702 0.00017218670687696 0.000409398107718409

WDR38 1.16003303542289 3.00715226761246 1.37423202983442 0.000235770930102611 0.000545267625427567

RNU6-130P 0.173646789054726 0.570570889065744 1.7162504077264 0.000662189244429874 0.00139582858074428

TTC21B-AS1 0.0202809227064677 0.0426811950795848 1.07347728130904 0.00210836968354269 0.004001937042466

RNU1-38P 0.105857692587065 0.537908852249135 2.34523561868452 1.72953496261009e-16 3.44521349070546e-15

LINC00968 0.145996426512438 0.339860623657439 1.21901016317674 8.11548851444964e-14 1.05744931334063e-12

ADAMTS7P3 0.0852981312238806 0.552081725882353 2.69429580881311 9.36019799339046e-21 3.40427413722379e-19

NAV2-AS2 0.0810121691542289 0.196408979896194 1.27765034950498 4.46663668660363e-06 1.44810945209352e-05

AC136475.3 9.20905488029851 20.5704470566436 1.15944814212108 4.77227787380708e-08 2.25777200914416e-07

POLQ 1.08477486552239 0.494511147231834 -1.13332070882256 2.98273435676716e-28 2.73750955226785e-26

SPX 1.67621853962687 0.339504160643599 -2.30370909492932 0.00321072314112805 0.00586046969499814

SPINK14 0.211169871641791 0.479285432491349 1.18248107609224 1.91538812405603e-06 6.66911078204105e-06

AC036164.1 0.0380583087562189 0.0867594042560554 1.18880869437817 0.000261111376764983 0.000597856521935729

TEPP 0.36274318300995 1.00225126212111 1.46622382699478 5.61059925581504e-12 5.3489195052554e-11

VIP 0.0685006205820896 0.147033977117647 1.10196061316839 9.57267143041496e-12 8.79746517546536e-11

ZNF415P1 0.0231806330895522 0.0686526402733564 1.56639523670712 0.0048455818705466 0.00851112094017401

AC025244.1 0.313439611940299 0.10785384633218 -1.5391098891114 3.67221460988315e-07 1.47568424302533e-06

HPDL 2.24123939651741 0.81496053100346 -1.45949466198775 7.68677009876155e-15 1.17975960831161e-13

TUBA3E 0.385251670840796 0.099411331567474 -1.95431899944921 0.00299555903480808 0.00550301783695687

CHIAP3 0.0318243613034826 0.0895645612318339 1.49279644146565 1.58032993919364e-06 5.5972576841422e-06

GPR37L1 0.179991939268657 0.0882832768442907 -1.0277202137788 1.15372774607805e-07 5.104322407836e-07

MACROD2 0.957298565223881 3.04185844622837 1.66791216621325 4.44452628689026e-12 4.30293075916632e-11

NR3C2 1.62980661278607 3.37083549539792 1.04840543276144 2.91060833009435e-25 1.92748517743536e-23

AL121895.1 0.0437978995870647 0.102191468515571 1.22234116811975 1.6233076842959e-07 6.97086907921069e-07

AC104162.1 0.0427090948059701 0.116583035813149 1.44874264856466 1.33575714545854e-14 1.9673106046468e-13

ENPP7P2 0.0470628425373134 0.11334043816609 1.26800231963008 3.47393412469038e-13 4.08127570061348e-12

CCDC114 0.881304176467662 1.82764182858131 1.05227141935803 5.91595114940727e-08 2.75760738559277e-07

CENPUP2 0.0216348649502488 0.0601504459584775 1.4752173184986 2.14934490358258e-06 7.39242745384602e-06

KIF26B-AS1 0.236752420447761 0.513899991868512 1.11810845409614 5.58381719404349e-06 1.77702350776227e-05

SNED1 1.63174250746269 3.50772346055363 1.10412159924759 8.64666595705037e-40 3.35959886483275e-37

PTPRT 0.188070319955224 0.400280882795848 1.08974052501642 7.94142916107761e-12 7.41037487394994e-11

PPP1R42 0.100927609482587 0.240144665418685 1.25058287268717 0.00165920192342468 0.00321612904714417

SFTPB 794.568402367164 3234.95412297232 2.02550192457958 8.67695548609775e-33 1.39596901496926e-30

RPL35AP2 0.0804916149253731 0.38402294183391 2.25428209535305 0.000898330273646908 0.00184177908427608

LL22NC03-63E9.3 0.040229205761194 0.0176670004982699 -1.18718613529818 2.27522256622539e-06 7.78411019218095e-06

CST5 0.396841797562189 0.796752738858131 1.00556808700379 1.14249101119964e-14 1.69821354110381e-13

LINC00922 0.299156802477612 1.32916779523875 2.15154946943776 2.03345595930215e-07 8.57893556700845e-07

MCCD1P1 0.0384648429850746 0.102796985224913 1.41818563022811 5.70789978916851e-12 5.43940972939926e-11

AC243960.7 0.037440399800995 0.0831213470934256 1.15062319466932 4.46436489005683e-05 0.000118948251089191

AP003064.2 0.105271934626866 0.406996797197232 1.95089657462061 1.06122188609453e-11 9.65870834764905e-11

MIR663AHG 0.38136794421393 0.853199074785467 1.16169881783143 1.10579821117078e-15 1.92144733643716e-14

AC006539.2 0.140363325621891 0.347340561245675 1.30718485859661 2.66918077305004e-05 7.43973159071108e-05

TMEM232 0.101380061671642 0.25180785116955 1.31254931918572 4.12098963058372e-09 2.35251651839835e-08

AL161781.1 0.054842010199005 0.111314717854671 1.02129099786333 1.7599589198764e-06 6.16880910401674e-06

SLC6A15 0.410076669905473 0.0618547876920415 -2.72893649585033 2.3205678150371e-08 1.16028390751855e-07

AP002358.1 0.0623557468905473 0.236172152764706 1.92124443116276 3.33205245084421e-08 1.62098247119511e-07

SIX3 0.555117733258707 0.235821911899654 -1.23509600513223 0.00202207938484485 0.00385446551265031

TRIP13 10.3579555975124 3.98245423564014 -1.37900959081594 1.35413485565371e-38 4.40899860739631e-36

AC110792.3 0.342412024577114 0.698690070242215 1.02891927073968 6.00082975692205e-19 1.71856223928605e-17

LINC00930 0.252785631298507 0.560934924 1.14991894753271 1.18895795326185e-09 7.51233535506528e-09

PRC1 10.0902840945274 4.09225805536332 -1.30199776590652 2.20013716532595e-41 1.11432873095675e-38

MUC6 2.59534731327363 14.3406962181903 2.46611554607844 0.000104237679852207 0.000258116844179073

RNU6-1165P 0.246407805174129 0.511395921384083 1.05339269914052 3.03203526373741e-07 1.23714999945126e-06

TNFRSF14-AS1 0.676790485522388 1.46336201602076 1.11250552494856 1.22599978486884e-13 1.55092942257922e-12

AURKA 13.8513191691542 5.49422786885813 -1.33403473048835 2.70795911439009e-39 1.00084705106174e-36

AC083843.3 0.427319327631841 1.00440731454325 1.23295796347876 1.85610295118293e-18 4.93337373322188e-17

AC113208.2 0.0250451321890547 0.0695676439792388 1.47388623612273 3.44420265560129e-05 9.3618507881828e-05

AC008522.1 0.0170237659004975 0.0351632882422145 1.04651977114909 1.44459950550579e-10 1.07568190785688e-09

ACSM4 0.126807468432836 0.0613758550899654 -1.04689659237737 8.70226016892265e-07 3.23422768881688e-06

AL645608.3 0.583048275323383 0.189010811557093 -1.62514658084217 0.00286321863839321 0.00528401010526682

RNU4-29P 0.0931888737313433 0.235698314809689 1.33871182371577 2.33681517266941e-09 1.39637087551908e-08

IL23A 3.71619050597015 1.75509434290657 -1.08227587873048 7.70206099341594e-08 3.51730452779973e-07

AL157938.3 0.0334845922587065 0.0869382907612457 1.37649433149521 0.00539458125202665 0.00937606744045049

RN7SL650P 0.110959434477612 0.320124657439446 1.52860146414904 0.00244807058471898 0.00459254616174389

PARM1-AS1 0.0202356207810945 0.126193962031142 2.64067387044848 7.67746253381777e-13 8.48055736267835e-12

C1QL1 0.899244576865672 0.306545963356401 -1.55261014668424 1.20003442553264e-10 9.09167355632069e-10

IRX4 0.368509598965174 0.0767633296989619 -2.26321300265314 9.06629416103958e-05 0.00022743927438817

AC004223.2 0.0769427210447761 0.198104559100346 1.36440532631368 0.00247226735041799 0.00463380701986925

KHDC1L 1.23834335915423 0.318760499342561 -1.95786662297617 0.000154325388109515 0.000369856235961728

SLPI 395.656099391045 907.538290117647 1.19771151558458 1.31523158648016e-05 3.90443763054731e-05

LINC01187 0.00707943041293532 0.016880067200692 1.25361545180436 6.31532708629524e-05 0.00016302425277034

THRB-IT1 0.0241981207462687 0.0617234420484429 1.35092350648871 0.00250224075185594 0.00468484970997125

AL162385.2 0.0883366729850746 0.20167764799308 1.19096679595517 3.98417976021833e-06 1.30359273169005e-05

CCR9 0.0501004283233831 0.111119204048443 1.14921332777083 3.16895462400038e-09 1.85036099415906e-08

ARLNC1 0.272922665293532 0.0510536410276817 -2.41840645091341 0.00556205809225558 0.00963531092115468

PNMA8C 0.0441127873631841 0.094373032283737 1.09717773569841 1.38904883548511e-08 7.22389915392998e-08

NSA2P3 0.970227443880597 2.29172007685121 1.24003594290601 1.67407703976554e-11 1.46609052313761e-10

MUCL3 0.839988626741294 20.0749350310727 4.5788817142466 2.34110790021331e-19 7.05168513995969e-18

MRAP-AS1 0.0520173670646766 0.109415113183391 1.07274674494313 0.00299681639532103 0.00550456201558295

ADRB3 0.0473862789701493 0.101200984678201 1.09468204519398 1.92730497908572e-07 8.17110388745845e-07

MAMDC2-AS1 0.0623074799502488 0.144040927647059 1.20900152301597 5.00143773812202e-21 1.8919684942965e-19

HMGA1P1 0.136734983034826 0.0505224180276817 -1.43638680327111 2.98871341123291e-17 6.6673174385987e-16

AL049555.1 2.25676721933831 0.84317223032872 -1.42035835726654 1.45166318590966e-06 5.1764000175527e-06

ZIC5 0.244849636452736 0.0786334510138408 -1.63868097701419 9.64557375510991e-07 3.55774028593737e-06

LINC01819 0.0329891838656716 0.395870427550173 3.58496330671613 3.11157660974983e-10 2.17539929132561e-09

LPA 0.00907500793532338 0.0190263914844291 1.06803115854099 0.00182467048381098 0.00350626977673226

AL022329.1 0.0568674535820895 0.17849017982699 1.65016959256855 0.000101275515745244 0.000251259556933275

ZBTB16 0.362910576552239 1.31766703824567 1.86029985327781 9.9421170561643e-19 2.74109779723885e-17

AQP10 0.0289275557661692 0.0609240791522491 1.07456811234508 1.58477800632709e-07 6.81933267354404e-07

KRR1P1 0.25673487681592 0.526655328858131 1.03657979875877 6.49390218267711e-09 3.5967643721389e-08

AC005100.1 0.0374119815870647 0.0842580712698962 1.17131450928706 2.36661098875082e-06 8.05861685039031e-06

LHX1-DT 0.203426328646766 0.0432503369411765 -2.23372313626023 2.51354411360879e-06 8.52075254179479e-06

CRABP1 9.94869223169154 1.65103135820069 -2.59113937225281 0.00532499880927834 0.0092669074467907

AC135178.2 0.041624257960199 0.100717686297578 1.2748205882771 3.0122702398537e-09 1.76565775953705e-08

MIR6793 0.173164917412935 0.373889919031142 1.11046689834032 3.41935091942026e-07 1.38117335173747e-06

SNX29P1 0.0351067868159204 0.0888917913148789 1.34030024183428 8.49055735400589e-12 7.87976734414866e-11

MIR642A 0.220132145771144 0.668855408442907 1.60332452967686 2.31166772119023e-12 2.34772046693475e-11

AL513548.1 0.0952277103283582 0.51323067950519 2.43015406348519 4.73706213247249e-06 1.52763411535341e-05

CFAP161 0.198323801144279 0.441963655259516 1.15606990692035 0.000306933321923059 0.000691826796983325

MAGEC2 3.45747492551244 1.16168422321799 -1.57350082961294 2.38892160820497e-10 1.70412032388793e-09

PBK 8.175348739801 2.76870286262976 -1.56207004177538 1.49837356636724e-34 2.92717978858172e-32

ERN2 2.8982435630796 6.8140517357128 1.23333405970536 5.27790832793128e-07 2.05569890263766e-06

TK1 53.3348117064677 22.6403652941176 -1.23618025378334 5.45269797407308e-38 1.60356225366558e-35

MS4A2 0.573133437696517 1.45916990121107 1.34820490256714 8.01833952404734e-18 1.92707896294108e-16

LINC01167 0.0401824701492537 0.113258248546713 1.49497796577872 3.51057247191606e-09 2.03204565305617e-08

CKS1B 20.5926024378109 8.69937151557093 -1.24314308280616 5.60663634249267e-42 3.03343131515365e-39

CCL14 0.259370913298507 0.570203675415225 1.13646063849435 1.07631063376788e-15 1.87265111516355e-14

AC012511.1 0.626877543482587 1.56552938020761 1.32039502929631 1.10271895759175e-21 4.60448297559303e-20

NEK5 0.272768248835821 0.588071293321799 1.10831534765336 7.53745778084951e-08 3.44904584751939e-07

AC112907.2 0.729118904029851 0.334047275121107 -1.12610181650542 1.97259220560696e-09 1.19517937136354e-08

LINC02679 0.0613703274129353 0.188230204290657 1.6168849627334 2.39874886175294e-10 1.70996544555900e-09

SPC25 4.73637382139303 1.65016135709343 -1.52117584924455 2.64064612810735e-50 1.44443343207472e-46

LINC00648 0.865429749676617 0.311778606719723 -1.47289477696001 6.6642709366652e-05 0.000171272138806421

CFAP65 0.174480364975124 0.534122939051903 1.61410715228455 1.19851373799084e-08 6.32073866834737e-08

AC007036.2 0.0982383930845771 0.224756268754325 1.19400248759405 3.25877349438999e-10 2.27134187236407e-09

PADI3 2.99259934472637 0.707988117134948 -2.07960208937877 9.06702798140011e-07 3.36029757498704e-06

LINC00609 0.0507150839303483 0.124114267404844 1.29118215861838 2.7781460637233e-05 7.71942444811869e-05

AC078925.3 0.0327059055323383 0.0819826078650519 1.32576672374589 5.83410248157632e-10 3.88892768391695e-09

AC027288.3 0.343068215253731 0.816856411266436 1.25158703210222 8.82957287725223e-11 6.84686187107594e-10

LYPD3 14.530189219403 3.94694193979239 -1.88024628838776 3.01191002885178e-16 5.71656761201223e-15

AL672277.1 0.0282060889054726 0.0829862840484429 1.55686627415836 5.6203384606117e-07 2.17605120183649e-06

GPIHBP1 0.924608072736318 2.63006814951557 1.50818631878559 1.09908433121213e-14 1.640827317612e-13

CDKN2A 10.442463158408 3.60692960359862 -1.53361888214992 1.35801606178596e-07 5.92466729779008e-07

AC087620.1 0.0681778171641791 0.198032830207612 1.53836530763865 0.00533570659427388 0.00928415875215351

C1orf105 0.123702259691542 0.0478892231211073 -1.36909891764866 8.27144310997728e-10 5.37349095149355e-09

AC092145.1 0.037900867318408 0.0879214832076124 1.21398486108585 2.2672169653081e-07 9.47271371848099e-07

HAR1B 0.252814295472637 0.556665635432526 1.13873298073031 0.000244444335790065 0.000563325967632143

AC010627.1 0.0516276194975124 0.200861363311419 1.95998509775404 6.83260118257308e-09 3.76909322999947e-08

TICRR 1.17287023910448 0.464469424463668 -1.33638787540047 8.8792376792889e-32 1.2327266524292e-29

LINC01797 0.0156306336318408 0.0362900039100346 1.21519594902168 2.12310151132165e-07 8.92374770779885e-07

LINC02323 0.674314575412935 0.251294907896194 -1.42404034455311 9.16927126137409e-10 5.90625456897271e-09

C1orf194 1.36313133865672 2.950104283391 1.11384137976335 6.13375174520211e-06 1.93492630024542e-05

AC008121.3 0.0348887269651741 0.0737489265743945 1.07986109407557 9.75911398997318e-05 0.000242956278559773

MAGI1-IT1 0.0448123476517413 0.126577804851211 1.49805623922254 0.000174350255916036 0.000414002387506823

AC087521.1 0.0943248754975124 0.220248876816609 1.22342446609336 1.34265495893451e-14 1.97534228762016e-13

AC027117.1 2.45794400288557 5.83020251349481 1.24609394818829 1.98659068654567e-17 4.5202375438456e-16

HIGD1C 0.0319067853233831 0.089970193633218 1.49558386655 6.81634868494742e-06 2.13572157788191e-05

GDF10 0.826635994189055 1.8751231750173 1.18166127853063 1.0879421563251e-14 1.62508017342935e-13

MYH1 0.0192898153532338 0.074494106200692 1.94928695380718 3.48286387938467e-10 2.41309254885417e-09

AL513318.1 1.00207479029353 0.301580266799308 -1.73237625130606 0.000454004387128553 0.000988458843175125

RSPO2 0.186872470686567 0.547486493010381 1.55076931994669 2.5622851802105e-21 1.01269508206297e-19

AC073439.1 0.132375965144279 0.32836415250519 1.3106554339325 5.19224076998467e-10 3.49859041781426e-09

SNX30 3.97071031094527 8.65820315363322 1.12467054118924 2.8489793870502e-27 2.34697548903081e-25

FAM187B 0.0159027862238806 0.0348432207785467 1.13159843461576 6.92811390416299e-09 3.81639305697599e-08

AC011373.1 0.0680730954726368 0.189313552871972 1.47562107694243 8.10929741802506e-07 3.03239382530743e-06

BEAN1-AS1 0.094410087960199 0.199704720702422 1.08085550760036 1.01182384103627e-10 7.76687680391299e-10

SCG3 2.26191409220896 0.757972427442907 -1.57732686268527 0.000235222455484244 0.000544229266347523

AC020765.3 0.0169198805472637 0.03518735 1.05633748394262 5.86760677535432e-06 1.85890241290329e-05

AC022613.3 0.0633193805671642 0.163850132290657 1.37165779045404 1.68009075216165e-07 7.19888486160444e-07

LDLRAD4-AS1 0.00836565134825871 0.0793498430346021 3.24567759145349 8.05271344983931e-14 1.04976984200717e-12

AC018742.1 0.0782025613930348 0.214431724083045 1.45523059451753 2.01462554330096e-05 5.753969153016e-05

PRG4 0.843664802487562 8.23392166138408 3.28683790381139 9.28389283331903e-17 1.93090850943936e-15

AC008543.4 0.0620841150248756 0.140328389688581 1.17651081887923 2.94377105029901e-14 4.11046483140777e-13

SPINK7 0.0240612170099502 0.0542059091107266 1.17174051616346 8.43883568903159e-09 4.58122580577638e-08

LINC01606 0.0986285312835821 0.0402610053425606 -1.29262185241412 0.000374387984095161 0.000828908877600798

ARC 0.249618568527363 0.56809638183045 1.18641045775622 1.032074802449e-11 9.4122193554452e-11

RNU6-50P 0.278895164825871 0.578281953010381 1.0520501565396 1.70804968478747e-10 1.25338150707692e-09

ZNF474 0.299602214626866 0.621390043425606 1.05245083716509 1.03634895032921e-05 3.14237279971565e-05

ERVW-1 0.0197953440248756 0.0480924053737024 1.28064794507301 4.66969802362308e-09 2.64258723248689e-08

CEACAM8 0.0676474937761194 0.362138114719723 2.42043163627394 6.0606649289067e-11 4.84879685265828e-10

CD1C 2.55046577756219 5.36558539186851 1.0729748367262 6.27808590499289e-19 1.79046558395783e-17

AC005277.2 0.0830778195522388 0.215109868581315 1.37253845566646 1.32599133960147e-15 2.280871895478e-14

SMC1B 0.888822045054726 0.420975288235294 -1.07815905187669 1.48897475569878e-05 4.37322375089794e-05

CCL7 1.94084360492537 0.921997260380623 -1.07384950000408 1.69803852624699e-09 1.04151948178639e-08

HMGB3P7 0.0966381583084577 0.218066933356401 1.17410615734616 1.45063758947771e-12 1.5236151333416e-11

IFNWP19 0.921718061393035 0.334550326989619 -1.46210226679227 2.61793474807036e-08 1.29758092351802e-07

PIGR 123.438440405567 293.966848965398 1.25186173031387 1.75533896506065e-16 3.49152877777518e-15

DLX2 0.0877191532736318 0.0202222154532872 -2.11695082522212 6.54149714480298e-14 8.63881926172678e-13

CDC6 7.47957938258706 2.76936157785467 -1.43340371232805 1.5999607244034e-43 1.28702722977744e-40

Z97832.1 0.0388773573631841 0.0788480900346021 1.02014565152862 0.000101905285805994 0.000252745868918648

AC091173.1 0.0697220735472637 0.0222449493875433 -1.64813766006776 2.29845978104281e-09 1.37495352168681e-08

DNAJB6P8 0.034637113681592 0.0712513964359862 1.04059957444045 0.000333074390635068 0.00074570928158719

KRT8P14 0.0910014691741294 0.0412888028442907 -1.14013924846732 2.76010812683866e-05 7.67242171654003e-05

AL132765.1 0.0748699603482587 0.16066353266436 1.10158360840826 0.000135793464871443 0.000329250998602302

MYMK 0.0299615101492537 0.0620304522318339 1.04986630113712 4.89722783284861e-06 1.57612592643457e-05

AL033384.1 0.181299630487562 0.083382041083045 -1.12056739170897 0.0054164285978699 0.00941045115942966

AC010168.1 0.514632056069652 0.240608643114187 -1.09685285657312 0.000921580991380167 0.00188506769233771

TOP2A 31.7292881293532 13.0367067543253 -1.28323568204106 3.57944501603506e-38 1.07580023284131e-35

AURKB 11.0187946890547 4.12441933529412 -1.41770349467996 1.05365700144024e-40 4.57420936339533e-38

LINC00941 1.01561655984577 0.321597603709343 -1.6590272618932 7.74487457379186e-06 2.40379391276904e-05

ASNSP1 0.422936376243781 0.0920439515743945 -2.20004582324897 3.26527224810643e-11 2.73942318974573e-10

NKAIN1 0.803179586049751 0.178324971449827 -2.1712138590275 4.85263331876833e-11 3.94528897943858e-10

RPL23AP55 0.0800347978109453 0.170290691384083 1.08930027346002 8.49628287294088e-06 2.61770121183883e-05

AL663070.1 0.716695069402985 2.15533075346021 1.58847794542273 4.55429781517937e-25 2.9657153629799e-23

MIR27B 0.511360721243781 1.31953563737024 1.36761706146139 7.78893602889512e-17 1.63867231069447e-15

CENPE 2.1696112199005 0.915496713806228 -1.24480993177956 2.25458026080042e-31 3.02268481043586e-29

LINC01117 0.416502744825871 0.184118833702422 -1.17768879425806 9.04496793584935e-07 3.35293945575332e-06

CNTN6 0.159304691761194 0.394051324041522 1.30659479174918 2.18086174069584e-12 2.22644899619377e-11

LINC01839 0.0475430783084577 0.0990750762975778 1.05928685561507 5.40812499362296e-07 2.10132431560716e-06

PAPPA2 0.0912997607014925 0.183652524875433 1.00829574654414 3.95899480955113e-06 1.29612769979918e-05

PEBP4 5.6236237738806 26.8480879865744 2.25524736390558 1.48413233789137e-19 4.5659189472811e-18

CCDC78 1.02824644422886 2.224598303391 1.11335876869907 5.34348022253068e-08 2.50676130508086e-07

S100A8 128.017236074129 25.5710774411765 -2.32375321074182 1.4758745960273e-05 4.33800861916676e-05

AC004386.6 0.254701448258706 0.989801001384083 1.9583313388355 0.00414491695177166 0.00738861230730332

FOXG1 0.261863966492537 0.027495640550173 -3.25154275111518 0.000475815877110957 0.00103126747277793

AL365205.1 1.943004560199 4.10243151314879 1.07818996237853 2.102365827101e-22 9.72922256704101e-21

AC105254.2 0.12581163920398 0.40731260200692 1.69487105480502 7.72066984472864e-18 1.86208395285122e-16

GLB1L3 0.879730141139303 3.99501354617993 2.18306744767944 2.77002831827277e-11 2.3488751748008e-10

LINC01807 0.623819055174129 0.19157318283737 -1.70323200082219 5.77091778307626e-08 2.69434280244342e-07

TM4SF19 1.13721577154229 0.497388617612457 -1.19306061437871 1.69933993135143e-05 4.92813574463663e-05

HOATZ 0.340787243686567 0.937138083435986 1.45939030495757 5.87624353211851e-05 0.000152814738616945

CPEB2-DT 0.012633113358209 0.0258054343287197 1.03046468583625 0.000163879559543908 0.000390938155562659

BSNDP2 0.0540324872139303 0.126103805190311 1.22271281124681 5.37357711839612e-07 2.08997915512136e-06

CDKN3 9.23689917412935 2.93576879307958 -1.65367026738807 2.56762820183487e-46 4.77270339358322e-43

LINC02137 0.0527747862736318 0.015010058816609 -1.81391920084343 0.000450407369373291 0.000981252314191454

AP002478.1 0.419934127348259 0.0960487116297578 -2.12832487113426 5.68517947615592e-08 2.65794288329683e-07

KRT16P2 6.27416818587065 15.443884712699 1.29953958202048 2.01465957208915e-08 1.02019883904163e-07

MUSK 0.0512733596119403 0.124021060712803 1.27430379713442 4.65948002194798e-11 3.80067934984424e-10

CFHR1 0.0832743639900498 0.0233120617612457 -1.8367958256345 0.00433331735449411 0.00769485973545084

H3P25 0.103829729800995 0.272442840069204 1.39173398370644 5.18543574211683e-06 1.65989779432228e-05

AC005740.4 0.148566159154229 0.321303956124567 1.11283321252372 1.42734727581118e-17 3.30549940672614e-16

RN7SL15P 0.194527054129353 0.480713229515571 1.30520569380303 6.87925061612959e-06 2.15296377561671e-05

AC092484.1 0.635850615522388 0.132185911799308 -2.26611943918863 5.05031343291301e-05 0.000133172071336455

SCTR-AS1 0.324277276074627 1.10482573590311 1.76851899663421 4.27392105754038e-18 1.07833709339234e-16

PRMT8 0.141263246313433 0.382258995373702 1.43616429570719 4.8447498922788e-18 1.2078752010376e-16

SGO1 2.05074638457711 0.786592462629758 -1.38246081790938 5.10505413946093e-41 2.40729708128028e-38

RDM1 0.729997608955224 0.317516431349481 -1.20106048586286 1.07232672998303e-24 6.74210024483583e-23

CSPG4P10 0.584844860696517 1.17585407768166 1.00758315127326 9.73548290665087e-16 1.70464441419271e-14

AP003385.4 0.0770107214925373 0.341905879238754 2.15046801280266 7.23372768935591e-15 1.11775396781855e-13

AP000907.2 0.0573706219900498 0.140137802906574 1.28846211848744 6.45872641279364e-13 7.22479212228655e-12

GINS1 6.51573106467662 2.6703874816609 -1.28687796147039 1.32362436092387e-36 3.29101147920617e-34

LINC02685 0.015122327721393 0.0312868946055363 1.04887824523778 0.000126915084909325 0.000309369658847597

AL021068.1 0.689031070646766 2.24114310304498 1.70159382645774 2.56517380497034e-07 1.06122377198516e-06

SLC5A7 0.0507965750298507 0.130194802975779 1.35786872980934 5.1477396215215e-10 3.47288304510639e-09

TEX15 0.161056717737313 0.0360563185588235 -2.15924483074231 5.81577775715597e-09 3.24681611876334e-08

CAPN9 2.41961257068159 5.4233567917301 1.16441002438528 7.12146790336994e-10 4.67640209260907e-09

ZNF488 0.430450916079602 0.155725040737024 -1.46684778799036 1.12645064711448e-08 5.96831173936091e-08

Z97653.2 0.0885183728208955 0.184576987878893 1.06017385999235 4.66331117767395e-13 5.34765453708103e-12

SPATA18 1.2365027638806 3.60283598010381 1.54286750975102 9.18584310996329e-24 5.06517760196564e-22

GNRHR 0.0435077410845771 0.112371250100346 1.3689289534731 2.24413534967145e-07 9.38487795313672e-07

HMMR 6.59473945621891 2.4527562183391 -1.42691181261156 8.72144495075413e-40 3.35959886483275e-37

STARD6 0.0387809718557214 0.0187558209619377 -1.04801054535014 4.77199529490334e-05 0.000126467123367836

RHOBTB2 9.47519957014925 25.479194916955 1.42709145722934 2.55168069645487e-32 3.85571641149396e-30

FGF9 0.378957031248756 0.854038826591695 1.17226738461304 1.24929348257732e-11 1.11777765301244e-10

RN7SL145P 0.283107144527363 0.611661885051903 1.11138622139716 1.7778709168101e-14 2.56324562861129e-13

AC126323.6 0.0317105154079602 0.0963739460346021 1.6036818509766 1.48190655200684e-16 2.97796797923491e-15

AC024940.1 0.282504382373134 0.100259852858131 -1.49452922570241 1.33561718133791e-08 6.97520143394918e-08

CYP4A11 0.0189266885273632 0.0547174407301038 1.53157873870704 7.74501315906505e-11 6.06256754151199e-10

LINC01063 0.856519935671642 0.412950190899654 -1.05251904812623 1.2218893456529e-12 1.30287226524783e-11

FOXE3 0.127031857507463 0.0518076988477509 -1.29395193697058 0.00222884223802113 0.00420899228128688

PTGFR 0.352477301557214 1.05929765733218 1.58750577583413 1.37734671917261e-07 6.00229967644533e-07

ANKRD36C 0.406664045870647 1.12311146816609 1.46559177202323 6.22678948905963e-05 0.000160981843771416

FOXL2 0.160442119676617 0.0450608059238754 -1.83210790963522 0.000664675746921043 0.00140053017552315

EVX1 0.112663575288557 0.026474341799308 -2.08935443845969 3.42246440733804e-07 1.38222683905339e-06

AC090181.3 0.198359955572139 0.708284376716263 1.83620791244446 7.34500180996659e-18 1.77786959153492e-16

AC092881.1 0.0474229491741294 0.233678943280277 2.30087044915244 2.80815271319481e-05 7.79171925594786e-05

HOXC12 1.12129387472139 0.427357181456747 -1.39165016572393 0.00144953086400382 0.00284701394114933

AC079384.1 0.692286517412935 1.9253292666782 1.47566403785079 2.42674917129755e-06 8.25311985016016e-06

AC022395.1 0.0223422462139303 0.0701231221349481 1.65011599473042 4.01334410896691e-07 1.60053895275948e-06

SCARNA9 1.11511726179104 3.51531718020761 1.65645943989273 0.00216622332671618 0.00410178676167873

RPL32P1 0.326108459900498 0.848453255847751 1.37948331107667 9.46220407447456e-15 1.42899658441126e-13

AL049869.2 0.346309042736318 0.701683243044983 1.01875985038031 3.76161953278925e-12 3.68746574271634e-11

RIC3 0.242917436666667 0.593820631460208 1.28956116794369 1.00273057111944e-14 1.50437087877766e-13

HMCN1 1.26536705208955 2.66541004394464 1.07480155654367 2.53149744827359e-18 6.60023405245783e-17

CDC25C 2.53768232089552 0.97650921816609 -1.3778059088666 8.04095422568105e-41 3.60524750938322e-38

RNU6-1016P 1.16805288109453 2.81162352595156 1.26729784105087 1.03624210413529e-08 5.53215333751712e-08

AC023051.1 0.0574560599502488 0.13013129650519 1.17943700259686 0.00339025061611287 0.00615283333770025

AC078925.2 0.0466267080447761 0.122162357463668 1.38957132761992 3.70765494677783e-13 4.32427986329952e-12

GAL3ST2 0.576516216900497 0.215324559764706 -1.42084830855549 0.00402319872692956 0.00719085643585959

BTNL8 0.0933419405472637 0.27761998066436 1.57251403838965 1.96513223438936e-05 5.62141686126441e-05

AL020995.1 0.0246120913781095 0.0741348776851211 1.59078518225595 0.00452870648201591 0.0080085427572181

STYXL2 0.0641096008358209 0.170608137249135 1.41207412719283 4.01381213578604e-11 3.31454595150206e-10

AC133963.1 0.506364476661691 1.20894457901038 1.25550000637474 6.2440940132765e-18 1.53437530335231e-16

DSCC1 4.84072600845771 2.05026215743945 -1.23941504585062 1.63157717104894e-38 5.18879484048704e-36

CEROX1 0.277671381537313 0.666435634463668 1.26308705190065 1.27165728904076e-19 3.97029986932246e-18

MYEOV 8.15679602803483 3.95508532392734 -1.04429375733836 0.000327130692502821 0.000733603184646782

ERVMER34-1 2.53751612900995 0.931356135086505 -1.44601215144224 2.65960170897354e-06 8.97361297069163e-06

ADGRF5-AS1 0.0141990270149254 0.161635103702422 3.50887657687576 9.21283091837438e-13 1.00547057309473e-11

AC098935.2 0.119447867960199 0.335769252906574 1.49108902253675 1.13181748370054e-08 5.99094410280816e-08

RN7SL775P 0.0374092611940299 0.0960246156401384 1.3600088100785 2.2665452162063e-06 7.75747862135431e-06

FAM214BP1 0.0115912069452736 0.0263709195467128 1.18591708232136 0.000184912655865215 0.000436845567756209

NECAB2 0.722448424965174 0.244449687522491 -1.56335703724016 1.2208438120529e-07 5.36645423652311e-07

HOXB13 1.53127495381095 0.521663099809689 -1.5535430625274 0.000789536732526933 0.00163726056824715

LINC02555 0.320393730666667 1.29387283962976 2.01377801477853 2.09181640021592e-10 1.51352324195517e-09

CHODL 1.27537532232836 0.628150398131488 -1.02173994113251 0.000282237235480882 0.000641181858161153

AL354811.1 0.230192938457711 0.113216479307958 -1.02375961114281 2.67259242203565e-08 1.32275430225615e-07

LINC02809 0.0214756944975124 0.0436692128650519 1.02391173976171 1.7813028050559e-05 5.14506618632156e-05

AC129507.2 0.734482615920398 1.51454445570934 1.04408367558783 4.14687526314306e-12 4.02759369484953e-11

TMEM132D 0.188780283641791 0.584395004408304 1.63023574919821 7.59328907935777e-14 9.93667255121699e-13

AC120498.2 0.421230802487562 1.49942092089965 1.83172259446436 2.63019444316562e-13 3.15093377225492e-12

FAM153A 0.0747100350199005 0.151549712117647 1.02041716827286 4.31257955863056e-07 1.70767411218396e-06

CYP4F25P 0.038915426318408 0.0980960376816609 1.33385270201159 1.87621690768011e-07 7.97676549433406e-07

RNU6-341P 0.276131566169154 0.833123083044983 1.59317383064485 7.13734335640178e-07 2.70265262109913e-06

ARNTL2 8.35742217810945 3.13093005536332 -1.41646673676119 7.15940720530749e-16 1.27729802390841e-14

KIF18A 2.48497011691542 1.12852187820069 -1.13879411496773 2.5703678313478e-35 5.49215313963768e-33

AC131182.1 0.0253136311442786 0.16387412650519 2.69460171396871 0.00148755976647078 0.0029156341990093

AL162724.1 0.141294965970149 0.288232327820069 1.02852208914637 3.3439907611737e-06 1.10912135966651e-05

AC116158.1 0.0880142363034826 0.199269469051903 1.17891188181021 1.05464919843232e-07 4.6978266412254e-07

CYP2B7P 21.3749645179602 56.9359457688581 1.41341774176892 1.67043018109979e-22 7.87694231949642e-21

GLP1R 0.0923411793283582 0.28101426116263 1.60559728459239 5.10171336398727e-06 1.63577796606157e-05

CAPSL 1.22238774228856 2.65238642418685 1.11758899424365 0.000510555536735393 0.00109898425387321

BCRP3 0.342186739134328 0.810625352110727 1.24425144431126 3.40428362764242e-14 4.70951730986445e-13

RNASE1 244.210206865672 517.814425778547 1.08431165818542 1.32348365012716e-16 2.67731344903682e-15

NR4A3 1.35704637298507 3.05053921730104 1.16859425650806 1.45857469078391e-05 4.29269533981921e-05

AL157786.1 0.288727033333333 0.732663221349481 1.34344400371689 4.55031689304503e-23 2.29614699307715e-21

AL450344.3 0.0655823484079602 0.171978768269896 1.39085099867126 0.000495383220997356 0.00107011540117508

ALDOB 0.674991822383085 1.87104260306574 1.47090047973884 1.33998389016108e-09 8.3787287141988e-09

OGN 0.821529955427861 3.28985241889619 2.00163778064425 1.28096177009215e-16 2.5970574063766e-15

Z93241.1 0.0577482151741294 0.162815544290657 1.49539017994031 0.00166158662116728 0.00321980969880439

AC008663.3 0.0348775569154229 0.0754111824913495 1.11247948563085 6.75148861368195e-07 2.56890948225099e-06

TEKT3 0.124293611323383 0.303348085650519 1.28722206298783 1.55243657585727e-14 2.26340160127452e-13

AC021231.3 0.0738656039800995 0.152973273529412 1.05030499176332 2.97034352679086e-11 2.50737331659661e-10

KLF15 1.83481961621891 3.77745583051903 1.04177664957871 4.38716514253161e-15 6.98422390269148e-14

FCRL1 0.292300010238806 0.666728914688581 1.18965041556714 1.85509495022968e-08 9.4466002723744e-08

SCGB1A1 101.824408809303 408.657593935917 2.00480910573779 1.3507859607674e-08 7.04362906008164e-08

AC025040.1 0.0152249750646766 0.0441497976228374 1.53596695945393 0.00364883064240284 0.0065763109106898

SNRPCP1 0.178281552537313 0.459953270276817 1.36732986599035 0.00305410325369625 0.00560000831245592

ACOXL 0.462965960606965 1.30079429861592 1.49041481030104 1.14928283784258e-19 3.62547700288288e-18

AL392048.1 0.0342514774129353 0.0806785373356401 1.2360187097657 1.86599489574039e-08 9.50017877857403e-08

DLX1 0.127846386154229 0.028026623384083 -2.18954153467578 0.00112574032023854 0.00226107055581435

SLIT3 1.29461463855721 3.0866620249827 1.25352480123843 7.93818467777737e-20 2.54823181851187e-18

SERPINB10 0.0183805422935323 0.0501147007231834 1.44705453582096 4.44414168624289e-05 0.00011845558436677

LINC01435 0.00231402430348259 0.00711506916262976 1.62047376395329 5.42367912580784e-08 2.54176874727287e-07

TROAP 6.25200314925373 2.35528291038062 -1.40841814207525 5.52394957281204e-36 1.31373931144704e-33

AC123595.1 0.242186391691542 0.510175231453287 1.07487505596092 2.05432235534706e-11 1.76740221512243e-10

AC079467.1 0.727200682238806 1.62023508882353 1.15577769871847 2.47127018860881e-12 2.49960205837467e-11

RN7SKP292 0.0535218379104478 0.1172963616609 1.13195870075078 0.00331292891024371 0.00602771458855545

AC111188.1 0.00797484901492537 0.0208062215882353 1.38348588510704 5.891549012098e-08 2.74878651451519e-07

PCDH20 0.0469267170945274 0.129252680252595 1.46171275658632 1.01530693038844e-17 2.40213188115258e-16

GFY 0.585976597915423 0.118580966058824 -2.30497059376314 1.3257901901698e-09 8.29756560666911e-09

AC073862.5 0.0642788238756219 0.225116577868512 1.80825686554001 5.38197805763255e-27 4.30400876831141e-25

SLC6A4 0.254453206134328 0.613546142349481 1.2697714777647 7.38322386202243e-09 4.04835366311892e-08

F2 1.79739510089055 0.199113924183391 -3.17424155649968 0.000272617060559851 0.000621495090965402

FMO5 6.44453666368159 13.653727349827 1.08314630477282 1.84386115339924e-14 2.64861357906876e-13

SERPINB5 7.84402884964677 2.06697020580623 -1.92407724835597 2.82251280791298e-10 1.99112007470776e-09

RNA5SP298 0.128330121741294 0.267314163460208 1.05867643920246 0.000147556755646347 0.000355128235386096

AC108451.1 0.00509269148756219 0.0166900066747405 1.71248430434314 2.76082700595918e-06 9.29222478623967e-06

AC008764.10 0.0103377522089552 0.040874630384083 1.98328315775881 0.00513540828670397 0.00896721040933114

PRICKLE2-AS3 0.00944610113930348 0.0501520023425606 2.4085164146693 2.33804377487784e-05 6.59435879580375e-05

AC091133.5 0.491548979950249 0.0966928670934256 -2.34585380675118 9.9990475696833e-12 9.14934596958308e-11

EIF4EBP1P1 0.0612346094527363 0.126940781903114 1.05173644259621 1.36912883150617e-05 4.05651322085297e-05

FAM83A 35.6127340492537 16.487384589308 -1.11103063733353 1.81201841473018e-06 6.33338065723584e-06

COCH 3.53141621383085 1.24467804387543 -1.50447425348917 1.46178118140403e-13 1.82806197125744e-12

AC073862.4 0.0205283304477612 0.120721066020761 2.55598924654444 2.60613746982334e-20 8.88751369073172e-19

LRRC36 0.428429869587065 1.44874481920415 1.757672530504 1.90013553964347e-12 1.96404788394743e-11

HHIP-AS1 1.25720532608955 3.14438029657439 1.32255542550104 3.08348307172173e-13 3.66188719112416e-12

AC026992.1 0.0798126704975124 0.181853923633218 1.18809035149746 4.53326562932999e-11 3.70435658685913e-10

SYNGR4 0.643354285024876 0.266823587477509 -1.26972721784115 1.43262529083388e-15 2.45195880502545e-14

LINC01843 1.65843481666667 0.772412509134948 -1.10237887549393 1.47319848771342e-05 4.33247082139377e-05

SEC14L3 0.052858176880597 0.286846214512111 2.44007890769528 1.56162951214524e-09 9.6564700784925e-09

AL035448.1 0.0828580235074627 0.19000825617301 1.19734879556331 4.60357932343699e-06 1.48897699262064e-05

AL136452.1 0.394683885920398 0.975385966643599 1.30527559741069 1.69217529987919e-14 2.45132385867033e-13

SLC24A3-AS1 0.015555845681592 0.0481832655813149 1.6310753463522 0.000195893856285483 0.000460085613517214

GIPR 0.748312636517413 1.67253130892734 1.16032017486342 3.33221458750576e-18 8.54934980940737e-17

LRRC18 0.198136416810945 0.501780279083045 1.34056170408579 7.90548291700551e-09 4.31394568595572e-08

STXBP5L 0.180071049144279 0.0261949086100346 -2.78120791734157 2.13957198919154e-05 6.0790872537283e-05

AP000487.2 0.0647538976119403 0.138950067370242 1.10152759602512 0.000314651207076784 0.000707822874942428

AC079416.3 0.0228605756915423 0.0768641783979239 1.74944966958404 2.57034894227246e-06 8.69930003355424e-06

GGT2 0.206543291049751 0.508221313207612 1.29901267950683 4.50036697862861e-17 9.79976408164749e-16

KLHL33 0.0313546493681592 0.0764529759273356 1.28589327314761 1.41548161250733e-13 1.77584505055392e-12

AL138789.1 0.326323797960199 0.058369056366782 -2.48302855337882 3.60858607093597e-14 4.96952814904828e-13

OTOP2 0.0680318428059701 0.00711663582698962 -3.25694285122891 5.95583412880458e-05 0.000154663941723135

GP9 0.0413756899502488 0.0938637394809689 1.1817845682762 5.58355067244853e-09 3.12290615319974e-08

AL445487.1 0.042137680278607 0.0163406961972318 -1.36664144355771 2.2286845652513e-08 1.11759621985142e-07

FOLR1 55.3679052420398 135.22729526263 1.28826453918633 2.53890774317679e-17 5.71515446715105e-16

AC010998.3 0.206471235124378 0.810568694186851 1.97299365240092 6.25043927608216e-14 8.27441985483287e-13

WIF1 6.86689401768657 23.9610753518339 1.8029630538318 4.18717298896994e-12 4.06413037857464e-11

LINC00893 0.494142552223881 1.00866883301038 1.0294533839354 7.53722473855517e-14 9.86802760169861e-13

LINC00519 0.673038074179104 0.31901229550173 -1.07707609129155 0.000338565341209466 0.000756640143984221

AC107871.1 0.0395334536318408 0.0162144297577855 -1.28579570729565 3.59769555476131e-08 1.73972646046563e-07

AP003717.1 0.347204729378109 0.909405683678201 1.38913742149313 2.72732265569151e-18 7.07035778513391e-17

TMPRSS2 11.4531944731343 26.0351908754325 1.18471293938235 1.42240491945416e-25 9.75006880878979e-24

ZMAT1 1.06802170074627 2.40818575224914 1.17300571585764 5.70924101155411e-20 1.85890168650006e-18

RNU6-853P 0.18814621721393 0.501971597231834 1.41575145180953 0.000304196503846932 0.00068633677447728

CXCL11 7.64153658905473 3.24905679200692 -1.23384180847469 1.88321317761387e-08 9.57892512697403e-08

AC007378.1 0.0457291800995025 0.0980248451211073 1.1000324046884 1.53942045645503e-06 5.46297515038862e-06

UBTFL8 0.0220378127810945 0.0499935666124567 1.18176140886015 1.86710960434979e-07 7.94422023630472e-07

AC096564.1 0.00940000294029851 0.0225116343287197 1.25993768605858 2.94265549546645e-06 9.86861393252523e-06

CR559946.2 0.101654803880597 0.209729689204152 1.0448527089596 9.0320011119568e-12 8.33418456180899e-11

SIGLEC17P 0.336264626965174 0.693064462733564 1.04339252142528 6.64482669714255e-14 8.76681187490828e-13

AC110048.2 0.0164184887313433 0.0382201534844291 1.21901223407342 2.57448976204697e-09 1.52904006497252e-08

TCF21 0.7333227399801 2.03999630927336 1.47604635964632 1.25661723144025e-19 3.92782643198752e-18

AP005264.6 0.00483449320895522 0.00993497424913495 1.03915156554295 5.46119364513658e-05 0.000142945397831836

AL139807.1 0.0894268285074627 0.300365557889273 1.74793977500234 5.09411168065532e-05 0.000134171758923269

DTHD1 0.266395348522388 0.720696178605536 1.43582230365973 6.38473832922054e-06 2.00902541698489e-05

SLC4A5 0.261155152885572 0.571061165743945 1.12873810937946 1.71723370735027e-18 4.57315889932131e-17

SFTPC 108.143897054642 505.460634864945 2.22464648863415 1.4048838286314e-10 1.04839216133885e-09

AL136982.1 0.0111363751542289 0.0250683061522491 1.17058479998459 7.38607973080248e-10 4.83507134125055e-09

FOXI2 0.0442789900248756 0.100092217543253 1.17663558419425 1.42412156163647e-09 8.85824987736126e-09

EIF2S2P6 0.0159667603482587 0.0370923837024221 1.21605136410265 0.00392654129281894 0.00703050110367254

PCSK2 0.962654908169154 19.8006406121038 4.36238458201463 1.68440998092873e-10 1.23807075996777e-09

PRIM1 6.05224068159204 2.70379398512111 -1.16248413082385 6.72727234787167e-38 1.93674630225569e-35

LINC00466 0.0529437560746269 0.0229876171626298 -1.203603622172 0.000405975308847383 0.00089270177656986

SLC22A31 21.4873746362687 66.2033548968858 1.62341510575261 8.48660592993955e-30 9.17425581754335e-28

APOA1-AS 0.118028767313433 0.241444365951557 1.0325522664603 2.70213310286532e-11 2.2929984599245e-10

TPPP3 11.6699459029851 23.5786462031142 1.01468301326401 3.06038913719699e-10 2.14289920384889e-09

FOS 60.0361170547264 139.400983010381 1.21533815850048 2.30039573968579e-19 6.93669498130169e-18

CARNS1 0.423684083333333 0.903771806608997 1.09296962090897 2.95017626413998e-17 6.58672006728396e-16

C21orf62 0.0227399497412935 0.110333635782007 2.27857170028655 4.9742644567657e-08 2.34319898195904e-07

CDHR4 0.474950886094527 1.60875519058478 1.76009456375095 8.77972440959566e-06 2.69834209014992e-05

CYP2F1 0.206106816353234 0.540493645539792 1.39088543822712 0.000149982836168674 0.000360301323602392

AC002064.2 0.225799468507463 0.537195578062284 1.25040533897433 9.94317038058572e-06 3.02700033291429e-05

RBFOX1 0.06460651160199 0.144614724245675 1.16246296611018 4.07916437919243e-05 0.000109560194216747

AC131097.2 0.410572873666667 0.176804396923875 -1.2154841591372 3.73973625507122e-05 0.000101068959067389

IL1A 1.99622626695522 0.428607525010381 -2.21954617103021 0.000106922588922765 0.000264335593363379

LRRK2-DT 1.38023194722886 4.7315317216955 1.77739656614363 5.69580626147458e-22 2.50450645098601e-20

SFTPA2 548.43264632408 1877.39891033218 1.77534886955413 3.6626476051456e-17 8.05252508044471e-16

AC027682.7 0.106435145975124 0.213973060591695 1.00745454921824 2.15480720713511e-14 3.0630965236562e-13

LINC02334 0.0290248657661692 0.0613356357128028 1.07943612151786 9.79851949214203e-07 3.60977246915523e-06

GULOP 0.0238299643781095 0.0619050398269896 1.37728007229597 6.90938672936759e-12 6.5034495406406e-11

AC020659.1 0.159703679084577 0.409511167598616 1.35850524708792 4.73795911393201e-12 4.5708353356628e-11

AC002563.1 0.230130375422886 0.962337438269896 2.06409143502309 6.08540365459554e-14 8.07548713989267e-13

AL021578.1 0.447430387910448 1.00356689480969 1.16540163805323 1.34811350949401e-11 1.20022475536006e-10

AC127496.5 0.496453155820896 1.44595509079585 1.54229324513391 9.13407283208418e-28 8.08555680775226e-26

GAPDHP42 0.0336689468656716 0.0862224369204152 1.35664474257492 0.00337162011921273 0.00612309497081462

AC104837.2 0.0731934229353234 0.162054180484429 1.14669031732892 0.00325474406715104 0.00593369219014671

IL1R2 2.86031978 1.37680302958478 -1.05485427036282 4.26085809394597e-11 3.50058482635693e-10

LINC02065 0.198904501343284 0.0673220202076125 -1.56292555070132 6.60959532026129e-06 2.07545846164347e-05

CDCA3 3.41117777910448 1.38570270207612 -1.29965218008751 7.7590563232067e-38 2.21052281708024e-35

AL445220.1 0.0575545746766169 0.134403572525952 1.22356897688748 0.000129785973709041 0.000315861041194365

GPR176-DT 0.0152822934527363 0.00744083138754325 -1.03832533622764 6.27234413645314e-08 2.90908279009655e-07

FAM83F 1.24080994204478 0.40180826703391 -1.62670299886602 4.87800197689603e-08 2.30340735614825e-07

TIMM9P2 0.138907076666667 0.501641567785467 1.85253680011635 0.000477544031895972 0.0010346030792423

LINC02561 0.711959687363184 0.268322114740484 -1.40782959066922 3.05439270731938e-06 1.01974658868634e-05

RAP1AP 0.299328053034826 0.620089405051903 1.05074874497547 4.64649677776058e-11 3.79235114508361e-10

CHIA 0.80463920921393 5.028433296 2.6436950251851 3.62039643602756e-17 7.97245108899789e-16

RAD17P1 0.0965404316567164 0.323940180259516 1.74652224285778 5.26781553830071e-17 1.13623623795366e-15

DMRTC2 0.242627750059701 0.0795497295674741 -1.6088156340545 1.01166712814928e-05 3.07434399498698e-05

ANKRD55 0.0984363136069652 0.217751379391003 1.14541932222366 1.01743479356646e-10 7.79917270813055e-10

DNAH12 0.139804337351244 0.435346743972318 1.63875581302207 9.09854621555355e-07 3.3696037778658e-06

ALX1 0.551176936651741 0.095739267816609 -2.52533284349339 7.46620665164168e-05 0.000190361472846462

AC006111.3 0.0886561678606965 0.211546164256055 1.25467961820702 1.59026681263605e-09 9.81801294031512e-09

RN7SL381P 1.1336591719403 2.89441574460208 1.35228519328111 3.07023879209939e-06 1.02466175672872e-05

AC007552.1 0.0315206781094527 0.0795890898961938 1.33627210705764 1.90189054616944e-07 8.07712832884071e-07

AL132656.3 0.0119537665820896 0.0330452057370242 1.46697570085914 4.0269771178948e-08 1.93393896706625e-07

EPHA5 0.0469548561691542 0.139212309470588 1.56794050598192 0.0018113201097455 0.00348257328657571

CARMN 0.0591639353432836 0.19216380616955 1.69954670883581 3.92507746849444e-14 5.37560684843881e-13

WWTR1-IT1 0.11263520079602 0.235345201487889 1.0631206683655 0.00013700012161455 0.000331764948305113

LINC02574 0.173615475721393 0.085572668200692 -1.0206735719552 0.000142605845536347 0.000344030155721892

AC126389.1 0.0998171201492537 0.234421303391003 1.23174449684734 2.49120465269756e-07 1.03390663507251e-06

AC018946.1 0.0353533700995025 0.140910671487889 1.99486122116546 3.72214100220754e-06 1.22341733457969e-05

SNORA80E 0.418797256567164 1.25920308737024 1.58818708653725 0.00123081466578592 0.00244980577172294

CCT8P1 2.40574671094527 5.74077538650519 1.25476085349332 2.8695414601946e-16 5.4997868911228e-15

TSPOAP1 1.05127583915423 2.28187099653979 1.11807597180064 4.25197556844671e-20 1.4044870989978e-18

AC114737.1 0.0533251154228856 0.130021083910035 1.28585849781015 0.000418626461362548 0.000917937442336702

MECOM-AS1 0.189973287810945 0.415752177128028 1.12992724322483 3.73349823183529e-12 3.66121106635694e-11

ZNF474-AS1 0.192897018358209 0.45231107366782 1.22948447348324 1.68641214665416e-05 4.8952846753334e-05

RPS26P41 0.109985530099502 0.277269794186851 1.33397672492584 3.80510456583254e-07 1.52349011675479e-06

BTBD18 0.0206094240895522 0.047563412550173 1.20654803766334 0.000459288094326288 0.000998690521531561

RIPOR3-AS1 0.035153887761194 0.0161222836574394 -1.12462813920993 7.06275258640476e-05 0.000180918126101124

PIMREG 4.37624378358209 1.40031136373702 -1.64394545659528 7.24133712682869e-41 3.3567893291316e-38

STAC2 0.474892132532338 1.04796042118685 1.14191247037175 1.63098840307556e-05 4.75205420518979e-05

AL590822.2 0.0263921164079602 0.0656016722214533 1.31362554403628 5.92883300970605e-15 9.2680849826305e-14

FTX 0.302855107910448 0.673209755467128 1.15242833899845 1.10469659670763e-14 1.64830616039027e-13

ZNF280A 0.215271870253731 0.0433504494152249 -2.31204095862021 9.61984946980028e-14 1.23813121411312e-12

AL136172.1 0.041767076880597 0.0190743630415225 -1.13073129695303 1.74820329879835e-08 8.95046054326748e-08

ENPP7P11 0.0915459369154229 0.360350050588235 1.97683128647836 1.229559363821e-16 2.50211671134705e-15

SLC14A2 0.627406580587065 2.03944449800692 1.70070367961574 3.76519313081743e-08 1.81523060334667e-07

LINC00940 0.0182300431243781 0.151846732640138 3.0582259863851 8.91101608304271e-20 2.84383068694537e-18

C22orf15 0.239644883631841 0.690944057802768 1.52767076867435 3.38513122699837e-08 1.64504866841516e-07

AP006623.1 0.377521148059701 0.760245430899654 1.00990777673886 2.00904280493215e-14 2.87833005316366e-13

KIF23 5.12313942537313 1.86911986851211 -1.45466906151366 1.56813733711182e-44 1.86471983347862e-41

SLC14A2-AS1 0.0216274945074627 0.0543902066782007 1.33048036565805 1.24399385569952e-11 1.11441965127356e-10

PGR 0.104805501154229 0.242301500432526 1.20908889385532 6.85209099786351e-19 1.93800091821683e-17

TNFRSF13B 0.299913309771144 0.619929494955017 1.04755859744559 1.37167697237541e-08 7.13900896887402e-08

NRG3 0.033730978 0.102504766439446 1.60354494240957 7.36161249667138e-07 2.78055657759926e-06

AC022973.3 0.0609487333333333 0.223596726055363 1.87523092065894 0.000824181467940899 0.00170393553164892

AC096708.2 0.0426141886567164 0.141964099273356 1.73612036878771 0.000475251214881915 0.00103028858013795

AC104581.1 0.0431256766169154 0.209946199965398 2.28340067845252 2.13240548663319e-17 4.83994108376911e-16

SLC7A5 46.4429929751244 21.512584083045 -1.11028011720372 1.04448239613757e-14 1.56272393514018e-13

PRH2 0.118914632039801 0.657524947923875 2.46711938971597 0.00402727197079102 0.00719719605339352

FAM83B 1.2192366349204 0.485837268114187 -1.32743309077108 1.11682599335791e-05 3.36030703172044e-05

AC119744.1 0.0198241398059701 0.0648309562941176 1.70942458670313 0.00192383969742764 0.00368054111112521

NSA2P1 0.0371941255223881 0.111819211384083 1.58802139163607 0.00440100270265359 0.00780238697851661

TMEM100 1.65116020527363 3.9484895316263 1.25782075964495 2.00926671560171e-12 2.06591897262056e-11

ERVV-2 0.167706856731343 0.0450673318788927 -1.89578772918472 9.47399000026347e-10 6.08962694494021e-09

LPO 0.140202810029851 0.0642417586643599 -1.12593197168522 0.00182245675950151 0.00350250807198133

AFP 4.22658789854726 0.0284582459031142 -7.21450290271648 0.00104973643042123 0.00212369933959765

AL359313.1 0.0665439463084577 0.0258194433806228 -1.36584952689937 7.88671197988529e-06 2.44365665174876e-05

HOXD11 0.325425018258706 0.0712054050242215 -2.192266505988 7.59353089576946e-09 4.15615509304172e-08

AC079949.1 1.13720529208955 0.260044066989619 -2.12866469010554 2.88585259075392e-05 7.98140037992919e-05

GK-AS1 0.588370433034826 1.18628852681661 1.01165828728991 7.43108053939192e-11 5.8385536556268e-10

KCNE1 0.10208865981592 0.208760714044983 1.03202762360539 1.87129440089275e-06 6.52479846488679e-06

AC069549.1 0.153474982437811 0.36357090283737 1.24423323820119 4.34147725281675e-05 0.000115854622757867

PHGDH 9.01272754726368 4.48767593633218 -1.00599527906607 8.34759725545524e-07 3.1138404928628e-06

LINC01605 0.588702447059702 0.234558705307958 -1.32758957914155 0.000992271581427171 0.00201714194678409

HILPDA 23.532643079602 9.18207240484429 -1.3577716530992 5.45249376624512e-21 2.04843000696159e-19

RPS12P27 0.0841268215422886 0.190464920069204 1.17888756294867 1.26178193753296e-06 4.54733640684233e-06

TNXB 0.743903050945274 2.33954463795848 1.65304123556153 8.52556549171512e-22 3.62634861894881e-20

CFAP46 0.0996968974975124 0.359736544778547 1.85132021216521 4.59194809123045e-14 6.1989032722188e-13

RPL31P40 0.0378535779104478 0.126183843806228 1.7370256289476 3.70231239706017e-12 3.63715162850744e-11

LINC01269 1.25618478835821 0.613070822041523 -1.03492305536519 3.81632092929347e-05 0.000103026727288695

TH 0.140420354099502 0.0652572223529412 -1.10554258506121 0.00142833626993315 0.00280921882515976

AK4P3 0.14354145761194 0.0508874123529412 -1.49608673932433 6.07447913577789e-12 5.77265477288135e-11

RN7SL34P 0.126828937860697 0.278765175986159 1.13616639271785 0.00545927675679273 0.00947466332243678

CRYBG2 2.1334205881592 1.02490274926298 -1.05768138831239 2.45620607051191e-08 1.22162640531916e-07

AC010998.1 0.0519137492537313 0.211120408096886 2.02387745528763 1.57224298196648e-16 3.15255465958821e-15

CAB39P1 0.111510301940299 0.0387030590657439 -1.52665749476525 3.0680091377703e-15 4.99167459357631e-14

LINC01357 0.497136056915423 0.154183157508651 -1.68899556637897 0.000602116857466504 0.00127989485335263

KCNMB2-AS1 1.99444399291045 0.935151467875433 -1.09271464637025 3.69610819086777e-07 1.48419555161112e-06

LINC02397 0.0287217720696517 0.0633036067301038 1.14014293671522 1.17766337903633e-05 3.52937688106987e-05

CSAG2 0.831438572537313 0.219127974567474 -1.92383600470614 1.91762407331574e-12 1.98017656168288e-11

FOXL2NB 0.0588320342985075 0.0170699540138408 -1.78514275095582 0.00109883544403301 0.00221223035659204

ELOVL4 1.32844183910448 0.50452518017301 -1.39673688695337 1.61998206236619e-08 8.33612594651276e-08

E2F2 2.07772901144279 1.02265593038062 -1.02268666632947 3.04181541168388e-22 1.38195434401253e-20

SHH 0.733329819452736 2.12411126730104 1.5343252306878 9.10789332500347e-20 2.8998938584266e-18

SORCS2 1.8003764419403 5.44128010930796 1.5956475059965 1.15458911460887e-24 7.20959184578826e-23

RNU6-761P 0.112240167562189 0.432731271937716 1.94688231506922 7.26343342459915e-07 2.74612806418008e-06

ECE2 3.0292707880597 1.5053121083045 -1.00890790373238 4.39842114605063e-31 5.70127101158695e-29

TTLL9 0.120890539258706 0.32712663800692 1.43614789772685 1.6754506190631e-09 1.02881846500619e-08

AC108860.1 0.0887577126865672 0.255371407370242 1.5246526084677 8.3344615599548e-05 0.000210555628731539

MST1L 0.923089593502488 2.78084217956055 1.59097928479675 5.06485237456751e-16 9.25960644681961e-15

AL445307.1 0.0456981083084577 0.0976178895847751 1.09501111665781 1.08339110455335e-08 5.76226205431141e-08

F11 0.147700233532338 0.595353541221453 2.01107453719269 1.32723910296572e-18 3.6083488534903e-17

LINC02347 0.0132730932537313 0.0444638739204152 1.74412902326951 2.04578648664617e-07 8.62529064433062e-07

AC099811.6 0.0251379784079602 0.0804232944013841 1.67774480173159 0.00235441618516863 0.00442748093126802

AMPD1 0.289382322014925 0.657197552453287 1.18335031516692 2.19001017372751e-09 1.31525643942572e-08

MIR1972-1 0.160684448258706 0.450481014878893 1.48723600076745 1.99819933608089e-07 8.4389672393163e-07

AC073862.2 0.0812779698507463 0.302389298650519 1.89547081222944 2.00313762914915e-21 8.03311058023889e-20

STMN2 0.608929607233831 1.32166401780623 1.11800810763842 0.000260226271124923 0.000596479091121911

GPR158 0.455686747293532 0.204079676889273 -1.15890989347343 1.33013751439175e-05 3.94654599898181e-05

ACTL8 1.05321563816915 0.19470268217301 -2.43545618432792 1.5410146088483e-11 1.36082067649438e-10

HOXD10 0.527471675870647 0.214322006422145 -1.29931363372366 4.20380149682331e-06 1.36922675882003e-05

DLC1 3.79637332885572 10.2983625813149 1.43972119345898 2.60172096213567e-33 4.50361191863358e-31

AC021739.3 0.0613398115920398 0.214685899342561 1.80733179708725 5.67896097885838e-06 1.80415359242394e-05

AL138721.1 0.267688829900498 0.555165864429066 1.05236192386231 6.5153081134048e-07 2.48839096357522e-06

SEC24AP1 0.0268830852139303 0.0876385360415225 1.7048666677593 1.70652800198262e-07 7.30186809358959e-07

LRRC74B 0.0508183994427861 0.222401346017301 2.12974267692743 3.03850166544097e-07 1.23947790830696e-06

LINC01980 1.41966406875622 0.568435361038062 -1.32048137993495 2.53506256964459e-07 1.04940156318722e-06

AL355607.2 0.0328153277412935 0.0145719793702422 -1.17117298426001 0.00172231903766341 0.00332712428874801

CRHR2 0.148062453547264 0.438122364103806 1.5651280185572 2.11409004466212e-06 7.28124451851265e-06

AC122129.1 0.208373033283582 0.455739833598616 1.12904189126602 6.73853251721066e-24 3.80782777573784e-22

MAP3K19 0.178747522771144 0.479044917581315 1.42223768842158 1.46501361678688e-05 4.30972527620523e-05

AC134312.1 0.0120559820646766 0.0329588113598616 1.4509150377158 4.55481945392603e-06 1.47460123182856e-05

HJURP 7.4189100681592 2.5650534982699 -1.53221833639659 4.22258564946026e-42 2.45718547899443e-39

AL136231.1 0.0392044089552239 0.128617432214533 1.71399837709162 2.43561862179494e-10 1.7329388477131e-09

AL139041.1 0.32539384039801 1.00381063948097 1.62522829592515 1.31691297562738e-24 8.16724940666867e-23

AL357093.2 0.661582212487562 1.66511895297578 1.33163289302685 3.23041223388241e-08 1.57545960407782e-07

AC064805.2 0.0590210780597015 0.296009211176471 2.32633989223219 1.97687050097955e-12 2.03567048952525e-11

LYPD5 1.40768442900498 0.652048991384083 -1.11027168080739 5.54204762088048e-10 3.71325336675848e-09

AC008080.4 0.0159146347164179 0.04663381483391 1.55102240914083 1.52041921229444e-05 4.45600787143731e-05

CFAP100 0.241083478437811 0.780670281871972 1.6951805652258 3.93667223875846e-10 2.70590564790259e-09

LYPD6 0.748950239383085 0.349391586529412 -1.1000250004344 8.49007288110869e-10 5.50406000781966e-09

AC092652.1 0.0177638955223881 0.0667614376124567 1.91006703012578 0.000295098379682518 0.000667847801763911

AC012158.1 0.21608631920398 0.0748255545190311 -1.53000476515274 2.418070575044e-09 1.44114687791356e-08

RN7SL280P 0.100909808606965 0.329093109515571 1.70542940622428 4.4313320206165e-05 0.000118160213282501

LINC00601 0.150310168318408 0.031532799 -2.2530174680606 2.50262750492347e-05 7.02379294609101e-05

DNAI1 0.370366333477612 1.11186303385467 1.58595421111041 2.05322150910561e-05 5.85564215579129e-05

STIL 2.82176802288557 1.33845912871972 -1.07602630365705 3.05449963406384e-37 8.19025146976922e-35

UHRF2P1 0.00826485764179104 0.0727834329342561 3.13854822443626 0.00447240645852106 0.00791717259809391

ITIH5 0.797448930895522 1.76309869311419 1.14464919887347 1.51303568395876e-23 8.06657426048189e-22

WFDC12 0.283318403233831 4.40038815124567 3.95713456560977 7.00474635384275e-05 0.000179516316320839

AL512328.1 0.150328941467662 0.374108883224913 1.31533543814041 8.46420080374029e-12 7.8606414934566e-11

AC156455.1 0.76731503641791 0.302275771176471 -1.34395368162829 0.000190743631398535 0.000449224000581239

AC023510.1 0.0452989886069652 0.101616596885813 1.16558530995301 0.00362476214914167 0.00653637797712301

PPIAP41 0.106516180895522 0.216032959896194 1.02017883227031 1.23893190006247e-06 4.47323926953248e-06

AP000842.3 0.091166256119403 0.0439675074394464 -1.05206218324708 0.00434796256296971 0.00771685763122788

C1orf141 0.0359927700646766 0.107795868044983 1.58252283487651 0.00116638015579003 0.00233549288094716

LHX1 0.345818490920398 0.0839264744325259 -2.0428171292355 7.36223086293365e-05 0.000187920685115479

NIBAN3 0.184233174875622 0.388749254532872 1.07730703774893 8.23749919587232e-07 3.07570789088202e-06

IGHA2 195.309218368159 448.506034429066 1.19936735166917 2.71070786927851e-06 9.137029852695e-06

AC091182.2 0.310174095522388 0.101706008961938 -1.60867328482794 0.00378617140355674 0.00679830540226345

RNU6-890P 0.166948089751244 0.429419588546713 1.36298841722368 0.000187644538368217 0.000442534976663856

AL162411.1 0.719899468308458 0.351817988269896 -1.03296620386664 0.000714669919387231 0.00149584619998781

C10orf105 0.015700600119403 0.0369120324913495 1.23327147517073 9.47434593740417e-11 7.30336418793698e-10

ADHFE1 0.76170850880597 1.73720511792388 1.18945719138658 5.45694227027604e-19 1.57600180667423e-17

ADAMTS9-AS2 0.0800343317860697 0.192592887979239 1.26686352767189 2.00793380482941e-19 6.09516648714482e-18

SLC25A39P1 0.0306339010248756 0.0764211068615917 1.31884205711565 0.000367698365535587 0.000815285796303065

HMGA2 2.24760708020597 0.469887595413495 -2.25800226249301 2.56841969285365e-05 7.18632006133476e-05

MAPRE3-AS1 0.0199717852835821 0.0409058876297578 1.03434520534134 0.00298818768363096 0.00549203233299555

CX3CR1 0.886266934179105 1.86277357591696 1.07163912853843 2.28019831971102e-15 3.79108960754385e-14

IGHVIII-67-2 0.487651352238806 1.12675041280277 1.20824601620089 0.00309085639348678 0.00566208455203372

ADGRF5 19.9254577109453 50.8346618477509 1.3511996757854 5.76858902327519e-27 4.59973497920048e-25

HHIP 0.571938068343284 2.15972480588581 1.91691665457668 2.96691184881025e-13 3.52804517673741e-12

MYCBP2-AS1 0.0383656744825871 0.0783297811903114 1.02974481006293 0.000177901235901504 0.000421715595519564

PACRG-AS3 0.00524467002985075 0.0164421056678201 1.64847115729549 1.92629084915221e-05 5.52157114879381e-05

CCDC17 1.01363198378109 2.63419414598616 1.37782772676363 6.68436124040097e-06 2.09773126706789e-05

TDRD10 1.34039230457711 3.08165531228374 1.20105019437873 7.63137017514615e-23 3.77428524937156e-21

MAGEA1 4.01743082277114 0.751259770352941 -2.41888942822856 2.22904781764516e-07 9.3303424873883e-07

PPY2P 0.310321518955224 0.0988554653633218 -1.65037110797771 1.59816654088853e-06 5.65530532970647e-06

AC037198.2 0.233627477263682 0.505259199446367 1.1128137229055 8.90110313008646e-07 3.30319091733873e-06

AC079193.2 0.0213959235870647 0.0429754483321799 1.00617673399325 1.00611026982931e-08 5.38284739433326e-08

CFTR 1.42067794618905 3.21142244339446 1.17663290866872 1.20722594143282e-12 1.29025515819412e-11

AC103810.2 0.106421082537313 0.252724212138408 1.24777990360333 3.22718179210826e-05 8.82369509288822e-05

TYMSOS 1.2958419681592 0.63528877799308 -1.02840534782622 1.01024387686426e-15 1.76437867383381e-14

FAM183BP 0.0722144813333333 0.177994774422145 1.30147480897605 6.80140495459213e-12 6.40779970747829e-11

MYCBP2-AS2 0.028881333681592 0.113061503183391 1.96889851364 0.000653699631616546 0.00137942172090985

UBE2T 29.2286837164179 12.7260407785467 -1.19960121073237 5.88867158685153e-39 2.01318959875487e-36

KIF14 2.27890023845771 0.943474711730104 -1.27228201589438 5.27693681784585e-30 5.89078457012588e-28

FAM72B 0.623936230995025 0.231234965155709 -1.43203902191613 4.76613363923258e-38 1.41688864166316e-35

FAM72A 0.476179268606965 0.208019695640138 -1.1947846799079 7.33016912813123e-32 1.02285778395097e-29

TMEM252 0.0961480834378109 0.212530969404844 1.14434307687525 1.58031967344886e-11 1.39155644136595e-10

PRDX3P4 0.0209254041791045 0.102715504463668 2.29532657255396 1.19797149384133e-07 5.27864030232969e-07

LRRC71 0.236898254238806 0.756623424103806 1.67530787404701 1.00420522463499e-07 4.48774720486389e-07

AC127496.2 0.229160477263682 0.523485238477509 1.19179060829626 1.43109562812816e-13 1.79378851646678e-12

TBR1 0.0279570217164179 0.0104375553217993 -1.42142683318656 1.11232554669195e-07 4.93705025998456e-07

AC080023.1 0.195876373731343 0.0936921670588235 -1.06394304824124 2.10627890421169e-08 1.06324710280896e-07

AC104117.1 0.01029414 0.0249797496539792 1.27893570998767 4.97560644973974e-07 1.94821526700618e-06

SPINK13 0.710584973731343 2.63214619224913 1.88916053375276 9.35379300466983e-08 4.20282961520815e-07

RNU6-1079P 0.137942487412935 0.299598064913495 1.11896141838622 1.96630058721757e-06 6.81946754506728e-06

AL356234.2 0.680214007114428 0.327984834740484 -1.0523596051345 2.84852357611839e-05 7.89092675041406e-05

AL445309.1 0.159605434223881 0.421109659480969 1.39968619557809 6.93165125137843e-19 1.9584779103843e-17

AC063950.1 0.114697616268657 0.236307164809689 1.04282796384303 2.6846719931175e-09 1.58861486394988e-08

SCUBE2 1.79988732368159 4.14149807612457 1.20224612449389 9.14701085818622e-15 1.38675580361083e-13

AC007362.1 0.0493970805323383 0.12861004533564 1.38050564830673 2.21261211014651e-08 1.11016219432227e-07

DLGAP5 8.23171767014925 2.81274044117647 -1.54921707446677 1.16654311697845e-43 9.6681679543517e-41

AL161669.1 0.0523440049751244 0.114048844117647 1.12355560699535 0.00135857532654443 0.00268262473330374

MKRN5P 0.0329010523333333 0.0697509888650519 1.08407994128359 4.74416789411783e-07 1.86453501802159e-06

LINC02163 0.761119117114428 0.340972085882353 -1.15846862090996 2.91435789704796e-07 1.19412267392152e-06

AC067747.1 0.188143816766169 0.493434439273356 1.39102253522464 1.47968899206337e-11 1.3096923602891e-10

AL591767.1 0.0222827211442786 0.0630047432179931 1.49953501988807 5.33580482980235e-09 2.99352332502757e-08

AL035588.1 0.0301111202985075 0.0820720702076125 1.44659495946357 1.30267317040439e-07 5.69958585995202e-07

AC114489.1 0.461512506517413 0.218060176262976 -1.08164342540002 2.53958042690535e-11 2.16176547388302e-10

AC093797.1 0.173867757905473 0.404285941349481 1.21738561256286 1.6218928867447e-17 3.73390323673969e-16

ACP7 0.236555687681592 0.0583371458373702 -2.0196931402587 4.04317392956332e-05 0.00010865756801961

AC104024.1 0.0433370618855721 0.0919474709100346 1.085208546053 6.33698114324309e-11 5.05001265348772e-10

HYDIN 0.0863401827726368 0.214522669315571 1.31302605816606 0.00186945902269254 0.00358428034969203

AC004836.1 0.468311504825871 1.42703119453287 1.60747648638819 6.46915132937085e-13 7.23349504735457e-12

AL022323.3 0.0141264807014925 0.0433079743224914 1.61623059863898 0.000231944597261423 0.000537418225609956

FAM167A-AS1 0.0225000197263682 0.0520788525778547 1.21077139652641 3.88303968276271e-07 1.55174072652776e-06

CYP4F32P 0.138013991293532 0.553455349723183 2.003652402649 0.000419663999927103 0.00091999121497325

AC011444.3 0.0478596014427861 0.115250774117647 1.26789615319311 7.09210697568506e-07 2.68692513900798e-06

ARL5AP3 0.0539251971144279 0.114871947128028 1.09099507048403 2.7824974819742e-09 1.64330179511972e-08

AL049874.1 0.0412801532338308 0.10074089083045 1.28712916237889 7.19715152838948e-05 0.000184085003555085

LRRC37A9P 0.0658294693283582 0.0328377625605536 -1.00337773704727 2.63404693001888e-09 1.56034618878095e-08

AC002543.1 0.0158961442587065 0.0760060033217993 2.25743650376382 0.00456311565374434 0.00806313562022921

KIAA2012 0.0680921456119403 0.190169090892734 1.48172247806935 0.000364243265117024 0.000808476976217384

PRDM16 0.906333830582089 2.27428642200692 1.32729951549641 2.66145040852896e-22 1.22131994418233e-20

CENPF 7.41040436119403 3.29114253217993 -1.17096375998369 5.07505214201417e-25 3.27364802085112e-23

AL157996.1 0.0256560968159204 0.0601804874083045 1.22999408820181 1.07916051009984e-11 9.79589776011637e-11

SNORA16B 0.408750066616915 0.850982790761246 1.05791099116309 5.15661833178074e-11 4.17753291985199e-10

SLC16A11 0.739299793980099 1.98724008903114 1.42653476635299 5.57528896886053e-16 1.01049803378619e-14

ARHGAP27P1-BPTFP1-KPNA2P3 1.51649598358209 3.3006926733564 1.12202713884056 3.11123688927772e-18 8.01246035044686e-17

CDH7 0.0689212755049751 0.0188131437743945 -1.87320845045775 0.000235896921558892 0.000545466757240082

IGBP1P4 0.42893375079602 0.191133631038062 -1.16617318841995 0.000642030439240552 0.00135699633023409

C1QTNF7 0.321394988980099 0.935413700899654 1.54125712445037 1.02925919138844e-28 9.94708087790595e-27

MIR3680-1 0.167017571641791 0.356576114878893 1.09421017356031 0.00118279147199827 0.00236506409995267

NDUFB4P6 0.0616015390547264 0.146504449307958 1.24990617882459 0.000354827481309874 0.000789564039852335

RERG-IT1 0.109166669651741 0.221230052041522 1.01901493018708 1.06231303967507e-05 3.21396699503464e-05

COLGALT2 0.343090026268657 0.722677002110727 1.07476379694438 1.9433351362498e-07 8.23396064700729e-07

SLC22A3 5.27943628152736 13.8449190366782 1.39090081969677 1.9335328842351e-24 1.15969571017171e-22

AC026401.1 0.0302899176616915 0.0984683690657439 1.70082270653559 0.000105417037698559 0.000260871876678935

ACOXL-AS1 0.0913581369054726 0.229877041204152 1.33125725038292 2.83101437168006e-16 5.43356091687366e-15

AC004584.3 0.0390726574676617 0.0827476703460208 1.08255931369439 1.82105474839311e-09 1.10975595740979e-08

FBXO47 0.0505507215074627 0.0201280975363322 -1.32852086544683 4.66633218855841e-06 1.50749096807315e-05

CEP55 12.2716165427861 4.55392540103806 -1.43014274441744 6.67765958998825e-43 4.56584974465447e-40

AC092071.1 1.23855934557214 3.29765619574394 1.41277799835919 3.37906859895157e-22 1.52252926163633e-20

AC008013.2 0.0742197210447761 0.199241165813149 1.42464127395979 1.17919561628919e-11 1.059842264394e-10

GATA6-AS1 0.229729851447761 0.810538449688581 1.81894228991056 1.36172543516387e-13 1.71311824524986e-12

AP000344.2 0.142692752636816 0.376365834809689 1.39922360892761 1.50124356716413e-11 1.32705273309434e-10

ALKAL2 0.187745509348259 0.525129157882353 1.48389390318434 7.68672197191901e-15 1.17975960831161e-13

LINP1 2.13530626313433 0.876239658062284 -1.28504559110775 0.0012034183093895 0.00240069225104324

LINC01844 0.0812099401144279 0.196940016539792 1.27802805489323 3.02683249059588e-15 4.92938589326535e-14

AL590282.1 0.0926986270547264 0.247906968235294 1.41917894706746 3.0124586077909e-17 6.71481197417124e-16

AC012317.1 0.0559809369651741 0.316808752249135 2.50060465334592 6.08986605827864e-11 4.86584389991004e-10

CYCSP6 1.98841165437811 0.452841499238754 -2.13453838366684 8.78485864260364e-12 8.13082517344195e-11

MAGEC1 1.31884279741791 0.246574469072664 -2.41917727700328 3.28642932337473e-07 1.33240204557217e-06

DNAH10 0.178814060865672 0.423402427923875 1.24356935637023 6.26930576969425e-08 2.90816677071129e-07

DDIAS 2.10689887661692 0.798338310207612 -1.40004892268132 4.52122668162287e-39 1.56526012332134e-36

AC010275.1 0.55730770840796 0.212266267508651 -1.39259898597296 6.11388918849082e-10 4.06255756329504e-09

AL592429.2 0.0559066712437811 0.120288881730104 1.1054109480606 3.84236188896656e-05 0.00010362745060964

ENPP7P4 0.0716722634079602 0.15722401384083 1.13333476595937 3.36476954533784e-07 1.36113662276276e-06

RNA5SP494 0.212579931243781 0.528069202249135 1.31272159915629 1.23491426437771e-12 1.31531573254704e-11

AC073346.2 0.0118248036119403 0.0257526481972318 1.12290457191739 7.39583785429744e-06 2.30251753346653e-05

CDCA8 13.7896280447761 5.3085643384083 -1.37718988998102 1.60997349837143e-45 2.20163875902293e-42

ELDR 0.0467557005572139 0.0103938230449827 -2.16941586983744 1.73349722193865e-13 2.14145415305399e-12

SCML2 0.844637473084577 0.324099746747405 -1.38189436166423 2.73321264185931e-10 1.93261028321748e-09

AC009806.1 0.09585777039801 0.200095920103806 1.06172446229965 5.45033152521214e-16 9.89161029957213e-15

AC110760.1 0.0270515312437811 0.113619378200692 2.07042674795158 4.95419754085338e-08 2.33535509724819e-07

ZIC4 0.123369213587065 0.0359232975951557 -1.77999072512426 0.00022075244558975 0.000513581499202951

IDO1 22.7028356099502 11.2274957290657 -1.01583632970232 0.000448469878602063 0.0009772648537779

AC015722.2 0.277717608208955 0.764646930380623 1.46117509696571 1.4009129888085e-06 5.00980259465383e-06

EPPIN 0.0213888432686567 0.0589284910484429 1.46210686217896 0.000105557011314773 0.000261171002303152

DRC7 0.219070700019901 0.501789681107266 1.19568626166319 0.000164379418775947 0.000392027999958332

CDH18 0.383874781900498 0.0966836676574395 -1.98929168019915 4.83981453001725e-08 2.28655946443206e-07

ADGB 0.050081253721393 0.162832027698962 1.70104190967541 2.63765960756498e-07 1.08956336304036e-06

CFAP44-AS1 0.0318700800497512 0.0687036535640138 1.10818417877074 0.00448628451821717 0.00794019812161002

AQP1 82.0354559004975 176.910708429066 1.10870189166816 4.61240564021965e-18 1.15521331739934e-16

CFAP58 0.19855012419403 0.452898903252595 1.18968578246316 3.13255283427433e-08 1.53128364642364e-07

CYCSP40 0.0786756209452736 0.17721658916955 1.17152509516956 0.00033517858918405 0.000750051907558809

DUOX2 0.8413644110199 3.77437356808305 2.16543452077095 2.74625840108375e-08 1.35480099692714e-07

AC010624.1 0.077204059960199 0.162487991394464 1.07358447814942 5.68418653845028e-05 0.000148186542585659

AC005532.2 0.0544876399502488 0.125698947854671 1.20597166470777 6.09151357222002e-06 1.92359884771063e-05

AC093525.9 0.0448129546517413 0.101848155740484 1.18443210039065 1.2201026811802e-15 2.10659058451643e-14

RN7SKP18 0.102924312189055 0.364472813737024 1.8242273993967 2.50613348971698e-15 4.13157027991316e-14

DNAH6 0.158954686452736 0.436183294813149 1.4563189659058 2.27778036392727e-12 2.31588449640932e-11

AC090515.5 0.0469295079104478 0.112566711072664 1.26221300988162 0.00262414303150812 0.00488865281055426

CELF2-AS1 0.108091326104478 0.303176259076125 1.48790602710813 2.03717550473288e-16 4.00839928449239e-15

TRPA1 0.276088135562189 0.0983882320311419 -1.48857121791687 6.58543982527026e-09 3.64082836509282e-08

MELTF 4.94553660497512 2.19515499515571 -1.17180425710758 1.12171628581052e-11 1.01350975939603e-10

SNCG 32.9997540970149 15.7067445340138 -1.07107108330431 7.51035723042988e-08 3.43779531802941e-07

H1-7 0.0276599760746269 0.0562560947612457 1.02420949717663 7.93612254404977e-07 2.97618197696094e-06

MTCO1P28 0.0418286038308458 0.0919623524602076 1.13655352763214 0.00105413254222426 0.00213164695229823

ZBTB20-AS4 0.060147001641791 0.130803552560554 1.12083699902534 0.00222450479700547 0.00420109143751551

AC011451.2 0.114377969402985 0.237096712560554 1.05166646127328 4.30615584255692e-09 2.44952916584717e-08

AC011383.1 0.0226242794029851 0.0609207141522491 1.42906101191837 4.74789995325932e-10 3.22221001790676e-09

CNR1 0.570215877562189 1.20368815120415 1.0778815538074 9.45854068900378e-11 7.29323619521436e-10

SVOP 0.0496984695522388 0.0200324383944637 -1.31086338516307 0.00167571816273768 0.00324443520818884

AC016745.2 0.0129902720547264 0.0259899047024221 1.0005196984637 0.000236823957407754 0.000547378960120179

DUSP13 1.87722736309453 0.68852750299308 -1.44701720625947 2.67167636208436e-11 2.26856095942276e-10

AC107909.2 0.047780294278607 0.119993279238754 1.32846595901052 0.004492374829816 0.00794891968658003

DNAAF6 0.198676817572139 0.466915394100346 1.23273761189051 0.00245731424572894 0.00460913297613455

TSKU 28.36625639801 13.8344412179931 -1.03591139152054 1.3387993490654e-13 1.68582698880933e-12

KRT6A 57.1607896027861 17.9761451594602 -1.66894216594019 0.00122158538345551 0.00243302943762804

OR7E91P 0.260887454975124 0.126392385847751 -1.04551801739547 7.34933824123843e-06 2.28908325814681e-05

LINC02313 0.867075730099502 0.338600967820069 -1.35657190656288 0.00141916911756931 0.0027929967162352

LINC01116 2.12562442472637 0.737767414048443 -1.52664873635795 5.2743322022347e-10 3.54778617144907e-09

PITX3 0.090320412880597 0.0319396614775087 -1.49970305912457 1.84920458358193e-10 1.35048719254915e-09

TMPO-AS1 1.18136841641791 0.580287186401384 -1.02561996998923 5.51872826413366e-22 2.43447125845251e-20

C1QTNF9B 0.0445861923134328 0.148307278290657 1.73392049783294 6.2644947949422e-09 3.47462852649907e-08

DEPDC1-AS1 0.0625360073034826 0.0223458859134948 -1.48467786916246 3.11303204731249e-22 1.4096262664569e-20

AC245100.3 0.462397575721393 0.185389962456747 -1.31857069959391 1.44383545114696e-07 6.26509592081062e-07

RN7SL40P 0.0652849797512438 0.385185632110727 2.56073087865336 6.85629790365222e-06 2.14725463946969e-05

RAD51 3.79785352089552 1.52282301972318 -1.31843597972452 3.10558259471668e-44 3.14584014687042e-41

AC008080.1 0.0378986961144279 0.106710088636678 1.49347645941509 2.92284874965055e-15 4.76684038777236e-14

AL109947.1 0.0899746404975124 0.197665793771626 1.13547289525693 6.8732311468894e-10 4.52316823550108e-09

SLC2A3P2 0.0244015562437811 0.0786606327058823 1.68866862998807 7.1251304454053e-07 2.69868879215946e-06

MAGEA10 2.57204640860696 0.906270267519031 -1.50490341467765 0.000553178854718755 0.00118244952532692

CLDN2 7.57654578233831 24.5032294546263 1.69335973946828 6.56437243641329e-13 7.31902104100707e-12

AC079584.2 0.0540706468159204 0.110357829204152 1.02927146375843 5.93874649410852e-06 1.87926317961203e-05

AC022164.1 0.0907651512139303 0.241091510124567 1.40937045427418 3.64056530844599e-20 1.22021398512252e-18

AC026348.1 0.066219373880597 0.190585462491349 1.52511280242088 1.56371393920532e-11 1.3791543449618e-10

AL133330.1 0.28542698721393 0.578253648131488 1.01858071413125 1.99479817498122e-10 1.44753860667913e-09

MIR3677 0.81746997761194 2.39941717958478 1.55344636426614 3.62385067153267e-17 7.97363764009803e-16

AC025809.1 0.022235703880597 0.0516996612802768 1.21727675395944 1.85634331750031e-05 5.34196580624583e-05

AC005480.1 0.0264369927761194 0.0576555392249135 1.12490114193852 0.00129967226899074 0.00257633236238548

CHRDL1 3.39921007283582 6.91241871916609 1.0239910873857 3.01191002885178e-16 5.71656761201223e-15

NCAPH 7.69359080248756 2.66365581868512 -1.5302494211137 2.92595017939253e-47 7.27497612785325e-44

AL512631.1 0.0802065587064677 0.161157912802768 1.00668290633047 0.000674074250184943 0.00141814851865832

ETF1P1 0.0314058106965174 0.072645625017301 1.20984440520504 9.59778047207333e-05 0.000239397442691478

CENPK 2.52053831840796 1.19187609273356 -1.0804976258144 8.42508665489773e-31 1.04739145459751e-28

FENDRR 0.321341371253731 0.894553383460208 1.47706084601124 2.15405913985441e-17 4.88503461650233e-16

RNU6-892P 0.141075609950249 0.373947375086505 1.40636666943878 1.33313915325194e-12 1.40831810897801e-11

SPAG5 9.66161480597015 4.05481269377163 -1.25262906307465 2.5703678313478e-35 5.49215313963768e-33

AC073862.3 0.0323617336716418 0.219205837304498 2.7599254142995 1.46592584461213e-22 6.99704569810502e-21

KIFC1 13.2131700393035 5.24846872318339 -1.33200815937861 1.94769853794491e-37 5.32695550127933e-35

CCDC33 0.175175617402985 0.438499535380623 1.32377333304912 6.80722572421807e-07 2.58795695798393e-06

DNMT3B 2.52735182885572 1.13675271799308 -1.1527080600824 5.2255881516953e-14 7.00587431121894e-13

RRM2 19.1755629751244 6.59752420380623 -1.53927229104335 3.90510445212909e-48 1.25631344758912e-44

AC079061.1 0.0248377828358209 0.0507611096470588 1.03118721109798 0.000129845118903561 0.000315976866448296

HOXA1 1.54917248381592 0.418949384570934 -1.88664992159947 5.61098722507413e-12 5.3489195052554e-11

AC027277.2 0.23843366721393 0.495646161695502 1.05572259721351 1.69972150192841e-12 1.77162283070663e-11

FAM153B 0.0424492595776119 0.111979374100346 1.39942172649671 9.57287776737891e-06 2.92240436363225e-05

DLX6 0.562707764268657 0.177677414096886 -1.66312556862788 2.81336059656977e-07 1.15585717765034e-06

AL158163.1 0.213371686318408 0.439748479619377 1.04330984150847 1.06009996041579e-17 2.49730696962721e-16

RNU6-757P 0.172249788756219 0.351625395017301 1.02953705463237 0.0011393437279799 0.00228585083900695

AC092506.1 0.121162603726368 0.0509152710103806 -1.2507741527581 1.52374408866632e-05 4.46479546014826e-05

AC009090.4 0.0987889854776119 0.222115905311419 1.16889060336572 3.57772579137679e-18 9.13931994615523e-17

AC015849.5 0.16198292920398 0.0697202909653979 -1.21619128527642 3.5624340454096e-10 2.46228382971829e-09

AL353795.3 0.0223982350149254 0.0547745845743945 1.29012158533366 2.94785597127278e-07 1.20622173570183e-06

KIF20A 8.14558600945274 3.50941356989619 -1.21478851938735 8.75885708607535e-37 2.23651497996192e-34

C4BPA 54.7340281508955 143.547498370242 1.39101824793206 1.52137495477479e-14 2.22273531052834e-13

CCDC60 0.198108158348259 0.486962603951557 1.29752269203641 3.04193726730001e-10 2.13216258997066e-09

TYMS 15.295844960199 7.29761625605536 -1.06764261109383 5.55927211554832e-32 7.83742744124982e-30

AC006020.1 0.0163723509850746 0.0401175865778547 1.29297331764517 2.41041664174094e-07 1.00266000230593e-06

NFIX 7.54095387462687 17.4503696782007 1.21043866925045 2.07516728864098e-26 1.57655070400919e-24

HLA-DQB1-AS1 2.99576510686567 6.22052015124567 1.05411071272487 2.32044682081854e-12 2.35576171304332e-11

RPL7AP15 0.056968518358209 0.142093784429066 1.31860665845136 6.13207907154557e-09 3.40601873693687e-08

LCN10 0.0171759388258706 0.0550521117716263 1.68040894792517 2.4742152108843e-11 2.10940729481564e-10

UNC45B 0.0333337793482587 0.0684422833079585 1.03790299254098 3.6520973838211e-16 6.85551567930728e-15

AC007787.2 0.0474232793532338 0.188701089134948 1.99243541507093 2.32233692466267e-17 5.24924916442347e-16

UCHL1 54.7362357731343 19.981587232872 -1.4538250357192 3.54826633246588e-15 5.71862605733305e-14

AC008985.1 0.0256244853432836 0.0632038452041522 1.30248930294748 0.00043574020891322 0.000952561323137764

ABCC12 0.0164041742736318 0.0423440262456747 1.36809547762521 1.28033798367465e-07 5.61083862417909e-07

CFAP43 0.523863811970149 1.34779266124567 1.36333486493536 5.16467539077782e-06 1.65402660348681e-05

LINC00342 5.30344989089552 12.4374781048443 1.22969094351633 2.23588936820112e-12 2.27752604172442e-11

AL109983.1 0.0338357908457711 0.0722320755709343 1.09408951733117 2.43795666939075e-05 6.85495167141328e-05

MIR3189 2.86549761741294 8.31810398581315 1.53746902374436 1.32861043682332e-13 1.67376763920395e-12

AL121748.1 0.158815433283582 0.333903725640138 1.07208107407136 4.87767059820471e-13 5.56546895539837e-12

PLCH1-AS1 0.0269562329154229 0.066982116100346 1.31315705781995 1.09415628669548e-07 4.86113944787547e-07

AC009139.1 0.207067307363184 0.0629385578200692 -1.7180837668401 0.000113726790956959 0.000279739880625311

AC129926.1 0.7917358760199 0.349703331868512 -1.17888768420784 7.10810991438868e-05 0.000181977727378574

LRP2BP 0.356101684776119 0.852647573356401 1.25966029117625 2.60081306039715e-28 2.41946385040347e-26

LBP 9.62166913740796 1.41929917218685 -2.76110846475583 0.000676609186109282 0.00142304377423015

AC027288.1 0.491600021283582 1.05215029749481 1.09778392207111 2.15889301621533e-10 1.55711297451185e-09

AC135012.3 0.0130823547562189 0.0330922036955017 1.338869125789 2.46141463967341e-11 2.09914843763853e-10

AL355076.3 0.110452530845771 0.238247699342561 1.10903580654921 5.62415183895989e-11 4.5321317853728e-10

RPL26P27 0.172733502587065 0.369083072975779 1.09539764525884 3.02574381493367e-07 1.23476713426493e-06

RPL3P13 0.126465274875622 0.323626857612457 1.35559004040082 9.40397048525588e-13 1.02377978676681e-11

GGT4P 0.16069966360199 0.330650329647059 1.0409394309186 0.00315817611753285 0.0057738045999013

LINC01750 0.0378487568557214 0.0907704688719723 1.26197709401306 1.77737132098104e-22 8.32381945699168e-21

PGBD4P3 0.0758319166666667 0.184256873564014 1.28084134608445 4.06351021799276e-13 4.70520764022447e-12

GBX2 0.204767687318408 0.0455448920519031 -2.16862690646015 4.30993409807315e-13 4.96532003295285e-12

CENPN 4.66677634328358 2.16419522076125 -1.10859568431922 4.83651792760132e-43 3.48102013999727e-40

PLCXD3 0.2780779489801 0.57904135849827 1.05817705114071 4.80213765414522e-17 1.03989283326106e-15

AC116407.1 0.79207624721393 1.85739482449827 1.22956930057966 1.78648677846392e-17 4.0921619255434e-16

CCDC170 0.93015408960199 1.99215129723183 1.09878558101323 1.51353403937047e-08 7.82961149551397e-08

AC025809.2 0.0231891346318408 0.0550240559480969 1.24661350515916 1.39021365696814e-08 7.22858241788567e-08

PTGR1 27.4614013701493 12.3865451176471 -1.14863140495134 2.29005559926748e-06 7.83010634328861e-06

BTNL9 0.496794590696517 1.75550986595156 1.82116873407558 5.78446195020555e-26 4.15236310598745e-24

HOXD8 1.4376336759403 0.693035431384083 -1.05269509182125 0.00265172514631152 0.00493298073402395

PKP2 4.52632318835821 1.92924132719723 -1.23030597933869 1.89693334864924e-09 1.15137876354986e-08

UBE2SP1 1.05005618636816 0.397901378408304 -1.39998372369932 1.56054162442086e-21 6.37027066088217e-20

AC016769.1 0.156751087114428 0.0552151509031142 -1.50533935012267 2.30489960637989e-12 2.34258655646563e-11

HOXD13 0.971551158756219 0.094966092266436 -3.35480557062792 8.32443277891089e-10 5.40534749532794e-09

NXF3 0.12012187021393 0.56668009515917 2.23803568621817 5.428033203983e-10 3.64579342163396e-09

ZDHHC11B 1.04435992785572 3.14303417779931 1.58953895275013 2.24737235457934e-25 1.50282723466369e-23

AC026785.3 3.17565053099502 1.02919158775087 -1.62554058762458 1.93476877229066e-08 9.82471702973441e-08

AC068594.1 0.219845074875622 0.478944552906574 1.12337143313894 3.32871493284055e-12 3.29856352946337e-11

MAB21L2 0.00948486272139304 0.054105867982699 2.51208627004445 0.000774264610259935 0.0016085178192639

AP000526.1 0.084055562960199 0.0327967812283737 -1.35778907147935 5.40038408776736e-14 7.23312952009977e-13

AC103681.2 0.0289551442288557 0.0682514594117647 1.23704021224297 1.49477437210806e-10 1.10911771777416e-09

VTA1P1 0.0138360648756219 0.0495850178200692 1.841470590744 2.26908681881196e-14 3.21052894436146e-13

AL162419.1 0.0284726579104478 0.056980176816609 1.0008829241997 6.86946199989109e-05 0.000176263988832931

BX324167.1 0.0593594854726368 0.156351935813149 1.39724658970292 9.87377491505347e-07 3.63504837699169e-06

DUOX1 2.41333020049751 7.01502956851211 1.53942386107983 1.96453976733622e-17 4.47378539855501e-16

SNORD89 1.47337203482587 3.04172108823529 1.0457661055759 0.00227918024351674 0.00429836128228007

LINC02718 0.0555479006467662 0.14083266982699 1.34217775053886 1.654942041972e-16 3.30625747610915e-15

AL139095.3 0.126677113890547 0.0311241043217993 -2.02505167811458 1.08462731416182e-11 9.83899072713956e-11

MRPL15P1 0.0260625371641791 0.0703683250519031 1.43294863776524 8.30947072642076e-07 3.10046417963994e-06

RNA5SP515 0.196758940597015 0.441442871107266 1.16579755266423 1.50575876995032e-06 5.35676198257455e-06

TTC34 0.102870925537313 0.225177112093426 1.1302249033491 2.42242357606249e-13 2.92122067042809e-12

TRPV6 0.0890827564079602 0.178800028342561 1.00512886126091 7.92437694447413e-06 2.4544927455421e-05

SPC24 5.0983583880597 2.18885867301038 -1.21985398315126 9.6441206016402e-39 3.21666705432755e-36

GAL 5.60846385109453 1.03145673280623 -2.44292236835546 1.19089397579218e-11 1.06965353819101e-10

AC243562.3 0.0321039652437811 0.0697026165813149 1.11846131539673 0.00045891547354411 0.000998038979121454

BRD9P2 0.259971810646766 0.823190240103806 1.66287068054619 7.0581426525969e-18 1.71946789082372e-16

HOXA11 0.569824483880597 0.0878278266712803 -2.69776760251722 1.15420625784541e-10 8.76632634048097e-10

SNX18P2 0.0483621204477612 0.101722030415225 1.07268275787157 0.000371399063810776 0.000822690888088177

PAK3 0.301328804223881 0.843161333321799 1.48447011997885 1.66733748047461e-13 2.06810340548665e-12

BNIP3P40 0.122131567960199 0.387751130865052 1.66669483968169 3.95769992743533e-11 3.27315068083932e-10

FOXD3-AS1 1.58986237289055 0.764938418612457 -1.05548637001724 2.21082853519024e-05 6.26917163685361e-05

BPIFB2 8.6655560379403 19.2983345925571 1.15511212110336 1.8377821595345e-05 5.29422183097415e-05

MKI67 10.0126659721393 4.2560832283737 -1.23422788890555 9.17288271022112e-26 6.39995770725887e-24

MIR6503 0.220444125373134 0.47247614982699 1.09982847420641 2.05161386407527e-07 8.64586119914617e-07

AC087286.2 0.214341646069652 0.520291143114187 1.27940695916098 0.000215852889380471 0.000503074267111707

PACRG 0.32239687118408 0.699442598512111 1.11736792112603 2.12043572663456e-06 7.30126112595433e-06

SP6 2.29749294930348 1.12730383408305 -1.02718402696078 3.97351663979007e-08 1.90893518528471e-07

MYH16 0.253719695437811 0.0928669833875432 -1.44999783798278 6.93728461509842e-08 3.19534890968838e-07

NOC2LP2 0.0967747397164179 0.0463147747577855 -1.0631580249134 0.00140319394851655 0.00276493908443283

LINC02257 1.63156006074627 3.4176127815917 1.06673685102685 4.82603491835691e-12 4.6460871009892e-11

CCDC58P1 0.0444470148258706 0.0183037004152249 -1.27995118306132 2.08333748698453e-07 8.76786250763234e-07

MLXP1 1.63133098169154 0.396982944463668 -2.03890058952603 0.00012943111193732 0.000315025443755958

CTSG 0.566777539716418 1.39302191249135 1.29736345865337 4.35223986237335e-11 3.56922819298084e-10

LY6K 4.86155664545274 1.91375124013495 -1.34501501873047 2.23319467857954e-10 1.60141254481254e-09

CLDN18 11.0408199746269 33.4140904422491 1.59760928191282 1.30392430095856e-10 9.81622065268831e-10

RPL18P10 0.0866518737810945 0.19548117266436 1.17372681222784 3.09745289865923e-05 8.50214138682557e-05

AC067817.2 0.266398281741294 0.610003917266436 1.19523373015867 1.3585552817307e-15 2.3339501856366e-14

AC104115.2 0.0721448081094527 0.182867324117647 1.34182982807441 0.00206464888259964 0.00392630697671396

AL391840.2 0.0714365762686567 0.208670277820069 1.5464902799061 6.59326131754054e-05 0.00016960656229753

ECRG4 0.596888923651741 1.92550724802768 1.68970416641704 6.19488557946023e-16 1.1139389914414e-14

GFRA1 0.258003403323383 0.856263274266436 1.73066435222255 2.06706366052497e-24 1.23168172364614e-22

CNGA4 0.233556116223881 0.60098600516609 1.3635621700307 3.98244238581777e-09 2.279610700128e-08

ACADL 0.492513279890547 1.05270713471626 1.09586960112456 1.08974357024462e-08 5.79065215585591e-08

FAM72D 0.473892741741294 0.171744671799308 -1.4642952228477 1.48631335351676e-34 2.92450864882614e-32

A4GNT 0.0522530121144279 0.514289881598616 3.29899566108149 1.1087235859147e-07 4.92266072642322e-07

NR4A1AS 0.753385935721393 1.9464963400692 1.36941862575304 4.92263349701759e-17 1.06430060192436e-15

AC004832.1 0.146321305671642 0.398192375034602 1.44432574134513 8.50868007415867e-10 5.51451184901042e-09

C18orf63 0.0133240680348259 0.0283285563287197 1.08822245562733 6.602051413836e-10 4.36044690095181e-09

FAM72C 0.304307210835821 0.100658820868512 -1.59605491711201 7.36598544615773e-31 9.19907314851205e-29

AC009336.1 0.0849018899303483 0.234862072546713 1.46794518005212 1.58759535397141e-14 2.31207310602333e-13

NDC80 6.0040837159204 2.08675200103806 -1.52468493849202 8.42441372669769e-44 7.43250694919941e-41

AC018816.2 0.0492801685074627 0.158311193356401 1.68368417022964 2.35456302609959e-10 1.68227008264952e-09

AC006159.1 0.0549864356716418 0.11581816733564 1.0747138970149 0.00133783776775925 0.00264492286744365

ITGB5-AS1 0.0424295204477612 0.103821180276817 1.29096051599097 0.000145311354749576 0.000350155555277613

MAFA-AS1 0.421732426318408 1.19912103733564 1.50757743194022 5.39186127849813e-05 0.000141279369579348

AC092068.1 0.0224453758706468 0.0794442604152249 1.82352473754732 4.17543529341986e-11 3.43556423811772e-10

BPIFB1 28.596120800393 92.239191399654 1.68956041404391 6.89144580422079e-06 2.15629858025246e-05

AP000851.1 0.171842635940298 0.0661285793183391 -1.37774221579257 4.77199529490334e-05 0.000126467123367836

AC020910.2 0.0560966156716418 0.126254559965398 1.17034985468088 0.00177844180015535 0.00342513789410949

HAGLR 5.49357894363184 13.4175944565744 1.28830780022892 7.47122255265986e-18 1.80510544889794e-16

AC012370.1 0.0407350428855721 0.163070973183391 2.0011576713995 0.000680953766791017 0.00143152079336928

ASH1L-IT1 0.0491223879104478 0.170128063564014 1.79216854212762 0.00427559171374178 0.00760370852271524

ELN 9.81674157960199 22.9254779557093 1.22363566899113 1.32832335534053e-25 9.15104377041902e-24

SHCBP1 4.06959158358209 1.80638178719723 -1.17178117028742 2.27912617999677e-36 5.51629212592139e-34

AC005165.1 0.140388999482587 0.298423708207612 1.08793226051036 9.97394384637478e-14 1.28069185069648e-12

RPL26P30 0.474981730497512 1.44558976224913 1.60571426570805 1.06753781932533e-11 9.70351000815423e-11

CCDC81 0.15136869300995 0.325151263875433 1.10304418235145 1.46767825775566e-07 6.35846671148698e-07

FAM222A-AS1 0.542183850248756 0.241572621418685 -1.16632518460676 8.04769579217444e-05 0.00020398932336976

TACR3 0.0778312090348259 0.0352074385224913 -1.14446849884413 0.000486957151260691 0.00105349454888308

CCL16 0.0314609874925373 0.0761338940276817 1.27497491520304 2.034432158362e-09 1.22992306656058e-08

AL158163.2 0.159384143482587 0.338693318200692 1.0874714171114 5.93021891027546e-15 9.2680849826305e-14

PHEX-AS1 0.0371609820945274 0.0985328561245675 1.4068162465602 0.00310639788807138 0.00568750717892303

AC007849.1 0.151692485572139 0.329820642629758 1.12053207597383 8.34807063576406e-06 2.57668132138751e-05

VEGFD 1.04756333497512 3.34370388380623 1.67440961802781 4.71842481562002e-18 1.18068544105405e-16

SEPTIN9-DT 0.341233940671642 0.68792473583391 1.01148958104925 3.97114353439428e-11 3.2832761688538e-10

PKMYT1 3.7592258039801 1.77249701550173 -1.08465237751233 1.87894532148917e-28 1.79056287605327e-26

BTG2 37.1404244129353 75.1404770968858 1.01659997485029 1.01301852107409e-22 4.91242137435751e-21

CLCN3P1 0.117433771935323 0.045744806432526 -1.36016749882099 0.000846029860659532 0.0017444901001989

C1orf116 25.6209164079602 58.6990986359862 1.19601627109223 2.03087626388378e-23 1.06816280417733e-21

LTC4S 0.0190036537263682 0.0410836055640138 1.11228597633163 5.21836922300301e-12 4.99903321362985e-11

SYT5 0.503304988099503 0.17510875916263 -1.52318164499289 2.84125210874952e-05 7.87237819616041e-05

U62631.1 0.180127946318408 0.406116444532872 1.17287141780614 2.0465646367188e-07 8.62724149418298e-07

AL355864.2 0.235502924925373 0.49074495467128 1.05922845738587 1.69312679679663e-11 1.48160077539645e-10

AL161457.1 0.0162543236318408 0.0419653280622837 1.3683743346141 3.7561275406847e-07 1.50586467660109e-06

GINS4 2.25384470845771 0.908958713148789 -1.31010144547308 1.18234181578355e-30 1.43084286113629e-28

RN7SL494P 0.067655738358209 0.459413745605536 2.76350981302825 1.0893258839779e-08 5.78955750617869e-08

LINC01446 0.33520440180597 0.0954558888200692 -1.81213498665481 5.22183672417087e-07 2.03617385808488e-06

AL158801.1 0.0898576126865672 0.358097181764706 1.99463852631041 0.000698853098416956 0.00146621910415033

AL360081.1 0.0249063644776119 0.0546522142214533 1.13376549732164 0.00090024315898963 0.00184542425411231

PSME2P5 0.0379763510945274 0.0866631858131488 1.1903179795161 3.39421547179312e-10 2.35733349805845e-09

AC025917.1 0.420658192039801 0.846355845121107 1.00861592308789 2.22039913179274e-13 2.7002223682067e-12

RPL6P6 0.0351581605472637 0.106873505743945 1.60397275236718 0.000532375997308693 0.00114190914645069

AL137779.1 0.135644444577114 0.281965033944637 1.05568630515533 9.98430278559015e-16 1.74486058265745e-14

GKN2 1.77343179110448 14.6126289768201 3.04260001041005 2.44763012988331e-14 3.45421486338021e-13

LANCL1-AS1 0.189008502636816 0.422343570415225 1.15996595197448 3.6377221583836e-08 1.75750741073114e-07

AC130456.1 0.0725509839353234 0.189237323083045 1.38312957265818 2.26076516016878e-10 1.61990901573529e-09

AL078645.1 0.0395397623880597 0.247714153287197 2.64730019371769 4.59669365245771e-16 8.48310198344928e-15

FAM216B 0.859255965159204 2.76060284623529 1.68382348244544 2.09499514680712e-09 1.26374321272992e-08

AL121904.1 0.133228854084577 0.296832445038062 1.15574222480976 1.34174442689359e-12 1.41686139287798e-11

AL390334.1 0.123497996935323 0.0356011631557093 -1.79449135975289 2.22882216051632e-09 1.33621845879266e-08

RAD51AP1 6.69357512835821 2.49504993979239 -1.42370828733435 4.79333750256103e-43 3.48102013999727e-40

AL359885.1 0.0249683484079602 0.0588829065397924 1.23774849267302 2.87395069134209e-06 9.64566835295204e-06

SEC14L6 1.09186892059701 2.76828470750865 1.3421926558785 2.0537426706557e-16 4.03809216696142e-15

RN7SKP74 0.111362607164179 0.266174434429066 1.25710711733571 4.33663081038128e-05 0.000115747880027255

RNU6-1136P 0.315350308955224 0.661935321557093 1.06973490992012 6.69356299690939e-10 4.41662118131416e-09

CLDN6 20.1022486646269 9.31176476041177 -1.11023037468213 0.00363534002273262 0.00655458828015935

AC024267.5 0.087648992238806 0.182323719584775 1.056692853898 1.33887902160281e-06 4.80808052006786e-06

RPL12P9 0.0504004202487562 0.10988206266436 1.12444822913716 3.37959828620687e-07 1.36648972118919e-06

LINC02771 0.1443697639801 0.057552412733564 -1.32682030770707 2.78342988463998e-07 1.14459190114123e-06

VIPR1 1.13003558771144 3.10796491799308 1.459602011508 4.34577163955055e-32 6.1904611636306e-30

MCM10 3.09160063333333 1.04671146605536 -1.56249015963972 5.6564898381293e-42 3.03343131515365e-39

HSPE1P11 0.0952070424378109 0.280798699342561 1.56039605477133 0.000214431920443761 0.000500060796737454

DCDC2B 0.238787122686567 0.678725656955017 1.50710351338629 1.57644004498228e-11 1.38885838258633e-10

AL023584.2 0.0838864719900498 0.198266780207612 1.24093289553251 0.00518922885337878 0.009049633943755

GPR12 0.0298708282139303 0.135851296179931 2.18521918640032 2.98917534508631e-10 2.09733057178324e-09

PLK1 9.41208869353234 3.55662498200692 -1.4040060540359 3.79075811157312e-41 1.81889884827237e-38

AC011365.1 0.0276465589552239 0.060826935467128 1.13761039558986 8.65040228200921e-08 3.91378829467249e-07

AL445489.1 0.0307387506467662 0.12007884650519 1.96585158931416 5.34730805367566e-06 1.70672044892087e-05

TMEM190 1.49793989965174 4.7206801715917 1.65601500180882 4.29079842922748e-05 0.000114658853971052

FAM166B 1.06477706557214 2.40587636183391 1.17601110198808 0.000726420696241046 0.00151730609761666

PGM5P4-AS1 0.0362241952587065 0.0912297425536332 1.33255060603329 4.99864455267509e-13 5.69162899732155e-12

MAD2L1 6.11708440199005 2.09969467370242 -1.54266462796009 2.66507535700121e-46 4.77270339358322e-43

SCRG1 0.0312030308905473 0.0854026561695502 1.45259476949808 2.04461583237014e-08 1.0346020909403e-07

ATP1A2 0.177128188039801 0.521736003737024 1.55852617398058 6.47671714407631e-24 3.67506667822587e-22

AL359740.1 0.0725484471641791 0.159383348096886 1.13548426855559 1.17470842826487e-05 3.52090690553909e-05

SLC16A12-AS1 0.0249525852736318 0.0563005135640138 1.17395878513717 2.90470766799821e-05 8.02867657602335e-05

CATSPERD 0.13264386039801 0.267507217574394 1.01201991718537 0.00411245214657312 0.00733647943439925

AL355073.1 0.0432178719900497 0.0900789541522491 1.05956204041479 1.3370925599862e-05 3.96503106533911e-05

ADRB1 0.885995156318408 1.81261093733564 1.0326985783921 6.14431144912382e-09 3.41212016514795e-08

PLK4 3.17954833930348 1.47371320138408 -1.10936605301466 1.4881215374741e-33 2.64286519804654e-31

LINC00443 0.0381960274975124 0.14713118215917 1.94560852918482 1.80577164621817e-06 6.31316049138016e-06

CYP2U1-AS1 0.0207772974179104 0.144449210217993 2.79748240120163 5.99653358063566e-08 2.79062775957777e-07

CFAP91 0.428817103850746 0.901633103114187 1.0721780340268 1.80166489808359e-05 5.20058416491675e-05

DNAH7 0.104668423141294 0.286013869747405 1.45025884076764 5.0192923433964e-10 3.39040863403042e-09

CFAP57 0.526427186616915 1.10799027467128 1.0736393161245 6.04275745070247e-10 4.01626771024818e-09

STC2 5.33958813880597 2.18486441349481 -1.28918471313641 2.11297126285487e-14 3.00831671208124e-13

PENK 0.602129169567164 1.4015194549827 1.2188468568671 3.34220160264461e-12 3.30952982738342e-11

AP001533.1 0.0436063923383085 0.0924657828027682 1.08437995370261 4.2220946733939e-10 2.88830138362489e-09

LINC00525 0.50873584521393 0.233717974961938 -1.12214805489626 3.3131608096953e-12 3.28553111476311e-11

MIR553 0.29606448159204 0.737748843944637 1.31721833199512 0.000883453896583969 0.00181399880417204

RGS20 1.11789207355224 0.329897974072664 -1.76068908713455 1.30667459243877e-11 1.16522823942616e-10

CDCA4 12.2186481492537 6.08493214186851 -1.00577159717441 1.4052496246496e-32 2.19620441338095e-30

LRRC55 0.169804165736318 0.559290782304498 1.71972670102632 7.13757216899415e-07 2.70265262109913e-06

LINC00216 0.196375300845771 0.401161738512111 1.03057052727988 1.0425711918935e-05 3.15914802276107e-05

MEGF6 3.84056521825871 8.96393188235294 1.22281303687734 1.62624798935314e-26 1.24587906187138e-24

AC099677.1 1.6345099 4.07751967612457 1.31883372336214 2.2482956090161e-21 8.93762862014394e-20

AL020997.2 0.155172523134328 0.373238041591695 1.26622291989936 0.00186108880681161 0.00356982032611377

AC127024.3 0.129283905273632 0.275608187439446 1.09207606325249 3.2327098470132e-06 1.07468839571911e-05

AC133963.2 0.0493309432835821 0.141155651591695 1.51672211332653 2.25515987375409e-12 2.29459161261809e-11

AL138930.1 0.108382234427861 0.260648671764706 1.26597821274007 0.00268761491541458 0.00499498966679728

CC2D2B 0.0318764598208955 0.0734222697024221 1.20372629971207 1.75578410186589e-12 1.82588194623696e-11

RS1 0.0480514876766169 0.160803113778547 1.74264234029954 7.31045069311433e-15 1.12833423508283e-13

SYBU 2.66963880134328 5.80232067785467 1.11998547105993 6.20153036449315e-08 2.87965798758723e-07

LGALS17A 0.467716371368159 0.167274895920415 -1.48341298138255 3.34833845677329e-06 1.11029409302558e-05

AC024267.4 0.0770222266169154 0.165781604290657 1.10593719383145 1.82925560780462e-07 7.79407086360124e-07

AC007688.3 0.0281347744278607 0.0747160973702422 1.40906470448436 2.84093104909239e-06 9.54656151771432e-06

HLF 1.57667171079602 4.58794299951557 1.54096516910085 1.22665320668124e-23 6.65654071482776e-22

CCNYL7 0.0577542975621891 0.276344497958477 2.25846768046692 1.99265521895373e-07 8.41815264726359e-07

AC007878.1 0.205190501144279 0.666274102802768 1.6991518730317 6.17146159211471e-07 2.36897508132403e-06

SHISA6 0.0688122954179105 0.140714327117647 1.0320309524078 1.02576218925617e-15 1.7891407603128e-14

AC002519.1 0.0225947276268657 0.060300166467128 1.41617581906658 4.61748159978341e-16 8.5157196058042e-15

AL450322.2 0.0523348787562189 0.0255435809688581 -1.03481196453658 1.28835696141068e-05 3.83381165211425e-05

CENPW 16.1618588557214 5.61401530138408 -1.52548823802768 1.51656799150528e-50 1.36189787205277e-46

C11orf97 0.35784915358209 1.03146533719723 1.52727186732295 1.04798699893645e-05 3.17343273039326e-05

SYT14 0.263260443393035 0.0516532045740484 -2.3495610012454 4.30797621935565e-05 0.000115039200936709

AC023509.5 0.0249451452736318 0.101532868754325 2.02511586350901 9.03921116521069e-06 2.77248430378504e-05

SIDT1-AS1 0.0554483065671642 0.159560116366782 1.52488477550715 0.000238209568320799 0.000550395513523178

ADRA1A 0.0182162483930348 0.082920501017301 2.18650296442391 5.3818522302111e-15 8.48378435137024e-14

AC110285.3 0.242543972039801 0.520484466574395 1.10160878354467 7.66988598303679e-11 6.00891955416947e-10

NEK2P4 0.0306212218756219 0.0137778724359862 -1.15217872585686 6.29473287321795e-14 8.32499729605952e-13

AL133230.1 0.157025745527363 0.335320675892734 1.09454032354932 1.34082454264594e-08 6.99707603296777e-08

LARGE-AS1 0.00614379821890547 0.0467850781937716 2.92884572522998 0.000203072554937884 0.000475557357440802

C19orf84 0.393433627850746 0.178118099352941 -1.14328615159547 0.00183507303200341 0.00352353604502199

SCN1A 0.183794029044776 0.451758265913495 1.2974611003665 2.26125164079544e-13 2.74867699447801e-12

AKR1C4 1.84084603808955 0.76386803800346 -1.26897363760956 0.000174948404849411 0.000415314544972779

ZSCAN4 0.0803658054427861 0.301240835560554 1.90626366195422 6.27358489726554e-14 8.30104242574807e-13

AP005131.1 0.0197869680199005 0.133672748913495 2.75608291295673 7.0343409458765e-09 3.871010560759e-08

AC079075.1 0.0587422313432836 0.151366047716263 1.36557166366426 2.90387107934384e-10 2.04219269786716e-09

MMP20 0.0407656145024876 0.0103854978754325 -1.97278238252371 2.03738588809321e-06 7.04367387679804e-06

AC009652.1 0.0779853260696517 0.295859285916955 1.92363658338839 2.35618534219032e-08 1.17686293054685e-07

YBX2 2.01784697761194 1.00025023270588 -1.01245580823259 8.36589386982004e-08 3.79385172176385e-07

AC005901.1 0.0325456681094527 0.111729442179931 1.77947196925087 2.3936288010396e-13 2.88777007977208e-12

RPSAP52 0.470216018741294 0.182868533041522 -1.36251684195671 0.000369557102367977 0.000819008731039962

LINC01571 0.0787187488059701 0.213024551280277 1.43624051692004 0.000236431996131 0.000546611588688322

KIAA0408 0.0241690667164179 0.0645550693010381 1.41736862535729 4.07588536228294e-12 3.9628675669548e-11

AC005072.1 0.0231551927462687 0.0904472391937716 1.96574070054231 0.00534693314732479 0.00930093630855009

FO681492.1 0.0995749405373134 0.202172973311419 1.02173553101601 8.04856678276813e-13 8.85471848385794e-12

EIF4EBP1 69.4860107562189 28.961807615917 -1.26257090777449 1.03998555607095e-26 8.08056959049446e-25

AC021231.1 0.0107866616865672 0.0437893262214533 2.021330810502 1.6282784119653e-08 8.37094258782913e-08

CA3 0.822464217049751 1.68262135283737 1.03268573963446 1.6153061564303e-17 3.72187223069661e-16

HAS3 4.57731633726368 12.5898070152249 1.45968226384149 7.4634091749549e-11 5.85554334294367e-10

SCARNA7 2.03792718144279 11.1013742946367 2.44556387844133 4.16289133671473e-05 0.000111611683226299

DLEC1 0.33368043960199 1.15254836484429 1.78828826919995 1.71343784456896e-23 9.0643181912884e-22

NSA2P5 0.0900604474626866 0.189234488754325 1.07120949887481 7.04954869743362e-08 3.24369375630568e-07

NCAPGP2 0.0431436774129353 0.0864425735986159 1.00259287334872 0.000196759466269167 0.000461880645649448

EPB42 0.0266035007910448 0.0534989176782007 1.00789360061318 2.12311673538406e-14 3.01961740575944e-13

RUNDC3A 0.963319809845771 0.423542458512111 -1.18550823240729 0.00397730302809001 0.00711486937132983

FRMPD2B 0.0364007102587065 0.0828665162525952 1.18682267098676 5.09637080620306e-06 1.63463986806208e-05

AC006504.1 0.130789544358209 0.284103294124567 1.11916834659122 2.15123735404938e-05 6.10911749676728e-05

AC244517.3 0.0314823625870647 0.0668256223252595 1.0858575562359 1.28191335068873e-05 3.8175446582466e-05

LINC01359 0.0596351816567164 0.142114862861592 1.2528218424616 3.42375485105118e-09 1.98768191840904e-08

SKA3 4.0568657960199 1.48968189238754 -1.44536128839928 4.59011586573305e-44 4.32895410095858e-41

RPS10-NUDT3 0.10363687 0.0443736666089965 -1.22376167607253 7.82941882076524e-09 4.27670470836687e-08

AC009090.5 0.0531275997512438 0.115683147193772 1.12264526691494 9.79128343528818e-16 1.7133179907558e-14

ANLN 13.7309520278607 4.90899522560554 -1.48393198872491 1.02318352952526e-37 2.88495562190885e-35

AC103702.1 0.0252013420696517 0.0537325662906574 1.09229617852826 0.00122976935266441 0.00244790333299648

INSYN1 0.157866274935323 0.544875793979239 1.78722440051088 9.92518288211299e-29 9.66027586568649e-27

ZFHX4-AS1 0.129593535905473 0.0339813628200692 -1.93117814076007 0.00434731923936223 0.00771621657450561

GAS2L2 0.393959954552239 1.3126268526955 1.73633595761852 3.19256475436738e-13 3.78321689913119e-12

ST3GAL5-AS1 0.445242831293532 1.05335769301038 1.24233113318428 1.51723473045481e-22 7.21823300029144e-21

TRIM63 0.0784007297711443 0.159723702107266 1.02663942810282 3.58935803835076e-07 1.44451062903021e-06

PWAR5 0.219995356970149 0.573623223941176 1.38263035894567 6.30733084696088e-07 2.41521896364167e-06

ANKDD1B 0.572832991487562 1.35015810349481 1.23694186669546 1.61420691517041e-15 2.74555715982032e-14

ODAPH 0.30355302818408 0.0789129511418685 -1.94361456634807 1.84028866915482e-05 5.29976783209269e-05

ARMC2-AS1 0.178801236318408 0.541687044186851 1.59910287347916 1.81937926942984e-08 9.28358638412428e-08

RN7SL600P 0.377359047462687 0.83212625065744 1.14086456716178 6.91227127115967e-07 2.62497388595136e-06

AK3P5 0.074947944079602 0.230690008408305 1.62199471089228 0.00466371692543611 0.00822348518037893

HASPIN 1.52161442905473 0.736435372560554 -1.04697200287723 2.19991002936909e-29 2.27907345845623e-27

LINC00668 0.356581333791045 0.0976583608961938 -1.86841571417661 0.000310656895978548 0.000699700741580605

AC108448.2 0.130617895621891 0.267705545709343 1.03529445537184 7.24403213912265e-09 3.97680206754325e-08

RNU7-45P 1.24395414079602 3.21834941314879 1.37138766607112 1.86954336023439e-05 5.37383193929696e-05

AC079210.1 0.526994922189055 1.17589950584775 1.15790380445267 6.17580583905046e-17 1.31651044191762e-15

RN7SL798P 0.0811720519402985 0.163775664982699 1.01266601761518 0.000152258215079085 0.000365382309591381

AZU1 0.21990689858209 0.741695665802768 1.75393447553926 6.78965928569031e-12 6.39893802424638e-11

GLRA3 0.128734582830846 0.264223529190311 1.03735927833352 1.98122424057992e-05 5.66567157882275e-05

CDHR3 1.02944735726368 2.66608994145329 1.37285539413827 1.18961332441637e-09 7.51233535506528e-09

AC089983.1 1.30964159298507 0.441421879065744 -1.56894200263935 5.54983600122626e-12 5.29431512499261e-11

AC099850.3 10.5323355487562 3.75700131107266 -1.48717186537721 9.08541579740588e-41 4.00784067837179e-38

CFAP221 0.911260182139303 2.34796077861592 1.36547337435757 1.88254591439782e-24 1.13408878323305e-22

AC112777.1 0.714077840945274 0.32716858799308 -1.12604711028983 3.15434064698655e-21 1.23244595278689e-19

AC005514.1 0.0516732746766169 0.116897662768166 1.17775586586101 1.82088125401212e-05 5.2532808330413e-05

TTN 0.0756939387910448 0.164027389598616 1.11568705303733 7.99486847082616e-07 2.99287780833692e-06

DIO3OS 0.18972944700995 0.627639282795848 1.72599204077819 4.83989496312795e-19 1.4052136649846e-17

FXYD1 0.22826996318408 0.457242161176471 1.00221740147622 1.63584830443727e-16 3.27291630407226e-15

ASPM 3.23255532910448 1.39815028740484 -1.20915561914577 9.84162322087075e-30 1.05971809090872e-27

AL157400.2 0.114562874527363 0.0539447683044983 -1.08658464169316 2.11635414196643e-09 1.27465945348562e-08

PRR20G 1.17044366656716 0.277030364602076 -2.07893947900249 3.23384398216515e-10 2.25569071441512e-09

KIF18B 4.92035378069652 1.90509630449827 -1.36889812197592 6.1566936993642e-33 1.02051862228855e-30

TCTEX1D1 0.299069880422886 0.608117432629758 1.02386732445835 9.88620056233375e-09 5.29548737524144e-08

EFCAB12 0.187669071542289 0.393559984899654 1.06839263347894 1.66874281756426e-09 1.02515983963123e-08

SCGB3A1 147.388412970697 804.791317074394 2.44899162938606 5.51188158483493e-17 1.1851412055443e-15

NHLRC4 0.575280605970149 1.19155572183391 1.05050867960944 1.95998471630215e-10 1.42416530262656e-09

VWA3B 0.305047934318408 0.755892036176471 1.30914422821338 1.76019693776039e-06 6.16880910401674e-06

AC108156.1 0.0628054617412935 0.146960953010381 1.22647095661887 6.18453728210706e-11 4.93284032270715e-10

KIF12 2.94887922747264 6.294222216609 1.09386137621859 3.09116719421792e-12 3.08665289382476e-11

LINC00898 0.0483203921691542 0.0189939356747405 -1.34709329080329 2.28608185081768e-06 7.81847425532869e-06

CCL26 1.29768175179104 0.586114227889273 -1.14668285070406 6.04548414347087e-11 4.83886424711526e-10

RNF216-IT1 0.0697039614925373 0.139735154221453 1.00338245965421 0.00188700471506905 0.0036161420233421

AC012557.2 0.107428485223881 0.260575748546713 1.2783262381899 2.33279472173313e-16 4.52495997442561e-15

AC078925.1 0.0629106402487562 0.136747764186851 1.12014129546686 1.43325235211765e-09 8.91099155044731e-09

ANKFN1 0.207856152641791 0.463626079134948 1.15737626768635 1.60799868460962e-14 2.34008256958066e-13

AC011944.1 0.295498544079602 0.762725837854671 1.36801355059146 9.41537229273786e-08 4.22772011502841e-07

LINC00844 0.0381578167661692 0.0783226567474049 1.0374510741679 1.55653211457034e-08 8.0353252800111e-08

HROB 3.52032319701493 1.70409086297578 -1.04670562435912 4.58145711167387e-29 4.55646734561019e-27

MROH9 0.0546700141094527 0.164775762470588 1.59168239332637 6.17501730644709e-06 1.9463722868656e-05

AP005119.2 0.21625352220398 0.0437548287577855 -2.3052094848274 5.08299696492969e-05 0.000133943508036253

AC084375.1 0.953157243532338 3.1209395366782 1.71119426549646 2.34910813781352e-16 4.55014926127477e-15

HOXA10-AS 0.129651353686567 0.0337123075674741 -1.94328998283369 4.62665825880247e-08 2.19457341967131e-07

CLEC4GP1 0.0734527128855721 0.158454526885813 1.10918119560487 4.92460605169127e-06 1.58344669073309e-05

ELFN1-AS1 1.34328475706468 0.599817487681661 -1.16316967808892 3.99261088400806e-05 0.000107383132734409

AC145285.4 0.0653134296517413 0.169098498477509 1.37241227706101 6.72261638135989e-06 2.10876887292342e-05

GABRA3 1.35156185598507 0.29507256849827 -2.19548582861842 1.00240786363124e-10 7.70108288491978e-10

AHCYL2 16.0737801189055 33.6034760311419 1.06390122503993 4.6819884400344e-20 1.53172707936532e-18

CA10 0.413064526756219 0.875563601349481 1.08384481106738 1.6426540369247e-08 8.44167378990803e-08

CYP3A5 0.985130625223881 2.18882362958478 1.15176877093426 0.00482733235780524 0.00848233472444416

GSG1L 0.00749981175621891 0.0244502134636678 1.70492077092776 3.85549779867803e-09 2.21203827971144e-08

AL354893.2 0.0734968550746269 0.166978496608997 1.18390790169523 1.10040349526215e-11 9.97217879238562e-11

MIR3671 0.581624208457711 1.40247192145329 1.26981266357102 3.01144211448078e-10 2.11132893696615e-09

AC003991.1 0.0775894852039801 0.3185995766609 2.03781129070817 4.91887370467334e-17 1.06430060192436e-15

LINC00337 0.698947034278607 0.261191832951557 -1.42007334647695 4.43061054081738e-25 2.89205723845717e-23

MIR31HG 0.881085705870647 0.302045267958478 -1.54451757674626 1.01393928074474e-07 4.52902814443388e-07

TUBA4B 0.579385052537313 1.5144336509827 1.38618400310706 2.51144682072244e-06 8.51575384908985e-06

C6orf15 0.832624395900498 0.197948157951557 -2.07254318794529 0.0018243443913347 0.00350588948166133

SLC15A1 1.67207343870149 0.618046984141869 -1.43584979146428 4.22176299642995e-05 0.00011297966531542

CXCL5 13.5479830606468 5.556272922609 -1.28588871715226 0.00206732697263105 0.00393085321895573

ADGRD1 1.19443463432836 3.66334014076125 1.61683175717119 2.32428031142385e-31 3.08587701540982e-29

ASPG 0.347328880199005 0.955463722065744 1.45989872167667 3.12364454044463e-07 1.27216743122041e-06

ADAMTS20 0.0925713167910448 0.0433307387404844 -1.09517441072697 1.48263340602935e-08 7.6813835300062e-08

MSLN 93.1191958167662 268.070603851038 1.52546252039635 4.18305892847649e-08 2.00081604921007e-07

FSCN1P1 0.0353662142985075 0.0171534010380623 -1.04387714636844 0.000362507103489962 0.00080481932628058

PHGR1 0.333578688457711 0.71802117567474 1.10599927082345 0.00315423847009652 0.00576776239601122

MTND5P25 0.0446173902487562 0.128556831314879 1.52672823949315 0.000279150578188334 0.000634907967854548

AC078883.3 0.291449112885572 0.138111648927336 -1.07740900256819 0.00429182286564842 0.00762612755817855

FCN3 3.15879015671642 6.34782899757785 1.00689116451125 0.000502728181235256 0.0010838416961047

SLC34A2 273.091505264527 561.090056069204 1.03884790625265 2.33607697719821e-18 6.13164158602409e-17

COL6A5 0.332143902447761 0.716701394155709 1.10956373147766 4.4608580526223e-11 3.65117625283552e-10

BPIFB4 0.238556702825871 1.30284446475087 2.44926073539415 0.0042876383105627 0.00762067245866194

CLCNKA 0.192116837925373 0.503484071816609 1.38996216957605 6.86055591387648e-09 3.78098084707596e-08

C5orf66-AS1 0.324977512686567 0.0682865803806228 -2.25066589780837 9.7197101112385e-05 0.000242129585155636

NWD1 0.322511638577114 1.02126938723183 1.66294033641246 3.86541596789997e-10 2.65965510995915e-09

LRRK2 3.37560565353234 10.7656389148097 1.67321566267536 3.58194473581496e-21 1.38175160119237e-19

FSD2 0.0144468956716418 0.03255032516609 1.17191243369667 9.86604488511102e-06 3.0065328981369e-05

MSI1 2.29450679661194 1.13210196833564 -1.01918017274554 0.00252182003609817 0.00471795457878685

AP005131.4 0.0135441119751244 0.112353915418685 3.05231269093406 7.56209535156174e-05 0.000192608779908003

AC010789.1 1.36958491706468 0.632373736297578 -1.1148893627161 6.5766687253506e-05 0.000169231904784448

AC087501.1 0.0678492778109453 0.152795679688581 1.17119838685595 2.96206896103547e-05 8.16988564787415e-05

IFNL3 0.0538287138308458 0.0199554884429066 -1.43159036281309 3.29657304845207e-07 1.33611844806112e-06

KCNG1 0.360841968303483 0.0708712697750865 -2.34809434068115 0.00102775026234928 0.00208368937548205

CAPN6 1.91417171300995 4.19667869449135 1.13252775776906 2.78445610676081e-06 9.36714323738108e-06

FAM133A 3.00358217155721 0.605093105955017 -2.31145508071102 7.97682245680395e-07 2.98775806893437e-06

CLCN1 0.148020837656716 0.0606982307024221 -1.28607391679897 0.00149537410127277 0.00292864172358111

GRIFIN 0.02021426 0.0437034435294118 1.11237356795111 1.86820881927119e-06 6.51645341245594e-06

ANKRD44-AS1 1.17531745507463 2.38964634110727 1.02374663682772 7.33838640425905e-08 3.36414462213351e-07

AC010261.2 0.0219057581293532 0.0773106768858132 1.81935752404994 2.35082443240302e-08 1.17455331067268e-07

HSPE1P18 0.230015624825871 0.527421420311419 1.19722429904091 1.39499653633356e-08 7.25207285092622e-08

FSTL5 0.429185854328358 0.129491368231834 -1.72874659416393 8.59094772376879e-05 0.000216435538177115

AC064801.2 0.0346851758208955 0.0903721117716263 1.38155843759974 1.46164578619475e-06 5.21063767628081e-06

AC087783.1 0.318078898656716 0.100164170034602 -1.66701813580054 0.00191245229024792 0.00366106041424236

FGF10 0.0858559996666667 0.192703263183391 1.16639014281403 1.53742993507608e-14 2.24379448902512e-13

AL160274.1 0.00853615160199005 0.0636212671280277 2.897851402185 1.21869304842053e-10 9.21516584857658e-10

C15orf48 71.8544221890547 30.7504597512111 -1.22446896784921 2.81256172945889e-09 1.65890798578177e-08

MORN5 0.928904383283582 2.03920474027682 1.13440462648762 0.00310803524803623 0.00569012410695434

SIX3-AS1 0.0853267769154229 0.037692657449827 -1.17871504042768 5.13687374436698e-05 0.000135180888009657

AC060834.1 0.0776535215174129 0.192948506920415 1.31309262388657 2.80471210948381e-14 3.93378852278883e-13

ARHGAP29-AS1 0.238727711243781 0.0482041143944637 -2.30813784652339 0.00510816371378887 0.0089287580732489

HNRNPMP1 0.00815200148756219 0.0290040673217993 1.83102900772673 0.00289697828281043 0.00534126709146995

AC008738.4 0.0403596186567164 0.0839978589273356 1.05744001161554 2.40688915691752e-05 6.7752592056087e-05

KCNE1B 0.0328022896865672 0.0719293316920415 1.13278367754864 1.5122475293491e-05 4.43586121060681e-05

AC092574.2 0.0788618919900498 0.183147536643599 1.2156060688214 0.00131152333990179 0.00259665291344389

AC100774.1 0.0225159245422886 0.0829647653806228 1.88155304450919 6.60572734054873e-06 2.07448206182119e-05

MIR133A1HG 0.009671547 0.0728062784913495 2.91224428970326 2.27077802037264e-07 9.48469438869757e-07

LINC01060 0.0756007973333333 0.0329411281038062 -1.19851148756227 0.000109604873561983 0.000270403508201356

AC010533.1 0.042783889318408 0.0980259895086505 1.19609666157633 1.04600641779262e-08 5.5821025417811e-08

AC073592.1 0.11988225639801 0.281671719131488 1.23239657582221 7.2606067123219e-14 9.53782870230567e-13

MANCR 0.408517327328358 0.142592245626298 -1.51850174375126 2.15052785985705e-06 7.39556607155668e-06

KIF1A 6.44038742215771 1.52851879082007 -2.07501318833546 0.00346572557984789 0.00627571279976102

AC231657.2 0.060193948159204 0.134754859965398 1.1626469528113 2.35630764448795e-08 1.17686293054685e-07

AC025180.1 0.0545693168656716 0.12770923899654 1.22670101080083 0.000261339018943869 0.000598277573291606

AC092068.3 0.0106294274626866 0.0259400306955017 1.2871162962954 2.08274947409634e-05 5.93366647047239e-05

AL157770.1 0.0459244656218905 0.0952909698961938 1.05307657160914 1.03042034748228e-05 3.12612274028179e-05

MYO16-AS1 0.384345578258706 1.06594842754325 1.47166166362036 0.000339719643672495 0.000758723848966417

CCR6 0.057818073358209 0.116908670536332 1.01578949082725 1.09817166560397e-12 1.18062087477471e-11

TMEM132C 0.0525192400099503 0.163700212543253 1.64013825027298 1.21339888243419e-16 2.47660145034143e-15

SNORA59B 0.0246459927363184 0.0532413549480969 1.11119419341715 0.000160133805221904 0.000382603264857087

RNA5SP101 0.131659616865672 0.361047877058824 1.45537725501344 2.92618905458043e-07 1.19808275225707e-06

CNTN3 0.399440770353234 0.834486515256055 1.06290714265408 2.73320979744736e-10 1.93261028321748e-09

APOBEC3B 8.13418500696517 2.83327435137024 -1.52152749697408 4.82722286097867e-18 1.20460351503437e-16

AC008737.1 0.180458274875622 0.368483990346021 1.02993664017669 1.21190399579728e-09 7.6478020962288e-09

FLJ42969 0.168282789199005 0.0797536508131488 -1.07726516884009 4.6534006769209e-09 2.63390953050055e-08

LEFTY2 0.247329017308458 0.634739870408304 1.35973395605341 9.51663720820932e-18 2.26133125873003e-16

CLDN14 0.327716831781095 0.16113106115917 -1.02421514417736 1.3386987091509e-05 3.96936358361634e-05

AF127577.3 0.725140868955224 0.292271061418685 -1.31095429552588 0.000390987021149844 0.000862309090270803

ARHGAP26-AS1 0.0595229888557214 0.12550965366782 1.07627945830033 0.00402269835749468 0.00719043200297232

TPRG1LP1 0.0969858561691542 0.327465664844291 1.75549737249679 3.33633285809578e-12 3.30491501879462e-11

DKK1 14.6508270781343 4.28862462386851 -1.77239516098537 4.89983977310397e-07 1.9204731698824e-06

AC090192.2 0.418088369950249 0.102674632387543 -2.02572813024276 5.7680810392438e-13 6.51350191673484e-12

AL109935.1 0.0279532545771144 0.0582662329411765 1.05964377504677 0.00113368339212384 0.00227618857543584

AL365361.1 2.24730504731343 4.71396439273356 1.06874489309735 4.72512600606672e-16 8.70250479905217e-15

AC092436.2 0.103730091343284 0.282694928096886 1.44641152945412 7.51780970826944e-05 0.000191576110402509

ADGRF4 2.99878828313433 1.27969582274394 -1.22857874074695 2.53013408285085e-13 3.04038520061383e-12

C8orf34 0.179179228074627 0.431318658712803 1.26735072991699 1.22706252982066e-19 3.83983526208181e-18

PTPN13 4.35120726169154 11.8932203103806 1.45065176073585 5.99164644994132e-19 1.71773092668653e-17

AC023906.4 0.0320625870746269 0.0762143662352941 1.24917212934403 7.45755008016298e-09 4.08581720137134e-08

SILC1 0.022571396880597 0.0986299428304498 2.12752999181419 1.83069900974748e-10 1.33875983734207e-09

RFTN1 9.57615104975124 19.9799531211073 1.0610353841702 1.30373719267847e-30 1.56391281665597e-28

MIR126 0.686231967661692 1.58674310761246 1.20930033638645 6.20908123057243e-10 4.12180513728534e-09

AC000120.1 0.105894787378109 0.216149272214533 1.02939640353277 1.69847318686518e-08 8.71379509674783e-08

AC010768.2 0.0489816026865672 0.118875452352941 1.27913894591767 1.00234277320033e-05 3.04992791064329e-05

MS4A8 2.17186385893532 4.49999121198616 1.05098851189857 1.89034559509167e-08 9.61341614461829e-08

AC012409.5 0.00631947606467662 0.0145946555224914 1.20756330185881 0.000266689500375663 0.000609149643834507

LYPD6B 2.82929764634328 1.38948692375087 -1.02589170077406 6.32626591631364e-06 1.99220924365202e-05

CADM3-AS1 0.0466045225522388 0.143745564965398 1.62497557730417 2.8807765592169e-18 7.46110216804756e-17

PRSS12 1.73044251019901 3.81956114584775 1.14226587497506 4.754874518061e-08 2.24992764825205e-07

LINC01564 2.01967118238806 0.894982723564014 -1.17418869205149 1.47435086892302e-11 1.30556032613185e-10

POPDC3 2.52040609700498 0.416476262103806 -2.59735003096848 5.88580327559811e-17 1.26256250656948e-15

CFLAR-AS1 0.228678006368159 0.594227119152249 1.37769683002625 1.80078200627238e-09 1.09764626413081e-08

BLM 2.10835558656716 1.04211840034602 -1.01659900768378 3.39310642076616e-29 3.41181840470421e-27

KCNA5 0.149055455656716 0.318339980688581 1.09471917697008 2.18044130114607e-18 5.7562808481028e-17

FAM153CP 0.029184323358209 0.0856681611141868 1.55356550149233 3.71565089761175e-05 0.000100477607326163

SLC25A14P1 0.0196668740298507 0.044065392283737 1.16387838319673 0.000242549518828319 0.000559436491545614

MFAP4 24.3075544900497 58.1151649204152 1.2575099228133 5.00143773812202e-21 1.8919684942965e-19

AC100791.1 0.100755219950249 0.0415463677508651 -1.27806032617938 3.3299646649971e-07 1.34825364304472e-06

SRXN1 2.82448701487562 1.02875833266436 -1.45708475199632 1.70857551419623e-11 1.49200496205007e-10

LINC01356 0.265391097845771 0.0635884209204152 -2.06128399022753 1.4076102673755e-05 4.15477453191452e-05

ZNF695 0.530008931452736 0.219647543602076 -1.27082630613539 7.56414011195789e-15 1.16355023656945e-13

AL139022.1 0.0908942470547264 0.186561034155709 1.03738680059207 1.31116966340036e-08 6.85669030478008e-08

AC007496.1 0.0222737391542289 0.171358190761246 2.94359948020091 5.59328680443601e-07 2.16741844858777e-06

HOXA13 0.373981751069652 0.0753696360484429 -2.31091254244153 5.75629701936547e-08 2.68889365464809e-07

STPG3-AS1 0.249178763915423 0.523765788650519 1.07174070783545 3.73582363484128e-10 2.57626768565076e-09

MIR34C 0.357354723980099 1.14294291532872 1.67732458452519 3.97741703698155e-06 1.30169146776888e-05

RN7SL382P 0.0530160991044776 0.304955996539792 2.52409865828197 0.000405528674121044 0.000892006533473585

AC018647.1 0.0567615556567164 0.11376276067474 1.00304234548511 2.33985457963525e-15 3.878486227456e-14

C4orf45 0.0211808442338308 0.0578011049965398 1.44833697936805 1.20109391325269e-06 4.3475275975994e-06

PCLAF 7.89798996119403 3.16823491072664 -1.3178062251551 3.31737818500013e-43 2.52028592665982e-40

KPNA2 56.295087840796 23.6393129757785 -1.25182093468446 1.9918067598578e-50 1.36189787205277e-46

EFCAB1 0.477700070935323 1.04306152403114 1.12664726026392 3.62944140996327e-05 9.83125006933604e-05

PHKBP2 0.0220693575621891 0.136576136228374 2.62958888684344 4.01402873375058e-05 0.000107927335694139

HLX-AS1 0.0530819915920398 0.107860614705882 1.02287375546324 1.73319437349536e-09 1.06094149765215e-08

ELANE 0.221427957114428 0.788900528096886 1.83300601749747 2.15062153653676e-16 4.20439592739674e-15

AC138409.1 0.0576974410746269 0.157327695906574 1.44719342380581 0.000243767438760174 0.000561950671367545

SLC25A47P1 0.128916783631841 0.512439751591696 1.99094229459301 0.000163060188725556 0.000389119288163682

SNORD9 0.442215237313433 1.21936695287197 1.46331171014959 0.00330288882710681 0.00601104667429939

ABCA8 0.373933271293532 1.15582490044983 1.62807010793017 2.98354891352903e-23 1.53962382613243e-21

POU4F1 0.41488607579602 0.195361729449827 -1.08656736196819 0.00105326924205142 0.00213005869344176

AL592430.1 0.083785014 0.201264492467128 1.26432854319621 5.65614646254013e-10 3.77947010724272e-09

IL5RA 0.176087828606965 0.466999206858132 1.40712490781686 2.91050685244855e-10 2.04633322402231e-09

AC105129.3 0.0377471693034826 0.107339717716263 1.50774363468712 0.000446964959511019 0.000974451306705968

MIR27A 0.309506385572139 0.883954774394464 1.51400338451211 5.38362359288989e-13 6.09698158449435e-12

MYH11 4.01524170945274 8.35141997093426 1.05653467856377 1.38304890141041e-18 3.74889865744051e-17

CPA3 8.8002916241791 18.1580194406228 1.04498361365953 1.18476204947108e-12 1.26723668568768e-11

AC133785.1 0.350009643402985 0.101745335422145 -1.78243201671964 7.67900111716922e-17 1.61678737917304e-15

AC005332.2 0.123601819791045 0.25747381533564 1.05872573582386 2.82049239175514e-13 3.3597764335585e-12

AL133466.1 0.223661442487562 0.982102864532872 2.13455757067579 3.12848126942237e-25 2.05682602689187e-23

PTCHD1 0.0653971603457711 0.132892926435986 1.02296441758364 2.3482527721215e-10 1.67863861258555e-09

CTSH 62.7964867512438 129.589370224913 1.04519163107883 1.85961541328393e-15 3.13180305131253e-14

BCL2L10 1.09522527059701 0.35074997100346 -1.64271275031678 7.19853849634258e-10 4.72134359412397e-09

LINC00973 3.06932243542289 0.718368375467128 -2.09512446511346 0.000188474095913667 0.000444299820999723

AP001107.8 0.111022082686567 0.230228493598616 1.05221973343306 7.97349405414721e-07 2.98692045447098e-06

KIF11 9.0833021840796 3.80107696089965 -1.25680864071858 3.02643033964307e-39 1.08911670775313e-36

UBE2SP2 0.252970480646766 0.0925195039792387 -1.45113960890771 7.85827620544844e-22 3.36344059810665e-20

AC009127.1 0.0371897517412935 0.0749274614532872 1.01058945638 0.000179137932625422 0.000424229150342479

AL392046.1 0.0743963710049751 0.0370878994221453 -1.00428369049861 5.52201858917882e-07 2.14162235414125e-06

BEND3P2 0.0088610707960199 0.0186236616089965 1.07158379625952 5.23492156217166e-05 0.000137509704884167

RSPH4A 0.915012617711443 2.18645125387543 1.25672764150535 0.000137818844858687 0.000333570389989831

RNF7P1 0.0638769854228856 0.138161791903114 1.11299056560629 0.00228187546800335 0.00430290223730637

AC127024.8 0.12035616038806 0.28061295200692 1.22127161216506 0.000134569264120222 0.000326804242025224

AC115989.1 0.192762283034826 0.485596469307958 1.33293513877339 5.67657325003091e-14 7.57337943357782e-13

AL590762.1 0.0964124728358209 0.262892586262976 1.44718175320401 1.34663346758896e-05 3.99202529141102e-05

TMEFF1 0.0871663082288557 0.0173477327024221 -2.32902348899292 1.56935634727762e-07 6.75935371622723e-07

ZWINT 21.8746461492537 9.59408781314879 -1.18904213020295 6.38529865580022e-43 4.4778953393881e-40

RSPO3 6.96092021427861 1.11149262222491 -2.64677966675734 0.00299577449997992 0.00550301783695687

AC009303.1 0.055485412238806 0.121776331384083 1.13405333119369 9.01838472731647e-06 2.76702739838575e-05

MFSD2A 7.1954155960199 15.3723850588235 1.09519109734626 1.13912923127613e-20 4.09397956312775e-19

IL36RN 1.58920436231841 0.665381111207612 -1.25605184190986 1.51678415649063e-05 4.44726057890424e-05

RN7SKP80 0.679237936915423 2.09667478809689 1.62611416073794 8.43385609333272e-14 1.09424081666342e-12

RN7SL851P 0.105054206666667 0.220106566574394 1.06706825393364 0.00574558069137482 0.00992471445931624

AC004594.1 0.0761919177363184 0.232396807480969 1.6088803768844 7.45668272089401e-17 1.57255829538374e-15

SUSD2 11.1949838109453 55.4126297871972 2.30736239455576 2.74013170576663e-26 2.04203275620483e-24

AC138951.1 0.0264607602039801 0.0603592356920415 1.18972002754021 0.00207743664809231 0.00394870333764158

SOSTDC1 0.740694823646766 1.50174373803806 1.01968748807499 6.96297018002369e-10 4.57891883682731e-09

AC093158.1 0.0168328384477612 0.0445203542041522 1.40318659829209 1.98557530477102e-11 1.71310676925828e-10

SPINK5 3.9263267568209 9.02690599702422 1.20105134197218 7.67773929389228e-12 7.18146955157161e-11

KCNA3 1.15272221318408 2.42279736422145 1.07162885754018 1.08241533956227e-14 1.6177081714223e-13

HMGB3P9 0.0273656577114428 0.0942589675778547 1.78426334857462 0.000504431490618791 0.0010869997847797

LINC02487 0.14296656340796 0.0464645687889273 -1.62147484905684 0.00274127841292873 0.00508366996159484

LIPK 0.382787756218905 0.163767421307958 -1.22489629844415 3.78790911217434e-07 1.51704955656711e-06

SFRP5 0.455109065029851 1.1094997032872 1.28562505388024 0.000517341311782646 0.00111262757547027

AL033397.2 1.63688550452736 0.573286760761246 -1.51362454538474 7.21039098203254e-07 2.7277085660311e-06

PTCHD4 0.223176020751244 0.565392033055363 1.34106952981125 9.89109681084272e-16 1.72967709575798e-14

ALOX15 1.47750562293532 3.04720655088235 1.04432367365181 0.00204269673261185 0.00388861666575723

ECT2 15.1003407552239 7.04599848442907 -1.09970503623663 4.78053953603503e-31 6.13839231504967e-29

IGF2BP1 2.320054985 0.290388989747405 -2.99810033850652 8.7224275086171e-14 1.12846921646489e-12

ARTN 1.78229792945274 0.649150769584775 -1.45711302057536 2.97703292302579e-07 1.21706801860621e-06

C9orf24 1.51515464348259 3.83669774955017 1.34040006648771 5.11703671294304e-06 1.64011431031281e-05

AL772337.3 0.264617990099502 0.0994836682698962 -1.41137953655378 0.000231813729844718 0.000537160510993225

DEFB124 0.0420827617910448 0.123147999653979 1.54909190023386 2.28146221619544e-10 1.63388299588754e-09

RTN4RL1 0.805802377865672 1.73822510294118 1.10911695767838 4.19838771657983e-14 5.71557511440808e-13

LHX5 0.309273788741294 0.0829100314982699 -1.89926599599659 7.64754956414143e-07 2.87861933084597e-06

AC108097.1 0.0279957805970149 0.108718588477509 1.95731731853513 3.53706922604493e-08 1.71401210723474e-07

CTD-3080P12.3 0.0255607507562189 0.140933666619377 2.46301417169143 0.00141555004527707 0.00278643766184247

LINC01524 0.0226317440597015 0.0106030753460208 -1.09386499779291 0.000232843906306442 0.000539227843986553

KRT6B 4.26404017624378 1.13399448067474 -1.91080741290956 0.0048430187145936 0.00850716517945632

SHROOM4 1.51601397935323 3.16550814256055 1.06215404973538 4.37676159336469e-23 2.22086140219897e-21

AL450326.2 0.0263275271940299 0.0658984725224914 1.32367300202418 3.1175412063354e-10 2.17845559512706e-09

RNU6-944P 0.180401630298507 0.445008074740484 1.30261913788259 4.12066429304385e-11 3.3976535548613e-10

LINC02516 0.0619408424079602 0.128935724121107 1.05768913627965 1.49900651666942e-12 1.57140008550819e-11

AL133553.1 0.0501291403980099 0.109951996608997 1.13315240173738 1.95797848989478e-06 6.79232771418344e-06

ORC6 2.95787633681592 1.29551143771626 -1.19103998462771 9.59259715700433e-37 2.40694983710155e-34

NPFFR2 0.628788053935323 0.240915529020761 -1.38404641840012 6.13736144065707e-07 2.35787098471655e-06

MT1H 2.02579333422886 0.955625793910035 -1.0839693017906 3.48031558959404e-12 3.43386116072861e-11

AC022540.1 0.041758631199005 0.148049801539792 1.82593622871585 0.00295497846250534 0.00543720808325626

ASF1B 13.4566017064677 6.06667480415225 -1.14933623681795 3.44040933777683e-39 1.22201552452203e-36

NT5C1A 0.0684692513681592 0.415289934013841 2.60059076005628 2.31849232425819e-15 3.84774059881441e-14

PBOV1 0.0110272569701493 0.0617317962387543 2.48493980553259 0.00133076388873596 0.00263226964322908

TEX49 0.0244443439800995 0.120294842214533 2.29900219360605 0.000639817866295943 0.00135273799035204

AC005486.1 0.0540147376119403 0.146890082560554 1.44331199691764 8.69142966677754e-12 8.04707519926763e-11

ANGPTL5 0.0426191438507463 0.0923403956124567 1.11546030129968 2.61749698553997e-09 1.5518868969113e-08

LINC01267 0.0816338451542289 0.196698797982699 1.26874882290541 0.000274516937872302 0.000625409267039355

GTSE1 4.35091503542289 1.79940757750865 -1.27379684083008 8.83157652050778e-37 2.23651497996192e-34

CR391992.1 0.0497733602985075 0.113094468027682 1.18408266755574 0.000189811999698376 0.000447222449323793

HCG21 0.0752211871144279 0.199062078892734 1.40400743563452 5.18323256931419e-05 0.000136295943438845

HRG 0.165659527706468 0.0160003646712803 -3.37204448896655 3.54607230242897e-08 1.71715788724207e-07

AC084880.3 0.0334577837810945 0.0692592743944637 1.04966538750948 7.81311200349061e-08 3.56294387756994e-07

SCARNA13 2.78036425472637 17.1509442284429 2.62494219723918 0.000828521871426615 0.00171227235990129

LINC00670 0.00639801458208955 0.0150614791245675 1.23516727228345 6.83964609303791e-09 3.77221860545648e-08

PPIAP84 0.0667137605472637 0.135441298927336 1.02161144323358 0.00133522127525178 0.00264032259982186

AC005725.1 0.031809925840796 0.0945061115986159 1.57093062124132 1.36784843949822e-13 1.72003010667937e-12

AC107222.1 0.0485957853233831 0.0992116656747405 1.02967857262729 2.11796797645731e-06 7.29368221557636e-06

RPL18P13 0.229374106119403 0.114014130588235 -1.0084898967172 4.58137762264526e-06 1.4824973731584e-05

MYPN 0.159958062875622 0.0272714566608997 -2.55223004838471 0.000174938154564161 0.000415314544972779

AC110285.2 1.04952235666667 2.46127469086505 1.22967278004767 3.91528104724057e-14 5.36487658527202e-13

NEAT1 21.1456264308458 47.7651794775087 1.17559998527089 1.52910302437935e-14 2.23283330041512e-13

KCNH5 0.0575466694706468 0.0175318945301038 -1.71475052948957 4.32023726807806e-05 0.000115344093402904

GGTLC3 0.0378189721890547 0.0791678294117647 1.06580414124186 1.28574115434464e-08 6.73530369111778e-08

EGLN3 14.1823290631841 7.04626777948097 -1.00916326783367 1.81851790224111e-11 1.58044056645359e-10

AC015871.4 0.0107399715870647 0.0279916146055363 1.38200452986411 0.00111423398924606 0.00223894641142309

KCNF1 0.985628550940299 0.421222483256055 -1.22646160353408 0.0023440834958454 0.00440970267129903

AC092666.1 0.0107471561293532 0.0469126018269896 2.12602056661686 1.86963100237591e-07 7.95174908519118e-07

DUOXA1 2.13374733791045 5.4295047317301 1.34743125102297 5.56200702647563e-15 8.76272420357768e-14

AC013474.1 0.019436303880597 0.0433574597923875 1.1575263413562 3.23878820601388e-05 8.85365891399096e-05

LHCGR 0.0454330626467662 0.107448304941176 1.24182825761552 1.91334088295971e-09 1.16082238573532e-08

CNTN4-AS1 0.00692012599004975 0.0234831931903114 1.76275838673684 1.58212962092854e-08 8.15976713792109e-08

AL354714.1 0.0335801591492537 0.0919685550484429 1.45353160610816 0.000675547214721194 0.00142091950493153

PFN1P2 0.119128472577114 0.257056220346021 1.10956565431193 5.45671898864541e-19 1.57600180667423e-17

AC020934.1 0.0808110305970149 0.0259251960899654 -1.64019733118378 9.8782692372868e-06 3.00858200044314e-05

SLC16A1 8.60061082935323 3.23673996536332 -1.40989765966409 1.0967757640316e-11 9.9425976620034e-11

RPL7P46 0.0371188099004975 0.0780906857093426 1.07300002506663 0.000120039209850721 0.000294076709908386

AC079781.3 0.0779452447263682 0.163838292110727 1.07173966656932 4.93248367249791e-15 7.78888154981627e-14

C12orf56 0.676188792825871 0.290666549252595 -1.21806105364898 1.63709242798647e-11 1.43692162726027e-10

AC131649.2 0.37949696438806 0.798442816228374 1.07310074127802 6.2927951688315e-15 9.80672067621319e-14

AC005358.1 0.00836330659701492 0.0181141458892734 1.11497142409454 3.4729312400618e-08 1.68472276366957e-07

NNAT 2.00197175452736 5.98350415605536 1.57956900663855 4.98599488392448e-08 2.34832030437979e-07

AC025271.2 0.0182699886069652 0.0636073606574395 1.79971798983879 2.05597254895041e-12 2.10975765292866e-11

AC016910.1 0.0143570517910448 0.0379792870276817 1.40345329919843 1.97054102862159e-05 5.63629963739808e-05

OMG 0.546702931064677 1.34256676183391 1.29616481544749 3.37275873786894e-09 1.95890744278436e-08

AC004691.1 0.053006132238806 0.141955085155709 1.42120335189576 1.94329539143279e-07 8.23396064700729e-07

AC003092.1 1.03326864404975 0.166508763771626 -2.63354537854144 0.00124899509792408 0.00248382286979013

TRIM50 0.0614336134427861 0.159541183802768 1.37682873963283 4.54808897530256e-09 2.57803592693316e-08

KRT16P3 0.158242228950249 0.463923953529412 1.55175368528635 7.14632610777223e-10 4.69159911299977e-09

TPPP 2.11787654995025 4.51524473183391 1.09218568792527 1.48358984847382e-23 7.92503561635917e-22

GPR87 9.99505986383085 4.39080770225606 -1.18672885553345 1.36202818757892e-06 4.88351742662342e-06

SELENOOLP 1.60369354034826 0.426652707508651 -1.91026436627153 5.9430978001834e-06 1.88020502990186e-05

RPL7P41 0.0226284824875622 0.0473789 1.06610486603106 2.86123624195074e-06 9.60653218970694e-06

SLC5A2 0.21136395158209 0.474763996574394 1.16748118922627 1.02938338212227e-21 4.32468540821708e-20

KCNK17 0.613110374830846 1.68659478256055 1.45989467389054 4.9722903682536e-14 6.69252665215236e-13

MIR657 0.196117134825871 0.408257514532872 1.05776385095813 2.73768854692914e-06 9.21774981638705e-06

CCDC198 0.260267968119403 0.579250065882353 1.1541885368797 0.00011258440343364 0.000277179173094793

VEZTP1 0.0464364097512438 0.12108746200692 1.3827211498953 0.00110495626910655 0.00222275330685968

ARHGAP15-AS1 0.0341708050248756 0.0960177468512111 1.490536845763 0.000381910320419529 0.00084392399317073

MYOC 0.0364868036766169 0.201554271816609 2.46572168218512 1.31282618615401e-16 2.65771992533769e-15

AC068587.2 0.562481907313433 1.65578889512111 1.55764015136026 1.57651937816831e-19 4.83383464046001e-18

AC064802.1 0.0275468031094527 0.0747216026297578 1.43964049896877 0.0010850415828264 0.00218751933438759

CFAP73 0.772405623134328 1.99074409377163 1.36587720507495 3.74649398687754e-08 1.80685259286018e-07

TCEAL3-AS1 0.0534591344278607 0.160217545294118 1.58352376097317 0.00128719865750387 0.00255366917762446

FOXD1 0.966168639074627 0.460494891709343 -1.06908987248171 1.79603239468684e-06 6.28151994816945e-06

SLC16A12 0.520177321278607 1.07901783971626 1.05264331045894 5.93206659515507e-06 1.8775848864388e-05

RAB3B 1.33445915856219 0.403807634737024 -1.72451505925061 1.62711166816976e-13 2.02187660719868e-12

AL590822.1 0.0819740393532338 0.1676095383391 1.03186525738613 7.84669347295064e-07 2.94668497164905e-06

RACGAP1 13.0061171542289 5.60286189273356 -1.21495448767493 2.19328540397557e-43 1.71389587996377e-40

AL031008.1 0.0729994399004975 0.173498926470588 1.2489694362689 4.88821929566696e-16 8.98473103067818e-15

AL031005.1 0.0543861673631841 0.120002361487889 1.14175112982315 0.000415763278731311 0.00091239073042617

AC061975.6 0.661087673781095 0.229961886612457 -1.5234468435511 3.1967078274465e-07 1.29872191147745e-06

ANGPTL7 0.0418724879850746 0.130001414813149 1.63445277629132 2.90938659581425e-08 1.42960336678979e-07

MBL3P 0.169101777014925 0.515150023771626 1.60710081966121 3.32918330024467e-11 2.78705733889476e-10

SRRM1P3 0.0237863144577114 0.0532132843217993 1.16165469694293 0.00109760936895828 0.00221041280030992

AL158834.1 0.0228779486069652 0.0512896780622837 1.16471081972983 2.64081669255175e-05 7.36777889842807e-05

AC003957.1 0.015208213119403 0.0305895758961938 1.00818945067945 3.81444406223118e-08 1.83703196164858e-07

DES 2.9500249839801 6.29756222799308 1.09406629989021 3.58209458987289e-12 3.52791815027092e-11

ZIC1 0.462445843706468 0.156414331034602 -1.56391172212882 0.00068693881516168 0.0014428827735713

CCND2-AS1 0.049047912238806 0.123690414429066 1.33447006740118 1.88742127744529e-10 1.37692643206532e-09

AL138899.2 0.0910088586567164 0.185743687647059 1.02923429585575 9.27456999147352e-08 4.17203107346712e-07

OIP5 3.86528337562189 1.30263130622837 -1.56914538177362 2.91399531978519e-44 3.06529892292788e-41

AC002551.1 0.0285909744776119 0.0608391121107266 1.08943930568827 0.000525536465006311 0.00112865507011563

AC015727.1 0.115340930995025 0.234352947301038 1.02277836621972 3.17964145418374e-11 2.67332289492546e-10

DNAAF1 0.370395258656716 1.1860473053218 1.67902401648395 1.02516890305292e-11 9.3554786448106e-11

AL355102.5 0.0357177107462687 0.0798554543598616 1.16075133444086 4.20198160254346e-05 0.000112516347003685

THOC7-AS1 0.032376418159204 0.0898253061591695 1.47217856005675 1.20975800656039e-06 4.37657162426279e-06

MTCO1P11 0.0296095602238806 0.0839451799134948 1.50338442573674 0.000220895530740334 0.000513863465658598

IL1R1-AS1 0.0307274296517413 0.0765251552941176 1.31640697685108 9.37774914657384e-07 3.46456084234492e-06

PGGHG 19.1266185303483 39.7458851384083 1.05522367046328 2.47458029466084e-08 1.23009398507768e-07

WDR11-AS1 0.0313648415621891 0.0649083224256055 1.04925519481584 0.00011490633950333 0.000282361939390483

AL122001.1 0.0284510651741294 0.100878675294118 1.82606666445562 3.56897241941598e-06 1.1771755387244e-05

MPHOSPH6P1 0.107620230646766 0.428004308719723 1.99167601648487 2.00579423626842e-09 1.21358410806807e-08

HPCAL4 0.330565369537313 0.830932462726644 1.32979562718304 8.94878029884306e-07 3.31998292421809e-06

RPL39L 25.931814021393 8.87876430242214 -1.54629232200356 4.13971287222312e-31 5.39148319310964e-29

SNORD116-24 0.40754271641791 0.889943619723183 1.12676265769485 7.03711313152122e-05 0.000180312014378026

AC139103.1 0.0238040478109453 0.0655639606435986 1.46169608801933 0.00334663784391612 0.00608257210467211

SLC1A7 3.57142001333333 8.84221034145329 1.3079092433808 2.23923911287684e-16 4.35274980363764e-15

CTXND1 0.0723779471044776 0.332505077622837 2.19975427845767 5.03771501186369e-16 9.21646797669898e-15

GGT3P 0.0879933861094527 0.186808121896194 1.08609018583398 2.75280519644918e-16 5.2871644749217e-15

MTND6P4 0.853817633731343 1.8692206667474 1.1304370296277 2.30448656908741e-07 9.61715322193119e-07

AC119424.1 0.0562917554726368 0.176680551903114 1.65014769926407 5.34812816261628e-06 1.70678302505899e-05

AC093849.1 0.00630444273631841 0.021925094550173 1.79814230590138 0.000260643747751456 0.000597235799346709

MAGEA6 8.64453873861692 1.91291908935294 -2.17601313221225 1.66028601745023e-09 1.02157081163698e-08

AC011498.4 0.147002312955224 0.30809170183391 1.06752097043059 1.72136408936735e-14 2.48965139313575e-13

GOLGA8O 0.0121576434228856 0.0252630949100346 1.05516777937449 1.00315556737132e-08 5.36808936951782e-08

AC020779.1 0.014796561641791 0.0320197714532872 1.11370104256513 4.68058772236689e-05 0.000124273443555708

AC018797.2 0.0327959152338308 0.107390765560554 1.7112819006111 0.00241659092737239 0.00453722536305587

LINC01394 0.0939067508955224 0.197302075467128 1.07110535105887 3.15985021848738e-12 3.14948627826639e-11

CA2 9.00806751791045 18.3439066968858 1.02601137606865 0.00544565213342281 0.00945393387582405

UCK2 6.96567139552239 3.2329694850173 -1.1074025252298 2.99956230185135e-19 8.92688019103748e-18

GNG4 4.00499307373134 1.28716584295848 -1.6376018002073 3.73361088462777e-16 6.98935202261416e-15

DUSP5-DT 0.342080581890547 0.107611906193772 -1.66849850557392 3.02551865472304e-06 1.01121758776335e-05

AL139317.4 0.0762977011442786 0.169388988858132 1.15062860092381 3.83085425448392e-06 1.25673340362403e-05

PLA2G4E 0.500672287522388 1.0534229813045 1.07314632974251 1.09420554288118e-06 3.99381236620157e-06

AC093523.1 0.0594329101492537 0.133087155363322 1.16303740942342 6.84102567178462e-11 5.41655919602456e-10

NUSAP1 17.1406133333333 7.43858474740484 -1.20431866572597 9.95920913488953e-42 5.13932773281564e-39

ABCA10 0.0980900925771144 0.226570030688581 1.20777771085813 5.4286237195328e-16 9.8587555597093e-15

NT5DC4 0.167921690253731 0.0604669678442907 -1.47356945256072 5.63161583678913e-10 3.76760676815084e-09

LHX2 0.494921538751244 0.190097953636678 -1.38045682720663 8.72318741351765e-10 5.6401696397094e-09

CKAP2L 3.6216005920398 1.27973521211073 -1.50078211207682 1.15448363873509e-45 1.66184881681077e-42

PFN2 48.9429409452736 18.0645706055363 -1.4379378321573 1.99593070389231e-31 2.68909875622929e-29

AP000753.2 0.0601723399004975 0.187805117889273 1.64206401395919 0.00028645237465114 0.000650271617422699

AUNIP 2.04882863283582 0.718993247612457 -1.51074919332728 9.39044635015823e-44 8.02589711490086e-41

TDRD12 0.470623243039801 0.0945378407820069 -2.31560875440308 1.88624283497616e-06 6.57600274526424e-06

ADAMTS8 0.487413129940299 1.67211183958478 1.77845432560308 1.71070548976348e-25 1.16387550112018e-23

CDKL2 1.69947696014925 4.23027376678201 1.31566022858984 1.39855859370596e-19 4.33188873588426e-18

MAL2-AS1 0.169659447980099 0.383303114290657 1.17584394694798 1.31020536044528e-09 8.2113007809758e-09

PPP2R2C 2.5123045918408 0.677971321429066 -1.88971523425126 1.72532273891635e-07 7.37651663426015e-07

AL512383.1 0.00821449505970149 0.020326188384083 1.30709590151139 1.28856605041611e-08 6.74880916868644e-08

AC090950.2 0.0206386762686567 0.054099182283737 1.39025634609143 0.000905289537886067 0.00185410130756207

MEGF11 0.174009993840796 0.59260286750519 1.7678954411393 6.86344993946509e-10 4.51783640926268e-09

FAF1-AS1 0.0369578169154229 0.22461686384083 2.60351480190133 0.00301549712464295 0.00553330066145486

RN7SL19P 0.0496933306467662 0.123923399723183 1.3183244833174 1.34283894412225e-05 3.98120814327843e-05

KAAG1 0.103482780393035 0.0430027191038062 -1.26689093140911 9.54550252311278e-05 0.000238289059882379

PTBP1P 0.202168804547264 0.448925646190311 1.1509161159644 1.10646684968313e-11 1.00122851141651e-10

FAM83D 9.75987062288557 4.19606772975779 -1.21782405851221 2.09631787127503e-34 4.03762632249099e-32

DMBT1 10.9383943666169 38.3753878572664 1.81078034952011 2.06874121736363e-11 1.77868822052484e-10

TRAJ4 0.204256491542289 0.415424142560554 1.02420313162695 4.33249629160566e-05 0.000115648812781002

AC097634.1 0.140931259114428 0.282221372916955 1.00183560621705 3.30417244548795e-11 2.76781367179465e-10

UPK2 1.72017870853731 0.506230680034602 -1.76469160407835 2.55990144939435e-06 8.66585320006823e-06

VWA3A 0.23968109720398 1.00994470282353 2.075088265366 3.99371471303891e-14 5.46413693854999e-13

AC106712.1 0.118990323313433 0.0579049975536332 -1.03908448151094 4.02921703716159e-05 0.000108303769991518

AL360091.1 0.168964633283582 0.352930591349481 1.06266318420445 5.66415665673528e-10 3.78302038001734e-09

MAT1A 1.47290687320398 0.46595844832872 -1.66039300271617 1.03628653888267e-05 3.14237279971565e-05

NR0B1 4.97438060319403 1.21709236505882 -2.03107823800552 1.39335570268042e-05 4.11892331045282e-05

CD109 6.23698379373134 2.93583172249135 -1.08707923278615 6.24663461750959e-08 2.89912534853024e-07

ZNF33BP1 0.0325863767164179 0.105226794294118 1.69116125761741 1.25798529289565e-09 7.90395078352769e-09

LAMC2 72.974642960199 34.9290002387543 -1.062969897595 3.65694360111791e-06 1.20314456261969e-05

LINC00332 0.0350010870149254 0.193312785605536 2.46546542671711 1.95033669817614e-09 1.18247556031193e-08

HSD17B6 5.92800958109453 13.8451300536332 1.22375892116053 2.07079269455365e-14 2.95441732895369e-13

AL117329.1 0.153027782716418 0.0568152111314879 -1.42944446373377 2.70638356495874e-10 1.91512523936925e-09

DAAM2-AS1 0.131154233701493 0.273909261536332 1.06243366886411 1.41061842200667e-20 5.0039447265736e-19

VWA5B1 0.0262751945771144 0.0529527988304498 1.01100549226467 2.49441946009666e-05 7.00291236231202e-05

HMGA1 191.627936716418 76.3339446989619 -1.3279112491672 2.06777010631473e-38 6.4265355008759e-36

SLC6A20 0.888698960965174 1.87442465802422 1.07668113152961 3.91745739184649e-06 1.28345064287256e-05

AL591721.1 0.0555766845273632 0.129578390795848 1.22127346946874 0.000963893448968708 0.00196383237703324

INA 2.32722854621393 0.676780928086505 -1.78185207950914 2.19547929795983e-10 1.57892082038394e-09

SEPTIN14P12 0.143486716467662 0.0674504516262976 -1.08901717494469 2.64564792198574e-11 2.24715747410901e-10

HHATL 0.163927924029851 1.09019939289619 2.73345848803324 1.00932610107742e-11 9.22629306967495e-11

HOXA9 0.682705126432836 0.115089619733564 -2.56850486662614 1.82013353225694e-05 5.25167761443478e-05

AC008870.5 0.308710007114428 0.827287531591696 1.42213658885794 1.38728744387781e-07 6.03792355029569e-07

MIR6071 0.443059004477612 1.61795597647059 1.86860160560051 1.00019663035823e-19 3.17347770769114e-18

OR5BT1P 0.0138784175621891 0.0315795856747405 1.1861491640539 2.47181525519464e-06 8.39281778144921e-06

AC126323.1 0.243826980766169 0.523128156768166 1.10130664674497 1.02309437194216e-16 2.11341624415544e-15

MBL1P 0.431950473830846 1.27374335128028 1.56013680397317 1.68393035118436e-26 1.28646634371207e-24

CDC20 30.5814657263682 10.8010018262976 -1.50149242166881 7.28847886202323e-44 6.64466322921118e-41

PARPBP 2.34188066467662 0.959911102422145 -1.2866948534045 2.81275742155809e-40 1.13130758058256e-37

AC011284.1 0.055938631840796 0.132586771695502 1.24501997000597 1.10723223968393e-05 3.33511032547968e-05

AC006946.1 0.112748225323383 0.234763939031142 1.05810609457322 2.01804713346666e-07 8.52015886080783e-07

SERPINB7 1.17485495204478 0.32320098000346 -1.86197917448952 0.00523879536922754 0.00912734446097421

LINC01055 0.0805971074527363 0.185762569602076 1.20465986590002 1.99712519648429e-07 8.43573345542013e-07

SRGAP3-AS2 0.728060575970149 2.18275676871626 1.58402097995668 6.7849704139351e-07 2.5809310267194e-06

KIF15 2.54146571681592 1.13389397785467 -1.16437501892747 1.22190046512411e-32 1.92063090351405e-30

CHRNA2 0.0090152687761194 0.0257390139515571 1.51351437660446 2.24078941455973e-10 1.60643749641438e-09

AC124319.2 0.137694016268657 0.277800225778547 1.01258190582171 5.47451382765327e-11 4.42197144673116e-10

AKR1B10 93.4087908573632 20.3855354433391 -2.19601247983468 8.85852313511971e-05 0.000222725324274245

ETF1P2 0.0443664691044776 0.157462725608997 1.82746870967892 0.00166020456712335 0.00321735952035879

MAGEA4 10.4556540694776 1.15225503581661 -3.18175133679473 7.19701666995246e-08 3.30432106636226e-07

GJB3 8.57318468130846 3.45887439633218 -1.3095285964689 3.2970674388011e-06 1.09461998605499e-05

RAB5CP1 0.0806285019900498 0.193147246920415 1.2603392904429 0.00360422772970892 0.00650277910202117

LINC01625 0.0205638271890547 0.0430335090968858 1.06535169391679 9.71923815679354e-08 4.35691804000055e-07

STMN1 40.2444475721393 17.6782418235294 -1.18681495216052 2.75854835032821e-30 3.22420074279814e-28

VILL 2.3622654721393 5.37090521418685 1.18499415664519 9.25748774803875e-11 7.15030471360801e-10

PI3 59.08228723 7.79556807871972 -2.92199961373061 0.000342104906462973 0.000763552243492926

PRX 1.37860828358209 2.9262030132872 1.08581727472618 6.97296395512356e-19 1.96608829043948e-17

GRIN2A 0.0714056550895522 0.212274446193772 1.57182046812699 3.74624706694171e-15 6.01643319323874e-14

LINC01894 0.0612298084577114 0.138485234186851 1.17742608384099 3.35835681659067e-08 1.63290771437786e-07

STOML3 0.430083737761194 1.08617350913841 1.33656509614578 0.000141015897373262 0.000340434706784245

AC112250.2 0.0256641433830846 0.063773380449827 1.31320024935733 0.000102026364215768 0.000252984683708183

C2orf91 0.0545987336666667 0.113619591034602 1.05727221966475 6.0822178802075e-05 0.000157631629890718

HMGB3P14 0.052482964079602 0.131806553010381 1.32850099198659 0.00489402632675123 0.00858738821047323

CCNB1 27.4145079502488 10.501701017301 -1.3843165516954 9.68423520012887e-46 1.47146573735291e-42

NCAPG 5.53165184179104 1.88500648027682 -1.55314087395154 1.78505017839225e-46 3.75547095223293e-43

MIR23B 0.199565194527363 0.66629304083045 1.73929669811911 2.47115575134538e-14 3.48382009274722e-13

SAXO2 0.527745743482587 1.26979869048443 1.2666848524991 1.21013726534294e-08 6.3771202711232e-08

CYP4B1 10.8207382746766 45.2848810069204 2.06523053230558 7.5384115876804e-24 4.23358433106897e-22

LINC00355 0.544292553726368 0.143685638923875 -1.9214664245196 1.82545295744517e-08 9.31284058685421e-08

PDE9A-AS1 0.0185750431044776 0.037269047650519 1.0046123952023 2.1655916728169e-08 1.08916756622917e-07

TEX19 0.57515296080597 0.117907806249135 -2.28628645191549 3.43201827487992e-13 4.03376449583007e-12

DNAI3 0.289609417895522 0.615570979134948 1.08781669933108 0.000110833208249136 0.000273187477074069

AC026992.2 0.0701649107910448 0.167849969906574 1.25835064877311 1.31183920898639e-10 9.86494428533895e-10

AL132657.2 0.0361395237810945 0.0821093238408304 1.18396855947467 2.55651663125103e-05 7.15887476857947e-05

RN7SL652P 0.099879695920398 0.326412127854671 1.70843132753272 0.000786186783879599 0.0016311795280767

AP000919.1 0.0628813427363184 0.145073775570934 1.20608282291618 7.47358932746738e-05 0.000190531942679188

GCNT1P1 0.0222426389850746 0.0447747707716263 1.00935807899134 0.000467999699543336 0.00101576285467531

SIM1 0.0818905600099503 0.0197090241107266 -2.05484081046966 2.49600702552232e-05 7.00665012296371e-05

COL4A3 1.70652469668657 3.72590711809689 1.1265304171084 6.15325099145897e-17 1.31272554302966e-15

CAPN8 10.9534620206965 25.8340689387543 1.23788796064214 1.38194389482122e-21 5.70077911362902e-20

AC006947.1 0.0597492805472637 0.11957039916955 1.00086703382645 7.61134473136166e-07 2.86538580045067e-06

AC025423.1 0.107298065024876 0.268897386643599 1.32543167475323 6.72306632674917e-05 0.000172750717809648

LINC00316 0.111382209502488 0.240819293044983 1.1124321601375 0.000501747213179393 0.0010820679924662

KCNJ15 3.56096434393035 7.26444570823529 1.02858473152126 2.88623692881308e-10 2.03083560594386e-09

AC009084.2 0.219352868621891 0.461824705823529 1.07409178019019 9.71058814624367e-12 8.91053560678149e-11

CHIAP2 0.165607245900498 0.874275868799308 2.40032278200228 8.60812308589712e-11 6.69031447568304e-10

AQP6 0.11990774320398 0.275846063605536 1.20193856725232 4.14658374862354e-07 1.64838758030311e-06

ITM2BP1 0.0528638539303483 0.110434973079585 1.06284361345876 3.7002170962416e-05 0.000100109741400938

CPAMD8 1.78507449208955 4.10448384307958 1.20121652822719 4.00329445273035e-20 1.33524516197774e-18

ITGA8 1.39199027034826 3.06395185882353 1.13824450248775 1.46034928044965e-22 6.98261412942271e-21

C3orf86 0.21054441960199 0.555505244048443 1.39967589298244 6.38490079003109e-15 9.93893207782301e-14

NME9 0.217393081626866 0.452503458961938 1.05762279188106 1.18699506444125e-07 5.23533543178007e-07

LINC01700 0.00872395544278607 0.0230790142906574 1.40352730089438 7.6707539005377e-07 2.88576505061494e-06

AL445493.3 0.238069967164179 0.812702214013841 1.77134119031369 8.27525787558394e-18 1.98012513470884e-16

AC005722.2 0.299794134825871 0.660090393079585 1.13869144264862 3.5070149635929e-07 1.41386879796972e-06

HEPACAM 0.0041087891840796 0.00836281586851211 1.02527548838283 0.00548758193136553 0.00951955891303103

ALDH3B2 6.696655165 3.07022293546713 -1.12509726379832 1.88946714209047e-06 6.58556471723899e-06

COL6A6 0.493230593781095 1.10808845019723 1.16773885109426 2.22040223257655e-13 2.7002223682067e-12

AC139887.4 0.505173387661692 1.16010163072664 1.1994006525931 1.26002130809575e-16 2.56029589720793e-15

LINC01266 0.0391584988756219 0.0877347614602076 1.16382310639689 3.66127935202985e-16 6.86803774197643e-15

CD1E 1.00997212949751 2.66436231861592 1.39947480161075 9.60559252115966e-18 2.27851652605132e-16

AL157931.1 0.535156039452736 0.249792153460208 -1.09923145092186 0.00165901969432175 0.00321612904714417

UPK1A 0.528213818885572 0.127836776629758 -2.04681910845268 3.66109831316922e-05 9.91065431653189e-05

IRX6 0.68267033758209 2.07719652681661 1.60537674585209 4.0361565457912e-12 3.92842994759393e-11

C20orf85 5.08303033253731 13.9723576044637 1.45881472715523 1.92040480803298e-06 6.68317489498689e-06

FANCB 0.647467309452736 0.31819060366782 -1.02491612036596 6.32430095924034e-31 7.97095074816697e-29

ETV5-AS1 0.132434646318408 0.322814716366782 1.2854257532432 2.13995317752028e-12 2.18958920333631e-11

RNU6ATAC16P 0.182336833233831 0.408710741764706 1.16447413621179 1.29857733490461e-05 3.86212376137898e-05

RPL12P44 0.0670972945771144 0.135719260692042 1.01629897438579 3.61762978462144e-06 1.19178721524207e-05

DPF1 0.333703299353234 0.12258056532872 -1.44483568716215 2.81099893229614e-05 7.79804904004896e-05

RPS3AP16 0.023956058358209 0.0516615962283737 1.10870166829761 4.49353287186994e-05 0.000119641380217949

PGC 147.354138390497 1285.00497048609 3.12441445613237 7.83436256582252e-19 2.19089791590231e-17

TTC16 0.125869925368159 0.307724036124568 1.28970352102081 1.3574502656057e-12 1.43234046158626e-11

OR7E36P 0.0395846085572139 0.0856016496193772 1.1126990139142 0.00466004085401492 0.00821794553854588

AC022893.3 0.0470949020895522 0.121973217404844 1.37292159382665 0.00336554095480053 0.00611372339157086

JMJD7-PLA2G4B 0.453278412338308 0.910958451972318 1.0069878018278 4.938305417414e-18 1.22672709506152e-16

AC087521.2 0.0476588025870647 0.11375565400692 1.25512364366582 9.97482698957088e-12 9.13023822505902e-11

AC012467.1 0.375500403233831 0.929923100449827 1.30829696118151 5.82830117722563e-15 9.14538366019053e-14

KLK6 9.43380627390547 2.15473450591695 -2.13032985536905 7.96697798516427e-05 0.000202111907888176

NR4A1 10.3817613129353 22.6678898034602 1.12659886990866 1.41388630769357e-10 1.05395994863503e-09

CCNB2 12.6866256303483 4.49316892525952 -1.4975031867566 1.00546957314428e-49 4.58326547091601e-46

DEPDC1B 3.60306943781095 1.29385715647059 -1.47754810473937 1.10056840614263e-40 4.70321029812515e-38

AP002784.1 0.352144532288557 0.12885120467128 -1.45046165684595 1.65953795271884e-05 4.82393059909239e-05

AC015983.2 0.0731385566169154 0.178088873079585 1.2838933196444 3.19618179020137e-07 1.29870111368307e-06

AL133445.2 0.111770558208955 0.36188551432526 1.69499314624155 5.65708949594794e-05 0.00014756451856383

SLC25A21 0.753512592965174 0.327628758788927 -1.20156962258022 0.000129049124411426 0.00031417959343533

DTL 5.17350640746269 2.44921077370242 -1.07882548190723 3.27648626797052e-34 6.2230485714579e-32

LINC01611 0.363106665820896 0.0882495110034602 -2.04073322556682 1.28478792161386e-08 6.7315995509845e-08

OFD1P17 0.0207548524527363 0.0550076468096886 1.40618351098693 2.08278682010438e-06 7.18247629931343e-06

AL353746.1 0.0417955000049751 0.0079966057266436 -2.38588795750396 0.000415114989621153 0.000911187396961359

GACAT2 0.0954843687562189 0.0457384537716263 -1.06185698145281 0.000315935874078988 0.000710420632743593

CENPA 6.03543047761194 1.87253624463668 -1.68846303137662 2.92595017939253e-47 7.27497612785325e-44

CPB2 1.37841606020398 5.73692760776125 2.05726689734694 8.63405375665472e-11 6.70856165467348e-10

CYP4A22 0.0119798121144279 0.0352210325190311 1.55583192121288 2.8788416839254e-11 2.4338893371054e-10

IGHD 34.7253571893035 84.0749189349135 1.27568594868314 2.83684074349309e-07 1.16497889391195e-06

RPL6P7 0.0608907454228856 0.166217610692042 1.44877836359437 2.80624274892795e-09 1.65625246403063e-08

DIAPH3 1.73148829094527 0.761947862179931 -1.18424844456616 3.93158028310795e-32 5.65940635489487e-30

MGC27382 0.0649521327810945 0.243445309010381 1.90614889715183 4.41867421267e-13 5.0841707916081e-12

AC091588.3 0.0984336779104478 0.256770731799308 1.38325685911613 1.83731946017813e-11 1.59475364125268e-10

HHATL-AS1 0.0289668901940298 0.11829427716263 2.02990356645659 1.54826696900777e-08 7.99567628443401e-08

BUB1B 5.91858771393035 2.24969846366782 -1.39552131876275 8.21119357376762e-40 3.25472672815282e-37

KIF2C 11.4809563875622 4.05540787231834 -1.50132390072981 3.33992730585878e-44 3.26239327911563e-41

KNDC1 0.930306486915423 2.34055620463668 1.33107341787359 7.88008907972092e-19 2.20143448754205e-17

Z99289.1 0.0895735357860696 0.220094367231834 1.2969777627841 4.92661875253193e-08 2.3242851139983e-07

AL022326.1 0.0276427857860696 0.0116699780449827 -1.24410116817684 0.00400916200966983 0.00716811014999476

ORC1 4.0819573880597 1.50514666678201 -1.43935704690804 8.80129220134405e-42 4.62914118666846e-39

SOX15 2.79460418159204 1.14501157453287 -1.28728177691831 0.000185412195148291 0.000437874409576527

DLGAP1-AS3 0.0555140386965174 0.116528668875433 1.06976037707844 6.73826101500527e-06 2.11343393073847e-05

SERPINB4 1.80917142123383 0.725917373709343 -1.31745186116985 0.000312943022215427 0.00070421191851176

ADGRF5P2 0.0637807563383085 0.171742073982699 1.4290504083182 6.03844030736941e-23 3.01904933940406e-21

AC022001.3 0.030095861840796 0.0769147514532872 1.35369518760304 0.0043615407461878 0.00773744174665864

LCN6 0.0388158264825871 0.0958381165536332 1.30395454968282 8.37067166783325e-13 9.17586653768495e-12

CD207 2.6777047378607 10.5843788565052 1.98286781430917 1.16304287665252e-19 3.66465699037401e-18

LINC01655 0.983703477363184 2.30788946961938 1.23027872404175 2.00615668833958e-12 2.06427334183926e-11

MTFR2 2.8390726920398 0.97694361384083 -1.5390725866934 4.17675038873849e-51 5.71170615659989e-47

AC008915.1 0.0389443504975124 0.0825967229757786 1.08467048645049 2.51955035258058e-09 1.49836273413957e-08

C6 0.376180926616915 1.18041292190311 1.64979301174167 8.21112539796336e-15 1.25390440890172e-13

MTND4LP13 0.0544137806965174 0.138236785570934 1.34509759976027 1.45203922719266e-05 4.27530120182143e-05

AC005076.2 0.0817748983084577 0.231879359238754 1.50364443872339 1.30521617670415e-06 4.69335556571897e-06

CDCA2 2.64240810995025 1.05186805574394 -1.32889955543565 8.41140733592292e-35 1.71680590027979e-32

AL033397.1 2.85083613952736 1.04587666106574 -1.44667239199258 1.9546174161948e-05 5.59367859505365e-05

B3GAT1-DT 0.523102385970149 1.48021647964014 1.50064292791342 1.6196616677742e-15 2.75312284733526e-14

AC131097.1 0.150725862885572 0.0598670429065744 -1.33209307250171 0.00373750148489748 0.00672106421276521

TEX101 0.660727289109453 0.1487334003391 -2.15132626878406 0.00495246018024471 0.00867989656710624

FAM182B 0.153241070104478 0.330635666038062 1.1094393526907 8.19216463017536e-09 4.45704600428279e-08

AC007151.1 0.0239595375621891 0.134098996193772 2.48462646953535 6.08701323191533e-06 1.92239967543746e-05

HBQ1 0.642457654427861 0.210575699757785 -1.60926240645469 6.05157752866662e-06 1.91209155971617e-05

SCARNA6 3.40596511243781 18.7669267202768 2.46205685060292 0.00384693139859271 0.00689925073780398

C10orf71 0.0542336158855721 0.114707478176471 1.08070018206993 3.52783285436551e-09 2.04117259502637e-08

AP001264.1 0.0447427860696517 0.0902742385121107 1.01265925193567 3.96589275843022e-10 2.72188624700292e-09

LINC02253 1.0647297160199 0.370154455916955 -1.52428794466217 0.000602436598016655 0.00128043526233723

MTND4P23 0.0525047402089552 0.130118294629758 1.3093042367068 2.49528525315901e-07 1.03525205453569e-06

LINC02315 1.65644748293532 0.459504492214533 -1.84994159323048 5.67120300565943e-07 2.1932607777826e-06

BX324167.2 0.0353303307910448 0.0798781342110727 1.17689337782046 7.18588484086319e-11 5.66054004601406e-10

AC009229.4 0.0103360718159204 0.026339398266436 1.34953438790523 0.00129240839913276 0.00256326103816396

TCTE1 0.167357062427861 0.493329678619377 1.55962264850824 1.61436235462926e-06 5.70670937044207e-06

DRC3 0.585050849303483 1.26945022249135 1.1175698992005 1.90391711900109e-16 3.77061065928167e-15

AC009951.4 0.0258773483980099 0.192777590982699 2.89717565848807 0.00199608598379482 0.00380836774724718

PWAR1 0.0150366768059701 0.0388016093391003 1.36763073285227 4.22352420706973e-10 2.88855681578787e-09

RASGRF1 0.93398084800995 2.67337066065744 1.51719500814953 1.64065705959175e-10 1.20753419213763e-09

BUB1 7.03179732985075 2.6085849266436 -1.43062608641961 6.81119770321204e-46 1.09580151284029e-42

RNU2-5P 0.124146503532338 0.296923320276817 1.25804677669109 2.6032683607531e-05 7.27489421340526e-05

CR382285.1 0.336311955562189 0.0332979259792388 -3.33629584180691 0.00382118183807158 0.0068553180236968

AC011611.5 0.145842970447761 0.304771226747405 1.06331085457137 0.000454796271419925 0.000990104117116528

KCNJ13 0.0241630386716418 0.118304953148789 2.29163667696594 0.00494378809206283 0.00866803027873059

AC010595.1 0.202039998079602 0.0681371228442907 -1.56812799852497 1.00382087319023e-12 1.08687651946765e-11

UHRF1 4.92829420497512 2.2286634349481 -1.14490962111743 6.29751304834931e-28 5.66569019316953e-26

LINC02512 0.0256185862189055 0.192126412491349 2.90679309971368 4.14336116311921e-11 3.41533839093762e-10

RNY4P25 0.165819599502488 0.458429645432526 1.46708580217302 0.000155291116842164 0.000371909986482766

TMEM212 0.126023290328358 0.39866358349135 1.66148144383861 1.41860681587112e-06 5.06976302287667e-06

RAB5CP2 0.0413673449253731 0.0875274380276817 1.08124297842796 0.00341603123880077 0.00619511003124468

FOSL1 12.1211824129353 3.96456744757785 -1.61229506456833 5.08190552999403e-10 3.43100755974665e-09

AC012485.2 0.216109738208955 0.460111104602076 1.09021819221236 9.47425398388444e-05 0.000236726517868847

AC104809.1 0.0439012483731343 0.18266406633218 2.05685898512436 1.59666689079917e-11 1.40414274801792e-10

CA4 0.464177275820896 1.43284259404498 1.62613232879693 3.77248697228597e-08 1.81842648382131e-07

TBC1D27P 0.120540884258706 0.344910097906574 1.51669781388461 1.08347781475609e-09 6.91057776557614e-09

AC243967.2 0.554135581691542 1.12714869816609 1.02436694216524 3.14627196058345e-08 1.53744038095332e-07

AL355102.1 0.0584827668159204 0.157943499723183 1.43332509134157 0.00115936695320037 0.00232315086599972

AP001189.1 0.173197931587065 0.366028981584775 1.07953618248976 2.07550035502909e-14 2.95958992231729e-13

DUOXA2 0.893446527417911 1.95148753291003 1.12712095416434 9.09660658869172e-07 3.36934168744202e-06

LINC02732 0.399533848258706 0.0811286335640138 -2.30003463790763 0.000260932780728244 0.000597720785277164

AL137856.1 0.0592494210696517 0.120956115404844 1.02961074968121 1.45653459179007e-07 6.31619170532082e-07

KLRC2 0.399817990830846 0.169464716083045 -1.23835846737184 3.47170285762366e-06 1.14702915143763e-05

AL513008.1 0.18494183721393 0.542107947923875 1.55150853306951 2.45178838771742e-08 1.22053899534167e-07

AC007494.3 0.0381093965671642 0.161975317024221 2.08755531202296 4.91330214907849e-05 0.000129859696344508

MC2R 0.0105496377512438 0.0219974084809689 1.06014010840958 0.000146895442709044 0.000353692257953372

EPN2-AS1 0.0630056099004975 0.141630744861592 1.16858228190304 1.28593155970038e-05 3.82742715832032e-05

CLSPN 2.41657852189055 0.952529519515571 -1.34313016454148 1.00235139430679e-27 8.81489087919315e-26

SDR42E2 0.0446276959502488 0.0950799719307958 1.09120215267744 1.63935852416598e-11 1.43844900981519e-10

ANKRD66 0.142701004363184 0.414615162536332 1.53877738810198 2.51876785207459e-08 1.25092247601671e-07

UBE2C 53.8604872537313 18.5841715266436 -1.53515290638104 1.8555221255362e-36 4.57193965165902e-34

AL031736.1 0.333106152039801 0.159706251245675 -1.06056121493306 1.73871914124906e-05 5.03163353223593e-05

GREB1 0.565933291243781 1.22330917065744 1.11208515454499 9.76438504391862e-05 0.000243021140186709

DPYD-AS1 0.368186620810945 1.06474331471626 1.53199656355113 2.30977699091128e-06 7.89556314228515e-06

AC008543.5 0.00445995937313433 0.0124029951799308 1.47558608340274 1.30104219295138e-05 3.86777217143698e-05

POLD2P1 0.0915723125870647 0.0313918219377163 -1.54452269378412 0.000616456937261904 0.00130799823383344

AL109659.3 0.0293964813432836 0.0683821204152249 1.21797567921592 1.8523885140184e-09 1.12784563353525e-08

AP001094.2 0.131673924258706 0.263904736242215 1.00304756953644 2.42785961009082e-16 4.68655093411708e-15

SLC5A9 0.231545144323383 0.708728155778547 1.61393886144232 3.72379540654124e-20 1.24505873311617e-18

CGA 9.40467854159701 4.29526132978547 -1.13063272263452 0.00505034818844774 0.0088373015325685

MOB4P1 0.0183487933333333 0.0528362690657439 1.52584340634163 1.93086720197204e-10 1.40637065176925e-09

PGM5P4 0.171049667313433 0.54628104934256 1.67522807826059 3.39723287963929e-16 6.4034679020079e-15

AC005838.2 0.194315714975124 0.401837736539792 1.04821047245978 4.33976443665786e-08 2.06997832826286e-07

SLC5A11 0.211734592373134 0.0756797112422145 -1.48427850133675 0.00281132695503968 0.00519907758522097

LINC01322 0.205933807686567 0.0493620698927336 -2.0607058961925 3.47461962884035e-09 2.01362163323965e-08

SYT1 2.55966618119403 1.11173695200692 -1.20314020097386 1.46835817253292e-06 5.23183898108069e-06

MIR146B 0.260678567960199 0.522019905882353 1.00183284666551 1.67998768404299e-06 5.90814750656754e-06

LINC02122 0.268652493383085 0.745533571245675 1.47253209041209 1.85329891614238e-09 1.12814879493644e-08

LHFPL3-AS1 0.0140233428656716 0.064631269449827 2.2044020304167 3.54539529543352e-12 3.49429049838223e-11

AC010894.2 0.389705314860697 0.134573208871972 -1.533992383679 0.000284638656390552 0.000646368918322949

CDH15 0.689550687363184 1.38532748442907 1.00649855231408 2.43423311067751e-13 2.93416816117364e-12

C16orf89 39.3859927134328 177.939581276125 2.17563291856008 2.17152558082498e-33 3.80712978433097e-31

TLK2P2 0.0262277733432836 0.0572286781349481 1.12564295151471 1.56574675119833e-08 8.07679623637765e-08

SCUBE1-AS2 0.034691868159204 0.124224130519031 1.84027600703521 2.10487281593298e-07 8.8552948032252e-07

LINC02321 1.09938973746269 0.47538714200692 -1.20952812959196 8.26387836699066e-11 6.43923285860953e-10

CDA 27.0740401905473 12.345560329481 -1.13291787560459 0.000250678208925545 0.000576526153221801

AC091053.2 0.0523387842288557 0.106726041107266 1.02795991836999 0.000244510144163783 0.000563430149370584

AC022001.2 0.0185304543781095 0.0760977295155709 2.03795515193785 4.7150594861778e-07 1.8544273360219e-06

VSIG1 8.09758233910448 22.4769947917647 1.472886019896 7.48182349463594e-06 2.32769733338975e-05

CKS1BP3 0.367627749950249 0.170182284705882 -1.1111648048179 2.13049522195243e-10 1.53947276936325e-09

LINC00634 0.260181846820896 0.106027323491349 -1.2950842085002 1.16461315481812e-12 1.24666026552938e-11

AL356752.1 0.0730029974626866 0.181368523633218 1.31289649240201 1.37094440407315e-12 1.44490672259733e-11

MIR200B 0.309204016119403 0.85062754 1.45996850661834 4.30105871887973e-16 7.9751834550075e-15

AC005740.3 0.117523031343284 0.238129475743945 1.01880269512207 6.33409393024251e-09 3.51180760170551e-08

PRDX2P3 0.0608982989054726 0.129242726228374 1.0856092535714 3.06696635350881e-06 1.02369452975916e-05

LINC01214 1.83706199925373 0.630187244290657 -1.54354785901205 1.89689433278232e-07 8.0571610501004e-07

AC009090.3 0.292505889402985 0.624012658304498 1.09310962228211 1.88924114838181e-20 6.55842158976193e-19

FOXP1-AS1 0.049766883880597 0.112400914186851 1.17539580784108 0.00301162216977894 0.00552804472103718

CASC19 0.888576165074627 0.305475270449827 -1.5404398510672 0.00329239219476121 0.00599473982932001

AL591686.1 0.0227171885074627 0.0782064975086505 1.78350417658934 1.41149631186302e-07 6.13840421838982e-07

RN7SL823P 0.0445475258706468 0.164614278927336 1.88567226994583 3.51834059165079e-08 1.70554085752657e-07

AL096803.3 0.0304686582587065 0.0694205633564014 1.18803710306795 2.39471266734481e-07 9.96430723442576e-07

MIR29B2CHG 0.316831167910448 0.88615843349481 1.48385039087123 5.92853649815515e-25 3.79731787411109e-23

GLDC 2.5191764139801 0.834411567041523 -1.59412109279648 3.00197805817794e-08 1.47219114023967e-07

PPP1R14BP2 0.636018467562189 0.268659177128028 -1.24329153660349 2.60296011721115e-06 8.79878373572178e-06

VIPR1-AS1 0.0797841993482587 0.275389129608997 1.7872966482199 1.11557000868232e-33 2.00729208799088e-31

CD300LG 0.0392913664328358 0.232248149778547 2.56338285704432 6.06723976598288e-17 1.29842728951199e-15

RN7SL262P 0.142290896218905 0.307156128269896 1.11012880663617 3.13775002369114e-09 1.83409837888337e-08

KRT16P1 0.378590439597015 1.13288354216263 1.58128967825455 2.62895694367238e-10 1.86274539920828e-09

MEG3 1.19203522791045 3.28767281795848 1.463639860268 4.61784513613173e-08 2.19077301774853e-07

AC046158.1 0.0268371785074627 0.117837800034602 2.13449749271345 0.00391589820315625 0.00701328111167071

MND1 3.44819548059701 1.63956105017301 -1.07253194166526 9.04069762346024e-27 7.08490200577758e-25

PTPRN 1.10974038281095 0.534058891622837 -1.0551514617464 1.08736089958242e-08 5.78136092604572e-08

RHCG 2.83490977361194 0.131729848986159 -4.42764862786829 2.11146424616679e-08 1.06527480414429e-07

RPL21P32 0.0296198892537313 0.0791821859861592 1.41860964994276 8.33794267561377e-06 2.57442687037747e-05

SGO2 2.86137596368159 1.22840785813149 -1.21991942321162 1.40189416660586e-42 8.71404669469779e-40

AFF3 0.339885907606965 0.833476231211073 1.29409051160354 5.9072475080355e-21 2.20413668956031e-19

AC024337.1 0.197565526666667 0.0729070024221453 -1.43820194078334 0.00284016055143207 0.0052460586939736

DNAH9 0.155149210992537 0.624096131214533 2.0081119083265 3.3327632880449e-09 1.93877281278559e-08

AL355076.1 0.0585594231840796 0.151421051418685 1.37059254284447 5.77643746954152e-07 2.23111940110098e-06

RNU6-1010P 0.383884041243781 0.874472092249135 1.18774175698138 0.00281513061530167 0.0052050988594173

SCN4B 0.612486767875622 1.49938905183391 1.29162419142186 4.63751715555417e-27 3.73047335895313e-25

AC084855.2 0.0161909897910448 0.0335086337058824 1.04934167925295 3.81209702495414e-09 2.18897446215611e-08

AL353662.2 0.0818741317412935 0.188738040761246 1.20490562484737 0.00274666913490868 0.0050922858486817

LINC01213 0.283470628557214 0.109462799377163 -1.37275860263896 2.30712789985724e-09 1.37953537518792e-08

CKS2 72.6147500845771 32.652565 -1.15306630351047 3.66803014822228e-32 5.33620343371699e-30

AC011379.1 0.0159129202189055 0.0616836115640138 1.9546886247931 3.78088580363837e-06 1.2416813968481e-05

AL138847.2 0.014150226278607 0.0498119134532872 1.81566570752147 0.00167314483048438 0.00324014098376746

FCER1A 2.87670523383085 7.5483370933564 1.39174145158588 7.90559559367047e-22 3.37840686698262e-20

B3GNT8 6.46533701791045 13.5429394636678 1.06674342620631 4.97066983603911e-21 1.8881641668843e-19

AGER 17.2152515228856 52.4699826972318 1.60780505471847 6.34503850158159e-12 6.00265662463703e-11

RPS3AP34 0.0975565880597015 0.29602446100346 1.60140518574975 5.83906700084204e-18 1.44002238478837e-16

ITGA9 2.71334487910448 6.13588504740484 1.1771990222468 2.36786632453531e-28 2.22546886515604e-26

FANCI 6.27951466666667 2.95770671384083 -1.08617405799714 2.68613868056892e-36 6.44437657136491e-34

AC020951.1 0.0855068507562189 0.177540637813149 1.05403736766398 1.77451651591625e-06 6.21580772416873e-06

BNIP3P26 0.0690600124875622 0.173961879896194 1.33284870411841 5.53764065726885e-06 1.76376466724471e-05

C1orf189 0.906378755024876 1.85832714550173 1.03581854969438 5.65250410568349e-05 0.000147487108653352

RNU1-134P 0.137523234925373 0.31420970384083 1.19205235005206 2.26376834713316e-05 6.40733357074324e-05

AC109454.3 0.479843366716418 0.120331913356401 -1.99554423774352 0.000338321973964193 0.000756158044285419

RPL21P135 0.053060126119403 0.112689331349481 1.08665092768871 2.55388448579244e-12 2.57649357013734e-11

RNASE13 0.0107791019751244 0.0226927233044983 1.07399276432358 0.000354790950432644 0.000789546988961173

TPSB2 6.5983553320398 13.9265350661246 1.07765798226613 9.21778153959696e-11 7.1276880154927e-10

LRRC31 1.43244875328856 3.11238902718685 1.11953887190921 1.54588351622691e-10 1.1433183928828e-09

LMNTD1 0.0422412545920398 0.113569971989619 1.4268568458955 0.00164436870670871 0.00319209909351148

AC116036.1 0.0221872769651741 0.0653252940138408 1.55790909572605 2.90185554539153e-09 1.70636625787908e-08

AL021026.1 0.0460599407960199 0.0985669918685121 1.09759204077495 8.78768368366207e-09 4.74986459976596e-08

AC007000.3 0.0923941408955224 0.199455081591696 1.11019060773319 0.000322084030345467 0.00072305657308943

HSD17B13 0.662218632283582 1.74765197792388 1.4000384112524 4.69737112077833e-17 1.01962777899434e-15

AC006254.2 0.0756611994029851 0.154678179377163 1.03164413685098 0.0034191005722044 0.00619946526936043

AL133320.2 0.0697985783532338 0.193846445079585 1.47364472071227 0.000144930717881917 0.000349330671901862

NUS1P2 0.599890328606965 0.287549175294118 -1.06089007265019 0.00210997185320091 0.00400470022103018

LINC00427 0.0657626848258707 0.135147861141868 1.03919757510588 2.52426669423764e-05 7.07871363553773e-05

IL20RB 5.88734185855721 2.63800277128028 -1.15817032246048 1.07295291673794e-06 3.92473749803165e-06

AC105074.1 0.0209614979900497 0.0596473496470588 1.50871621390409 5.78874910602918e-07 2.23555899533886e-06

SCN7A 1.06574505827363 2.86933366539792 1.42885337841365 1.08896772478062e-25 7.54006766398733e-24

CASC9 4.07424040300995 1.82387823816609 -1.15952169128972 1.70844835772678e-10 1.25338150707692e-09

MIR374B 0.196609621940299 0.574845578235294 1.54784052651738 6.12601424485658e-12 5.81758644433429e-11

SNTN 0.544894518512438 1.46501224810727 1.4268638432356 3.42143364421706e-13 4.0282483929977e-12

AL139246.1 0.0698876578109453 0.189646223391003 1.44020103946252 1.11861876055267e-09 7.11162787101709e-09

GUCA1A 0.205136019661692 0.0938547891107266 -1.1280785674968 1.36976948521095e-13 1.72165420131064e-12

AL645924.1 0.512057655970149 1.14194381878893 1.15711350782004 4.47694130032725e-06 1.45127823354214e-05

AC022809.1 0.0934522412935323 0.0314997836678201 -1.56888734438066 5.32666528228379e-11 4.30891143065548e-10

AC009754.1 0.0995314339800995 0.31944182349481 1.68232907841759 4.01309030412339e-06 1.3119533805615e-05

DYNLRB2 0.545718723830846 1.25562382827336 1.20217486353627 8.8902781237746e-06 2.72973141240817e-05

ANTXRL 0.0193778180746269 0.0560063741764706 1.5311848982843 1.55033982489099e-05 4.53398141689142e-05

TMEM132E 0.119201671900498 0.263640442314879 1.14516722512102 2.8540406837824e-20 9.69664754055263e-19

AC007342.6 0.0862041870447761 0.179427990356401 1.05757511531761 1.79654630369919e-12 1.86331215040473e-11

AC090236.2 0.0868781181293532 0.196594087197232 1.17815517323459 8.34482378745108e-12 7.75504351297272e-11

SPAG8 0.438097833681592 1.05098435588235 1.26241620873601 1.84777202556195e-12 1.91353899655885e-11

AL355994.2 0.0949000577114428 0.0473266136678201 -1.0037572670956 1.16276959086047e-07 5.14092277886095e-07

LINC00470 0.304655308383085 0.0903864232906574 -1.752999887859 0.000105474473708091 0.000260990396807771

AC115099.1 0.150856266268657 0.463277957785467 1.61870341788287 3.1845582511024e-17 7.0926439875937e-16

AC010255.2 0.0208861001691542 0.0627087916816609 1.58612458192271 0.00425759439091741 0.00757268690847312

AC079949.2 0.974145578164179 0.269953970204152 -1.85142395289255 4.01855778659243e-07 1.60238453802745e-06

SUN3 0.993688024920398 0.458012147204152 -1.11740711762807 1.02093900139e-05 3.10010899167498e-05

AC007598.3 0.0246356723781095 0.053525131183391 1.1194675790098 7.07606242015237e-06 2.20925008209095e-05

AC090519.1 0.0345976643781095 0.10896440017301 1.65511031549749 2.67157599947071e-05 7.44524185709435e-05

SLC24A5 0.00429714617412935 0.00928880822145329 1.11211465344138 0.00549452014650931 0.0095309904234813

LRRTM2 0.0653957952835821 0.149439210851211 1.19228895801908 1.68776479139605e-06 5.93274966186118e-06

AP003472.1 0.239163669154229 0.106063245813149 -1.17307344492193 3.64693585157955e-05 9.87659130019811e-05

LINC02577 1.53307320846269 0.663596429401384 -1.2080485625482 9.72243277213346e-08 4.35691804000055e-07

AC072022.1 0.123394076766169 0.276926704290657 1.16623103772296 1.67334151836458e-06 5.8878027180331e-06

FSD1 0.519317216084577 0.189392840391003 -1.45523425985454 8.36586856102766e-08 3.79385172176385e-07

SHISA5P2 0.0264568523383085 0.0683105080622837 1.3684660928496 0.00043524338591331 0.00095185563767224

FHL1 3.69260399402985 7.64184035051903 1.04928156607256 2.66625232538602e-15 4.37707089431619e-14

BCRP4 0.0243767596517413 0.0517082865051903 1.08488913351564 0.000683705088563642 0.00143653177938201

CDH16 0.228980274144279 0.623208984674741 1.44449271207916 0.000145609192831709 0.000350797467437933

AC134775.1 0.107704607164179 0.227213101626298 1.07696606251501 5.15169422374909e-09 2.89736452847085e-08

KYNU 4.04993511373134 1.48164424307958 -1.45069970958142 1.26174485948063e-05 3.76445095525202e-05

IGSF9B 0.344817463716418 0.998088961051903 1.53333556730956 2.73594190973837e-20 9.30696657106274e-19

CACNA1D 0.778072242895522 1.74142767927336 1.16229454096195 5.59589576246737e-18 1.38254515901972e-16

TCTEX1D4 0.364701819701493 0.855598408062284 1.230216400103 4.85913644671053e-08 2.29529156852389e-07

AC132812.1 6.71745970099502 13.8168858892734 1.04044482494529 1.76487814355652e-16 3.50795183330457e-15

CAV3 0.0177233267960199 0.0689030244567474 1.95891787699271 8.34492429228857e-15 1.27291511095422e-13

EME1 2.14335215074627 0.992104821453287 -1.11130444030804 5.90843782926417e-31 7.48128586251736e-29

ICAM5 1.49048518895522 3.63505272816609 1.28619425202411 1.16999873173252e-19 3.67387661456767e-18

HMGA1P3 0.526808212985075 0.235561519238754 -1.16117395427271 2.11599850659209e-18 5.59155160920712e-17

AC133065.3 1.82923258701493 3.9246719100346 1.10133352887733 1.02938581380231e-21 4.32468540821708e-20

ERVFRD-1 0.0487401066567164 0.106696897280277 1.13033691036312 5.44201829961399e-12 5.20053111441099e-11

CXCL10 51.7441833338308 23.3535375629758 -1.14775559149384 3.88673893048752e-11 3.22225855558756e-10

PLIN5 0.559048390895522 1.18591433 1.08495472149825 1.71768793625854e-16 3.42910693844314e-15

ELN-AS1 2.2036290219403 4.46640572048443 1.01923294047733 4.43151744602557e-16 8.18379487837943e-15

FPGT-TNNI3K 0.0414741876766169 0.100324052650519 1.2743819033651 3.20184621700306e-20 1.08111721030906e-18

HOPX 29.1659307094527 61.6133245190311 1.07895826989167 1.14265186895746e-18 3.12828114274139e-17

AC009275.1 0.896910296467662 0.32720152350173 -1.45478423703446 7.37191448402141e-13 8.16613451348666e-12

AC105206.1 0.00915169815422886 0.0555906274429066 2.60273029131412 3.1155034895954e-08 1.52376645995054e-07

NBPF4 0.0799836136218905 0.018896733449827 -2.08156759581099 3.20895509710324e-06 1.06731025058706e-05

SULT1C2P1 0.0712361734676617 0.162106349318339 1.18625867125619 1.21562657892988e-09 7.66775528914488e-09

B4GALNT1 1.2649986839801 0.533779152941177 -1.2448210176478 1.01857398962437e-07 4.5475022226945e-07

NAPSA 223.234855914478 614.786120486851 1.46152228755999 2.15510903980353e-30 2.52970953813848e-28

LTF 18.6758561045274 42.3687933612457 1.18182766424102 0.000240712176804552 0.000555614654030255

CLEC3B 4.82320987462687 11.6850420512111 1.27659743273336 9.43142418890997e-16 1.65352212542749e-14

AL359881.3 0.0248254785621891 0.0550365165086505 1.14856762978811 1.46490301563992e-06 5.22088838646753e-06

AL035665.1 0.184562777313433 0.391760436020761 1.08586008777651 8.08555520218496e-13 8.89183493284112e-12

ACRV1 0.196848687139303 0.0955855352387543 -1.04222287085204 8.13573359686063e-17 1.71031755475894e-15

DEFB1 27.7789328913433 9.091310836609 -1.61143094978357 1.63579625990626e-05 4.76462255225717e-05

CREG2 0.472419264383085 0.123594714792388 -1.93445074492027 1.1959296864889e-12 1.27868166245002e-11

CIP2A 3.53943076368159 1.36762758892734 -1.37184192310362 2.4484621810671e-40 1.01462788866947e-37

RNU6-403P 5.45844605472637 13.0497383463668 1.25745868127055 5.04840975577571e-13 5.73873677558045e-12

LIVAR 0.2671758860199 0.130043555778547 -1.03879489387193 0.00106758361728024 0.00215565979568952

AC006270.1 0.235149192189055 0.622854288615917 1.40531832057438 0.000184435809251632 0.000435831984018674

AC113349.1 0.116396632587065 0.495678316055363 2.0903548270893 2.5473932331306e-06 8.62801299394699e-06

AC007496.2 0.0360375175621891 0.113027430622837 1.64910140283556 0.00467870086696502 0.00824712997624989

AC025423.5 0.0776250547263682 0.373287180968858 2.26569168043456 3.33104889801093e-05 9.08226371853244e-05

AC103702.2 1.39102328341294 0.647599173460208 -1.10297351897171 4.57605337454219e-08 2.17396317168193e-07

RBMS3-AS2 0.0187297200049751 0.117346072564014 2.64736831968658 1.38928178587871e-06 4.97117761698914e-06

AC022973.4 0.0741676860199005 0.378374425536332 2.3509519159453 0.000507181592164263 0.00109275378491355

MIR924HG 0.574182667427861 0.183535083892734 -1.64545391155367 1.58352338764232e-23 8.42594643035359e-22

AC090337.1 0.0360388322288557 0.111513270072664 1.62959123180813 1.56157080959533e-08 8.05981536939654e-08

GIP 0.330688211044776 0.0815935244982699 -2.01894504937723 2.47446404227668e-09 1.47283115465217e-08

AC104088.1 0.403846869452736 0.145609460795848 -1.47170426002689 1.16093027028298e-05 3.4818996482333e-05

AC022960.1 0.0796550616915423 0.173100435363322 1.11977140719287 0.000137755814689089 0.000333476857120427

AC025161.1 0.106462696119403 0.0526308247058824 -1.01636810080797 3.69704831347729e-08 1.78457944535129e-07

TGM5 0.225557797661692 0.0900856176366782 -1.32412846155147 1.06710266748193e-05 3.22738670304443e-05

LRRC77P 0.0141110371094527 0.0328153782110727 1.21754803624167 5.73926120418681e-06 1.82119496385322e-05

TMPRSS11CP 0.0813301195522388 0.20277485550173 1.31801712656718 5.0439234678764e-10 3.406205107319e-09

RNA5SP527 0.0889664271144279 0.19439773799308 1.12767851173693 0.000353606972925548 0.000787424744301721

AC133065.1 0.0468993666169154 0.11122949100346 1.24589900579578 0.000140547430469154 0.000339423595879149
