# Supplementary material for: Characterization of polyamine metabolism predicts prognosis, immune profile, and therapeutic efficacy in lung adenocarcinoma patients
Source: Front Cell Dev Biol. 2024 Apr 8;12:1331759. doi: 10.3389/fcell.2024.1331759 (PMC11033315; doi:10.3389/fcell.2024.1331759)
Supplement: Supplementary file 11 [file Table4.DOCX]

id HR HR.95L HR.95H pvalue

TMEM171 1.17318435338345 1.09547354556754 1.25640781796396 4.93051970852251e-06

STEAP1B 1.0733735145997 1.01906786207223 1.13057309009952 0.00751720757105255

SLC6A17 1.41017212505921 1.19184242154431 1.66849693075811 6.20549194673187e-05

MCM4 1.01796363938542 1.00671825785439 1.02933463561032 0.00168161096900414

CASC8 1.27816274835852 1.11964115470533 1.45912822552629 0.000280504141693503

GAS6-AS1 0.710539411788634 0.580515940916498 0.869685430012262 0.000919762742994863

SLC10A2 1.03890917546806 1.01505047983579 1.06332866819228 0.00128108487948608

CENPM 1.04059798205993 1.01201618828232 1.06998699507474 0.00510167913223162

ESYT3 0.846344411191514 0.762999025810717 0.938793940914948 0.00161024475666339

AC005884.1 0.237800717623125 0.0919760125340338 0.614825319603288 0.00304062306834169

CCNA2 1.02948505049055 1.01366449819246 1.04555251868187 0.000235438678701584

BIRC5 1.01893390710116 1.00625800209624 1.03176949140042 0.00331718209089207

AL353152.1 0.176667271820829 0.0570262924077375 0.547314644084765 0.00265850129488465

TERT 1.25294034592101 1.10073608155922 1.42619065254309 0.000643846053532667

MGAT5B 1.14682684204022 1.05904205270464 1.24188817834484 0.000746697207126217

FOXM1 1.02660197635894 1.01187839883421 1.04153979280346 0.000367923125033489

SLC2A1 1.00669416019441 1.00429267343266 1.0091013894442 4.37221519610268e-08

GRIA1 0.317379268582125 0.158163294668253 0.636870901918202 0.00123932336878579

RAB44 0.107340853965087 0.0241459894246453 0.47718313494306 0.00336846930639842

TPX2 1.01105149741507 1.00489666748508 1.01724402468518 0.000418920681340226

KIF4A 1.06146719917751 1.02987826084401 1.09402504914162 0.000108872779773949

AC239859.5 1.17762472707829 1.09446565745392 1.26710234202538 1.20993964876631e-05

TBX4 0.890290620752033 0.82407772656121 0.961823580290841 0.00320753764353479

CDCA5 1.0397894615781 1.01842324488633 1.0616039351395 0.000230265851379657

AC013726.1 0.0387797829328071 0.00336987234180981 0.446269594743156 0.00912698194387517

ABCA9-AS1 3.3308650496528 1.75703935072342 6.3144072296563 0.000226802603701277

FGF12 1.16169121249826 1.08741789494504 1.24103758037189 8.74712425830376e-06

TESMIN 1.15706895465196 1.06446110830336 1.25773366013653 0.00060883099613492

CTSV 1.02211324910331 1.00577234953299 1.03871964115699 0.00781563673446871

APCDD1L 1.0628111027402 1.01675608771959 1.11095222713766 0.00703551245057754

ABCC2 1.01836580965413 1.00810372404415 1.02873235911893 0.000428544532361284

ERCC6L 1.22755718095401 1.0831752604603 1.39118449942384 0.00132077419252014

MAMDC2 0.921346965166948 0.867519184806242 0.978514648539953 0.00765068101736389

AC011458.2 0.186884888263789 0.0543097100180789 0.64308871194014 0.0078108177442942

TXNRD1 1.00221621271578 1.00108512892382 1.00334857447153 0.000121847516754995

CCDC13-AS1 0.111290185830336 0.0219396627763776 0.564525790044783 0.00804718884662429

AC022497.1 0.57430268111159 0.408128909438323 0.808135767657018 0.00146104270144216

HTR1D 1.10214374255043 1.03802210851244 1.17022635576024 0.00147180858792948

DEPDC1 1.07368375241757 1.02141958207016 1.12862218469421 0.00523250571658341

KCNV1 2.15566804398125 1.57020730728732 2.95942115049122 2.02758019668822e-06

ROBO2 0.607444830916387 0.429311162330899 0.859491331657089 0.00487765024123794

NEK2 1.04608408924196 1.02162121248481 1.07113273333823 0.000190171141300014

SKA1 1.07205381895676 1.02064729278714 1.12604951667615 0.00551824123653844

INMT-MINDY4 3.30127592814379e-05 4.48620369615272e-08 0.0242931964125656 0.00218567462337984

MS4A4E 0.439733026808996 0.238757110192675 0.809882205018134 0.00837193510047951

AC010343.3 1.36024564155648 1.12549803696231 1.64395507109655 0.00145687322653302

ESCO2 1.37582311497479 1.13027165128477 1.67472062273464 0.00146858611441757

MELK 1.03582863877075 1.01185291645945 1.06037246268159 0.003217701598353

GCLC 1.0062543846865 1.0018258005948 1.01070254539226 0.00559643961099322

AC025278.1 0.00872735635392114 0.000308731834107604 0.246708439213887 0.0054223166111753

SLAMF9 1.14814442066043 1.05963351738615 1.2440486159266 0.000737910007467866

AC007671.1 0.142465078444607 0.039617393180167 0.5123077756259 0.00284295060315518

C5orf38 0.956895257918966 0.927636987913406 0.987076352666178 0.00541972342597941

ZNF540 0.4331468328051 0.271377097839357 0.691348607759635 0.000452822137938727

IGF2BP3 1.06218626673231 1.0214835925167 1.10451080516624 0.00247651457979984

HPSE2 0.375315724182185 0.195286892169764 0.721307463359834 0.00328139659256725

C7 0.987983940904387 0.979268685207448 0.996776760280232 0.00749272445551771

PRSS3 1.02247461279422 1.01016805354065 1.03493109898334 0.000321358127557249

RNU6-247P 0.851179144093506 0.754701270449479 0.959990348112517 0.00865958772370523

SH2D5 1.35268744237316 1.09557400206593 1.67014123491763 0.0049759383424606

FAM111B 1.05508554526543 1.02077135862131 1.09055323547829 0.00147957727279693

ARHGAP11A 1.08593177801629 1.04293922629532 1.1306965897663 6.33801334453536e-05

AHSG 1.70532132615841 1.24553545310036 2.33483584767646 0.00086953666563183

AC114684.1 0.0489297562473047 0.00562027358795223 0.425979449034787 0.00627859646548015

KNL1 1.21919267039416 1.09162502279485 1.36166791389336 0.000440351315450528

NTRK3 0.0306748826323321 0.00225760935412859 0.416789743888417 0.00886057059119249

LINC00592 1.36280659944776 1.17483009906012 1.58085992943505 4.3577232168332e-05

PRR11 1.05972318594484 1.02569528770988 1.09487997486709 0.000494789166723887

ADH1B 0.974116012530429 0.95570049748474 0.992886378489444 0.0070793699326488

E2F7 1.33771175774008 1.20769769736288 1.48172241340158 2.43967611107663e-08

LNCAROD 1.0629561167244 1.01607874684521 1.11199620067827 0.00797530476021794

EXO1 1.08015647889113 1.04240760624101 1.11927235747811 2.15383176336328e-05

PLOD2 1.01017871665564 1.00484772069474 1.01553799502924 0.000175921615673651

APCDD1L-DT 1.26565522083427 1.0779924858018 1.4859872950168 0.00401319642065458

FBXO5 1.09919251815556 1.02880325395141 1.17439771630643 0.00509536399352767

LINC01910 14.8254154442662 5.32366761238147 41.2860003851303 2.46996570841088e-07

AC093648.1 0.315120832152946 0.135690796169606 0.731819266007136 0.00722612874761899

CACNA2D2 0.984052039367745 0.972253565290904 0.995993690076189 0.00899478288137215

CDC25A 1.13690768801724 1.04999297047849 1.23101689955475 0.0015657013394231

RHOV 1.00487210449629 1.00286041009191 1.00688783427224 1.99826935227468e-06

UBE2S 1.02883741380714 1.01281098148235 1.04511744382957 0.000386510114833462

ITGB1-DT 1.04272060458598 1.01237117120319 1.07397987038287 0.00550652824766995

AKR1C7P 1.10183068495827 1.02927390429926 1.17950222311537 0.00526831130579862

PNPLA1 1.94632756182231 1.19811683297939 3.16178762674503 0.00714269049536862

ATP13A4 0.966481188381379 0.944532906135634 0.988939486837687 0.00362668377682187

AL391807.1 0.0780246612840098 0.0131666320544091 0.462369400415181 0.00495937374893072

LINC02802 1.33836612529648 1.19300120874808 1.50144347902279 6.7577541744967e-07

CHEK1 1.08731296147709 1.03674078819683 1.14035204330326 0.00057147324799455

AC087763.1 0.448182632801227 0.281616779816987 0.713265993862922 0.000711082996124937

SFTA3 0.980856723038961 0.969401476775393 0.992447333927098 0.00126045225678

CENPU 1.03835850898699 1.01241800154351 1.06496367265487 0.00354476535669368

LINC00968 0.31616605686 0.154364512767874 0.647564480449711 0.00164459702873696

NR3C2 0.878406276877725 0.805457058900009 0.957962412436905 0.00338049819333337

AC006539.2 0.348616395227297 0.166801998685564 0.728608721591975 0.00508207345746231

AP002358.1 0.274979286329296 0.103853888984496 0.72807680722921 0.00935640943950193

PRC1 1.04395656533979 1.02260710239737 1.06575175134325 4.49386630070277e-05

AURKA 1.02356552488281 1.00894373886409 1.0383992123368 0.00150940436699153

HMGA1P1 2.63578690507671 1.26319005375725 5.4998632931834 0.0098077491713688

AL049555.1 1.07745115397675 1.03739126911412 1.11905799072049 0.000113901264619426

ZIC5 1.79598363376678 1.35489559768115 2.38066845761292 4.65706846083211e-05

CRABP1 1.00343594740967 1.00109037106827 1.00578701948704 0.00407054682947679

PBK 1.032646349542 1.01085543642837 1.05490700726719 0.00315549098833149

TK1 1.00891735653161 1.00426674026502 1.01358950913989 0.000165786502997927

MS4A2 0.777386139756359 0.661698692285546 0.913299689618405 0.0021902674054988

SPC25 1.07884393030378 1.03078444409891 1.12914415095851 0.00109838418180446

LYPD3 1.01176207692485 1.00571841004299 1.01784206203357 0.00013060143438867

TICRR 1.31169093316012 1.12628471503095 1.527618266654 0.000483957569650584

LINC02323 1.38983192451712 1.20810957660686 1.59888872318373 4.13795179672361e-06

SNX30 0.941210620010121 0.90811801905805 0.975509143777058 0.000907448880973119

Z97832.1 0.013838880929188 0.000861998641132152 0.222175089650613 0.00251063147327723

LINC00941 1.12123040171785 1.07616591213633 1.16818196855981 4.57415148434013e-08

NKAIN1 1.09763777614489 1.02365183803676 1.1769711564539 0.00888312505122964

CENPE 1.18290455763664 1.07396707825408 1.30289207258782 0.000655357215603164

LINC01117 1.79678793743614 1.47513686308699 2.18857447936047 5.78806522301831e-09

TM4SF19 1.11807433666106 1.0423776991112 1.19926800368617 0.00180637114335558

CDKN3 1.0343100400551 1.01184695313644 1.05727180938057 0.0026018280045965

SGO1 1.18395958161749 1.06677856798386 1.314012422984 0.00149507147866778

ZNF488 1.21867156771429 1.04854094480305 1.41640667187696 0.00994264830306456

HMMR 1.06083068739381 1.03043664566819 1.09212124010465 6.8485741810674e-05

RPL32P1 0.648106699134694 0.483561562043206 0.868642850123264 0.00370352120771823

RIC3 0.647694923622303 0.467863217231293 0.896648205363646 0.00886114015210385

CDC25C 1.19076642696594 1.09665977172611 1.29294856996302 3.23038078104173e-05

MYEOV 1.00975635965047 1.00306941132834 1.01648788642087 0.00418332380591981

ARNTL2 1.04655709899599 1.03073971733357 1.06261720882581 4.72628001603863e-09

KIF18A 1.23086703494813 1.13026388843724 1.34042472136021 1.80040698022767e-06

ACOXL 0.765726480923235 0.641199122906704 0.914438311969422 0.00320140584195799

AC123595.1 0.477815856641518 0.303693212359021 0.7517717998523 0.00140372443168148

SERPINB5 1.01998426466465 1.00915963575149 1.0309250026521 0.000278037117436913

AC091133.5 1.39011956004557 1.153233642693 1.67566425369686 0.000548881742651875

FAM83A 1.01010444987523 1.00719512100618 1.0130221824729 8.3966767449891e-12

LINC01843 1.12544595170569 1.07062776449687 1.18307093484163 3.50689888354152e-06

AL138789.1 1.14751107969025 1.05740990939104 1.24528970867146 0.000973977521140331

LINC00519 1.18891144302469 1.07540085444861 1.31440328832526 0.000725290009191576

SIGLEC17P 0.586321605664699 0.413395689588349 0.831583477833434 0.00275072265709112

SFTPC 0.99957666773691 0.999258104136534 0.999895332895398 0.00922537662566571

IL1R2 1.02372545553382 1.00789760266505 1.03980186631738 0.00318324280633091

FAM83F 1.086532607382 1.04841286200644 1.1260383668367 5.25055936679653e-06

LINC02561 1.12216908617346 1.02887916566777 1.22391773493254 0.00924464773275694

EPHA5 1.08561750922056 1.02108604120514 1.15422729208524 0.00860554961474054

KIF23 1.05151128283569 1.01470468532451 1.08965297383755 0.00572827547448522

SLC7A5 1.00730231128712 1.00392736866911 1.01068859958414 2.14673457379347e-05

FAM83B 1.13884499809283 1.0685072481221 1.21381294507873 6.41200188455749e-05

TH 2.06765413688693 1.37233691946264 3.11526533255377 0.00051390289817227

CRYBG2 1.09126068537309 1.03876367423231 1.14641078907677 0.000516875243062865

AMPD1 0.630809828631467 0.455871332637367 0.872880155889503 0.00542928595630613

AL355607.2 4.1117256186887 1.50975256159443 11.1980519148955 0.00567774112164296

HJURP 1.04972583374028 1.02086872270411 1.07939865480735 0.000644422456747269

HMGA2 1.04331382993365 1.01556473550215 1.07182113525497 0.0020498988570149

MAPRE3-AS1 3.95995975351901e-05 2.17937735470434e-08 0.0719530338132639 0.00811470411334542

CX3CR1 0.842444434705273 0.751651472296081 0.944204397548663 0.00321145897752415

ADGRF5 0.993205610401035 0.988551386408519 0.997881747064222 0.00444405039840435

KIF14 1.14880383136773 1.0661797230241 1.237830934565 0.000269794435225116

FAM72B 1.49330622688034 1.11984467811276 1.99131498396518 0.00631810023922429

FAM72A 2.32250630160092 1.45855758313838 3.69819853760567 0.000384912187014291

AC080023.1 3.95840671988697 2.16048350951768 7.25253568982084 8.45056532931024e-06

DLGAP5 1.04465717096331 1.02242208287536 1.06737581584308 6.88984815983129e-05

PRDM16 0.867804606030032 0.788749118357179 0.954783741394829 0.00362125117773417

CENPF 1.03762025618237 1.01345879726656 1.06235773861144 0.00212559753426348

C1QTNF7 0.662989317390507 0.51305185756008 0.856745431279249 0.00167829208143245

CEP55 1.02779218351185 1.00894806461644 1.04698825393915 0.00369016201869491

LINP1 1.0608365560821 1.02242983856183 1.10068598966467 0.00169556991201476

ELDR 9.25105961967832 3.46632998945434 24.6895432192579 8.91459355087699e-06

TRPA1 1.96816918045722 1.60752770107244 2.40971892448096 5.50456719197131e-11

MELTF 1.04755030869414 1.03066768176284 1.06470947780983 2.09642737644235e-08

KRT6A 1.00161985059017 1.00084101905121 1.00239928819798 4.53976110874983e-05

LINC01116 1.11158478295451 1.07609838715475 1.14824141030734 1.65361940851875e-10

PITX3 6.40883256309123 3.85568900661211 10.6526057343583 7.75105712314535e-13

TMPO-AS1 1.29010462626625 1.10621957422608 1.50455658667763 0.00116784616852696

DEPDC1-AS1 19.982454295 2.17432225089985 183.64273257403 0.00813830729754396

AC245100.3 1.17319296279657 1.0670376550933 1.28990923739711 0.000963874888767279

RAD51 1.10401727306011 1.04079251269288 1.17108273200534 0.00100624347254369

CHRDL1 0.958049121270352 0.92923845365239 0.98775305214634 0.00594223028589931

NCAPH 1.03755183353474 1.01051087005124 1.06531640497515 0.00621916132206008

RRM2 1.02440486960462 1.01279811399806 1.0361446396529 3.36397213290097e-05

HOXA1 1.06121512972753 1.02034043484064 1.10372725916572 0.00302923266223253

KIF20A 1.05417920950315 1.02709039563311 1.08198247250056 7.11411361617659e-05

TYMS 1.02374761293153 1.01114859698751 1.03650361391537 0.000203390003299217

NFIX 0.979041518030202 0.963594895217122 0.9947357533592 0.00904189041752473

HLA-DQB1-AS1 0.925851586329316 0.889540106422385 0.963645319328054 0.000160603486035914

UNC45B 0.00407699133484223 0.000142357848342565 0.116761095632608 0.0013059219476221

MIR3189 0.970917250235954 0.95047576989486 0.991798356848195 0.00655726598064272

LRP2BP 0.534118848760502 0.359498018014099 0.793559158342997 0.00190496742833686

HOXD8 1.12244022424007 1.06792980622647 1.17973302144627 5.43009807024278e-06

PKP2 1.0584954188015 1.03490477282663 1.08262381336168 7.67481171641991e-07

UBE2SP1 1.16555000584811 1.05135549355965 1.29214792185366 0.00359251592817142

ZDHHC11B 0.912428192573812 0.8531718230879 0.975800166009164 0.00747272662939759

AC103681.2 0.0260408670078505 0.00177289077713557 0.38249776199761 0.00779204771939392

MKI67 1.03684219375928 1.01641727761601 1.05767754881248 0.000365073560446006

NDC80 1.0494112760515 1.02087338518632 1.07874692619501 0.00060682523314576

HAGLR 0.976925823942226 0.960805955921659 0.993316142144121 0.00596032013791648

SHCBP1 1.10959635415865 1.05366586791674 1.16849573157045 8.11630768660843e-05

VEGFD 0.892976325584253 0.835254495861899 0.954687130694352 0.00089997359704416

BTG2 0.993083606837657 0.989107181663707 0.997076018102353 0.000697805606078606

LINC01446 1.42919553518958 1.12587571109772 1.81423211964871 0.00334539647785608

SEC14L6 0.910596599545222 0.848758114612241 0.976940488494933 0.0090501618512507

VIPR1 0.866324819477453 0.788505589875935 0.951824188032364 0.00280673746277146

PLK1 1.04895334614922 1.02545498592379 1.07299017265631 3.55784624141706e-05

MAD2L1 1.04663756068328 1.0123546031642 1.08208149595915 0.00730543035863663

ATP1A2 0.553252932596166 0.370009532372629 0.827245734626108 0.00392699725186595

PLK4 1.10520506164264 1.02715088570459 1.18919064889148 0.00743210637325062

STC2 1.05929036672424 1.03140674599476 1.08792780868673 2.31569933118467e-05

RGS20 1.26155606544183 1.14372773918798 1.39152321983816 3.41200337325761e-06

CDCA4 1.04204755385112 1.02056511014876 1.06398219348183 0.000106503839727677

LRRC55 1.05688209735472 1.02030926677729 1.09476587548501 0.00207753998009276

CC2D2B 0.00115846800001058 9.81927017286896e-06 0.136674934432159 0.00547590233211133

KIAA0408 0.0113271268011008 0.000401321254658371 0.319703479641105 0.00856094188523509

SKA3 1.09987616510098 1.0419558449616 1.16101616436713 0.000562676036254194

ANLN 1.03834900693465 1.02550680926359 1.05135202464077 3.09176124622398e-09

HASPIN 1.26686944757045 1.09552557739667 1.46501207301922 0.00142023740278324

AC099850.3 1.03744555941863 1.01510960362213 1.06027298423441 0.000931526221961334

AC112777.1 1.51801696216002 1.19503001851561 1.92829925750978 0.000626984031160423

ASPM 1.09298780599903 1.03755752570991 1.15137938327343 0.000812756739274043

GABRA3 1.08855171382489 1.02544575229446 1.15554121807005 0.00535911738327733

SLC15A1 1.08026660680595 1.03545869790421 1.12701351018831 0.000354177841257885

CXCL5 1.01167915165654 1.00757638069351 1.01579862877694 2.1385906915927e-08

FSCN1P1 613.073713072919 34.8328818733676 10790.361217525 1.15216118349802e-05

SLC34A2 0.999204540852246 0.998759106589794 0.999650173772892 0.000468861440136903

KCNG1 1.27914713802104 1.14747964808176 1.42592280694712 8.90676311054754e-06

CLCN1 1.4146276393277 1.14569129592715 1.74669334144712 0.00126331970076125

MANCR 1.22855287640783 1.0866577934962 1.38897652891608 0.00101205743418132

MYO16-AS1 1.08083732731606 1.026144021485 1.13844577725955 0.00334556021673661

CCR6 0.0636203639628486 0.00980119759147153 0.412964913010966 0.00389315954548795

DKK1 1.00853696578136 1.00461707823512 1.01247214822822 1.88275818464068e-05

POPDC3 1.05790029979575 1.0162140209728 1.10129659816797 0.00606766420157937

BLM 1.1198096029966 1.04087728793523 1.20472755193928 0.00241144149609605

MFAP4 0.99387428797424 0.989760035175969 0.998005642974545 0.00369360227431464

HOXA13 1.16708813740049 1.09793799940272 1.24059347722907 7.1144694256049e-07

AC018647.1 0.0106783732922383 0.00110012476004999 0.103649749836743 9.0515597979549e-05

KPNA2 1.01026129956554 1.00541065626218 1.01513534498853 3.21895745947506e-05

CPA3 0.984052644071159 0.972467506344042 0.995775797120412 0.0078013763541891

AC133785.1 1.60484913855494 1.23084748675833 2.09249381846951 0.000475374972081614

BCL2L10 1.10126114723372 1.04872439811027 1.15642976991084 0.000109944808565513

KIF11 1.03847838598959 1.01404320297465 1.06350237840361 0.00188444634011216

UBE2SP2 2.45745848774449 1.29535607157903 4.66211750690744 0.0059222209019062

SUSD2 0.994873153819535 0.991214572079083 0.998545239417506 0.0062485497370086

ECT2 1.03512030255576 1.02102397854032 1.04941124134511 8.05571251413547e-07

IGF2BP1 1.05503966239129 1.0283561605718 1.08241554035112 4.14350344481787e-05

LINC01524 47.6878378360065 3.99803646556316 568.811689703516 0.00224555671159747

ASF1B 1.02244919776956 1.00660280437462 1.03854505220567 0.00534030815632944

GTSE1 1.08017680034108 1.02654086050907 1.13661517517819 0.00299714704259833

EGLN3 1.00873530672426 1.00277151779797 1.01473456412739 0.00404312211204516

SLC16A1 1.01558084412064 1.00652434198504 1.02471883482787 0.000717274137752735

TPPP 0.935720971998785 0.891621349088147 0.982001763791091 0.00698967076742256

RAB3B 1.05647838962722 1.01363660169193 1.10113090419809 0.00928875807907144

RACGAP1 1.0258663502524 1.00679935379969 1.04529444184523 0.00763305387478171

AC003957.1 0.000106433907347363 1.06529185504817e-07 0.10633871440531 0.00943353841902762

OIP5 1.0840810669901 1.03338131247211 1.137268252892 0.000954341340695181

AC020779.1 0.000248011685226999 6.78467560050887e-07 0.0906598924265779 0.00582876801041902

UCK2 1.0598858295591 1.03321278538296 1.08724745530883 7.73302977303341e-06

NUSAP1 1.01986577701683 1.00606848423077 1.03385228683056 0.00464689339520613

LHX2 1.19468311800107 1.05287417333236 1.35559194876956 0.00579495175677106

CKAP2L 1.11232781151091 1.05052071188369 1.17777131499111 0.00026257892602074

ADAMTS8 0.79499956876228 0.692125588192645 0.913164207644198 0.00117541451220021

PPP2R2C 1.09801917335386 1.05078726496146 1.14737411201583 3.06894904210442e-05

CD109 1.05011972718362 1.02801658845969 1.07269810020527 6.61365864330527e-06

LAMC2 1.00266245886845 1.00164034419799 1.00368561654643 3.22836181531469e-07

HMGA1 1.00188133448145 1.00092044416943 1.0028431472546 0.00012343901865756

MBL1P 0.740753907412466 0.603320617271644 0.909493784297078 0.00415696243464087

CDC20 1.0094889686924 1.00265109418233 1.01637347610209 0.00646031230695874

AC011284.1 0.0788422603577567 0.011744676425808 0.529269755330242 0.00892521325083042

GJB3 1.02886692339201 1.01920578610611 1.03861963941002 3.37872167520751e-09

CCNB1 1.01675959924352 1.00833942391874 1.02525008755103 8.95349636935462e-05

NCAPG 1.04910513680793 1.01436522288755 1.08503482103191 0.00526890232457043

CYP4B1 0.994353777926068 0.990685915379549 0.998035220170705 0.0026730009738571

COL4A3 0.908202195676134 0.853548401933772 0.966355541598155 0.00236015060332405

ITGA8 0.874724303885647 0.805447639539753 0.949959463839817 0.00147578457314529

COL6A6 0.783936992692121 0.654345806065011 0.939193317684547 0.00827994865101043

DEPDC1B 1.06872553550123 1.01756794255679 1.12245504448825 0.00791118287302045

DTL 1.10267936707755 1.05254366749626 1.1552031750577 3.83998572518379e-05

AL353746.1 11.7875888013338 5.09103415622588 27.2925392927113 8.44574971710363e-09

DIAPH3 1.23100021158867 1.11315856173222 1.36131686268778 5.16539134159521e-05

BUB1B 1.04986271223834 1.01554304670667 1.08534219019354 0.00411112524330965

ORC1 1.07794171297312 1.02681791970098 1.1316108866758 0.00246595225789295

ADGRF5P2 0.149037015272802 0.0414992113107713 0.53523985685145 0.00352118250407335

CDCA2 1.15007036603452 1.06807715878565 1.23835795565047 0.000211223399014497

LINC02253 1.10875771471219 1.04043195632526 1.18157046451721 0.00146592483286638

BUB1 1.04183898818336 1.01287284939271 1.071633500641 0.00438499541453333

UHRF1 1.06158177467731 1.01980797739512 1.10506672756728 0.00352779607805645

FOSL1 1.01327353784926 1.00585762111642 1.02074413013441 0.000434279216832473

LINC02732 1.15267293168604 1.03554763603278 1.28304564774229 0.00935272359403282

KLRC2 1.35217149823743 1.15931096766875 1.57711590042343 0.000121666804453013

CLSPN 1.09304660667389 1.02839030308668 1.1617679404165 0.00423863091767419

LINC01322 1.63511655363316 1.3277029149298 2.01370812242777 3.70095050791238e-06

SGO2 1.16168593780492 1.07780650272634 1.25209322330127 8.87334431099123e-05

SCN4B 0.76546205920714 0.647398800554942 0.905055992663843 0.00176512305581269

ITGA9 0.944464043783179 0.904289635613744 0.986423259616222 0.00998548519437034

FANCI 1.06962188408246 1.03021170329677 1.11053967960848 0.000441541227451889

NUS1P2 1.20921208677564 1.05323146443397 1.38829300128259 0.00701796499085658

SCN7A 0.875026748135576 0.802286910927981 0.954361587511244 0.00257072180579294

AC079949.2 1.29314702527299 1.17297719933485 1.42562807693163 2.39074832085531e-07

FHL1 0.957340573670387 0.928826572239107 0.9867299250345 0.00471502663747254

KYNU 1.05649878713239 1.03630716067519 1.07708383148195 2.37374826003598e-08

CAV3 0.00791428372641314 0.000357698552575369 0.175108024483745 0.00219333839471153

ICAM5 0.908406444978611 0.85298510988363 0.967428692150625 0.00278096455226577

ERVFRD-1 0.0411094327971991 0.00395691312173025 0.427096934634856 0.00753322157189945

AC009275.1 1.19767746989814 1.05961572794587 1.35372785064483 0.00389410230947063

CLEC3B 0.955620161357068 0.933270871466466 0.978504655735333 0.000170154970270092
