# Supplementary material for: Characterization of polyamine metabolism predicts prognosis, immune profile, and therapeutic efficacy in lung adenocarcinoma patients
Source: Front Cell Dev Biol. 2024 Apr 8;12:1331759. doi: 10.3389/fcell.2024.1331759 (PMC11033315; doi:10.3389/fcell.2024.1331759)
Supplement: Supplementary file 13 [file Table6.DOCX]

id coef HR HR.95L HR.95H pvalue

PSMC6 0.422955249542883 1.52646598438741 1.0685538626274 2.18060921679932 0.0201067659615184

SMOX 0.426295382386244 1.53157310804373 1.05330925628043 2.22699665012554 0.0256228344034142

SMS 0.363200292708783 1.43792383606057 1.0477421766096 1.97341006639801 0.0245306172228401
